# Towards DNA‐Encoded Micellar Chemistry: DNA‐Micelle Association and Environment Sensitivity of Catalysis

**DOI:** 10.1002/chem.202100980

**Published:** 2021-06-07

**Authors:** Mateja Klika Škopić, Christian Gramse, Rosario Oliva, Sabrina Pospich, Laura Neukirch, Magiliny Manisegaran, Stefan Raunser, Roland Winter, Ralf Weberskirch, Andreas Brunschweiger

**Affiliations:** ^1^ Medicinal Chemistry Faculty of Chemistry and Chemical Biology TU Dortmund University Otto-Hahn-Straße 6 44227 Dortmund Germany; ^2^ Polymer Hybrid Systems Faculty of Chemistry and Chemical Biology TU Dortmund University Otto-Hahn-Straße 6 44227 Dortmund Germany; ^3^ Physical Chemistry – Biophysical Chemistry Faculty of Chemistry and Chemical Biology TU Dortmund University Otto-Hahn-Straße 4a 44227 Dortmund Germany; ^4^ Department of Structural Biochemistry Max Planck Institute of Molecular Physiology Otto-Hahn-Straße 11 44227 Dortmund Germany

**Keywords:** copolymers, DNA conjugates, heterocycle synthesis, micellar catalysis, partitioning

## Abstract

The development of DNA‐compatible reaction methodologies is a central theme to advance DNA‐encoded screening library technology. Recently, we were able to show that sulfonic acid‐functionalized block copolymer micelles facilitated Brønsted acid‐promoted reactions such as the Povarov reaction on DNA‐coupled starting materials with minimal DNA degradation. Here, the impact of polymer composition on micelle shape, and reaction conversion was investigated. A dozen sulfonic acid‐functionalized block copolymers of different molar mass and composition were prepared by RAFT polymerization and were tested in the Povarov reaction, removal of the Boc protective group, and the Biginelli reaction. The results showed trends in the polymer structure‐micellar catalytic activity relationship. For instance, micelles composed of block copolymers with shorter acrylate ester chains formed smaller particles and tended to provide faster reaction kinetics. Moreover, fluorescence quenching experiments as well as circular dichroism spectroscopy showed that DNA‐oligomer‐conjugates, although highly water‐soluble, accumulated very effectively in the micellar compartments, which is a prerequisite for carrying out a DNA‐encoded reaction in the presence of polymer micelles.

## Graphical abstract

**It′s a matter of environment**: Copolymer micelles attracted DNA oligonucleotide conjugates in aqueous solution despite lacking canonical DNA‐binding motifs. Following accumulation inside micelles, DNA‐coupled starting materials underwent Povarov and Biginelli reactions to target molecules by a localized sulfonic acid. The kinetics of a given reaction were tuned by the chemical environment within the micelles.

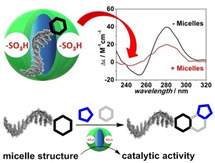

## Introduction

DNA‐encoded chemistry combines the highly efficient combinatorial approach to small molecule screening library synthesis with a selection‐based technique for compound identification from complex encoded compound mixtures.^[1]^ For a long time, encoded library design relied mainly on a few robust reactions that included carbonyl chemistry and sp^2^ cross‐coupling reactions.^[1]^

Current research in this field aims at diversifying the scope of encoded compound classes.[^1^, ^2^] The choice of reactions for DEL synthesis reflects the need to preserve the integrity of the genetic information during multistep library synthesis. This implies a pH, temperature and redox‐potential range that avoids nucleobase deamination, depurination, 8‐oxopurine formation, and phosphoribose backbone fragmentation (pH 4–11, <100 °C, oxidation potential <1.2 V).[^3^, ^4^] Furthermore, a reaction for DEL synthesis needs to yield products in aqueous co‐solvents or moist solvents, and it must provide defined compound mixtures with little and/or well‐characterized side‐product formation to obtain meaningful screening data for compound identification. DNA‐coupled starting materials are highly diluted, thus high product yields demand reactions with fast kinetics and/or large excess of starting materials. Some of these challenges have been met with fast radical‐based reactions,[^4^, ^5^] reversible immobilization of DNA on ion‐exchange matrix,[^6^, ^7^, ^8^] or solid phase chemistry.[^9^, ^10^, ^11^] We and the Waring research group are investigating the potential of micellar catalysis for DNA‐encoded library synthesis.[^12^, ^13^] Micelles designed as nanoreactors hold much promise for encoded chemistry as they accelerate reactions under mild conditions and in aqueous systems.[^14^, ^15^, ^16^, ^17^, ^18^] They are formed in water from amphiphilic molecules that assemble reversibly into spherical structures with a hydrophobic core and a hydrophilic shell or corona. Such systems have been applied in the context of DNA‐based technologies such as nanotechnology,[^19^, ^20^, ^21^] drug delivery,[^22^, ^23^] sensor technologies,[^24^, ^25^] and in controlling chemical reactions.^[26]^ For instance, DNA lipid conjugates or DNA polymer conjugates formed micelles that recruit oligonucleotides to the shell in a sequence‐programmable manner for bioconjugation reactions.[^27^, ^28^] Micelle‐ or nanoparticle‐forming polymers were chemically modified with quaternary ammonium salts to recruit DNA oligonucleotides by Coulomb interactions or with polycyclic heteroaromatic structures that intercalate into a DNA duplex.[^29^, ^30^]

**Figure 1:**
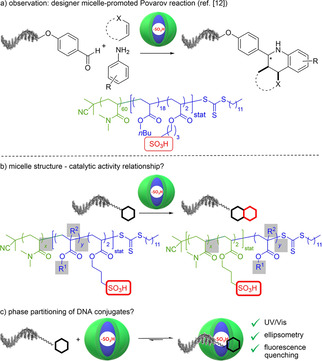
Investigations into micellar catalysis for the reaction of DNA‐tagged starting materials. a) Previously, we observed efficient micelle‐promoted reactions of DNA‐tagged aldehydes. In this study, we investigated b) the structure sensitivity of polymer micelle catalytic activity for Povarov and Biginelli reactions as well as for the removal of Boc groups and c) the partitioning of DNA conjugates between the aqueous bulk phase and block copolymer micelles.

**Figure 2:**
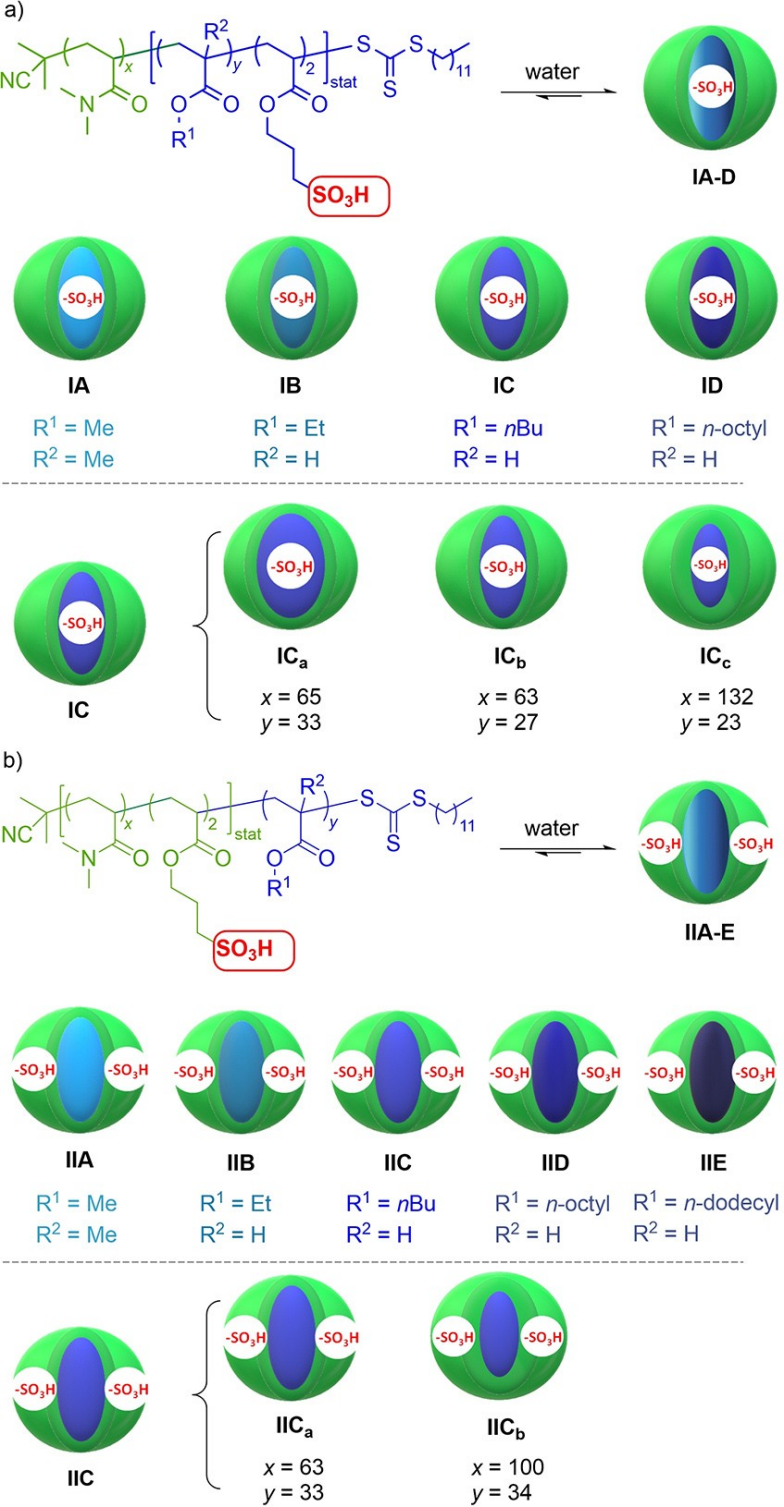
Copolymer structures and a schematic depiction of the formed micelles. Copolymers differed with respect to positioning of the catalyst, lipophilicity of the micellar core, and degree of polymerization in the core and in the corona. a) General structure of the copolymer series **I** consisting of a *N*,*N*‐dimethylacrylamide portion and a lipophilic acrylate ester portion co‐polymerized with a sulfonic acid moiety and schematic depiction of the formed micelles. b) General structure of the copolymer series **II** consisting of a *N*,*N*‐dimethylacrylamide portion co‐polymerized with a sulfonic acid moiety, and a lipophilic acrylate ester portion. Both copolymer series **I**/**II** contained copolymers of different core lipophilicity indicated by different shades of blue: **I**/**IIA** (methyl metacrylate), **I**/**IIB** (ethyl acrylate), **I**/**IIC** (*n*‐butyl acrylate), **I**/**IID** (*n*‐octyl acrylate), **IIE** (*n*‐dodecyl acrylate). Each copolymer series contained copolymers with different degrees of polymerization in the core: **IC_a_** vs. **IC_b_** (**IC_a_** had a larger hydrophobic core than **IC_b_**), and in the corona: **IC_b_** vs. **IC_c_** (**IC_c_** had a larger hydrophilic corona than **IC_b_**), and **IIC_a_** vs. **IIC_b_** (**IIC_b_** had a larger hydrophilic corona than **IIC_a_**).

We recently discovered that amphiphilic sulfonic acid‐substituted block copolymers assembling to micellar nanoreactors facilitated Brønsted acid‐promoted heterocycle‐forming reactions on DNA‐coupled aldehydes.^[12]^ Our approach used amphiphilic block copolymers that were built from a water‐soluble poly(*N*,*N*‐dimethylacrylamide) block and a hydrophobic poly(*n*‐butyl acrylate) block. The RAFT‐polymerization technique allowed sulfonic acid moieties to be precisely located in the core or shell of the copolymer micelle. These polymer micelles enabled the synthesis of DNA‐tagged heterocycles by, say, an acid‐promoted Povarov reaction (Figure 1a).^[12]^ Following this empirical work on micellar catalysis in the context of DNA‐encoded chemistry, we wished to gain a better understanding of this system for future reaction design. Here, we explored how a systematic variation of amphiphilic block copolymer composition (Figure 2) affecting key physicochemical parameters impacted the micellar catalytic activity in the on‐DNA Povarov reaction, Biginelli reaction and removal of the Boc group. Furthermore, we explored whether DNA could be directed to copolymer micelles with lipophilic pyrene anchor groups and we carried out fluorescence quenching, UV/Vis absorption and ellipsometry experiments of reporter group‐ and substrate functionalized DNA 14‐mer oligomers (Table 1) with one block copolymer micelle to gain insight in underlying forces driving micellar catalysis (Figure 1b). The results provide evidence for an unexpectedly strong interaction of the DNA oligomer conjugates with the polymer micelles.

**Table 1:** DNA oligonucleotides designed for characterization of DNA‐copolymer micelle interaction and reaction kinetic studies.

DNA	Sequence (5‘‐3‘) counterstrand (3‘‐5‘)
**DNA‐1**	ald‐link‐GTC TTG CCG AAT TC
**DNA‐2**	ald‐link‐GTC TTG CCG AAT TC GAA TTC GGC AAG AC
**DNA‐3**	ald‐link‐GTC TTG CCG AAT TC fluo‐GAA TTC GGC AAG AC
**DNA‐4**	ald‐link‐GTC TTG CCG AAT TC GAA TTC GGC AAG AC‐pyr
**DNA‐5**	ald‐link‐GTC TTG CCG AAT TC pyr‐GAA TTC GGC AAG AC
**DNA‐6**	link‐GTC TTG CCG AAT TC GAA TTC GGC AAG AC
**DNA‐7**	link‐GTC TTG CCG AAT TC fluo‐GAA TTC GGC AAG AC
**DNA‐8**	GTC TTG CCG AAT TC GAA TTC GGC AAG AC

ald: *para*‐(carbonylmethyleneoxy)benzaldehyde; link: 5’‐(C6)‐aminolinker; fluo: Alexa Fluor 430; pyr: pyrene.

## Results

### Design of block copolymers

The block copolymer design was based on three considerations to probe the structure sensitivity of micellar catalysis: i) localization of the sulfonic acid either in the core or in the shell, ii) micelle core polarity, and iii) degree of polymerization *dp* in the shell, and in the core. RAFT‐polymerization technique gave access to two series of copolymers.^[12]^ Series **I** directed a catalytically active sulfonic acid to the core and series **II** immobilized it in the corona (Figure 2 and Figure S1 in the Supporting Information). Both copolymer series were composed of shell‐forming *N*,*N*‐dimethylacrylamide (DMA) units and the following core‐forming acrylate esters: methyl methacrylate (MMA, **IA**, **IIA**), ethyl acrylate (EA, **IB**, **IIB**), *n*‐butyl acrylate (BA, **IC**, **IIC**), *n‐*octyl acrylate (OA, **ID**, **IID**) and *n‐*dodecyl acrylate (DDA, **IIE**). The log *P* values of these hydrophobic monomers range from 1.34 (MMA) to 4.64 (DDA) indicating that large changes in micellar core polarity can be achieved with these monomers. For the **IC** and **IIC** polymers, we additionally varied the degree of polymerization *dp*. In the copolymer series **I** the ratio of the hydrophilic versus the hydrophobic block was set to 3 : 1 to ensure an adequate hydrophobicity for micelle formation. The ratio of alkyl acrylate to 3‐sulfo propyl acrylate within the hydrophobic block was set to 10 : 1, which ensured sufficient core hydrophobicity as well. The series **II** copolymers were designed in the same manner but with 3‐sulfo propyl acrylate copolymerized during hydrophilic block synthesis.

### Structural characterization of block copolymers

**Figure 3:**
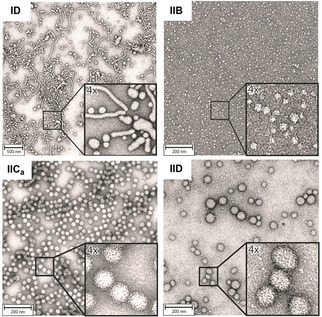
Copolymer structures and micelle shape. Representative transmission electron microscopy (TEM) images revealed that copolymer micelles **ID** (top left) assemble into spherical to highly elongated structures; copolymer micelles **IIB** (top right) formed small, well‐defined spherical structures; copolymer micelles **IIC_a_** (bottom left) assembled into homogenous, large spherical micelles; copolymer micelles **IID** (bottom right) formed spherical micelles of different diameters. The larger vesicles may be interpreted as liposome‐like structures. TEM images of all copolymer micelles and of selected reaction mixtures can be found in the Supporting Information.

Transmission electron microscopy (TEM) images and dynamic light scattering (DLS) analysis revealed that all copolymer designs led to formation of nano‐heterogeneous systems, yet, block copolymer composition had a profound impact on micelle shape and size.^[31]^ Most series **I** copolymers and all series **II** copolymers assembled into regular spherical micelles (Figures 3 and S3–S8) with an average diameter ranging from ∼10 nm (**IA**) to ∼100 nm (**IID**). Copolymers with longer alkyl acrylate units, that is, higher logP values, in the lipophilic polymer portion assembled into larger spherical structures that resembled liposomes (**IIC_a_**, **IIC_b_**, **IID**, **IIE**). Some micelles were highly homogeneous (**IC_b_**, **IC_c_**, **IIB**, **IIC_a_**), while others adopted a range of diameters and shapes (**IB**, **IC_a_**, **ID**, **IIA**, **IID**). For instance, polymer **ID** formed complex, highly elongated polymer sheets of micron length (Figure 3). This increasing complexity of aggregate formation from block copolymers with longer alkyl side chains has also recently been shown for other amphiphilic block copolymers with transitions from spherical to worm‐like to pseudo‐vesicle‐like nanostructures depending on the block copolymer composition and the method of preparation.[^31c^, ^31d^] In addition, zeta potential measurements were performed for selected copolymer micelles. They were between −19.01 and −25.39 mV for the core functionalized micelle IC_a_ and IC_b_ and −10.33 and −33.75 mV for the shell‐functionalized micelle **IIA** to **IIE**. Regardless of the localization of the sulfonic acid group, all micelles were negatively charged but the extent of surface charge depend also on the length of the hydrophilic block and the different hydrophobic monomers employed (Table S6). Moreover, fluorescence measurements with pyrene were carried out to analyze the micropolarity of the micellar core with and without sulfonic acid groups located in the hydrophobic block. The results did not show significant differences in the micropolarity experienced by pyrene and the I_1_/I_3_ values for both types of micelles were in the range of 0.87–1.04, thus suggesting a very nonpolar micellar core (see below).

### Impact of copolymer structure on the reaction kinetics of on‐DNA Povarov reaction, Boc protective group removal, and Biginelli reaction

**Figure 4:**
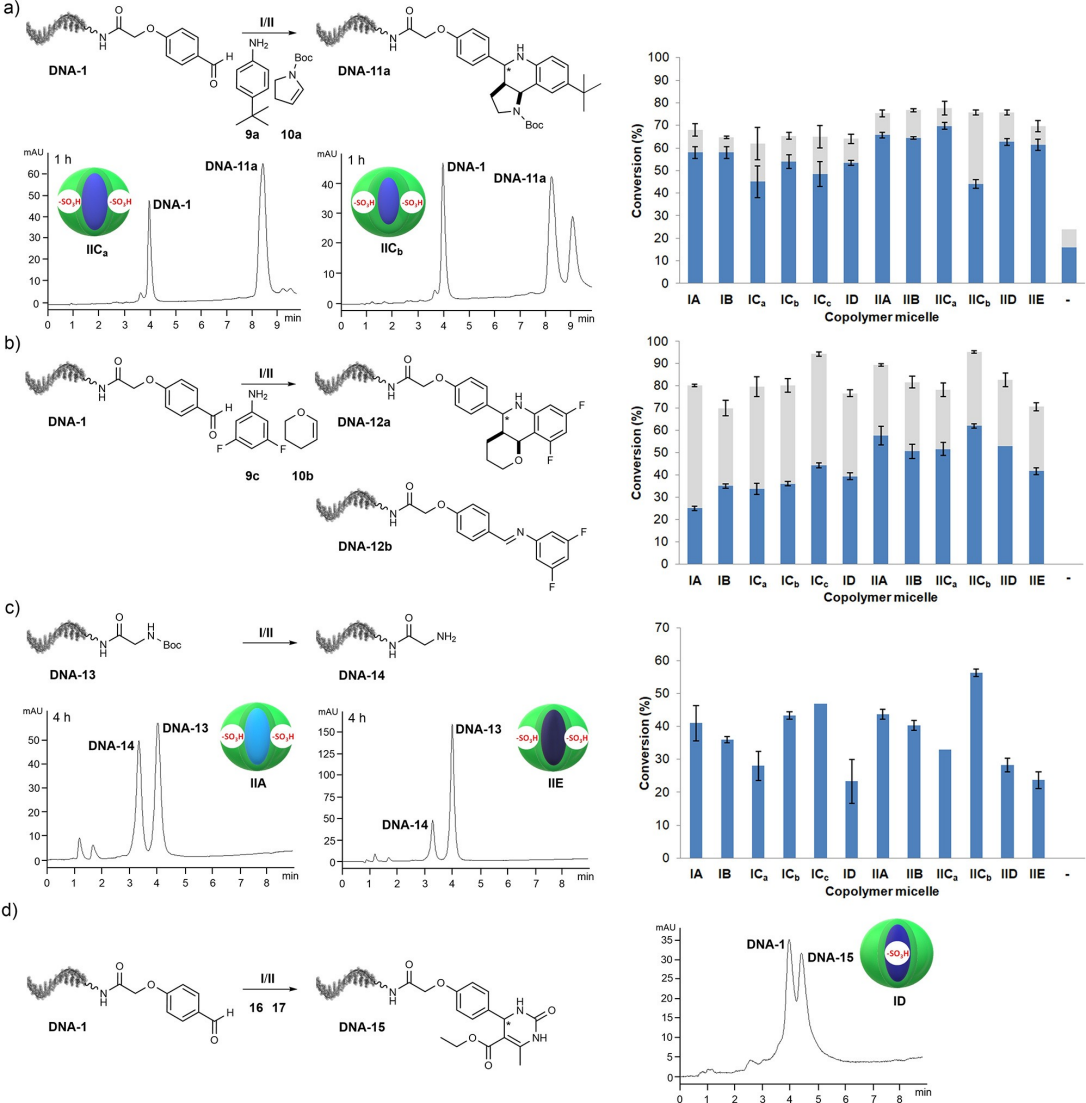
Investigations into the copolymer micelle structure‐catalytic activity relationships for three copolymer micelle‐mediated reactions: the Povarov reaction, Boc‐group removal, and the Biginelli reaction. a) Representative HPLC traces of an on‐DNA micelle‐mediated Povarov reaction to **DNA‐11 a** (left). Head‐to‐head comparison of micelles **IA‐ID** and **IIA‐IIE** in the micelle‐mediated Povarov reaction to **DNA‐11 a** (right). The blue portion of the bars indicates conversion to **DNA‐11 a**; the gray portion of the bars indicates conversion to the later‐eluting side product. Reaction conditions: 2000 equiv. aniline **9 a** and 2000 equiv. olefin **10 a**, 50 equiv. (0.5 mM) **I/II**, room temperature, 1 h. b) Head‐to‐head comparison of micelles **IA‐ID** and **IIA‐IIE** in the micelle‐mediated Povarov reaction to **DNA‐12 a**. The blue portion of the bars indicates conversion to **DNA‐12 a**; the gray portion of the bars indicates conversion to **DNA‐12 b**. Reaction conditions: 8000 equiv. aniline **9 c** and 8000 equiv. olefin **10 b**, 50 equiv. (0.5 mM) **I/II**, room temperature, 18 h. c) Representative HPLC traces of an on‐DNA micelle‐mediated Boc deprotection of DNA‐glycine conjugate **DNA‐13** (left). Head‐to‐head comparison of the micelles **IA‐ID** and **IIA‐IIE** in the micelle‐mediated Boc deprotection of **DNA‐13** (right). The blue bars indicate conversion to **DNA‐14**. Reaction conditions: 25 equiv. (0.25 mM) **I/II**, 50 °C, 4 h. d) Copolymer micelle‐mediated Biginelli reaction. HPLC trace of the micelle **ID**‐mediated Biginelli reaction (right). Reaction conditions: 8000 equiv. urea **16** and 8000 equiv. ethyl acetoacetate **17**, 25 equiv. (0.25 mM) **I/II**, 40 °C, 63 h. In (a)–(c) error bars represent the standard deviation of triplicates of one experiment.

Positioning of the catalyst, log *P* differences of the two amphiphile portions, and degree of polymerization in the core and corona can all impact reaction kinetics of a micelle‐promoted reaction. We studied these parameters with three model reactions (Povarov reaction, Biginelli reaction, and removal of a Boc protective group) and measured product formation and DNA degradation by depurination. In a first catalyst screen the ssDNA‐aldehyde conjugate **DNA‐1** was reacted with the lipophilic *tert*‐butylaniline **9 a** and *N*‐Boc‐pyrroline **10 a** by Povarov reaction to the heterocycle conjugate **DNA‐11 a** in the presence of all copolymers **IA**–**D** and **IIA**–**E**. Reaction progress was measured after 30 min, 1, 2, and 4 hours (Figures 4a and S9‐S26, Tables S8–S12). After 4 hours, more than 90 % of the starting material was converted either to **DNA‐11 a** alone (**IA**, **IB**, **IIB**, **IIC_a_**) or to a mixture of **DNA‐11 a** and minor amounts of a later‐eluting side product that we could not characterize. This side‐product could be an adduct of the pyrroline to the imine, that is, a Mannich‐type reaction product. We selected an early time point (1 h) at which we did not expect full consumption of the DNA‐conjugated aldehyde to perform reactions in triplicates for a head‐to‐head comparison of the catalytic activity of the copolymer micelles (Table S10). A first series of experiments showed the effect of catalyst positioning with core‐modified copolymers **IA**, **IB**, **IC_a_**, and **ID** and shell‐modified copolymers **IIA**, **IIB**, **IIC_a_**, and **IID**. Localization of the sulfonic acid in the shell provided higher conversion rates to **DNA‐11 a** for all copolymers: **IIA** (14 % higher than **IA** after 1 h), **IIB** (10 % higher than **IB** after one hour), **IIC_a_** (55 % higher than **IC_a_** after 1 h) and **IID** (14 % higher than **ID** after 1 h; Table S10, Figures S10–S21).

Next, we investigated the impact of copolymer log *P* on the reaction rate with the shell‐modified block copolymers **IIA**–**E**. As the catalyst was placed outside the variable core phase, it had a small impact on the reaction rate: The fastest reaction rates were observed with *n*‐butyl acrylate copolymer **IIC_a_**, and the slowest with the very lipophilic *n*‐dodecyl acrylate **IIE**. However, differences were minor as for instance, *n*‐butyl acrylate copolymer **IIC_a_** furnished approximately 13 % more product than *n*‐dodecyl acrylate copolymer **IIE** after 1 hour (Table S10, Figures S18 and S21). A more pronounced effect of core log *P* on reaction kinetics was evident in the Povarov reactions mediated by the core‐modified copolymers **IA**–**D** (Table S10, Figures S10‐S15). The least lipophilic methyl methacrylate and ethyl acrylate copolymers **IA** and **IB** showed the fastest reaction kinetics. For instance, the reaction promoted by methyl methacrylate copolymer **IA** gave 68 % higher product yields than the more lipophilic *n*‐octyl acrylate copolymer micelle **ID** at the first time point (30 min; Table S9, Figures S10 and S15).

As the last parameter we investigated the impact of the degree of polymerization *dp* on product formation (Tables S8–S12, Figures S12–S14 and S18–S19). Block copolymer **IC_b_** consisted of 63 DMA units in the corona and 27 *n*‐butyl acrylate units in the core. Increasing the degree of polymerization either in the core (25 to 33, **IC_a_**) or in the corona (65 to 132, **IC_c_**) led to lower product conversions. This impact was more pronounced with copolymer micelle **IC_a_** displaying a larger hydrophobic core (Figures S12–S14). Both copolymer variations yielded increased amounts of the later‐eluting side product. In a second example, increasing the degree of polymerization of DMA units in the corona of copolymer micelle **IIC** from 63 (**IIC_a_**) to 100 DMA units (**IIC_b_**) returned slower reaction kinetics (Figures S18 and S19) and pronounced side‐product formation. All these trends in micelle catalytic activity were generally consistent at all tested reaction times (Tables S9–S12), and they were noted at room temperature as well as at elevated temperatures (Table S13, Figures S27–S36).

We then explored a small substrate scope after 18 hours reaction time, again under conditions that would not give full substrate conversion, to compare micelle catalytic activity on substrates with different solubility and reactivity. The reaction of aldehyde **DNA‐1** with the more polar unsubstituted aniline **9 b** and *N*‐Boc‐pyrroline **10 a** to the heterocycle conjugate **DNA‐11 b**, gave similar micelle structure‐catalytic activity relationships as noted for the Povarov reaction to **DNA‐11 a** (Table S14, Figures S37–S51). However, the reaction involving aldehyde **DNA‐1**, polar 3,5‐difluoroaniline **9 c** and water‐soluble olefin dihydropyran **10 b** to **DNA‐12 a** (Figure 4b, Table S15, Figures S52‐S65) showed a much more pronounced environment sensitivity of micellar catalysis. In this reaction, we also detected imine intermediate **DNA‐12 b**. The effect of catalyst positioning was very clear with the shell‐modified copolymer micelles **II** giving higher yields of heterocycle **DNA‐12 a** than core‐modified micelles **I**, while micelles of series **I** furnished higher yields of the imine **DNA‐12 b** than copolymer micelles **II**. Overall, copolymer micelles **IIC_b_** and **IC_c_** displaying a very large hydrophilic corona demonstrated the highest conversions to the mixture of desired heterocycle **DNA‐12 a** and intermediate imine **DNA‐12 b**. As the last example, we reacted **DNA‐1** with lipophilic *tert*‐butylaniline **9 a** and dihydropyran **10 b** to DNA‐hexahydro‐1*H*‐pyrano[3,2‐*c*]quinoline conjugate **DNA‐12 c** and observed a pronounced environment sensitivity of micellar catalysis, too (Table S16, Figures S66–S70). Again, *n*‐butyl acrylate copolymer micelles exhibiting very large hydrophilic corona **IIC_b_** and **IC_c_** led to the highest consumption of **DNA‐1** and formation of **DNA‐12 c**, while reactions promoted by other copolymer micelles furnished in most cases only traces of the desired product (Table S16, Figures S66 and S67). In neither case did we detect the imine intermediate. Both Povarov reactions involving olefin **10 b** did not take place in the absence of copolymer micelles (Figures S64 and S70). The copolymer without covalently linked sulfonic acid moiety **III** (Figure S1c) did not mediate heterocycle synthesis (Tables S8 and S12, Figures S26 and S35).

Taken together, the micelle‐mediated Povarov reactions were sensitive to both copolymer environment and substrate reactivity and polarity. The most robust reaction outcome was observed with longer reaction times (18 h) and the corona‐modified micelle **IIC_b_** displaying a large hydrophilic shell.

Further exploration of the reaction to **DNA‐11 a** showed that the reaction was not abrogated by high‐salt conditions (200 mM NaCl), in line with a non‐ionic interaction of the DNA with the micelles, but proceeded with markedly slower reaction kinetics (Figures S71 and S72).

Next, we investigated the impact of micelle structure on removal of a Boc protective group from the DNA‐glycine conjugate **DNA‐13** at 50 °C and we monitored reaction progress after four and eight hours (Figure 4c and S73–S82, Tables S17, S18). Localization of the sulfonic acid in the shell provided slightly higher yields for all copolymers at both reaction times (Figures S75–S79). Corona‐modified block copolymers **IIA**–**IIE** demonstrated micellar catalytic activity inversely correlated to core lipophilicity (Figures S77–S79). The highest difference in product formation was detected at the first time point (4 h), where the methyl methacrylate **IIA** gave 83 % more of the deprotected glycine conjugate than *n*‐dodecyl acrylate copolymer micelle **IIE**. Among the core‐modified copolymer micelles, copolymers **IC_b_** and **IA** returned the fastest reaction kinetics, followed by **IB**, while lipophilic **ID** returned the slowest reaction kinetics (Figures S75–S77). *n*‐Butyl acrylate copolymer **IC_b_** gave 87 % higher yields of the deprotected amine than **ID** at the first time point (Table S17, Figures S76 and S77). The degree of polymerization had a partially similar impact on Boc‐group removal as observed in the Povarov reaction, with copolymer **IC_b_** superior to the copolymer displaying a larger hydrophobic core, **IC_a_**. Copolymer micelle **IC_c_** exhibiting a very large hydrophilic shell proved to be slightly superior to **IC_b_** (Table S17, Figure S76). In strong contrast to the Povarov reaction to **DNA‐11 a**, the shell‐modified copolymer micelle **IIC_b_** that contained a very large hydrophilic shell exhibited faster reaction kinetics for the Boc‐group removal than **IIC_a_** (Figure 4c, Table S17, Figure S78) and indeed the fastest reaction kinetics of all copolymers. The micellar reaction systems were not able to separate amine deprotection from DNA depurination, instead depurination and Boc‐removal proceeded in parallel (Tables S17, S17). Addition of *para*‐*tert*‐butylaniline or pyridine buffered the solution and protected the DNA against depurination but led to lower yields of the unprotected amine (42 % amine deprotection and 10 % depurination; Table S19, Figure S83).

Finally, we investigated the micelle‐mediated Biginelli reaction of **DNA‐1** to the pyrimidinone **DNA‐15** (Figures 4d and S84–S105, Tables S20 and S21). We set up two sets of experiments, each in triplicate: one series of Biginelli reactions was set at 40 °C for 21 hours and the other at 40 °C for three days. One‐day reactions did not yield the product (Figures S84–S95) while three‐day reactions provided pyrimidinone **DNA‐15** with at least 73 % conversion (Figures S96–S105). However, similar to Boc‐deprotection, the Biginelli reaction does not include a basic component (e. g., aniline) that would buffer the reaction mixture. The lack of the buffering effect was especially pronounced under more forcing reaction conditions (e. g., prolonged reaction time under elevated temperature) when formation of **DNA‐15** was often accompanied by severe DNA degradation (Figures S96‐S105), following the same trends observed in micelle‐mediated Boc‐deprotection (Tables S18, S20). In this reaction we observed that the most lipophilic micelles (**IC_a_**, **ID**, **IID**, and **IIE**) yielded less of the desired product **DNA‐15**, but significantly less DNA‐depurination. To confirm product formation, we compared the micelle‐mediated Biginelli reaction to **DNA‐15** with the previously reported Biginelli reaction on a controlled pore glass (CPG) solid support (Figure S110).^[10]^

Micelle **ID** was then selected to perform the Biginelli reaction on a small scope of aldehydes (Figures S112–S115). The ethoxy‐substituted benzaldehyde **DNA‐1 b**, 3‐phenylbenzaldehyde **DNA‐1 c**, and furan‐containing heteroaromatic aldehyde **DNA‐1 d**, also gave the desired DNA‐coupled pyrimidinones **DNA‐15 b**–**d** at 40–50 % conversion. The indole‐3‐carbaldehyde **DNA‐1 e** gave a lower conversion of only 23 %.

### Impact of DNA appendages on the reaction kinetics of copolymer micelle‐mediated Povarov reaction

**Figure 5:**
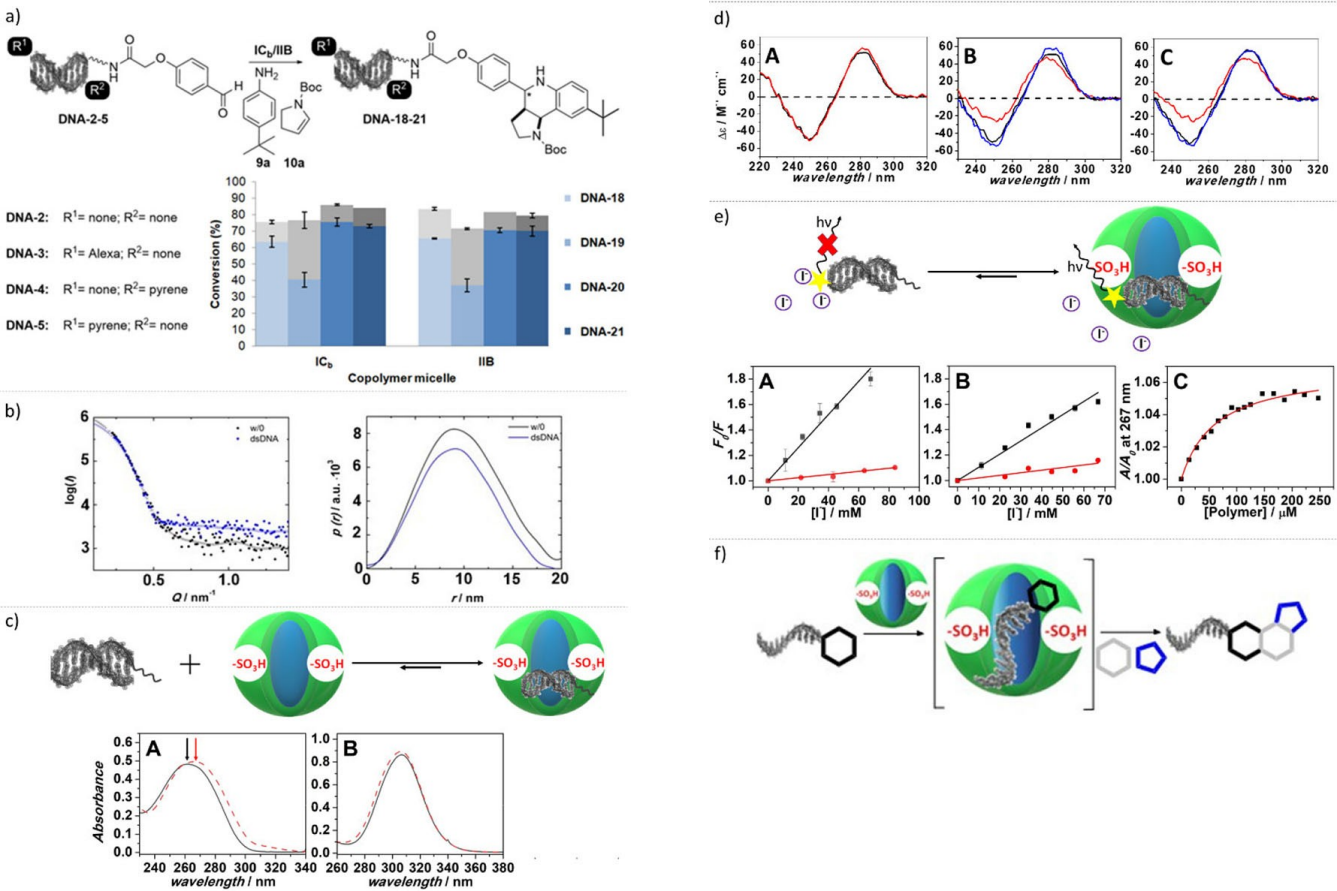
Investigations into the impact of DNA chemical modification on the micelle‐promoted Povarov reaction and characterization of copolymer micelle‐DNA interaction by SAXS measurements, circular dichroism (CD), UV/Vis analysis, and fluorescence‐quenching experiments. The blue portions of the bars indicate conversion to **DNA‐18‐21**; the gray portions indicate conversion to the corresponding later‐eluting side product. Error bars represent the maximum error of duplicates of one experiment. a) dsDNA **DNA‐2**, Alexa‐labeled dsDNA **DNA‐3**, 3’‐ and 5’‐pyrene labeled dsDNAs **DNA‐4** and **DNA‐5** were treated with **9 a** and **10 a** to give DNA‐hexahydropyrroloquinoline conjugates **DNA‐18‐21** with copolymers **IC_b_** and **IIB**. Reaction conditions: 2000 equiv. *tert*‐butylaniline **9 a** and 2000 equiv. *N*‐Boc‐pyrroline **10 a**, 50 equiv. (0.5 mM) **I**/**II**, room temperature, 1 h. b) left: SAXS curves [•] (background corrected) and their fits [x] of 6 mM copolymer **IIB** in the absence (black) and in the presence (blue) of 50 μM dsDNA **DNA‐2**. Right: The corresponding pair‐distance distribution functions, *p*(*r*). c) A: The absorption spectra of 5’‐aminolinker‐dsDNA **DNA‐6** in the absence (black) and presence (red) of 250 μM copolymer **IIB**. B: The absorption spectra of the S‐(C=S)‐S moiety present in copolymer **IIB** in the absence (black) and presence (red) of dsDNA. d) CD spectra of A: dsDNA conjugate **DNA‐6** (black) and dsDNA conjugate **DNA‐8** in water. B: dsDNA conjugate **DNA‐6** in the absence (black) and presence (red) of 250 μM copolymer **IIB** and with the additional presence of 20 mM NaCl (blue). C: dsDNA conjugate **DNA‐8** in the absence (black) and the presence of 250 μM copolymer **IIB** (red) and with the additional presence of 20 mM NaCl (blue). The spectra were normalized per mol of DNA. e) Fluorescence quenching experiments with Alexa‐labeled DNA conjugates in the absence and presence of copolymer **IIB**, the yellow star denotes the fluorophore. Stern‐Volmer plots show fluorescence quenching as a function of iodide concentration. Fluorescence of A: Alexa‐labeled benzaldehyde‐conjugated dsDNA **DNA‐3** and B: Alexa‐labeled 5’‐(C6)‐amino linker‐dsDNA **DNA‐7** in the absence (black) and presence (red) of 250 μM copolymer **IIB** as a function of iodide concentration. C: Binding isotherm of the 5’‐(C6)‐amino linker‐dsDNA **DNA‐6** to copolymer **IIB**, followed by absorption measurements at 267 nm. Here, *A*_0_ and *A* are the absorbance at 267 nm of the dsDNA in the absence and presence of different copolymer concentrations. f) Hypothesis for the copolymer micelle‐promoted reactions of DNA‐tagged substrates: DNA‐copolymer micelle complex formation preceded chemical reaction to target compounds.

The kinetics of micellar reactions on DNA‐conjugated substrates might be modulated by dyes that are appended to the oligonucleotide to gain insight into the reaction system or by a lipophilic pyrene designed to anchor the DNA oligonucleotide in a core‐shell structure (Figures 5a and S116–S120, Table S22). The Alexa‐labeled dsDNA **DNA‐3**, and the 3’‐ and 5’‐pyrene labeled dsDNAs **DNA‐4**,**5** were reacted to DNA‐hexahydropyrroloquinoline conjugates **DNA‐19**–**21** with copolymers **IB**, **IC_b_**, **IIB**, and **IIE** and product formation was compared head‐to‐head with the unlabeled **DNA‐2** that was reacted to **DNA‐18**. To our delight, **DNA‐3**‐carboxaldehyde being labeled with the charged, sulfonic acid‐substituted Alexa dye turned out to be a viable substrate, too. Positioning of a lipophilic pyrene anchor on the DNA oligonucleotide either proximal or distal to the reactive carbaldehyde had only a marginal beneficial effect (ca. 6–16 % higher product conversion) on the copolymer micelle‐promoted reactions after 1 hour (Figures 5a and S116‐S120).

### DNA oligonucleotide‐copolymer interactions

Not the least prompted by the small effect of the lipophilic pyrene anchor on the kinetics of the micellar reaction, we explored the interactions between DNA oligomers and the copolymer micelles. Recently, the research groups of O'Reilly and Tirrell could demonstrate that interactions of DNA oligomers and micellar aggregates can be controlled by electrostatic attraction forces between the anionic DNA oligomer and cationic micellar surface or by intercalation.[^29^, ^30^] However, the block copolymer system presented herein is composed of core‐shell like particles with a poly(*N*,*N*‐dimethylacrylamide) block that is neutral for the series **I** block copolymers and weakly negatively charged for the series **II** copolymers due to the sulfonic acid groups. Therefore, we labeled double‐stranded (ds) 14‐mer DNA‐oligonucleotides (Tables 1 and S7) that served as a proxy for an encoded compound with a fluorophore (**DNA‐3**,**7**) and studied their interactions with polymeric micelles based on block copolymer **IIB**. Interactions of unlabeled oligonucleotide conjugates (**DNA‐1,2,6**) with micelle **IIB** were also investigated (Tables 1 and S7).

Neither the form nor the size of the micelles was significantly affected by the presence of the dsDNA **DNA‐2** at a concentration of 50 μM (Figure 5b). The symmetrical shape of the curves of the pair‐distance distribution functions, *p*(*r*), obtained from the SAXS measurements confirmed the presence of spherical polymeric micelles with an average diameter, *D*, of about 20 nm, which corresponded to twice the maximum *r* value, both in naïve micelles and in the presence of DNA (Figure 5b).

UV/Vis spectra provided first evidence for the interaction between dsDNA linker conjugate **DNA‐6** with the copolymer micelles **IIB** (Figure 5c). Spectrophotometry offers the opportunity to follow both the DNA bases (absorbance: 240–280 nm) and the trithiocarbonate moiety of the RAFT polymer (270‐360 nm). The absorption spectrum of the dsDNA has a band centered at about 260 nm (Figure 5c, panel A, black line). Upon addition of the copolymer, we observed distinct batho‐ and hyperchromic shifts of the UV absorption by **DNA‐6** (Figure 5c, panel A, red dashed line). The noted red‐shift hinted at DNA bases experiencing a more hydrophobic environment.^[32]^ The shoulder at ∼310 nm can be explained by the absorption of the trithiocarbonate moiety of the inner copolymer portion.^[33]^ Figure 5c, panel B, shows the absorption spectra of the trithiocarbonate moiety in the absence and in the presence of dsDNA **DNA‐6**. Upon addition of **DNA‐6**, a slight increase of the absorbance coupled with a small, yet significant blue‐shift indicated that the chromophore was experiencing a more hydrophilic environment. These observations can be plausibly explained by the poly‐anionic dsDNA modifying the dielectric environment inside the micelles and were thus a first indication of the interaction between the copolymer micelle and the DNA oligomer.

CD spectroscopy provided further indication of the interaction between the polymer micelles and DNA oligomers as it reveals conformational changes of helical DNA upon external stimuli such as changes in the chemical environment (e. g., addition of NaCl). We used this technique to further confirm the interaction of DNA with copolymer micelle **IIB** and investigate its sensitivity to ionic strength. In Figure 5d, panel A, the CD spectra of desalted 14‐mer **DNA‐6** and 14‐mer **DNA**‐**8** in pure water are reported, showing that both oligonucleotides are helical. In Figure 5d, panel B, the CD spectra of 14‐mer **DNA‐8** in the absence and in the presence of copolymer **IIB** are shown. The addition of 250 μM of copolymer **IIB** to 14‐mer caused a dramatic loss of ellipticity, particularly pronounced in the negative band at 250 nm, (Figure 5d, panel B). This decrease of ellipticity can plausibly be ascribed to a reduced stacking of nucleobases, hinting at perturbation of DNA helical structure imposed by interaction with the polymeric micelle. Copolymer **IIB**‐DNA interaction differed markedly from the well‐described polyethylene glycol‐DNA interactions. The changes in CD spectra rather resembled those previously observed upon dsDNA binding to tryptophane‐oligomers.[^34^, ^35^] The addition of 20 mM NaCl and higher concentrations of salt restored the original conformation of the dsDNA, pointing out that the interaction is a reversible process (ruled by an equilibrium constant) and that it can be modulated by simple addition of sodium chloride. The same observations were also made for the 5’‐(C6)‐amino linker‐modified dsDNA **DNA**‐**6**: its interaction with copolymer **IIB** in desalted water was reversed by addition of NaCl (Figure 5d, panel C). Next, we used fluorescence quenching experiments to gain further evidence that a DNA conjugate does indeed associate with the copolymer micelles. Due to their size and charge, iodide ions are likely not penetrating the micelles, rendering I^–^ a selective quencher for fluorophores dissolved in the bulk aqueous phase.^[36]^ Figure 5e depicts the Stern‐Volmer plots for both an Alexa‐labeled benzaldehyde‐modified dsDNA **DNA‐3** that served as a surrogate for a DNA‐encoded starting material (panel A) and 5’‐aminolinker‐dsDNA **DNA‐7** (panel B) in the absence and in the presence of 250 μM copolymer **IIB**. For water‐exposed Alexa‐labeled dsDNA conjugate **DNA‐3** a value of *K*_SV_ was determined at ∼13.5 M^−1^. Notably, in the presence of copolymer **IIB**, the *K*_SV_ value dropped to 1.2 M^−1^, indicating that the labeled dsDNA or its appended fluorophore label partitioned into the iodide‐inaccessible micelles. This partitioning was not disturbed by increasing KI salt concentrations up to 100 mM. The same experiment was repeated for the Alexa‐labeled 5’‐(C6)‐amino linker‐modified dsDNA **DNA‐7**, which yielded a similar result (KSV of ∼10.4 and ∼2.0 M^−1^ in the absence and in the presence of copolymer **IIB** micelles, respectively). These results can be interpreted in two ways. Either KI, even at higher concentrations, does not disrupt the micelle‐DNA interaction, or the appended fluorophore remains in the Iodide‐inaccessible compartment of the micelle. We also verified the ability of pyrene labeled dsDNAs to interact with copolymer **IIB** in water. In Figure S121, fluorescence emission spectra of pyrene‐labeled **DNA‐9**, **DNA‐10**, **DNA‐11** and **DNA‐12** are reported (for oligonucleotide composition, see Table S7). Our data clearly shows that, independent of the positioning of the pyrene moiety on the DNA (5’ or 3’), and the presence of the amino linker, a strong quenching of pyrene fluorescence was observed, indicating that all DNA conjugates or their pyrene appendages tightly interacted with the copolymer **IIB** micelles. Figure S122 shows emission spectra of pyrene incubated with different copolymer micelles as control experiments. A spectroscopic titration experiment recording changes in the absorbance intensity of 5’‐aminolinker‐modified dsDNA **DNA‐6** at the absorption maximum of the complex formed (267 nm) determined the mole‐fraction partitioning constant, *K*_x_, to quantitatively describe the partitioning of dsDNA **DNA‐6** between the bulk water phase and the copolymer micelles (Figure 5e, panel C). The measured high *K_x_* of 8.9±1.1 ⋅ 10^5^ indicated that more than 99.99 % (only 1 out of ≈10 000 DNA oligonucleotide conjugates remained in the aqueous phase) of the dsDNA oligomers accumulated in the polymer micelles at copolymer **IIB** concentrations used for reactions on DNA‐tagged starting materials. This observation supported the circular dichroism spectra that were recorded under low salt conditions. Taken together, the fluorescence quenching experiments, UV/Vis absorption measurements and CD spectra indicated strong interaction of DNA conjugates with micelles formed by copolymer **IIB** in water.

## Conclusion

A series of block copolymer micelles has been designed that are composed of a water‐soluble poly(*N*,*N*‐dimethylacrylamide) block and different hydrophobic polyacrylate ester blocks that locate a sulfonic acid catalyst either in the core (type **I**) or in the shell (type **II**). Structural characterization showed that all of these block copolymers formed micelles in water. The shape of these micelles depended on core lipophilicity. Copolymers with short alkyl esters (e. g., ethyl) formed spherical structures, whereas those with longer alkyl esters (e. g., *n*‐octyl) formed worm‐like structures and larger aggregates. Profiling these copolymer micelles over three reactions on DNA‐coupled substrates, namely the Povarov reaction, the Biginelli reaction and Boc removal from an amine revealed trends in micelle structure‐catalytic activity relationships. The micellar catalytic activity was investigated with respect to both target product formation and DNA degradation by cleaving purine bases from the DNA oligomer backbone, commonly called depurination. We found more pronounced DNA depurination in those Biginelli reactions and Boc removal reactions that were promoted by micelles with a lower log *P* in the core, and especially with a large hydrophilic shell, that is, a higher degree of polymerization of the DMA units. Micelles with longer alkyl chains in the core tended to cause less depurination, but were not able to discriminate entirely desired on‐DNA compound synthesis from undesired DNA depurination. Depurination was completely prevented or much reduced by the addition of anilines as reactants (Povarov reaction) or additive (Boc deprotection) to the reaction mixture. Head‐to‐head comparison of all copolymer micelles with one model Povarov reaction (Figure 4a) designed, after a short reaction time, not to give full consumption of the starting material revealed that micelles with a lower log *P* in the core, such as methyl methacrylate‐based **IIA** tended to provide faster reaction rates in this reaction, whereas the *n*‐butyl acrylate copolymer micelle with the larger hydrophilic shell, **IIC_b_**, stood out with considerably slower reaction kinetics. However, profiling these micelles in the Povarov reaction over reactants with different solubility and reactivity revealed that copolymer **IIC_b_** gave the most robust reaction outcome after long reaction times. Copolymer micelle **IIC_b_** also gave higher conversions in the Boc‐deprotection and Biginelli reactions. Unfortunately, in those reactions that lacked a buffering reagent, it produced more DNA depurination than any other micelle. Taken together, the copolymers investigated in this study showed that polymer structure has an impact on catalytic activity and that the micelles that we investigated provide a window of DNA‐compatible reaction conditions. More forcing reaction conditions demanded the addition of buffering components as additives. Future copolymer designs or exploration of further additives might increase this window, and enable further on‐DNA reactions. Our observations suggest that the micellar microenvironment, that is, the interactions of the sulfonic acid catalyst with the substrates and the solubility of the substrates within the micelle, can be fine‐tuned by the composition of the amphiphilic block copolymers. Such optimized host‐guest interactions in polymer micelles have also recently been described to improve drug loading in a specific micellar microenvironment.^[37]^ Zeta‐potential measurements and characterization of the micellar core micropolarity by pyrene measurements indicated that the micellar core is very hydrophobic independent of the sulfonic acid location, thus suggesting that the sulfonic acid groups are more likely located at the core‐shell boundary (for the core‐functionalized micelles, series **I**) and in the hydrophilic shell for the shell‐functionalized micelles (series **II**). However, the most salient finding of this study was the unexpected observation that DNA conjugates accumulated in copolymer micelles under low‐salt conditions, although copolymers **I** and **II** did not contain any motifs belonging to the canon of DNA‐binding structures. This last finding has the potential to be exploited in the construction of nano‐ to micrometer‐sized objects, and could find use in DNA‐based nanotechnologies beyond our intended application in encoded chemistry.

## Supporting information

As a service to our authors and readers, this journal provides supporting information supplied by the authors. Such materials are peer reviewed and may be re‐organized for online delivery, but are not copy‐edited or typeset. Technical support issues arising from supporting information (other than missing files) should be addressed to the authors.

Supplementary
