# Supplementary material for: Towards DNA‐Encoded Micellar Chemistry: DNA‐Micelle Association and Environment Sensitivity of Catalysis
Source: Chemistry. 2021 Jun 7;27(39):10048–57. doi: 10.1002/chem.202100980 (PMC8361662; doi:10.1002/chem.202100980)
Supplement: Supplementary file 1 — Supplementary [file CHEM-27-10048-s001.pdf]

Chemistry–A European Journal

Supporting Information

Towards DNA-Encoded Micellar Chemistry: DNA-Micelle Association and Environment Sensitivity of Catalysis

Mateja Klika Škopić[+], Christian Gramse[+], Rosario Oliva[+], Sabrina Pospich, Laura Neukirch, Magiliny Manisegaran, Stefan Raunser, Roland Winter, Ralf Weberskirch,* and Andreas Brunschweiger*

Content		page
1.	Materials and instruments	S2
2.	Synthesis and characterization of copolymers **I/II/III**	S4
3.	DNA oligonucleotides used for reaction kinetics studies and characterization of DNA-copolymer micelle interaction	S33
4.	Micellar Brønsted acid-mediated synthesis of DNA-hexahydro-1*H*-pyrrolo[3,2-*c*]quinoline conjugates **DNA-11, DNA-18-21** and DNA-hexahydro-1*H*-pyrano[3,2-*c*]quinoline conjugates **DNA-12**	S34
5.	Micellar Brønsted acid-mediated cleavage of the Boc protective group from DNA-(Boc)glycine conjugate **DNA-13**	S111
6.	Extending the scope of the micelle-mediated reactions: Biginelli reaction	S125
7.	Impact of DNA chemical modification on copolymer micelle **I**/**II**-mediated synthesis of DNA-hexahydro-1*H*-pyrrolo[3,2-*c*]quinoline conjugates **DNA-18-21**	S161
8.	Characterization of partitioning of DNA conjugates to copolymer micelles **IIB**	S168
9.	Literature	S172

1. Materials and instruments

Unless otherwise noted, chemicals were purchased from Sigma-Aldrich (Taufkirchen, Germany), Alfa Aesar (Kandel, Germany), Thermo Fisher Scientific (Geel, Belgium), abcr (Karlsruhe, Germany), TCI Chemicals (Eschborn, Germany), and VWR (Langenfeld, Germany). Non-functionalized oligonucleotides in solution and all desalted oligonucleotides in solution were purchased from IDT (Leuven, Belgium). Oligonucleotides in solution: 5'-Alexa Fluor 430-, 5'-pyrene-, and 3'-pyrene-modified DNA oligonucleotides were synthesized by IBA (Göttingen, Germany). 5'-Aminolinker-modified DNA oligonucleotides attached to controlled pore glass solid phase (CPG, 1000 Å) were synthesized by IBA (Göttingen, Germany). Controlled pore glass solid phase was filtered on a synthesis column plugged onto a vacuum manifold (Vac-Man®, Promega). Oligonucleotide-small molecule conjugates were purified by ion pair reverse-phase high-pressure liquid chromatography (HPLC, Shimadzu Prominence) using a C_{18} stationary phase (Phenomenex, Gemini; 5 µm, C18, 110 Å, 100*10.0 mm) and a gradient of 100 mM aqueous triethylammonium acetate/MeOH. The triethylammonium acetate buffer was set to pH= 8. Oligonucleotide-small molecule conjugates were analyzed by ion pair reverse phase high-pressure liquid chromatography (HPLC, Shimadzu Prominence) using a C_{18} stationary phase (Phenomenex, Gemini; 5 µm, C18, 110 Å, 100*4.6 mm) and a gradient of 10 mM aqueous triethylammonium acetate/MeOH. HPLC traces were recorded at 254 nm wavelength. Oligonucleotide concentrations were determined by UV spectroscopy using a spectrophotometer (NanoDrop 2000, Thermo Fisher Scientific). Oligonucleotides were analyzed by MALDI-MS (Bruker Daltonics) using THAP matrix (Dichrom) or HPA matrix. ^{1}H NMR-spectra were measured at 400 MHz, 500 MHz or 600 MHz on a Bruker AVANCE III HD NanoBay, AVANCE HDX-III or AVANCE HD-III spectrometer, respectively. The pure substance was dissolved in deuterated chloroform ($CDCl_3$, 99.8%, VWR, Langenfeld, Germany) or deuterated methanol (CD_3OD, 99.8%, VWR, Langenfeld, Germany). Chemical shifts are listed relative to the deuterated solvent. Each proton signal was analyzed regarding its multiplicity, coupling constant *J* [Hz], and the amount of protons. The multiplicity was abbreviated as follows: s = singulet, d = doublet, t = triplet, q = quartet, quint = quintet, m = multiplet, and br = broad signal. Size exclusion chromatography (SEC) was performed on an instrument from Merck Hitachi (L-5000 LC Controller, 655A-11 Liquid Chromatograph) equipped with an RI detector from Knauer (RI Detector Smartline 2300). The columns were purchased from PSS Copolymer Standards Service GmbH, Mainz, Germany (PSS GRAM pre-column, PSS GRAM analytical 1000 Å, PSS GRAM analytical 30 Å) and operated with a flow rate of 1 mL/min at 35 °C with DMF (HPLC-Grade +0.025 M LiBr) as solvent. The system was calibrated with a PMMA calibration kit from PSS. Copolymers were dissolved in DMF (HPLC-Grade +0.025 M LiBr, 3 mg/mL) and filtered with a 0.2 µm PTFE syringe filter. Dynamic light scattering (DLS) and zeta potential measurements were carried out with an instrument

from Brookhaven Instruments (Holtsville, USA) (ZetaPALS, 35 mW solid state laser, λ = 660 nm) at 25 °C with 1 mM aqueous copolymer solutions. The DLS results are an average of five, the zeta potential results are an average of ten measurements. Fluorescence measurements were carried out on the device F-2700 Fluorescence Spectrophotometer from Hitachi (Chiyoda, Japan). Reactions sensitive to air and/or moisture were carried out in heated Schlenk flasks under argon atmosphere. Liquid monomers were distilled before use. AIBN was recrystallized from methanol. All copolymers used in this work were synthesized using the RAFT[1] technique. SAXS measurements were carried out at ambient pressure at 25 °C on an Anton Paar SAXSess mc^2 (Graz, Austria) using a monochromatic X-ray beam (λ = 1.54 Å) and an image plate detector. Fluorescence quenching experiments were analysed with a K2 fluorimeter from ISS (Champaign, Illinois, USA) equipped with a xenon arc lamp as light source. UV/Vis spectroscopy experiments were performed with a UV-1800 spectrophotometer (Shimadzu Corporation, Kyoto, Japan). Circular dichroism spectroscopy spectra were acquired on a Jasco J-715 (Jasco Corporation, Tokio, Japan).

2. Synthesis and characterization of copolymers **I/II/III**

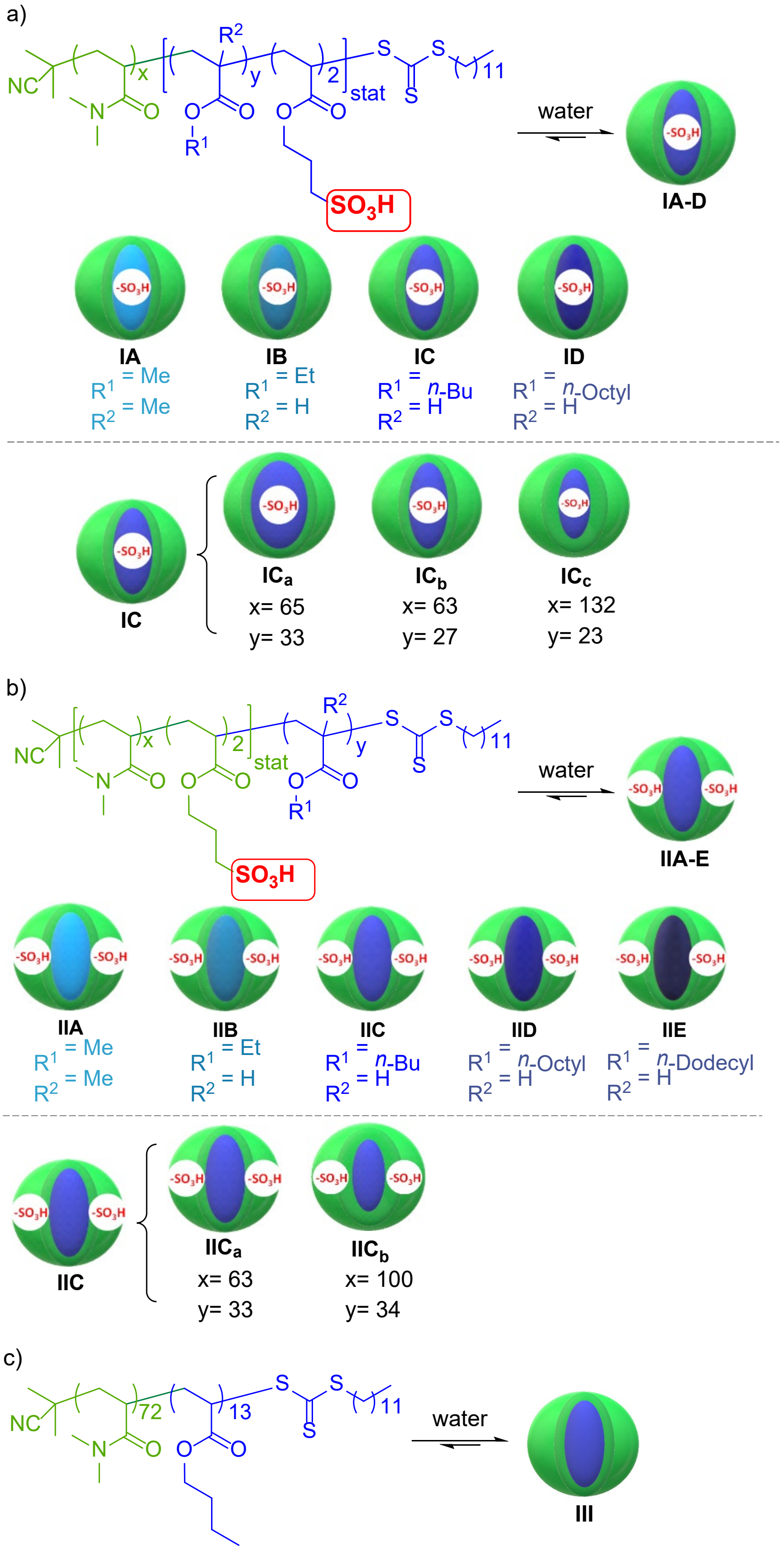

Figure S1. Copolymer structures and the schematic depiction of the formed micelles. Copolymers differed with respect to positioning of the catalyst, lipophilicity of the micellar core, and degree of polymerization in the core

and in the corona. a) General structure of the copolymer **I** series consisting of a *N*,*N*-dimethylacrylamide portion and a lipophilic acrylate ester portion co-polymerized with a sulfonic acid moiety and schematic depiction of the formed micelles. b) General structure of the copolymer **II** series consisting of a *N*,*N*-dimethylacrylamide portion co-polymerized with a sulfonic acid moiety, and a lipophilic acrylate ester portion. Both copolymer series **I/II** contained copolymers of different core lipophilicity indicated with different shades of blue: **I/IIA** (methyl metacrylate), **I/IIB** (ethyl acrylate), **I/IIC** (*n*-butyl acrylate), **I/IID** (*n*-octyl acrylate), **IIE** (*n*-dodecyl acrylate). Each copolymer series contained copolymers of different degree of polymerization in the core: **IC**$_a$ versus **IC**$_b$ (**IC**$_a$ displayed larger hydrophobic core than **IC**$_b$), and in the corona: **IC**$_b$ versus **IC**$_c$ (**IC**$_c$ displayed larger hydrophilic corona than **IC**$_b$), **IIC**$_a$ versus **IIC**$_b$ (**IIC**$_b$ displayed larger hydrophilic corona than **IIC**$_a$). c) Structure of the unfunctionalized copolymer **III**.

2.1 Synthesis of the hydrophilic polymer block

The synthesis of the hydrophilic polymer block without the sulfonic acid monomer was carried out according to the protocol of Laschewsky *et al.*[2] Here we describe the synthesis of pre-polymer **Pre1**.

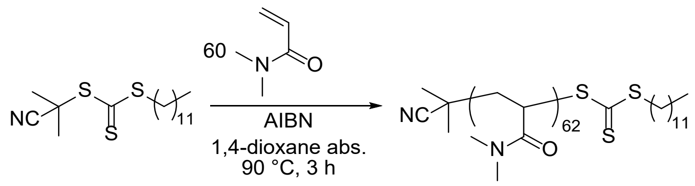

2CPT **Pre1**

Scheme S1. RAFT polymerization of the hydrophilic polymer **Pre1**.

In a pressure stable Schlenk flask *N,N*-dimethylacrylamide (DMA) (4.7 g, 47.4 mmol, 60.0 eq.), 2-cyano-2-propyl dodecyl trithiocarbonate (2CPT) (273.1 mg, 0.8 mmol, 1.0 eq.) and AIBN (25.9 mg, 0.2 mmol, 0.2 eq.) were dissolved in anhydrous 1,4-dioxane. The mixture was degassed via three freeze-pump-thaw cycles and polymerized for 3 h at 90 °C. Afterwards, the polymer mixture was precipitated into cooled diethyl ether. After centrifugation and decantation the precipitate was dried in high vacuum. The polymer was obtained as yellow solid in 92% yield (4.6 g)

The polymerization of **Pre5** was carried out in toluene.

The degree of polymerization (*dp*) was determined by ^{1}H NMR spectroscopy. The methyl group of the dodecyl chain of the 2-cyano-2-propyl dodecyl trithiocarbonate (0.88 ppm) was used as reference and compared to the methyl protons of the *N,N*-dimethylacrylamide (3.13-2.93 ppm).

Table S1. Analytical data of the pre-polymers **Pre1 – Pre6** and **Pre11**.

Polymer	DMA[a] (theor.)	M_n theor. [g/mol]	M_n NMR [g/mol][a]	M_n SEC ($Đ$) [g/mol][b]	yield [%]
**Pre1**	62 (60)	6293	6492	6990 (1.16)	92
**Pre2**	62 (60)	6293	6492	6400 (1.15)	88
**Pre3**	65 (60)	6293	6789	7640 (1.19)	85
**Pre4**	63 (60)	6293	6591	6398 (1.24)	71
**Pre5**	132 (132)	13430	13430	11720 (1.23)	45
**Pre6**	59 (60)	6293	6194	7010 (1.16)	85
**Pre11**	72(60)	6293	7483	8240 (1.15)	76

[a] determined by ^{1}H NMR spectroscopy; [b] DMF + 0.025 M LiBr, 35 °C, PMMA-standard.

Pre1: ^{1}H NMR (400 MHz, $CDCl_3$): δ = 3.13-2.93 (br, 372 H), 0.88 ppm (t, J = 6 Hz, 3 H).

Pre2: ^{1}H NMR (400 MHz, $CDCl_3$): δ = 3.13-2.89 (br, 373 H), 0.87 ppm (t, J = 6 Hz, 3 H).

Pre3: ^{1}H NMR (600 MHz, $CDCl_3$): δ = 3.13-2.90 (br, 391 H), 0.88 ppm (t, J = 6 Hz, 3 H).

Pre4: ^{1}H NMR (500 MHz, $CDCl_3$): δ = 3.11-2.88 (br, 377 H), 0.86 ppm (t, J = 7.5 Hz, 3 H).

Pre5: ^{1}H NMR (500 MHz, $CDCl_3$): δ = 3.11-2.89 (br, 794 H), 0.87 ppm (t, J = 7.5 Hz, 3 H).

Pre6: ^{1}H NMR (500 MHz, $CDCl_3$): δ = 3.12-2.90 (br, 353 H), 0.88 ppm (t, J = 6 Hz, 3 H).

Pre11: ^{1}H NMR (500 MHz, $CDCl_3$): δ = 3.13-2.93 (br, 433 H), 0.88 ppm (t, J = 6 Hz, 3 H).

Synthesis of the polymer **Pre10** (hydrophilic polymer block with the sulfonic acid monomer):

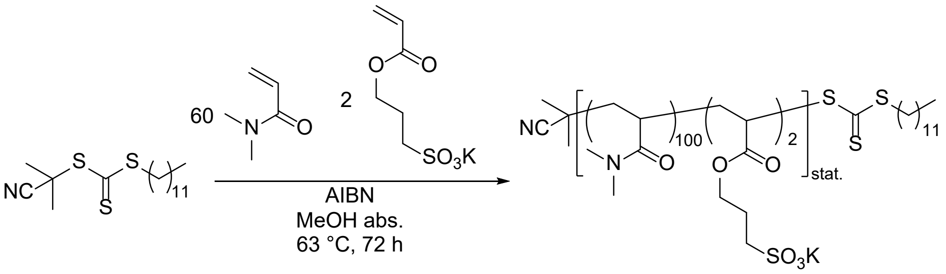

2CPT **Pre10**

Scheme S2. RAFT polymerization of the hydrophilic polymer **Pre10**.

In a pressure-stable Schlenk flask *N,N*-dimethylacrylamide (DMA) (0.5 g, 5.0 mmol, 60.0 eq.), 3-sulfopropyl acrylate potassium salt (39.5 mg, 0.2 mmol, 2.00 eq), 2-cyano-2-propyl dodecyl trithiocarbonate (2CPT) (29.0 mg, 0.1 mmol, 1.0 eq.) and AIBN (2.8 mg, 0.02 mmol, 0.2 eq.) were dissolved in anhydrous methanol. The mixture was degassed via three freeze-pump-thaw cycles and polymerized for 72 h at 63 °C. Afterwards, the solvent was removed and the residue was dissolved in dichloromethane before the polymer mixture was precipitated into cooled diethyl ether. After centrifugation and decantation the precipitate was dried in high vacuum. The polymer was obtained as yellow solid in 68% yield (0.5 g).

The degree of polymerization (*dp*) was determined via ^{1}H NMR spectroscopy. The methyl group of the dodecyl chain of the 2-cyano-2-propyl dodecyl trithiocarbonate (0.90 ppm) was used as reference and compared to the methyl protons of the *N,N*-dimethylacrylamide (3.17-2.92 ppm) and to the characteristic methylene signal of the 3-sulfopropyl acrylate potassium salt (4.20 ppm) .

Table S2. Analytical data of the pre-polymer **Pre10**.

Polymer	DMA[a] (theor.)	SPA[a] (theor.)	M_n theor. [g/mol]	M_n NMR [g/mol][a]	M_n SEC ($Đ$) [g/mol][b]	yield [%]
**Pre10**	100 (60)	2 (2)	6682	10723	11020 (1.09)	58

[a] determined by ^{1}H NMR spectroscopy; [b] DMF + 0.025 M LiBr, 35 °C, PMMA-standard.

Pre10: ^{1}H NMR (500 MHz, CD_3OD): δ = 4.20 (br, 4 H), 3.17-2.92 (br, 598 H), 0.91 ppm (t, J = 7.5 Hz, 3 H).

The syntheses of the pre-polymers **Pre7**, **Pre8** and **Pre9** (hydrophilic polymer block with the sulfonic acid monomer) were carried out under a general protocol of Laschewsky *et al.*[2] and are shown for polymer **Pre8** as an example.

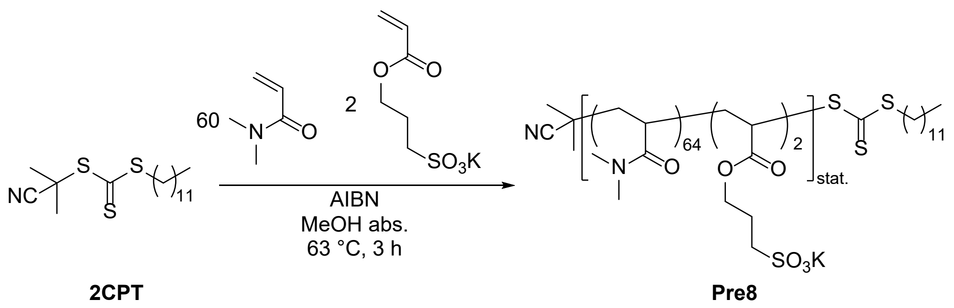

Scheme S3. RAFT polymerization of the hydrophilic polymer **Pre8**.

In a pressure-stable Schlenk flask *N,N*-dimethylacrylamide (DMA) (2.0 g, 20.2 mmol, 60.0 eq.), 3-sulfopropyl acrylate potassium salt (156.2 mg, 0.7 mmol, 2.0 eq), 2-cyano-2-propyl dodecyl

trithiocarbonate (2CPT) (116.1 mg, 0.3 mmol, 1.0 eq.) and AIBN (11.0 mg, 0.1 mmol, 0.2 eq.) were dissolved in anhydrous methanol. The mixture was degassed via three freeze-pump-thaw cycles and polymerized for 3 h at 63 °C. Afterwards, the solvent was removed and the residue was dissolved in dichloromethane before the polymer mixture was precipitated into cooled diethyl ether. After centrifugation and decantation the precipitate was dried in high vacuum. The polymer was obtained as yellow solid in 85% yield (2.05 g).

The degree of polymerization (*dp*) was determined as described above.

Table S3. Analytical data of the pre-polymers **Pre7 – Pre9.**

Polymer	DMA[a] (theor.)	SPA[a] (theor.)	M_n theor. [g/mol]	M_n NMR [g/mol][a]	M_n SEC ($Đ$) [g/mol][b]	yield [%]
**Pre7**	62 (60)	2 (2)	6682	6956	9210 (1.14)	89
**Pre8**	60 (60)	2 (2)	6682	6682	8830 (1.13)	85
**Pre9**	63 (60)	2 (2)	6682	7055	9400 (1.09)	87

[a] determined by ^{1}H NMR spectroscopy; [b] DMF + 0.025 M LiBr, 35 °C, PMMA-standard.

Pre7: ^{1}H NMR (400 MHz, CD_3OD): δ = 4.20 (br, 4 H), 3.17-2.92 (br, 372 H), 0.90 ppm (t, J = 6 Hz, 3 H).

Pre8: ^{1}H NMR (400 MHz, CD_3OD): δ = 4.20 (br, 4 H), 3.17-2.92 (br, 360 H), 0.91 ppm (t, J = 6 Hz, 3 H).

Pre9: ^{1}H NMR (400 MHz, $CDCL_3$): δ = 4.26 (br, 4 H), 3.11-2.89 (br, 381 H), 0.87 ppm (t, J = 8 Hz, 3 H).

2.2 Synthesis of the hydrophobic polymer block

The synthesis of the hydrophobic polymer block with the sulfonic acid monomer is shown for polymer **IC$_c$** as an example.

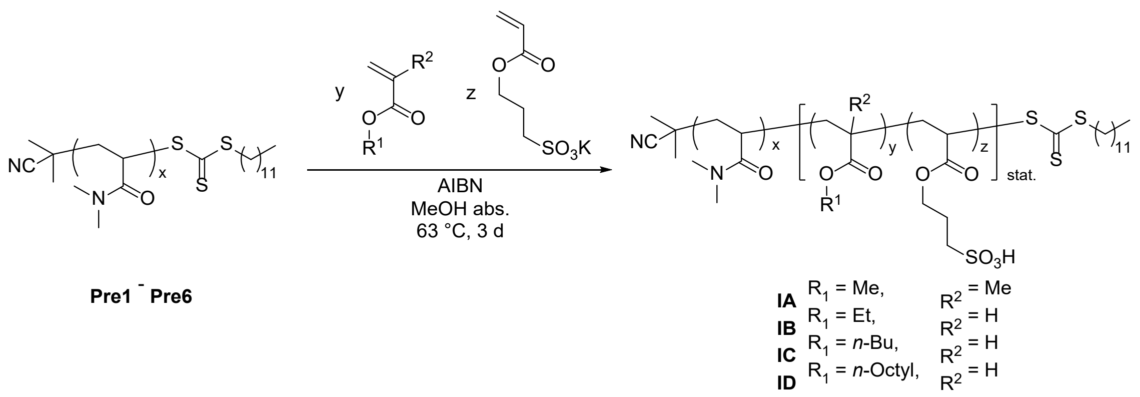

Scheme S4. Synthesis of the amphiphilic block copolymers with the sulfonic acid inside the hydrophobic block.

In a pressure-stable Schlenk flask pre-polymer **Pre5** (Macro-RAFT) (0.5 g, 0.04 mmol, 1.0 eq.), *n*-butyl acrylate (BA) (143.2 mg, 1.1 mmol, 30.0 eq.), 3-sulfopropyl acrylate potassium salt (SPA-K) (17.2 mg, 0.1 mmol, 2.0 eq.) and AIBN (1.2 mg, 0.01 mmol, 0.2 eq.) were dissolved in anhydrous MeOH (6 mL). The mixture was degassed via three freeze-pump-thaw cycles and polymerized for 3 d at 63 °C. The solvent was removed and the residue was dissolved in dichloromethane and precipitated into cooled diethyl ether. After centrifugation and decantation the precipitate was dried in high vacuum. After this the polymer was dissolved in a small amount of MeOH and treated with Amberlyst® 15 to obtain the desired sulfonic acid. The solvent was removed and the residue was dissolved in H_2O and dried by freeze-drying. The polymer was obtained as yellow solid in 74% yield (0.5 g).

The *dp* was determined via ^{1}H NMR-Spectroscopy. Due to a possible hydrolysis of the RAFT end group the methyl protons of the DMA (3.18-2.92 ppm) were used as a reference and compared to characteristic signals of the BA (4.08 ppm, 0.98 ppm) and 3-sulfopropyl acrylate potassium salt (SPA) (4.19 ppm).

The polymerization of the polymers with the other acrylate monomers (methyl methacrylate, ethyl acrylate, *n*-octyl acrylate) followed the same procedure. Precipitation of the *n*-octyl acrylate did not work. To determine the *dp* of these polymers the characteristic signals of the acrylates were used as well.

Table S4. Analytical data of the copolymers with the sulfonic acid inside the hydrophobic block.

Polymer	DMA[a]	Acrylate[a] (theor.)	SPA[a] (theor.)	M_n theor. [g/mol]	M_n NMR [g/mol][a]	M_n SEC ($Đ$) [g/mol][b]	d_h [nm][c]	yield [%]
**IA**	62	MMA 25 (25)	2 (2)	9384	9384	8960 (1.56)	1.86 ± 1.08	69
**IB**	62	EA 23 (25)	2 (2)	9384	9183	10150 (1.28)	1.20 ± 0.98	69
**IC$_a$**	65	BA 33 (30)	2 (2)	11023	11407	11240 (1.28)	16.08 ± 2.80	56
**IC$_b$**	65	BA 27 (30)	2 (2)	11023	10382	11190 (1.31)	16.26 ± 2.80	18
**IC$_c$**	132	BA 23 (30)	2 (2)	11664	16767	12360 (1.69)	7.72 ± 1.33	74
**ID**	59	OA 26 (25)	2 (2)	11190	11374	11730 (1.24)	40.43 ± 2.83	86

MMA = methyl methacrylate, EA = ethyl acrylate, BA = *n*-butyl acrylate, OA = *n*-octyl acrylate;
[a] determined via ^{1}H NMR spectroscopy; [b] DMF + 0.025 M LiBr, 35 °C, PMMA-standard; [c] 1mM aqueous polymer solution.

IA: ^{1}H NMR (600 MHz, CD_3OD): δ = 4.16 (br, 4 H), 3.67 (br, 76 H), 3.18-2.89 ppm (br, 372 H).

IB: ^{1}H NMR (400 MHz, CD_3OD): δ = 4.23 (br, 4 H), 4.13 (br, 46 H), 3.18-2.92 (br, 372 H), 1.27 (br, 70 H), 0.91 ppm (t, *J* = 6 Hz, 2 H).

IC$_a$: ^{1}H NMR (600 MHz, CD_3OD): δ = 4.19 (br, 4 H), 4.08 (br, 67 H), 3.18-2.92 (br, 390 H), 0.98 (t, *J* = 6 Hz, 99 H), 0.90 ppm (t, *J* = 6 Hz, 3 H).

IC$_b$: ^{1}H NMR (400 MHz, CD_3OD): δ = 4.19 (br, 4 H), 4.08 (br, 54 H), 3.18-2.92 (br, 378 H), 0.98 (t, *J* = 8 Hz, 75 H), 0.91 ppm (t, *J* = 6 Hz, 2 H).

IC$_c$: ^{1}H NMR (400 MHz, CD_3OD): δ = 4.19 (br, 4 H), 4.08 (br, 46 H), 3.18-2.92 (br, 792 H), 0.98 (t, *J* = 6 Hz, 70 H), 0.91 ppm (t, *J* = 6 Hz, 2 H).

ID: ^{1}H NMR (600 MHz, CD_3OD): δ = 4.19 (br, 4 H), 4.07 (br, 50 H), 3.18-2.91 (br, 372 H), 1.27 (br, 70 H), 0.93 ppm (br, 81 H).

The synthesis of the hydrophobic polymer block without the sulfonic acid monomer is shown for polymer **IIC$_a$** as an example.

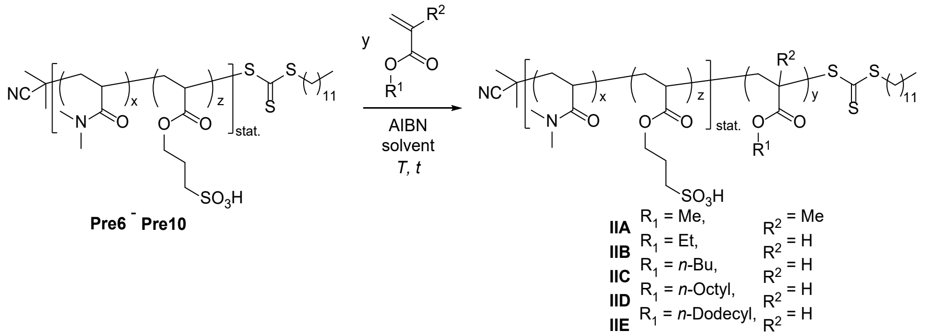

Scheme S5. Synthesis of the amphiphilic block copolymers with the sulfonic acid inside the hydrophilic block.

In a pressure stable Schlenk flask pre-polymer **Pre9** (Macro-RAFT) (1.0 g, 0.2 mmol, 1.0 eq.), *n*-butyl acrylate (BA) (495.5 mg, 3.9 mmol, 25.0 eq.) and AIBN (5.1 mg, 0.03 mmol, 0.2 eq.) were dissolved in anhydrous MeOH (2 mL). The mixture was degassed via three freeze-pump-thaw cycles and polymerized for 18 h at 63 °C. The solvent was removed and the residue was dissolved in dichloromethane and precipitated into cooled diethyl ether. After centrifugation and decantation the precipitate was dried in high vacuum. After this the polymer was dissolved in a small amount of MeOH and treated with Amberlyst® 15 to obtain the desired sulfonic acid. The solvent was removed and the residue was dissolved in H_2O and dried by freeze-drying. The polymer was obtained as yellow solid in 76% yield (1.3 g). The *dp* was determined as described above.

The polymerization of the ethyl acrylate followed the same procedure. The polymerization of the methyl methacrylate and *n*-octyl acrylate was carried out at 90 °C in 1,4-dioxane for 18 h and the polymerization of *n*-dodecyl acrylate was carried out at 90 °C in 1,4-dioxane for 72 h. This polymer was also dialyzed against EtOH prior to the Amberlyst® 15 treatment.

The synthesis of the unfunctionalized block copolymer **III** was carried out as follows.

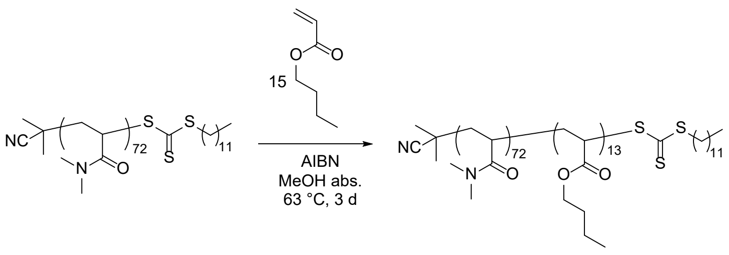

Scheme S6. Synthesis of the unfunctionalized amphiphilic block copolymer.

In a pressure-stable Schlenk flask **Pre11** (400.0 mg, 0.05 mmol, 1.0 eq.), *n*-butyl acrylate (BA) (165.5 mg, 1.3 mmol, 20.0 eq) and AIBN (2,1 mg, 0.01 mmol, 0.2 eq.) were dissolved in anhydrous methanol. The mixture was degassed via three freeze-pump-thaw cycles and polymerized for 3 d at 63 °C. Afterwards, the solvent was removed and the residue was dissolved in dichloromethane before the polymer mixture was precipitated into cooled diethyl ether. After centrifugation and decantation the precipitate was dried in high vacuum. The polymer was obtained as yellow solid in 75% yield (323.9 mg).

Table S5. Analytical data of the copolymers with the sulfonic acid inside the hydrophilic block and copolymer **III**.

Polymer	DMA[a]	SPA[a]	Acrylate[a] (theor.)	M_n theor. [g/mol]	M_n NMR [g/mol][a]	M_n SEC ($Đ$) [g/mol][b]	d_h [nm][c]	yield [%]
**IIA**	62	2	MMA 14 (25)	9383	8284	9460 (1.32)	280.17 ± 47.44	82
**IIB**	60	2	EA 23 (30)	9582	9258	11620 (1.15)	0.40 ± 0.32	67
**IIC$_a$**	63	2	BA 33 (25)	10183	11209	13280 (1.16)	26.03 ± 4.76	76
**IIC$_b$**	100	2	BA 34 (30)	14492	15005	12150 (1.34)	21.30 ± 2.17	67
**IID**	62	2	OA 26 (25)	11487	11671	13350 (1.13)	46.33 ± 3.48[d]	69
**IIE**	62	2	DDA 12 (25)	12890	9765	10890 (1.21)	24.89 ± 2.71[d]	67
**III**	72	–	BA 13 (15)	9406	8149	11305 (1.13)	21.75 ± 2.69	75

MMA = methyl methacrylate, EA = ethyl acrylate, BA = *n*-butyl acrylate, OA = *n*-octyl acrylate, DDA = *n*-dodecyl acrylate;
[a] determined via ^{1}H NMR spectroscopy; [b] DMF + 0.025 M LiBr, 35 °C, PMMA-standard; [c] 1mM aqueous polymer solution; [d] sample was filtered before measurement.

IIA: ^{1}H NMR (400 MHz, $CDCl_3$): δ = 4.28 (br, 4 H), 3.60 (br, 42 H), 3.14-2.93 ppm (br, 372 H).

IIB: ^{1}H NMR (600 MHz, CD_3OD): δ = 4.19 (br, 4 H), 4.13 (br, 46 H), 3.16-2.92 (br, 360 H), 1.27 (br, 70 H), 0.91 ppm (t, *J* = 6 Hz, 3 H).

IIC$_a$: ^{1}H NMR (400 MHz, CD_3OD): δ = 4.21 (br, 5 H), 4.08 (br, 66 H), 3.17-2.92 (br, 378 H), 0.98 (t, *J* = 8 Hz, 98 H), 0.91 ppm (t, *J* = 8 Hz, 3 H).

IIC$_b$: ^{1}H NMR (400 MHz, CD_3OD): δ = 4.20 (br, 5 H), 4.08 (br, 68 H), 3.18-2.92 (br, 600 H), 0.98 (t, *J* = 8 Hz, 101 H), 0.91 ppm (t, *J* = 6 Hz, 3 H).

IID: ^{1}H NMR (400 MHz, CD_3OD): δ = 4.21 (br, 4 H), 4.07 (br, 50 H), 3.16-2.92 (br, 372 H), 0.93 ppm (br, 82 H).

IIE: ^{1}H NMR (600 MHz, CD_3OD): δ = 4.22 (br, 4 H), 4.07 (br, 25 H), 3.17-2.92 (br, 372 H), 0.95-0.91 ppm (br, 37 H).

III: ^{1}H NMR (400 MHz, $CDCl_3$): δ = 4.04 (br, 25 H), 3.15-2.94 (br, 432 H), 0.94 (t, *J* = 6 Hz, 38 H), 0.88 ppm (t, *J* = 6 Hz, 2 H).

a)

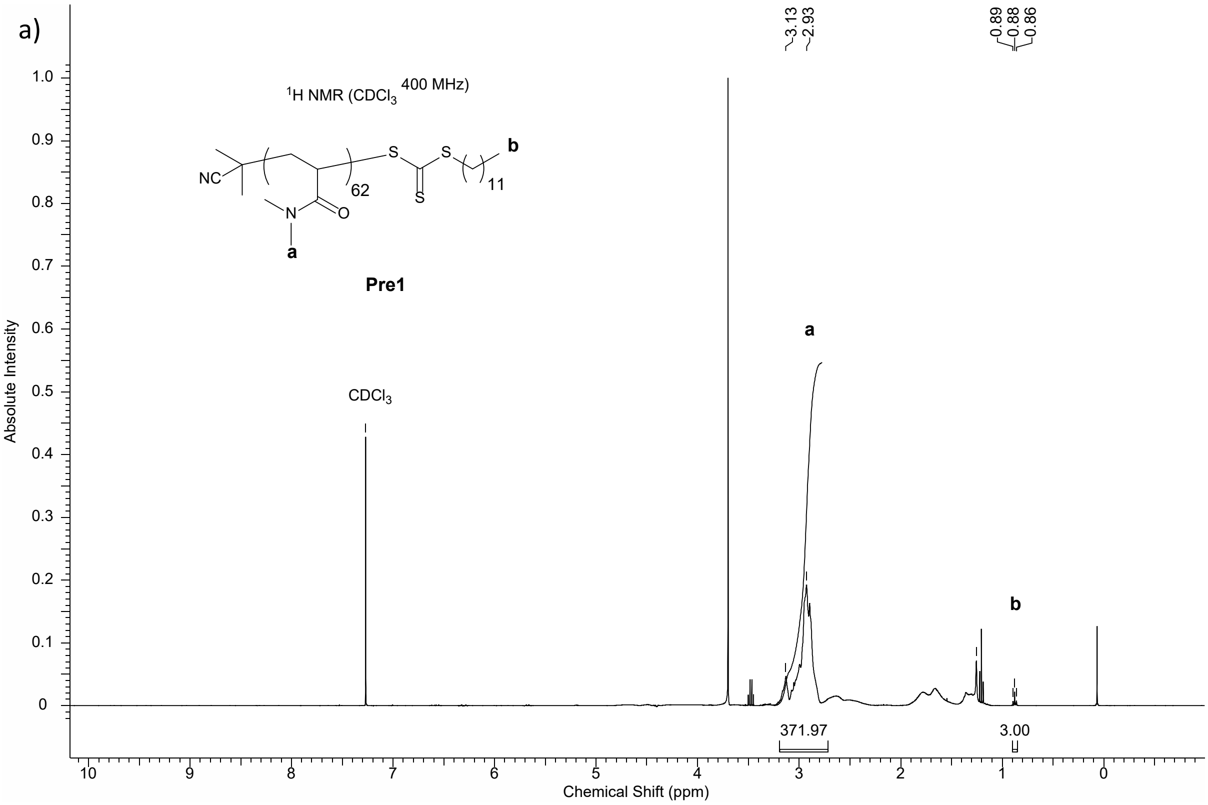

3.13
2.93
0.89
0.88
0.86
^{1}H NMR (CDCl$_3$ 400 MHz)
NC
62
S
S
S
11
b
N
O
a
Pre1
a
CDCl$_3$
b
371.97
3.00
Absolute Intensity
Chemical Shift (ppm)

b)

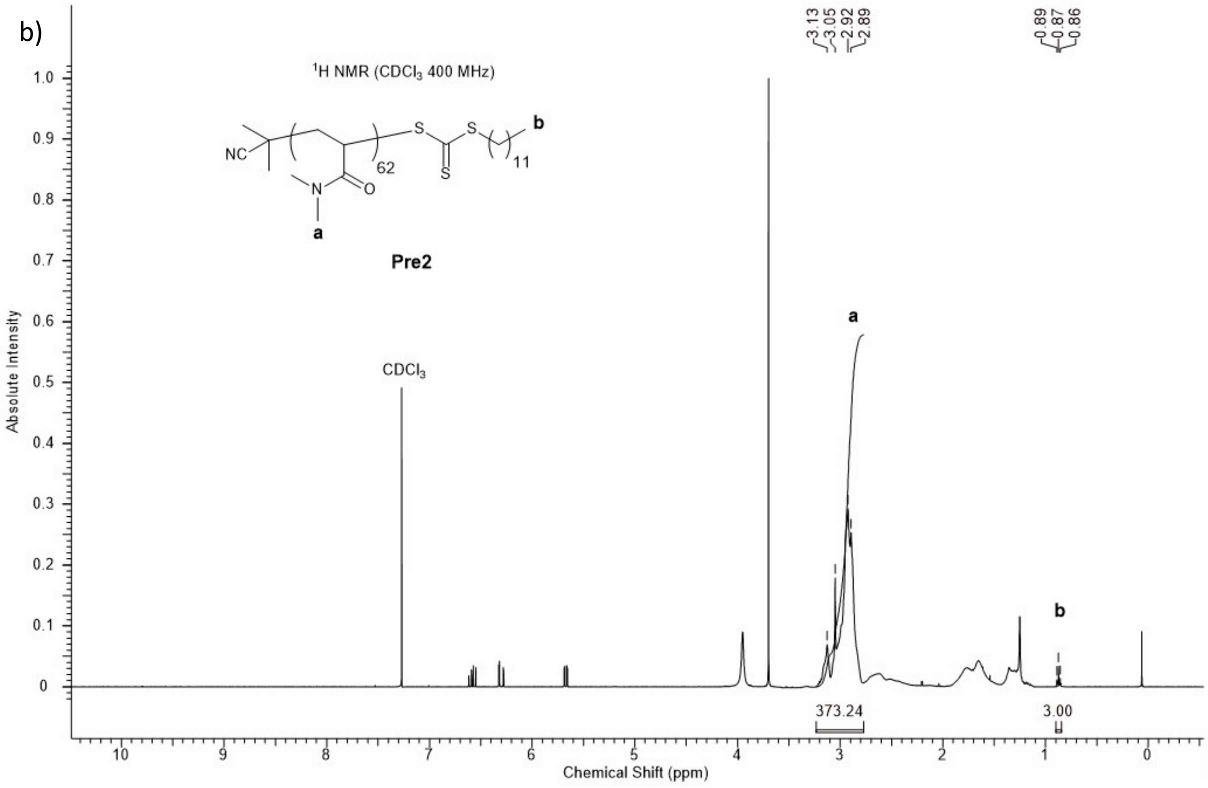

3.13
3.05
2.92
2.89
0.89
0.87
0.86
^{1}H NMR (CDCl$_3$ 400 MHz)
NC
62
S
S
S
11
b
N
O
a
Pre2
a
CDCl$_3$
b
373.24
3.00
Absolute Intensity
Chemical Shift (ppm)

c)

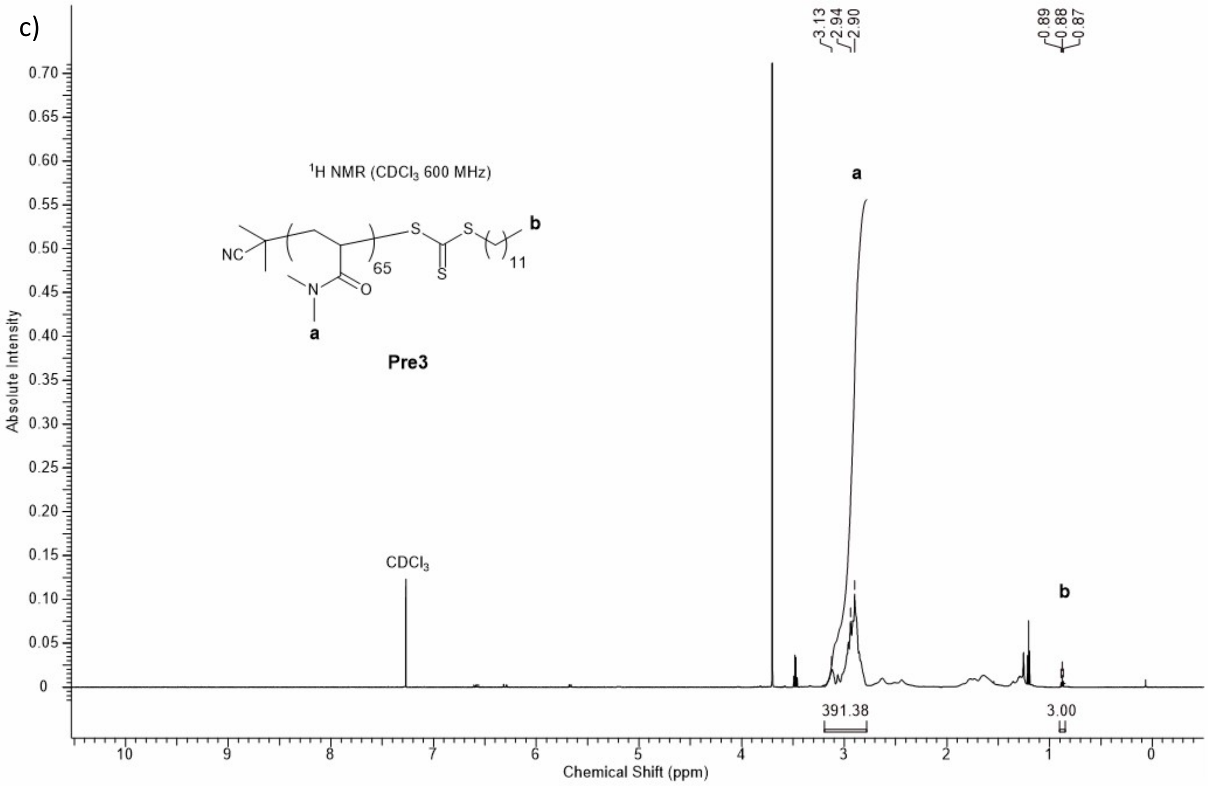
3.13
2.94
2.90
0.89
0.88
0.87
¹H NMR (CDCl₃ 600 MHz)
a
b
NC
65
S
S
S
11
N
O
a
Pre3
CDCl₃
b
391.38
3.00
Absolute Intensity
Chemical Shift (ppm)

d)

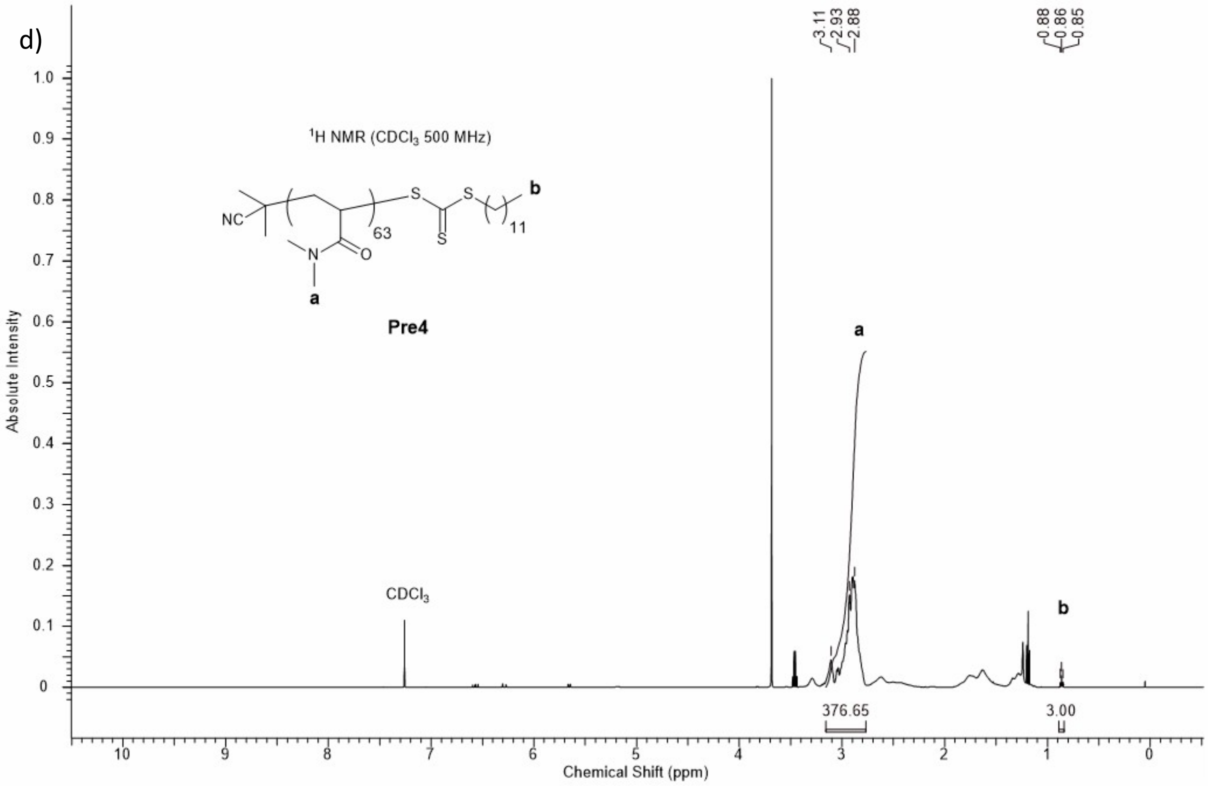
3.11
2.93
2.88
0.88
0.86
0.85
¹H NMR (CDCl₃ 500 MHz)
b
NC
63
S
S
S
11
N
O
a
Pre4
a
CDCl₃
b
376.65
3.00
Absolute Intensity
Chemical Shift (ppm)

e)

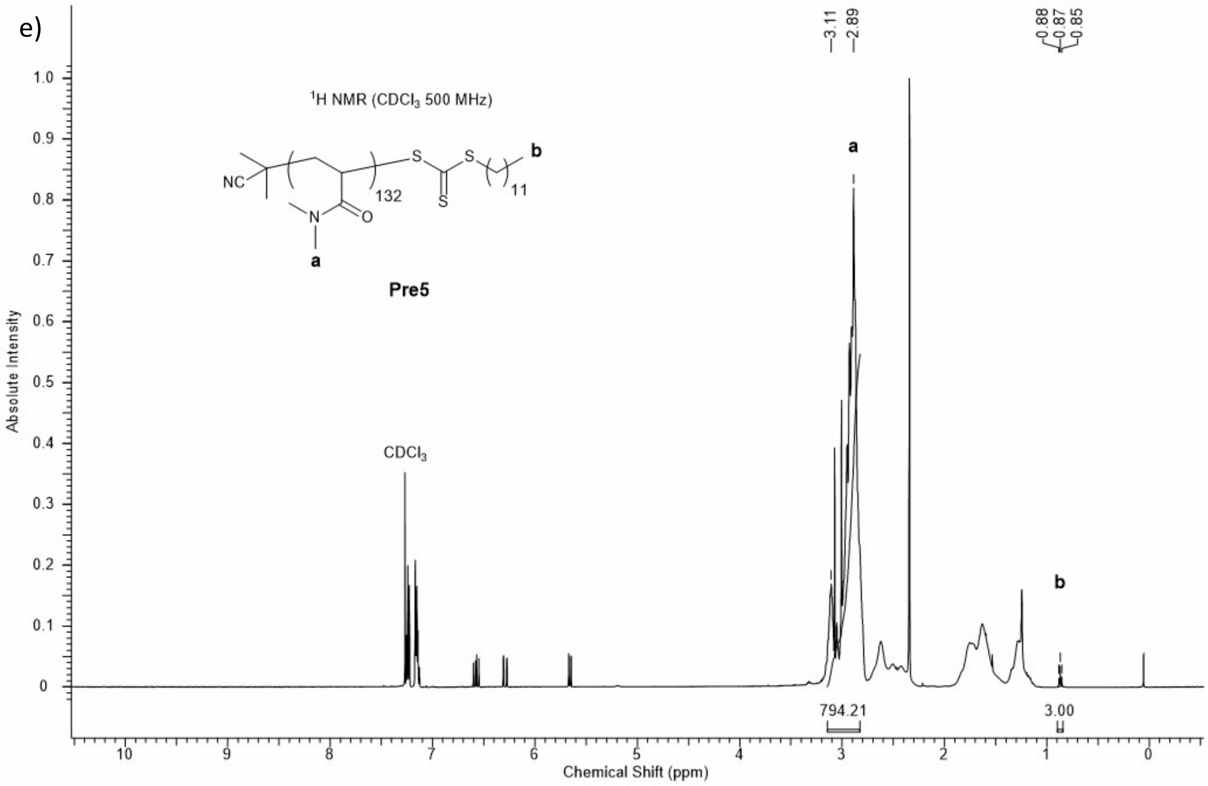

3.11
2.89
0.88
0.87
0.85
¹H NMR (CDCl₃ 500 MHz)
NC
132
S
S
S
11
b
N
O
a
Pre5
a
b
CDCl₃
Absolute Intensity
794.21
3.00
Chemical Shift (ppm)

f)

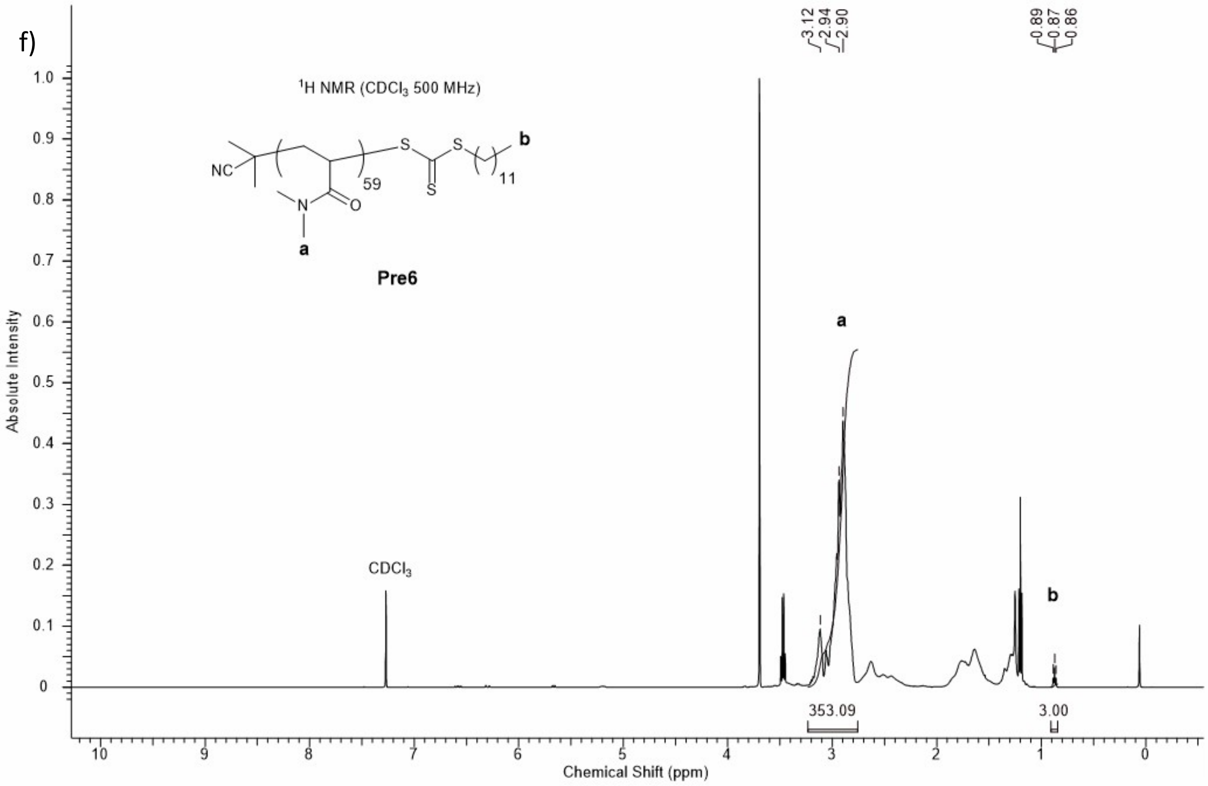

3.12
2.94
2.90
0.89
0.87
0.86
¹H NMR (CDCl₃ 500 MHz)
NC
59
S
S
S
11
b
N
O
a
Pre6
a
b
CDCl₃
Absolute Intensity
353.09
3.00
Chemical Shift (ppm)

g)

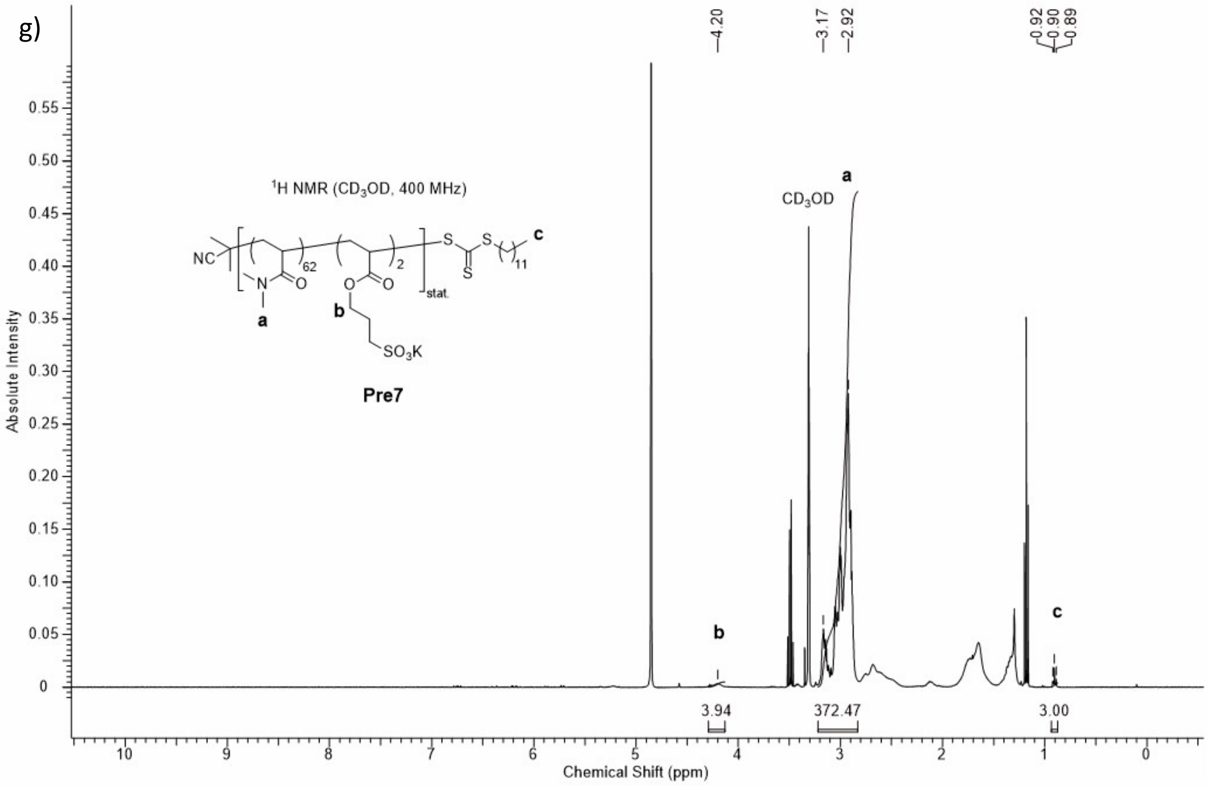

^1H NMR (CD_3OD, 400 MHz)
Pre7
CD_3OD
a
b
c
4.20
3.17
2.92
0.92
0.90
0.89
3.94
372.47
3.00
Absolute Intensity
Chemical Shift (ppm)

h)

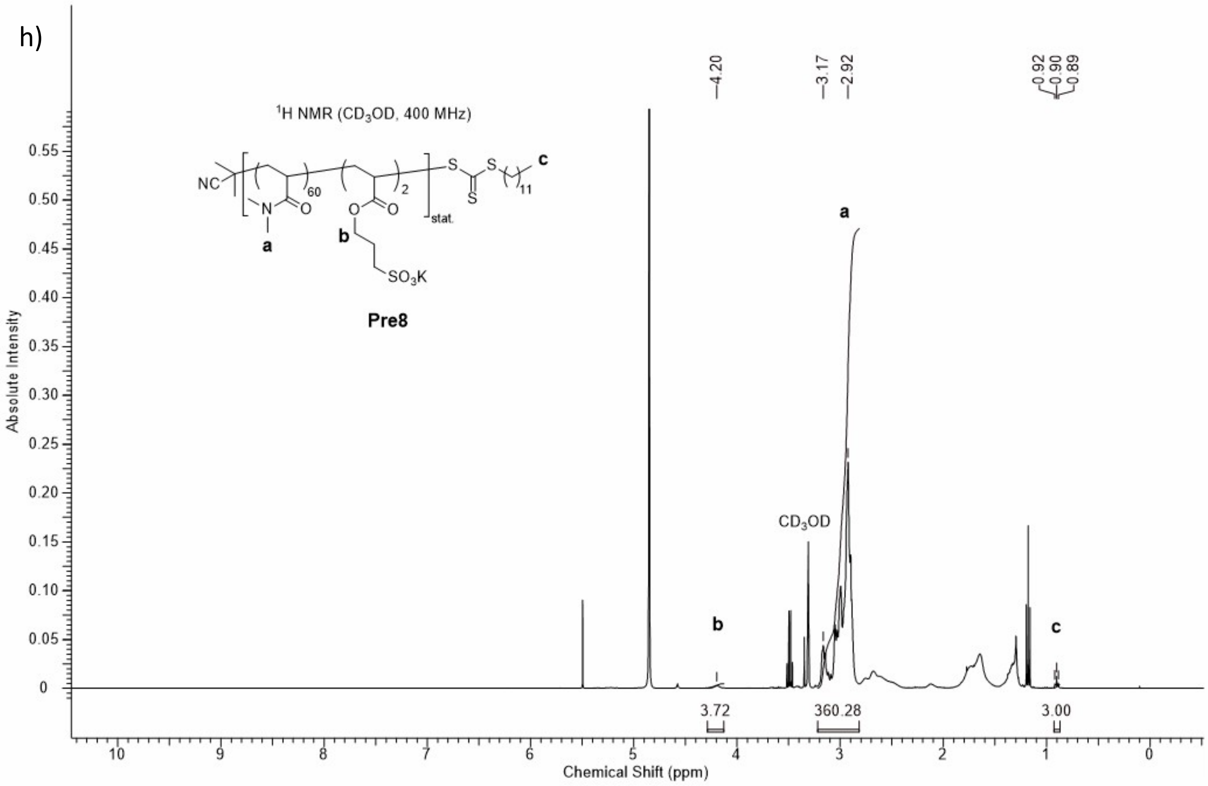

^1H NMR (CD_3OD, 400 MHz)
Pre8
CD_3OD
a
b
c
4.20
3.17
2.92
0.92
0.90
0.89
3.72
360.28
3.00
Absolute Intensity
Chemical Shift (ppm)

i)

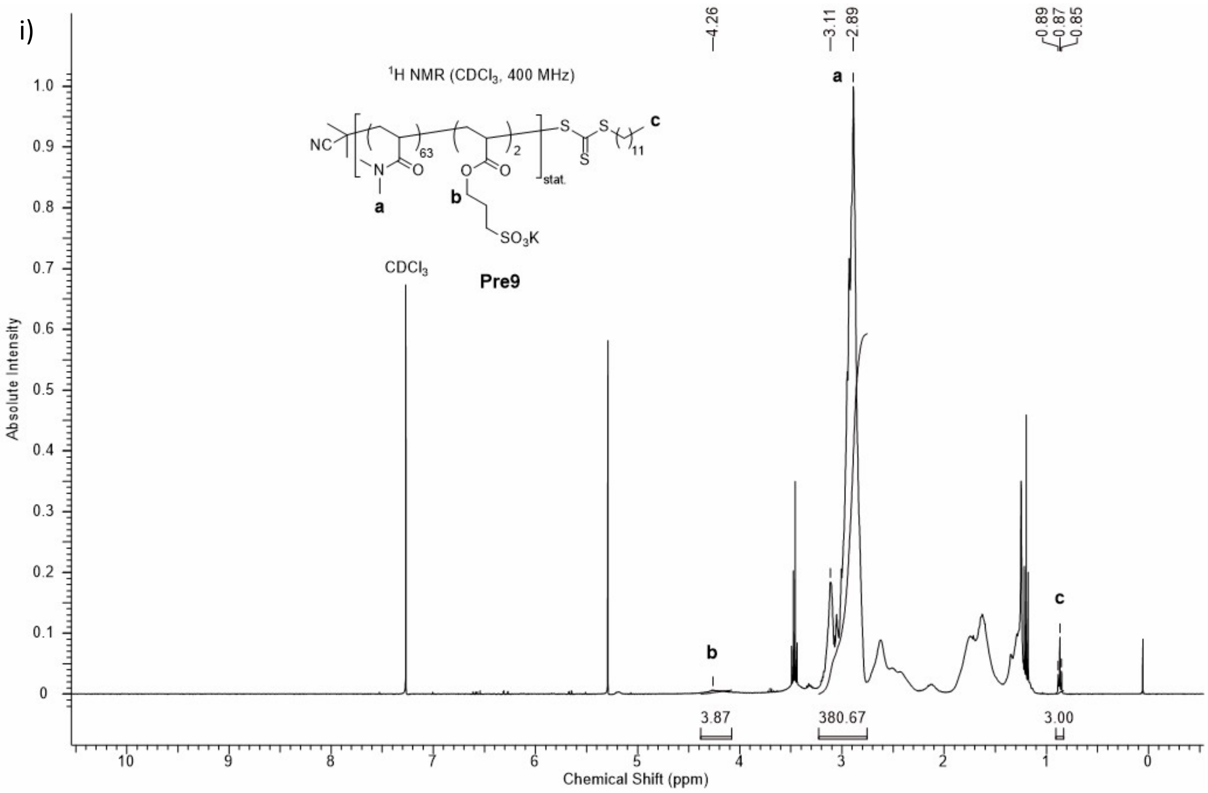

¹H NMR (CDCl₃, 400 MHz)
Pre9
CDCl₃
a
b
c
4.26
3.11
2.89
0.89
0.87
0.85
3.87
380.67
3.00
Absolute Intensity
Chemical Shift (ppm)

j)

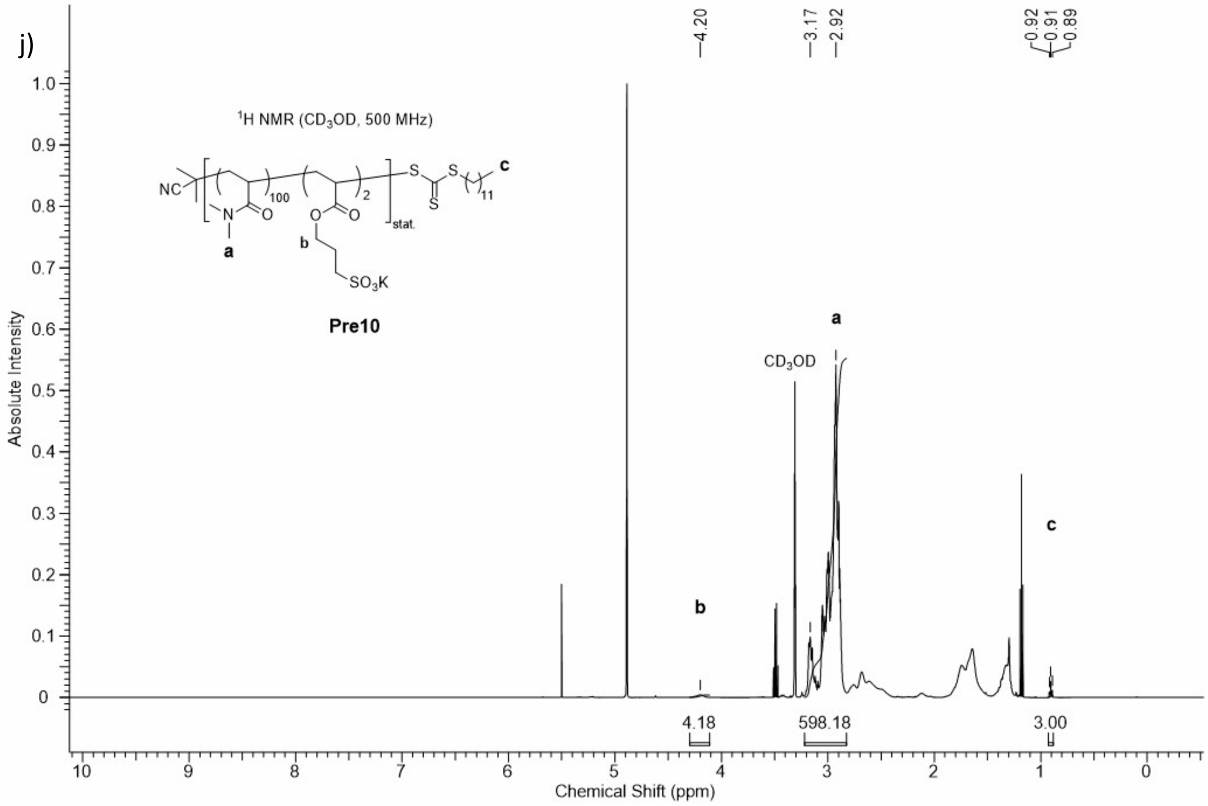

¹H NMR (CD₃OD, 500 MHz)
Pre10
CD₃OD
a
b
c
4.20
3.17
2.92
0.92
0.91
0.89
4.18
598.18
3.00
Absolute Intensity
Chemical Shift (ppm)

k)

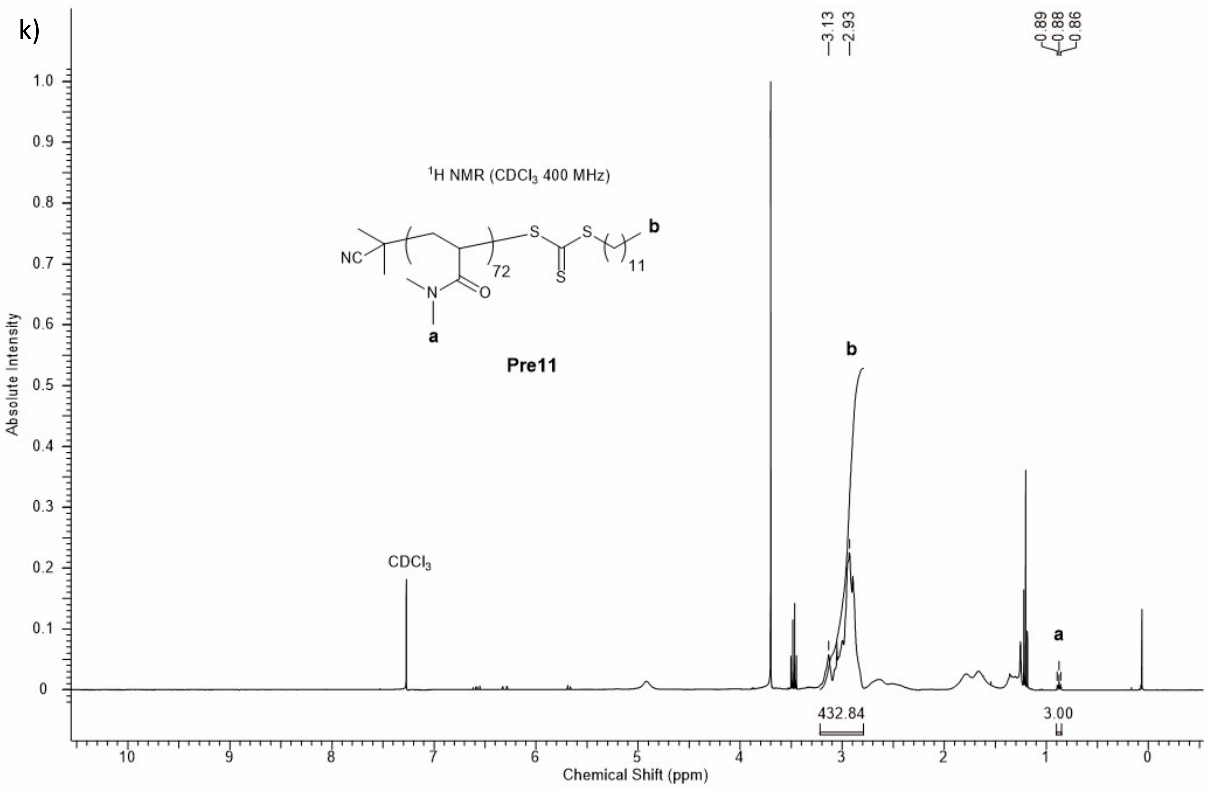

–3.13
–2.93
0.89
0.88
0.86
^{1}H NMR (CDCl$_3$ 400 MHz)
NC
72
S
S
S
11
N
O
a
b
Pre11
CDCl$_3$
432.84
3.00
Absolute Intensity
Chemical Shift (ppm)

l)

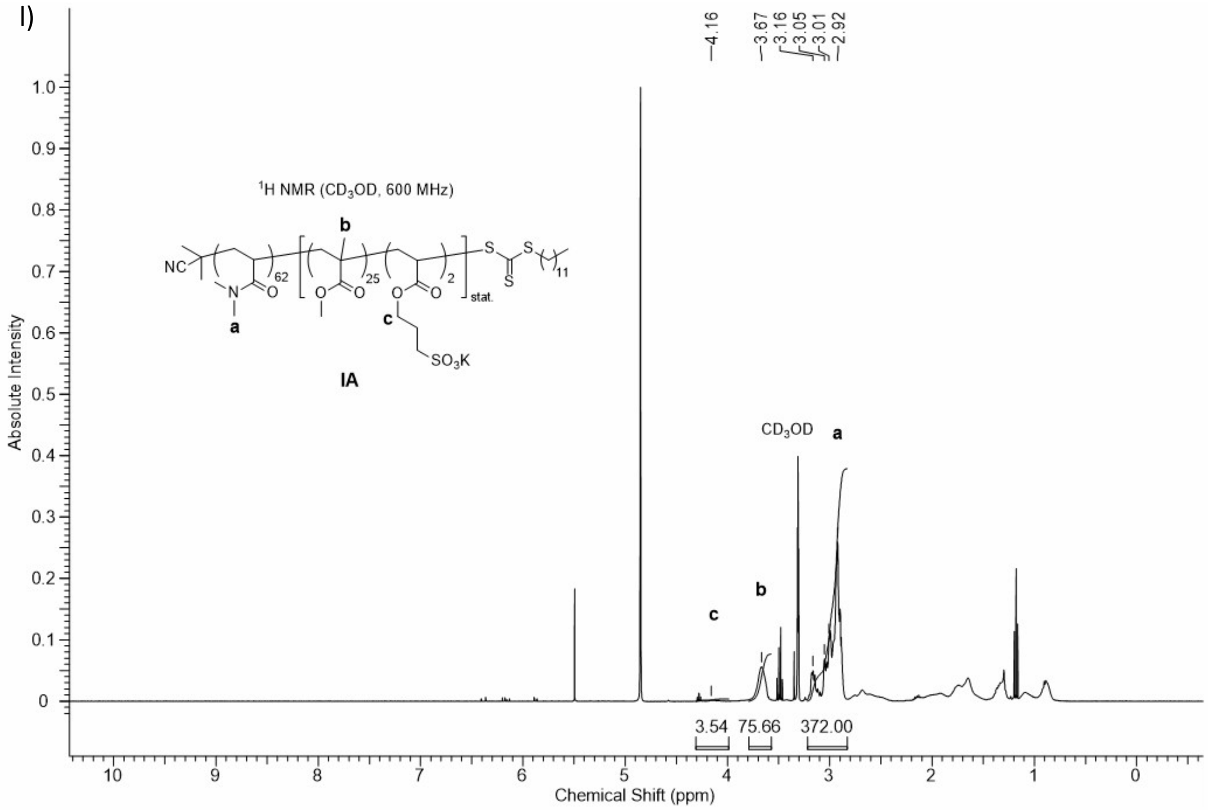

–4.16
–3.67
3.16
3.05
3.01
2.92
^{1}H NMR (CD$_3$OD, 600 MHz)
NC
62
25
2
stat.
SO$_3$K
11
a
b
c
IA
CD$_3$OD
3.54
75.66
372.00
Absolute Intensity
Chemical Shift (ppm)

m)

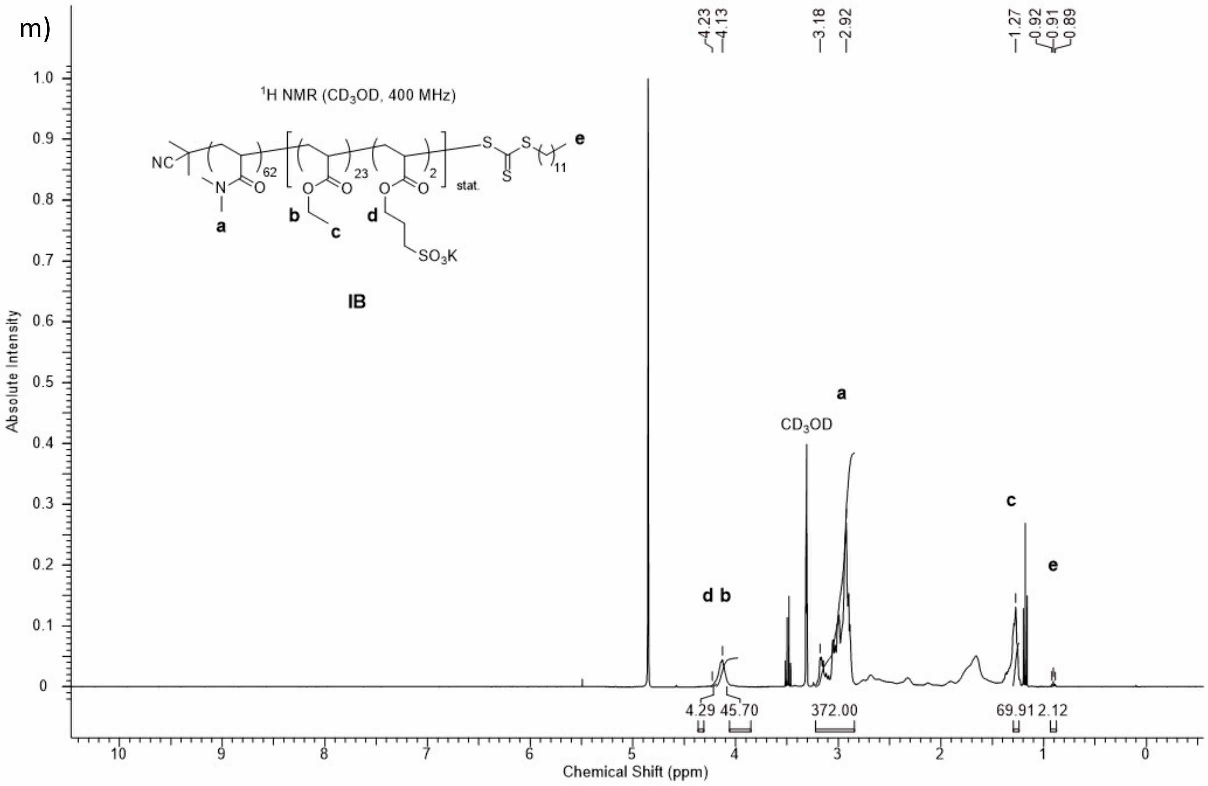

^{1}H NMR (CD$_3$OD, 400 MHz)
IB
Absolute Intensity
Chemical Shift (ppm)
CD$_3$OD
a
b
c
d
e
4.23
4.13
3.18
2.92
1.27
0.92
0.91
0.89
4.29 45.70
372.00
69.91 2.12

n)

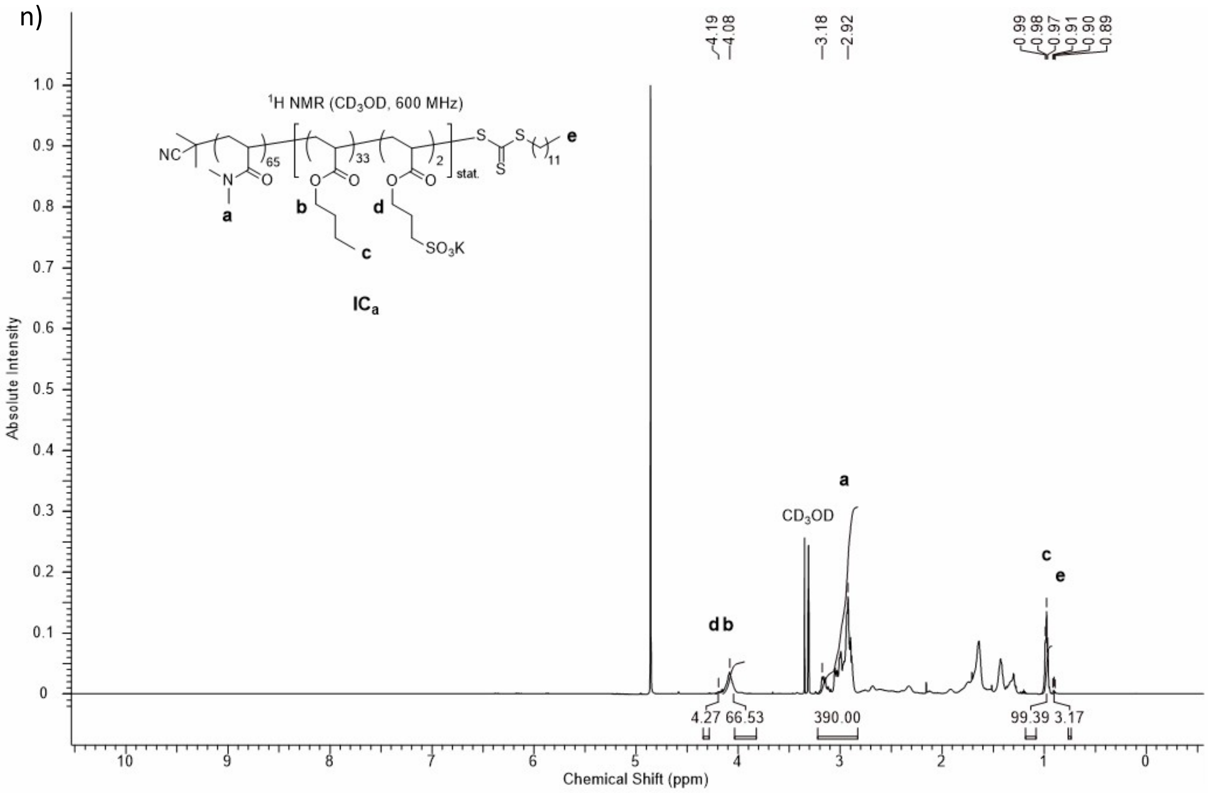

^{1}H NMR (CD$_3$OD, 600 MHz)
IC$_a$
Absolute Intensity
Chemical Shift (ppm)
CD$_3$OD
a
b
c
d
e
4.19
4.08
3.18
2.92
0.99
0.98
0.97
0.91
0.90
0.89
4.27 66.53
390.00
99.39 3.17

o)

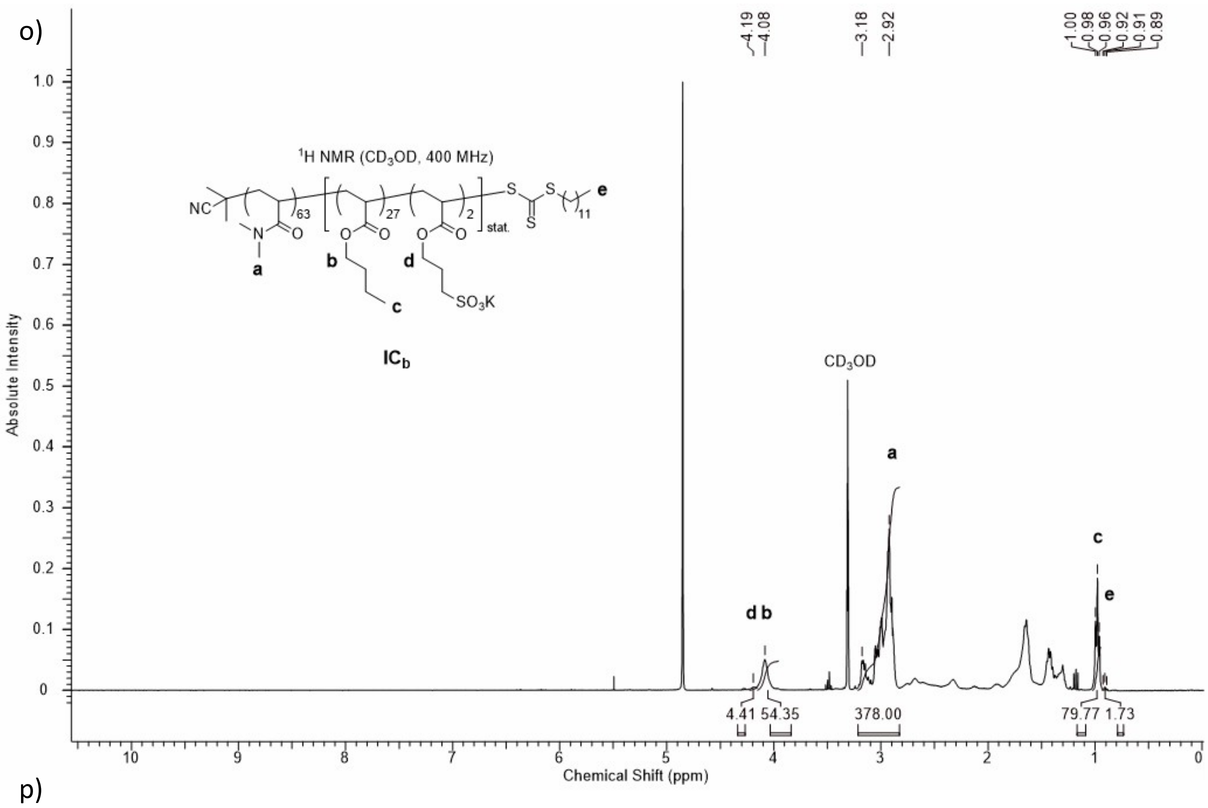

4.19
4.08
3.18
2.92
1.00
0.98
0.96
0.92
0.91
0.89
^{1}H NMR (CD$_3$OD, 400 MHz)
NC
63
27
2
stat.
S
S
S
11
e
N
O
O
O
O
O
a
b
d
c
SO$_3$K
IC$_b$
CD$_3$OD
a
c
e
d b
4.41
54.35
378.00
79.77
1.73
Absolute Intensity
Chemical Shift (ppm)

p)

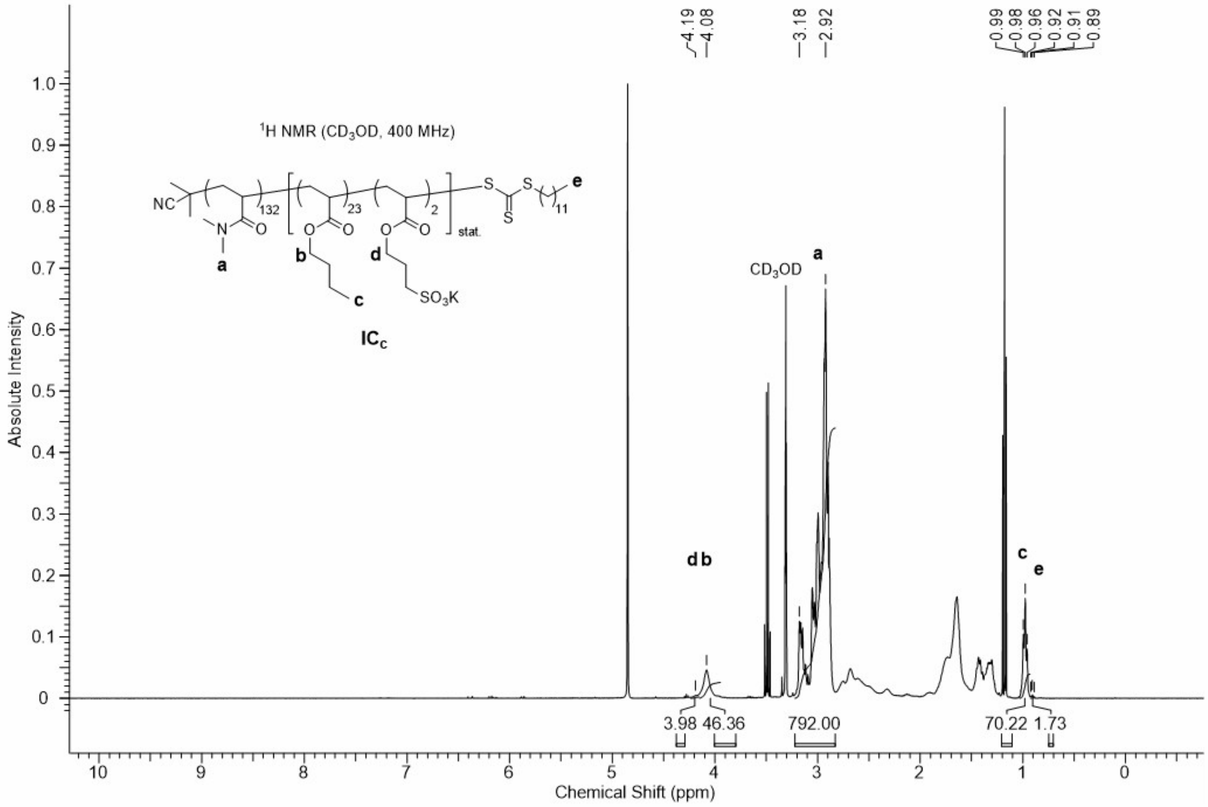

4.19
4.08
3.18
2.92
0.99
0.98
0.96
0.92
0.91
0.89
^{1}H NMR (CD$_3$OD, 400 MHz)
NC
132
23
2
stat.
S
S
S
11
e
N
O
O
O
O
O
a
b
d
c
SO$_3$K
IC$_c$
CD$_3$OD
a
c
e
d b
3.98
46.36
792.00
70.22
1.73
Absolute Intensity
Chemical Shift (ppm)

q)

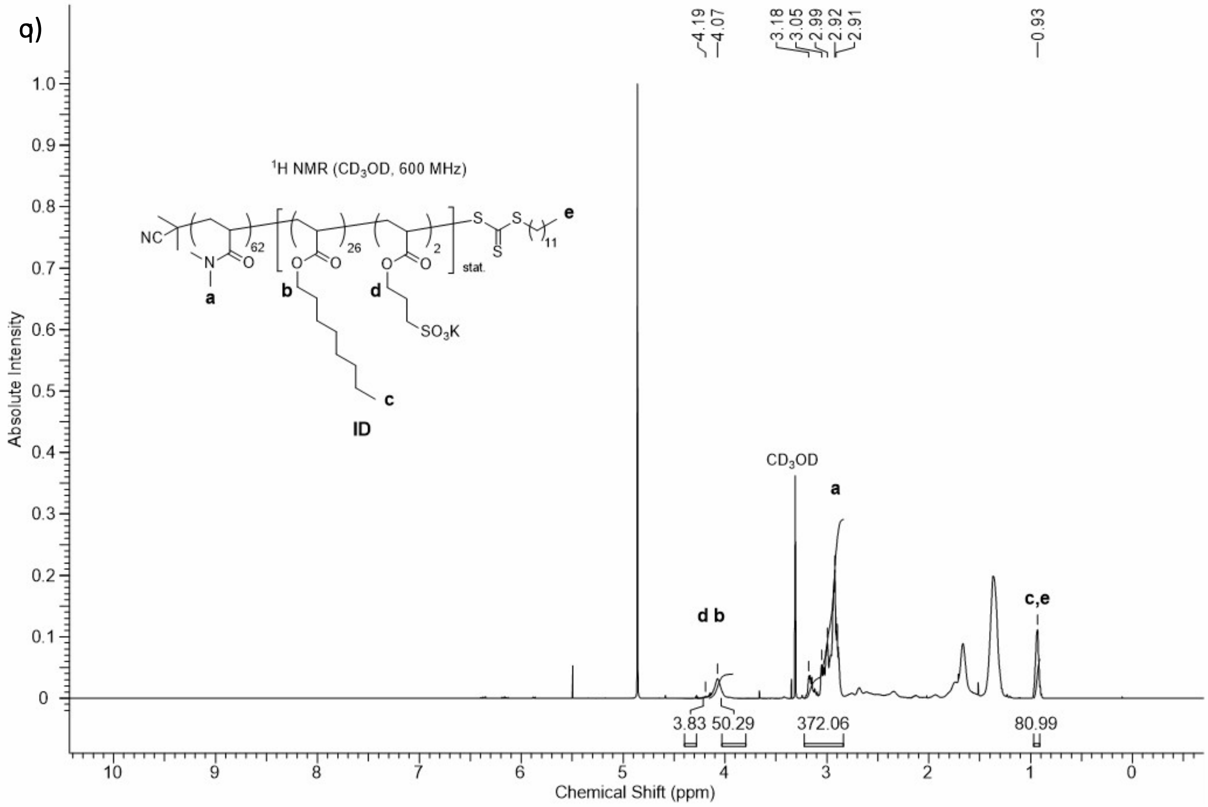

¹H NMR (CD₃OD, 600 MHz)
ID
CD₃OD
a
d b
c,e
Absolute Intensity
Chemical Shift (ppm)
4.19
4.07
3.18
3.05
2.99
2.92
2.91
0.93
3.83
50.29
372.06
80.99

r)

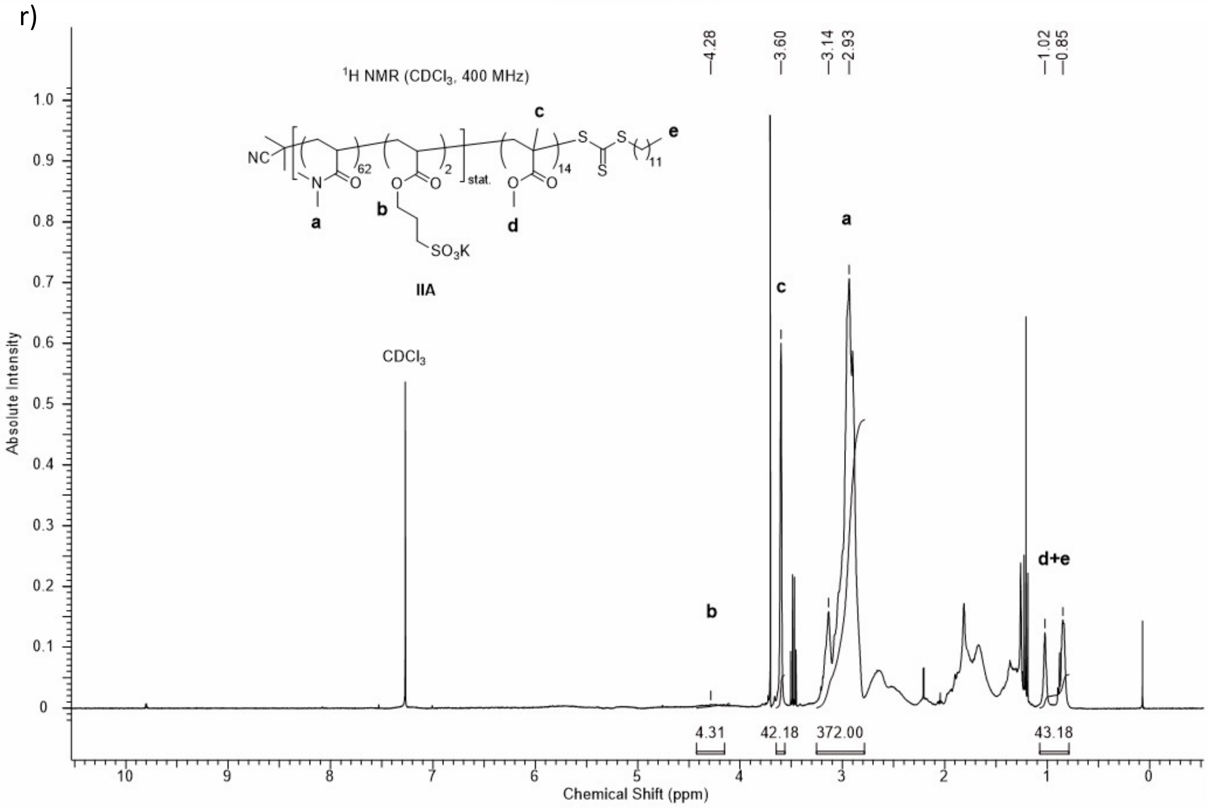

¹H NMR (CDCl₃, 400 MHz)
IIA
CDCl₃
a
b
c
d+e
Absolute Intensity
Chemical Shift (ppm)
4.28
3.60
3.14
2.93
1.02
0.85
4.31
42.18
372.00
43.18

s)

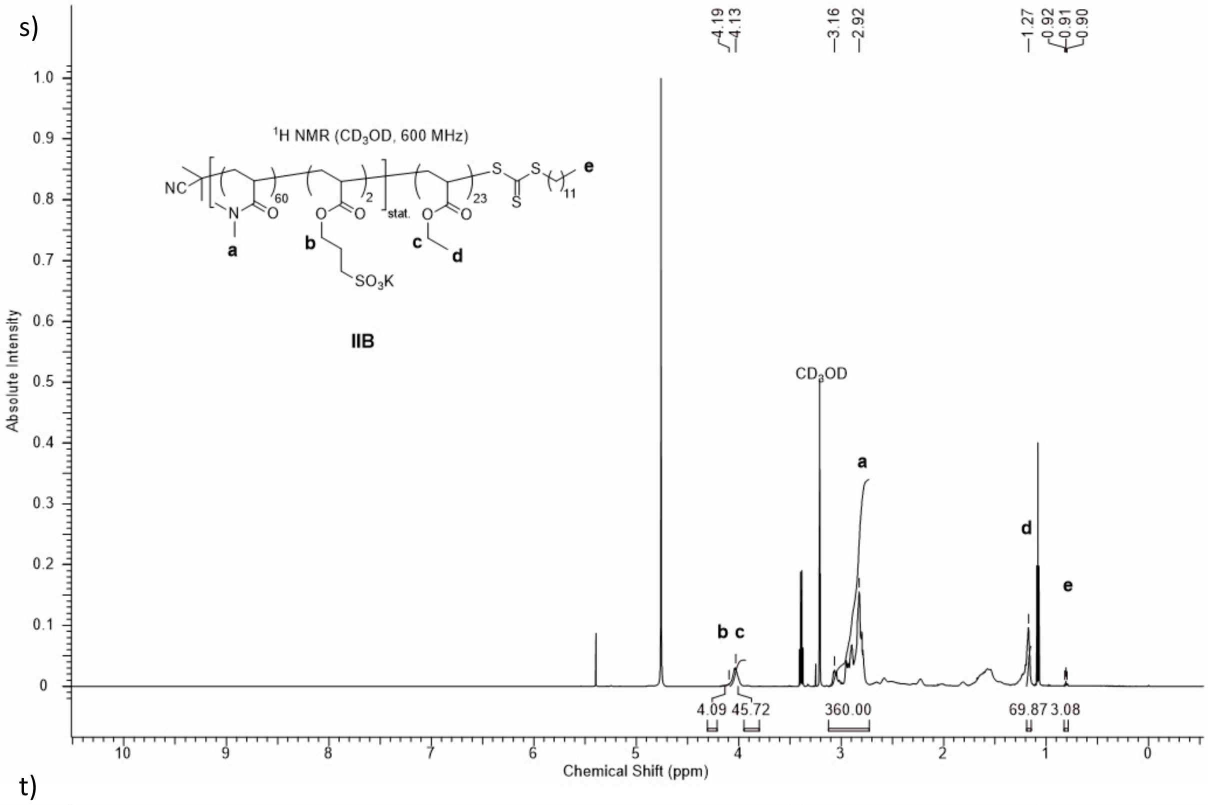

4.19
4.13
3.16
2.92
1.27
0.92
0.91
0.90
1H NMR (CD3OD, 600 MHz)
NC
60
2
stat.
23
S
S
S
11
e
N
O
O
O
O
O
a
b
c
d
SO3K
IIB
CD3OD
a
d
e
b c
4.09
45.72
360.00
69.87
3.08
Absolute Intensity
Chemical Shift (ppm)

t)

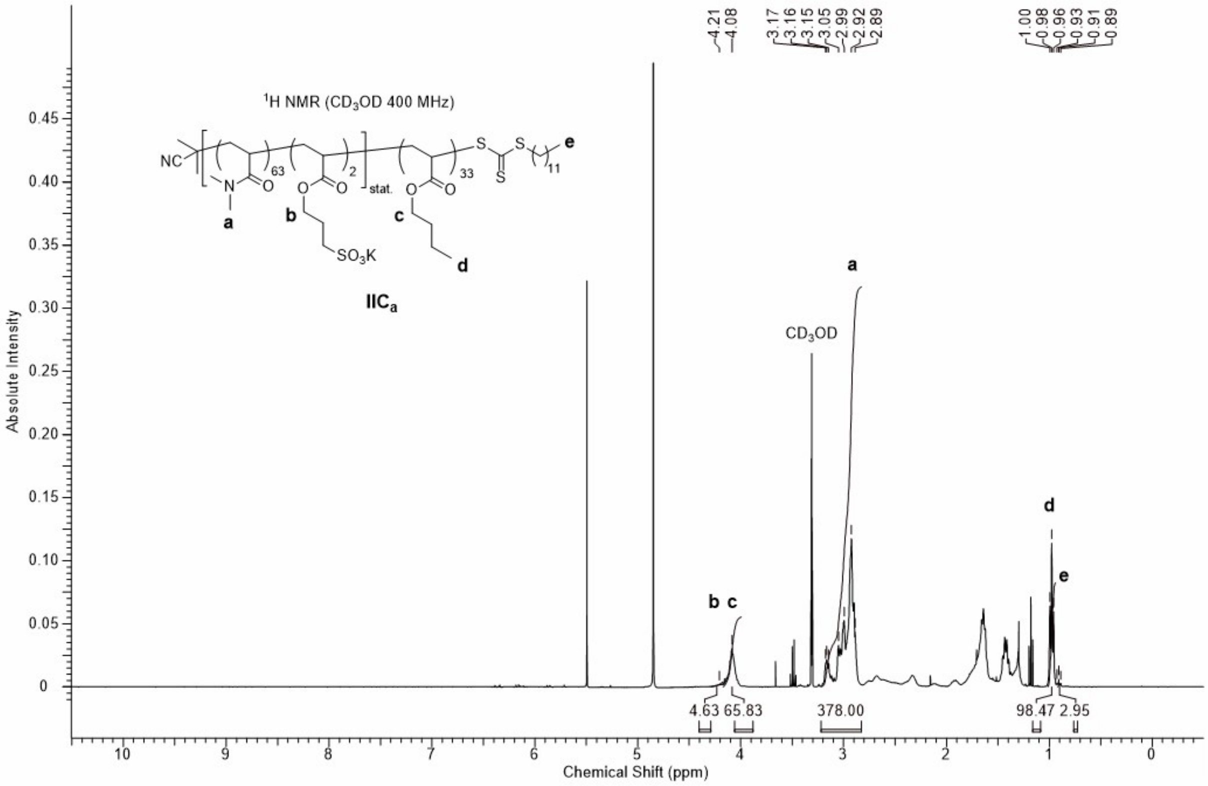

4.21
4.08
3.17
3.16
3.15
3.05
2.99
2.92
2.89
1.00
0.98
0.96
0.93
0.91
0.89
1H NMR (CD3OD 400 MHz)
NC
63
2
stat.
33
S
S
S
11
e
N
O
O
O
O
O
a
b
c
d
SO3K
IICa
CD3OD
a
d
e
b c
4.63
65.83
378.00
98.47
2.95
Absolute Intensity
Chemical Shift (ppm)

u)

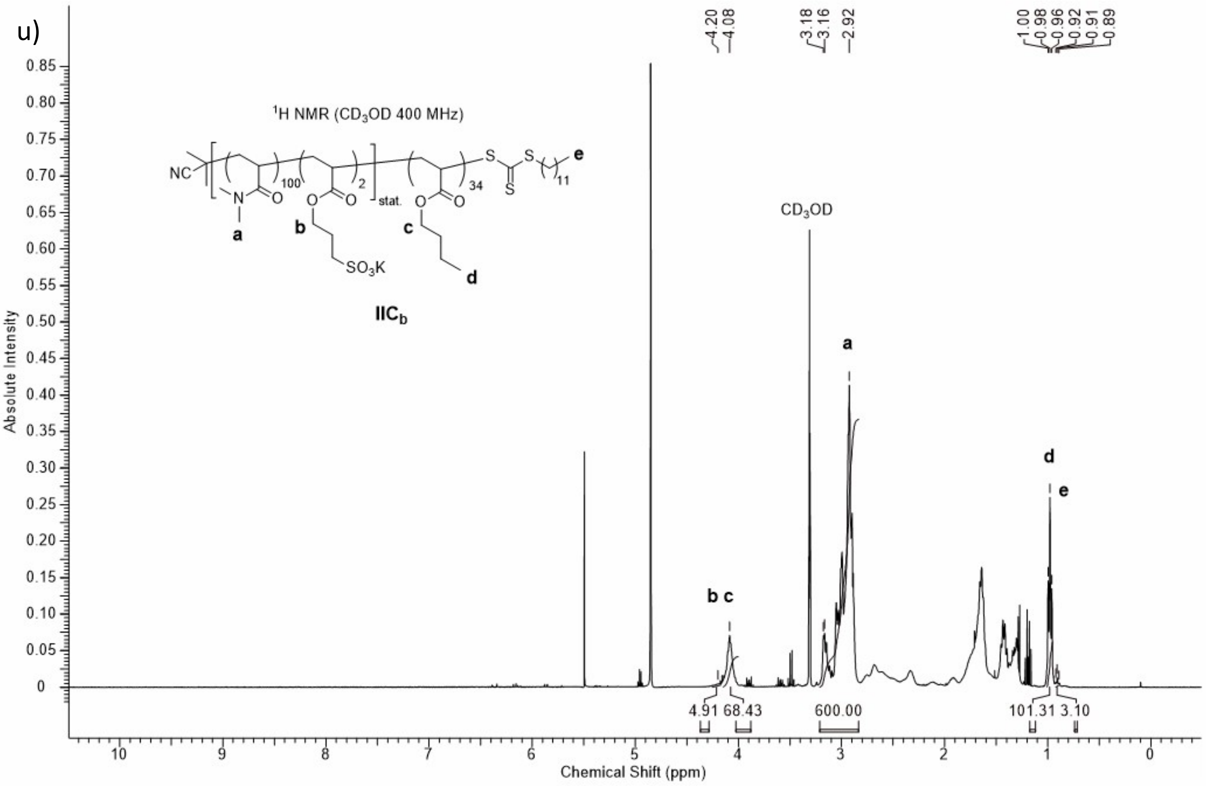

4.20
4.08
3.18
3.16
2.92
1.00
0.98
0.96
0.92
0.91
0.89
1H NMR (CD3OD 400 MHz)
NC
100
2
stat.
34
11
e
a
b
c
d
SO3K
IICb
CD3OD
a
d
e
b c
4.91
68.43
600.00
101.31
3.10
Absolute Intensity
Chemical Shift (ppm)

v)

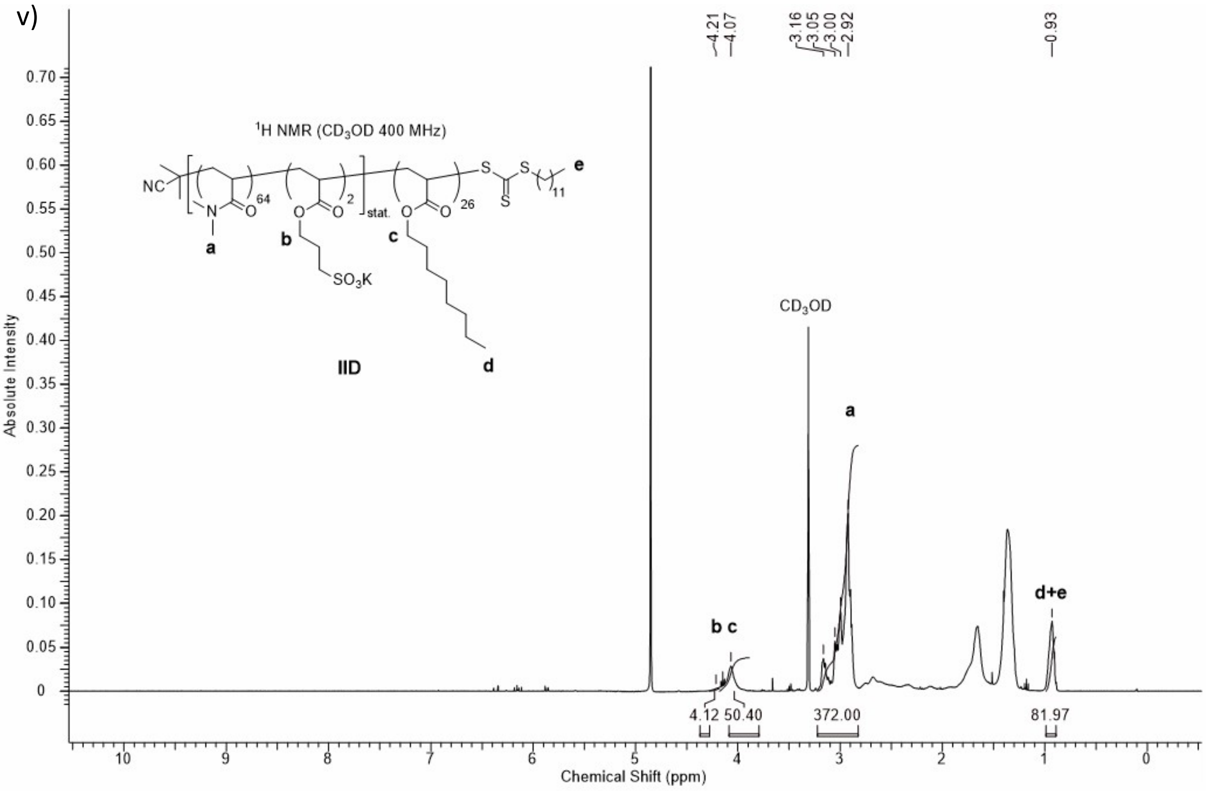

4.21
4.07
3.16
3.05
3.00
2.92
0.93
1H NMR (CD3OD 400 MHz)
NC
64
2
stat.
26
11
e
a
b
c
d
SO3K
IID
CD3OD
a
d+e
b c
4.12
50.40
372.00
81.97
Absolute Intensity
Chemical Shift (ppm)

w)

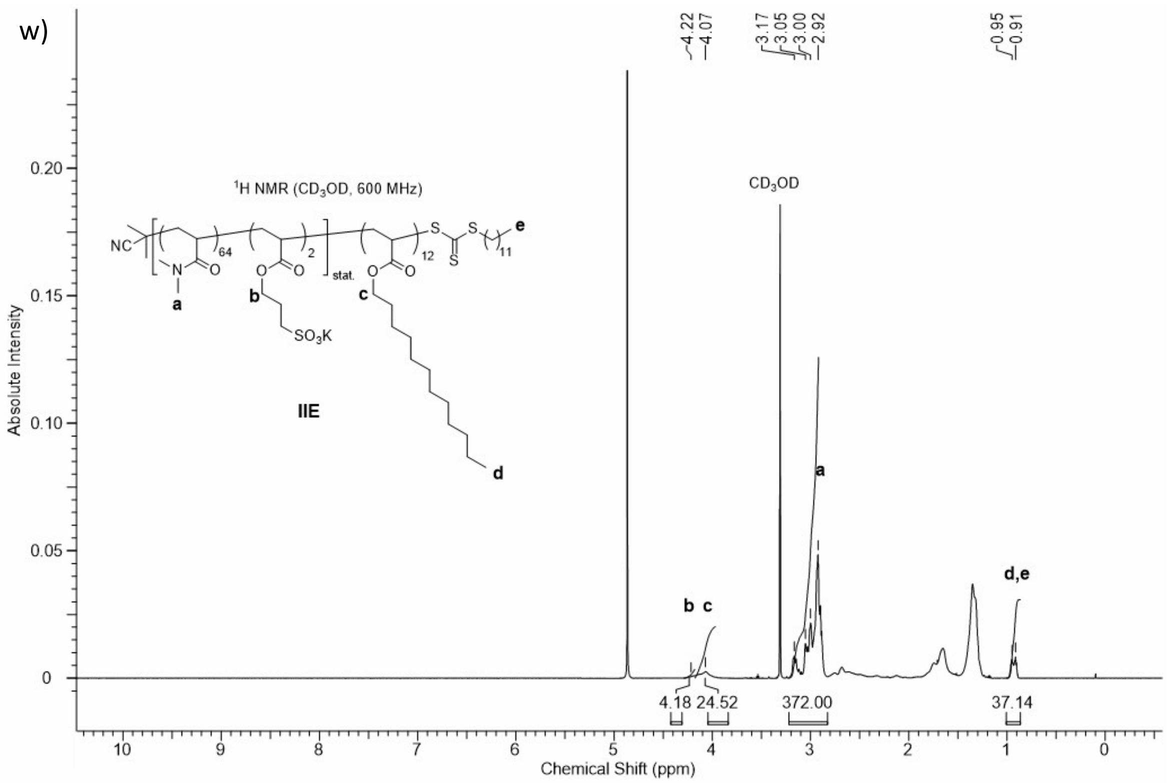

x)

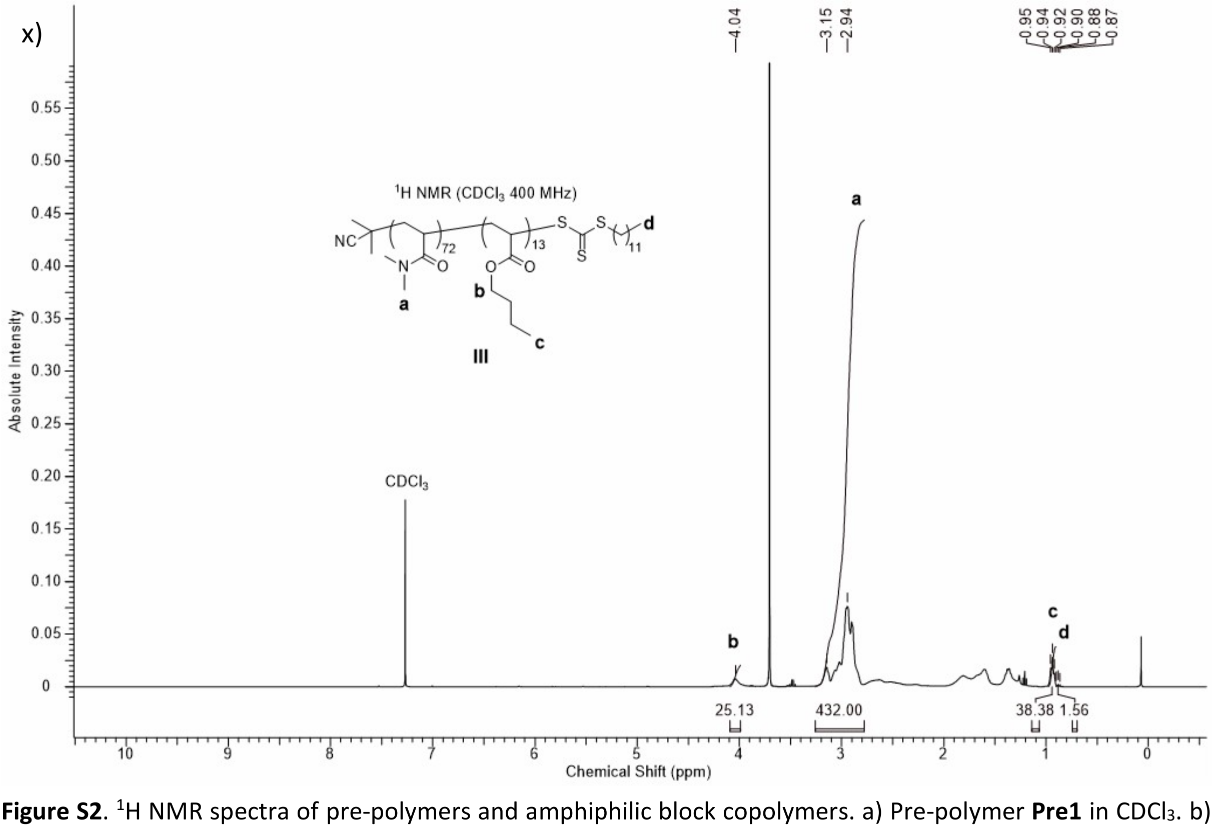

Figure S2. ^{1}H NMR spectra of pre-polymers and amphiphilic block copolymers. a) Pre-polymer **Pre1** in $CDCl_3$. b) Pre-polymer **Pre2** in $CDCl_3$. c) Pre-polymer **Pre3** in $CDCl_3$. d) Pre-polymer **Pre4** in $CDCl_3$. e) Pre-polymer **Pre5** in $CDCl_3$. f) Pre-polymer **Pre6** in $CDCl_3$. g) Pre-polymer **Pre7** in CD_3OD. h) Pre-polymer **Pre8** in CD_3OD. i) Pre-polymer **Pre9** in $CDCl_3$. j) Pre-polymer **Pre10** in CD_3OD. k) Pre-polymer **Pre11** in $CDCl_3$. l) Copolymer **IA** in CD_3OD. m) Copolymer **IB** in CD_3OD. n) Copolymer **IC$_a$** in CD_3OD. o) Copolymer **IC$_b$** in CD_3OD. p) Copolymer **IC$_c$** in CD_3OD. q) Copolymer **ID** in CD_3OD. r) Copolymer **IIA** in $CDCl_3$. s) Copolymer **IIB** in CD_3OD. t) Copolymer **IIC$_a$** in CD_3OD. u) Copolymer **IIC$_b$** in CD_3OD. v) Copolymer **IID** in CD_3OD. w) Copolymer **IIE** in CD_3OD. x) Copolymer **III** in $CDCl_3$. Please note: NMR spectra l)-w) were measured prior to ion exchange and lyophilization.

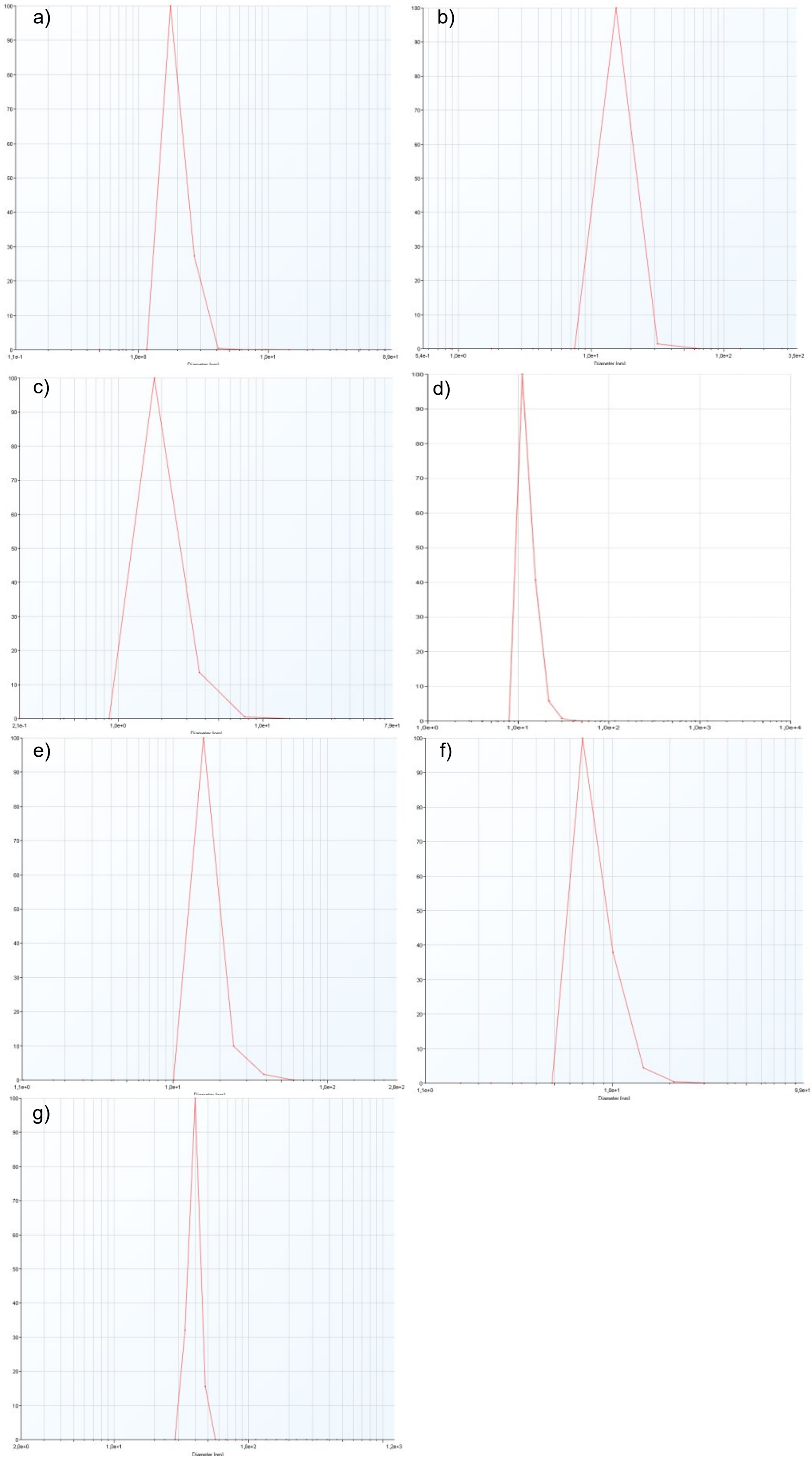

Figure S3. DLS measurements of a 1 mM solution of block copolymers **IA**-**ID** in distilled water. a) Copolymer **IA**. b) Copolymer **IB**. c) Copolymer **IC$_a$**. d) Copolymer **IC$_b$**. e) Copolymer **IC$_c$**. f) Copolymer **ID**. All mixtures were vortexed prior to DLS measurements.

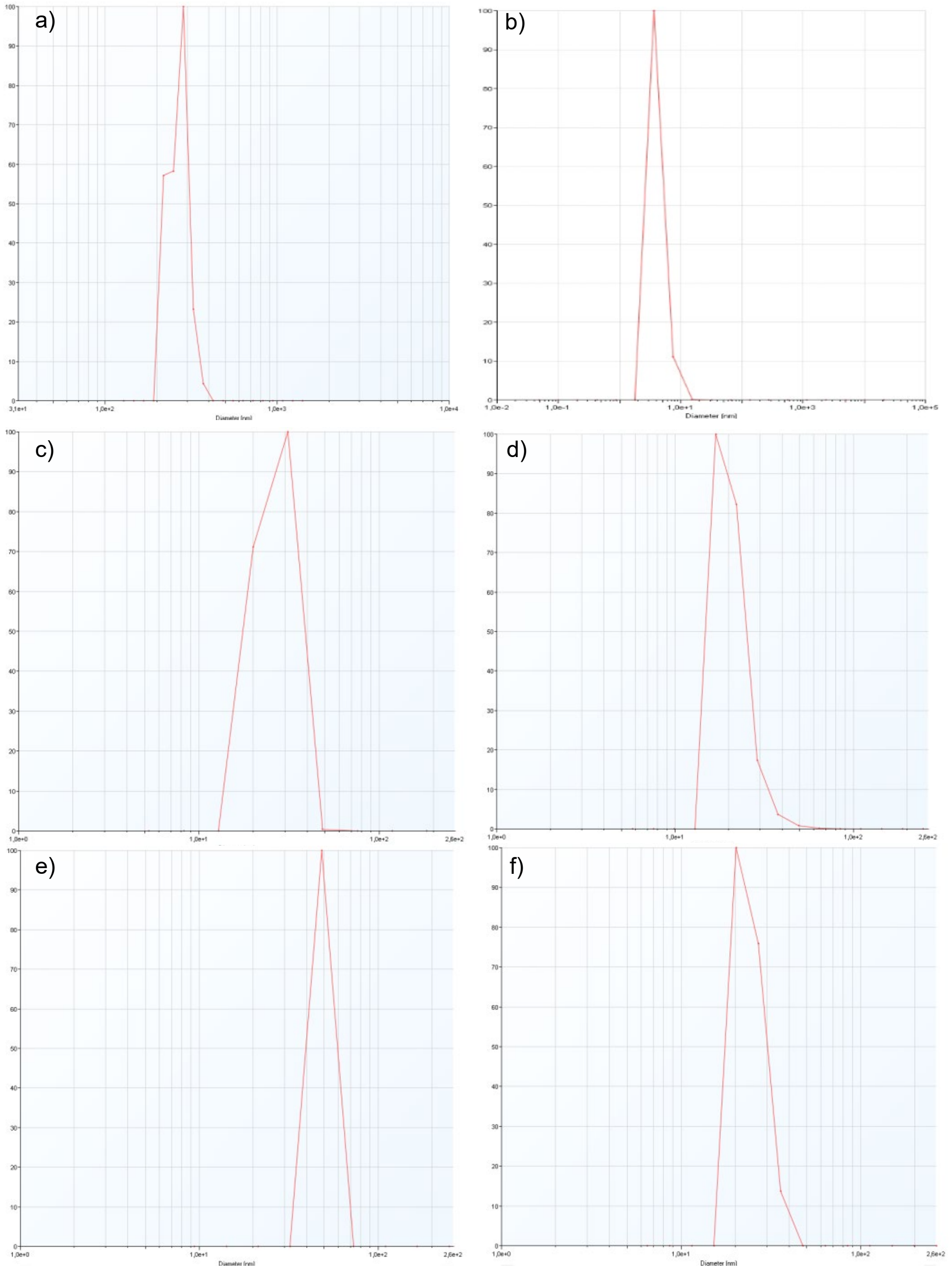

Figure S4. DLS measurements of a 1 mM solution of block copolymers **IIA**-**IIE** in distilled water. a) Copolymer **IIA**. b) Copolymer **IIB**. c) Copolymer **IIC$_{\mathbf{a}}$**. d) Copolymer **IIC$_{\mathbf{b}}$**. e) Copolymer **IID**. f) Copolymer **IIE**. All mixtures were vortexed prior to DLS measurements.

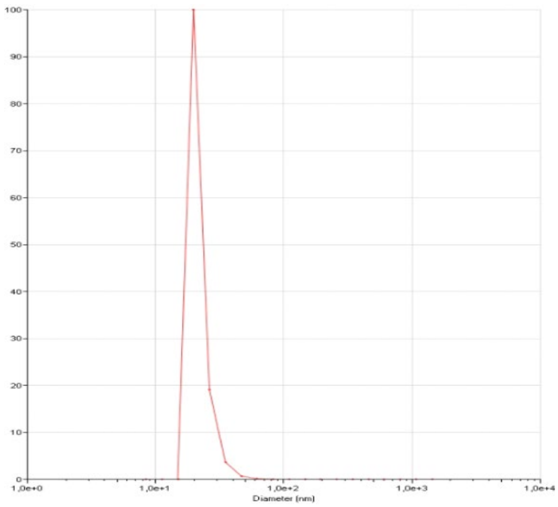

Figure S5. DLS measurements of a 1 mM solution of block copolymer **III** in distilled water.

2.3. Characterization of cocopolymer micelles **I/II** by negative stain transmission electron microscopy and measurement of the zeta potential of selected copolymers micelles

2.3.1. Negative stain transmission electron microscopy

Samples were diluted to a block copolymer **I/II** concentration of 0.25 mM in water. 4 µl of sample were applied onto a previously glow discharged copper grid (Agar Scientific; G2400C) covered with an additional continuous carbon film. After 1 min of incubation the solution was blotted using filter paper (Whatman no. 4) and directly stained with 10 µl of 0.75% uranyl formate solution for 30 s. Grids were imaged using a Tecnai G Spirit electron microscope (FEI) operated at 120 kV and equipped with a CMOS TemCam F416 (TVIPS) detector.

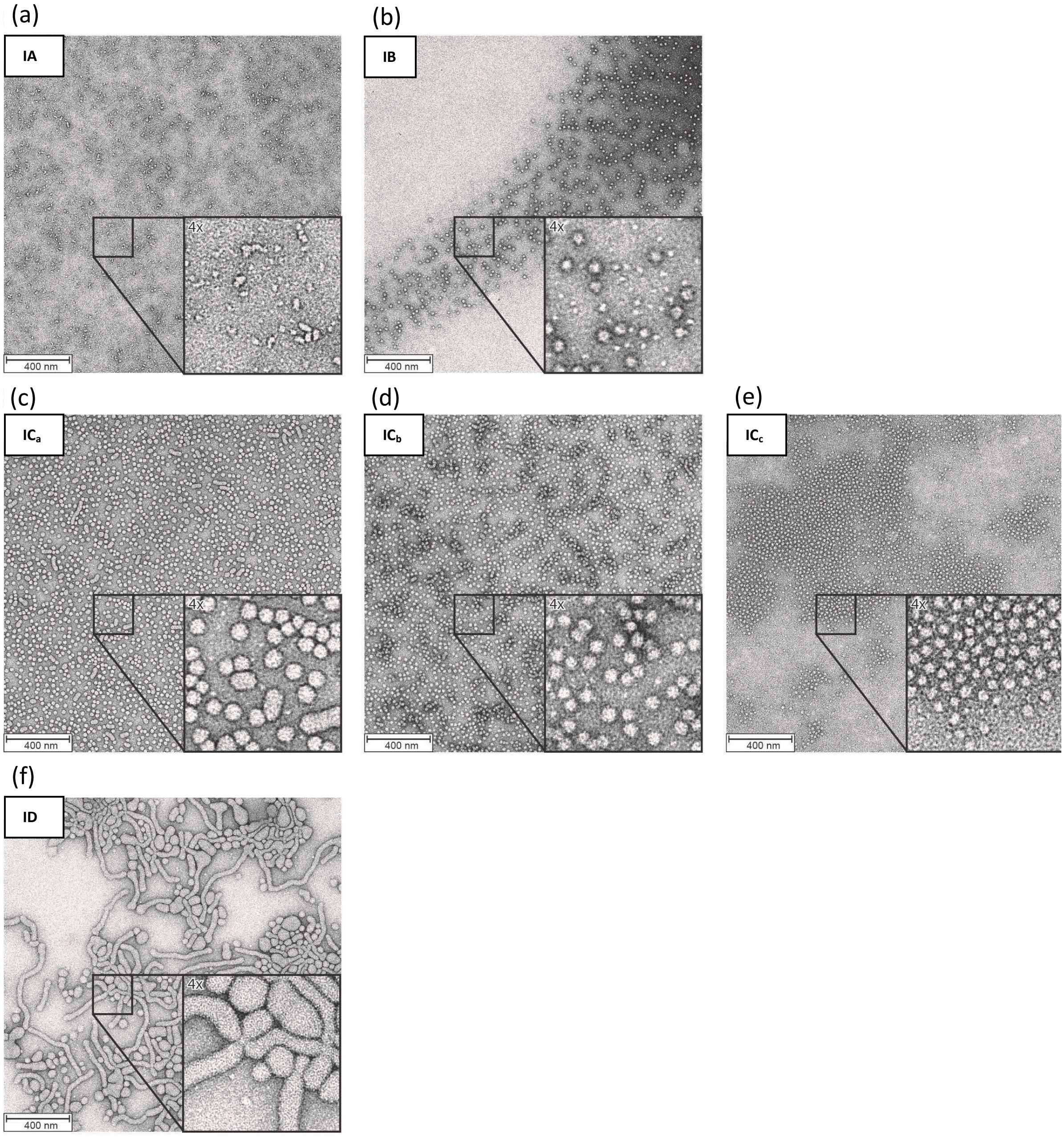

Figure S6. Structural characterization of series **I** cocopolymer micelles by transmission electron microscopy. a-f) Representative micrographs of negatively stained copolymer micelles (scale bar 400 nm). Inserts show 4-fold magnified close-up views of the areas highlighted by black squares. While all copolymers of this series formed micelles, their shape and size varied. a) Copolymer **IA** formed the smallest and most irregularly shaped and sized micelles imaged in this study. b) Copolymer **IB** assembled into spherical micelles with varying diameter. c) Micelles of copolymer **IC$_a$** were spherical with a comparably large diameter or partially elongated forming rod-like structures. d, e) Copolymers **IC$_b$** and **IC$_c$** formed spherical micelles similar to c) but with a defined radius. f) Copolymer **ID** assembled into large and complex structures, ranging from spherical to highly elongated.

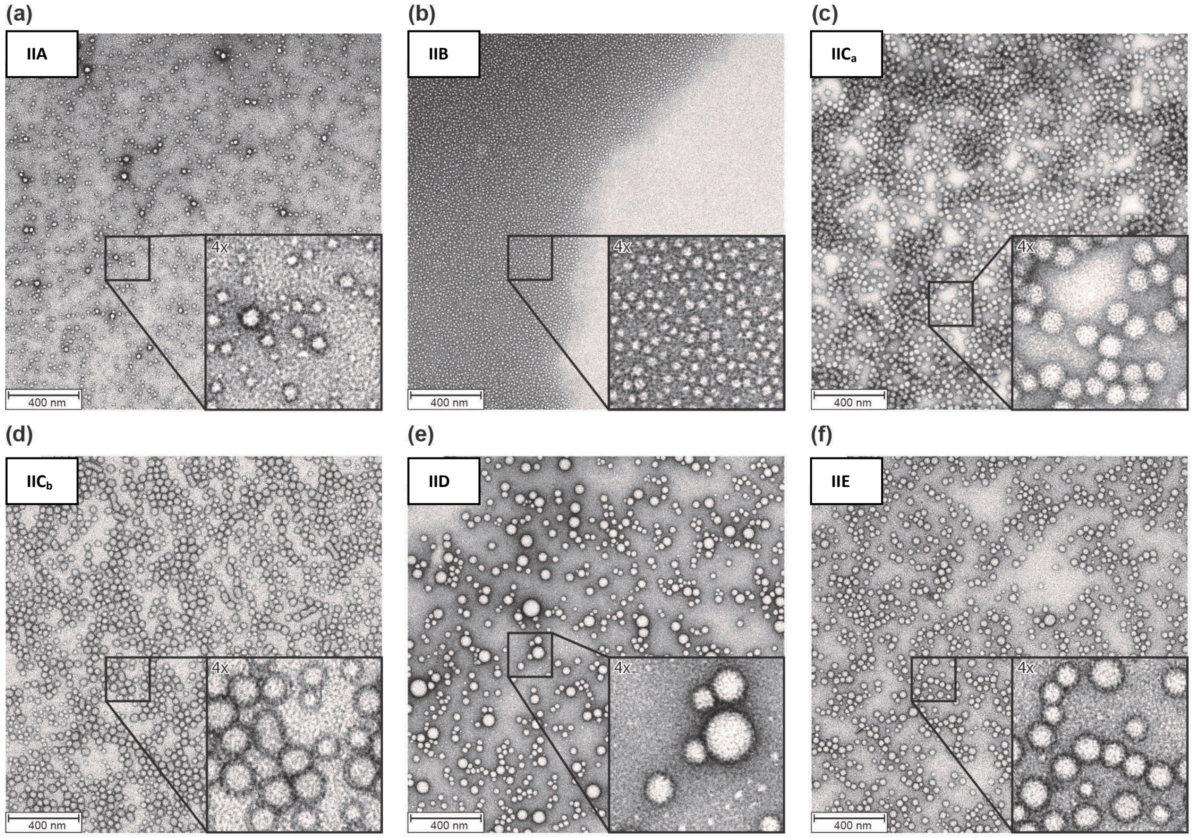

Figure S7. Structural characterization of series **II** copolymer micelles by transmission electron microscopy. a-f) Representative micrographs of negatively stained copolymer micelles (scale bar 400 nm). Inserts show 4-fold magnified close-up views of the areas highlighted by black squares. All copolymers of this series formed spherical micelles, which varied in size. a) The spherical micelles formed by copolymer **IIA** were highly heterogenous in size. b) In contrast, micelles of copolymer **IIB** were small and had a well-defined diameter. c) Copolymer **IIC_a** assembled into highly homogenous, large spherical micelles. d) Micelles formed by copolymer **IICb** were larger and partially elongated. e) Copolymer **IID** formed highly spherical micelles of different diameters. f) Micelles of copolymer **IIE** are most similar to d) with minor variations in diameter.

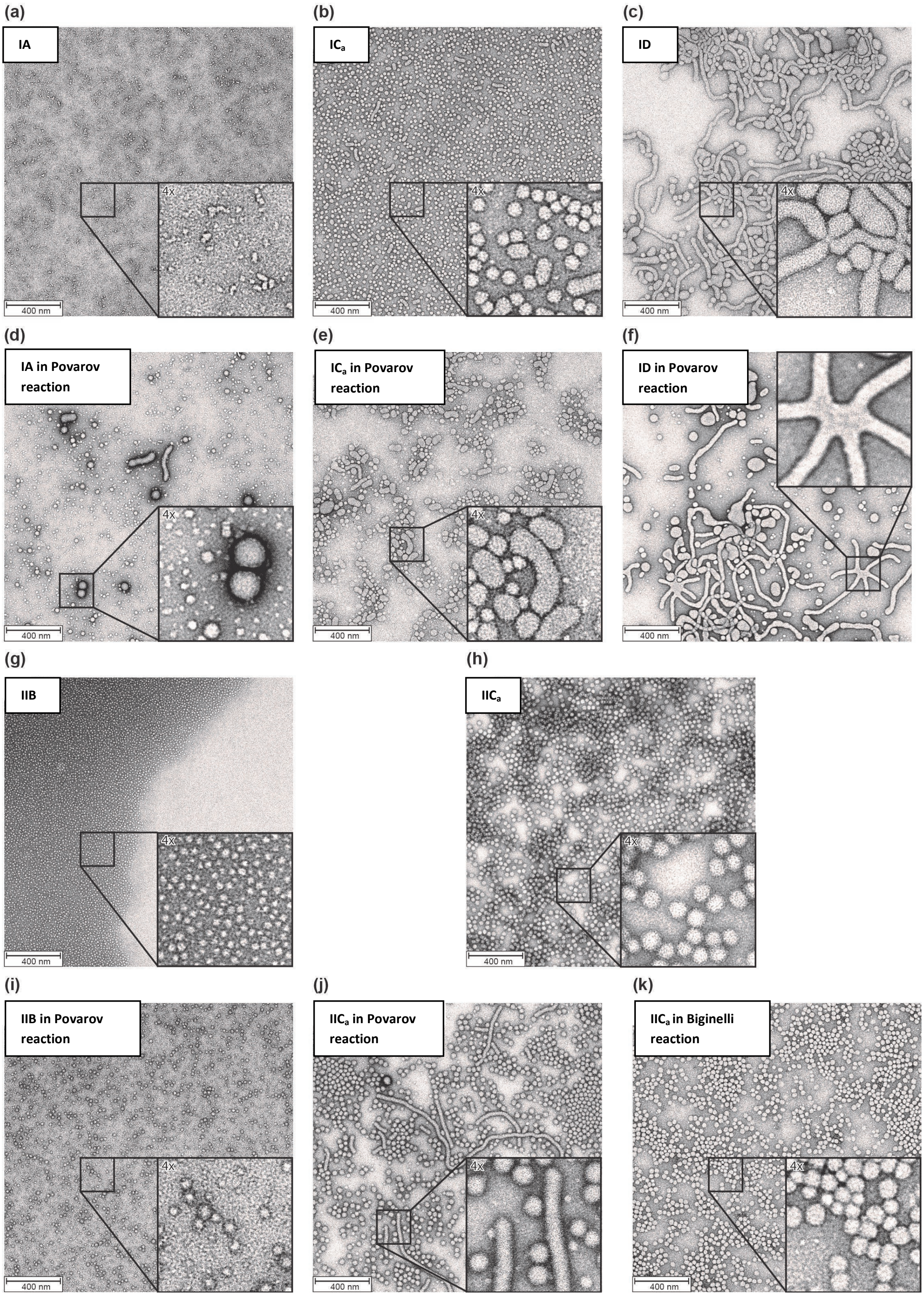

Figure S8. Characterization of the structural transition of copolymer micelles during Povarov and Biginelli reactions, respectively, by transmission electron microscopy. a-k) Representative micrographs of negatively stained copolymer micelles (scale bar 400 nm). Inserts show 4-fold magnified close-up views of the areas highlighted by black squares. a, d) While copolymer **IA** formed tiny, irregular shaped micelles in solution a), the

morphology of micelles changed to significantly larger and partially highly elongated structures during Povarov reaction d). b, e) Round and homogenous micelles were formed by copolymer **IC$_a$** in solution b), they increased in size and got more heterogenous during Povarov reaction e). The already elongated structures formed by copolymer **ID** c), got even more pronounced during Povarov reaction resulting in highly elongated and branched structures f). g, i) Copolymer **IIB** assembled into small spherical micelles in solution g), which kept their shape but increased slightly in size in Povarov reaction i). h, j, k) Micelles formed by copolymer **IIC$_a$** were spherical with a homogenous radius in solution h). While the Povarov reaction had a pronounced effect on the morphology resulting in the formation of highly elongated structures j), micelles did not change their morphology during Biginelli reaction k). Samples of Povarov reaction: **DNA-1**, 50 equivalents of copolymer **I/II**, 8000 equivalents of *tert*-butylaniline **9a** and 8000 equivalents of *N*-Boc-2,3-dihydro-1*H*-pyrrole **10a**. Sample of Biginelli reaction: **DNA-1**, 50 equivalents of copolymer **IIC$_a$**, 8000 equivalents of urea **16** and 8000 equivalents of ethyl acetoacetate **17**.

2.3.2. Zeta potential

Samples were diluted to a block copolymer **I/II** concentration of 1 mM in water and measured at 25 °C.

Table S6. Zeta potential of a 1 mM aqueous solution of selected polymers.

Polymer	Composition [a]	zeta potential [b] [mV]
**IC$_a$**	DMA_{65}-(BA_{33}-SPA_2)	-19.01 ± 0.63
**IC$_b$**	DMA_{63}-(BA_{27}-SPA_2)	-25.39 ± 0.71
**IIA**	(DMA_{62}-SPA_2)-MMA_{14}	-14.44 ± 0.80
**IIB**	(DMA_{60}-SPA_2)-EA_{23}	-19.92 ± 1.00
**IIC$_a$**	(DMA_{63}-SPA_2)-BA_{33}	-27.02 ± 0.52
**IIC$_b$**	(DMA_{100}-SPA_2)-BA_{34}	-10.65 ± 1.11
**IID**	(DMA_{62}-SPA_2)-Oa_{26}	-33.75 ± 0.98
**III**	DMA_{72}-BA_9	-9.03 ± 0.35

[a] determined via ^{1}H NMR spectroscopy; [b] 1mM aqueous polymer solution.

3. DNA oligonucleotides used for reaction kinetics studies and characterization of DNA-copolymer micelle interaction

Table S7. DNA oligonucleotides used for characterization of DNA-copolymer **IIB** interaction and for reaction kinetics studies.

DNA	sequence (5`-3`)	complementary sequence (5`-3`)	sketch
**DNA-1**	**R-linker**-GTC TTG CCG AAT TC	-	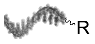
**DNA-2**	**R-linker**-GTC TTG CCG AAT TC	GAA TTC GGC AAG AC	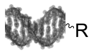
**DNA-3**	**R-linker**-GTC TTG CCG AAT TC	**Alexa**-GAA TTC GGC AAG AC	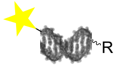
**DNA-4**	**R-linker**-GTC TTG CCG AAT TC	GAA TTC GGC AAG AC-**pyrene**	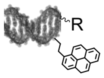
**DNA-5**	**R-linker**-GTC TTG CCG AAT TC	**pyrene**-GAA TTC GGC AAG AC	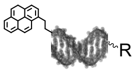
**DNA-6**	**linker**-GTC TTG CCG AAT TC	GAA TTC GGC AAG AC	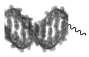
**DNA-7**	**linker**-GTC TTG CCG AAT TC	**Alexa**-GAA TTC GGC AAG AC	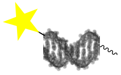
**DNA-8**	GTC TTG CCG AAT TC	GAA TTC GGC AAG AC	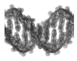
**DNA-9**	**linker**-GTC TTG CCG AAT TC	**pyrene**-GAA TTC GGC AAG AC	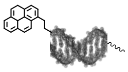
**DNA-10**	**linker**-GTC TTG CCG AAT TC	GAA TTC GGC AAG AC-**pyrene**	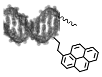
**DNA-11**	GTC TTG CCG AAT TC	**pyrene**-GAA TTC GGC AAG AC	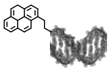
**DNA-12**	GTC TTG CCG AAT TC	GAA TTC GGC AAG AC-**pyrene**	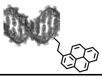

R = 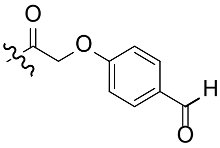 ; **linker**/ wavy bond: 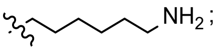 ; = Alexa Fluor 430 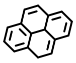 = pyrene

4. Micellar Brønsted acid-mediated synthesis of DNA-hexahydro-1*H*-pyrrolo-[3,2-*c*]quinoline conjugates **DNA-11, DNA-18-21** and DNA-hexahydro-1*H*-pyrano[3,2-*c*]quinoline conjugates **DNA-12**

4.1. Procedure for coupling of carboxylic acids to 5’-aminolinker-DNA–conjugates

The DMt-protective group of 5’-aminolinker-modified DNA (5`-GTC TTG CCG AAT TC-3`) bound to 1000 Å controlled pore glass (CPG) solid support (1 µmol, ca. 40 mg) was removed by addition of 3% trichloroacetic acid in dry CH_2Cl_2 (3 x 200 µL) for 3 x 1 min. A yellow to orange color indicated a successful removal of the protective group. The CPG containing the amine-deprotected DNA was washed three times with each 200 µL of 1% TEA in MeCN, DMF, MeOH, MeCN, and CH_2Cl_2. The CPG, a carboxylic acid, and HATU were then dried *in vacuo* for 15 min. Stock solutions of all reactants in dry DMF were prepared immediately before the reaction was started. To 300 µL of a solution of a carboxylic acid (100 µmol, 100 eq.) in dry DMF were added HATU (38 mg, 100 µmol, 100 eq.) dissolved in 300 µL of dry DMF and DIPEA (42 µL, 250 µmol, 250 eq.). This reaction mixture was shaken for 5 min and added to the solid support-bound DNA suspended in dry DMF (300 µL). The amide coupling reaction was shaken at room temperature for 4 h. Then, the CPG containing the DNA-conjugate was filtered over a filter column and washed subsequently with each 3 x 200 µL of DMF, MeOH, MeCN, and CH_2Cl_2. Unreacted amines were capped with acetic acid anhydride (a 1:1 mixture of THF/methylimidazole, 9:1, vol/vol, and THF/pyridine/acetic acid anhydride, 8:1:1, vol/vol was used), and the CPG was washed again with each 3 x 200 µL of DMF, MeOH, MeCN, and CH_2Cl_2, and dried *in vacuo* for 15 min. For purification, the DNA-conjugate was deprotected and cleaved from the CPG by treatment with 500 µL of AMA (AMA= aqueous ammonia (30%)/ aqueous methylamine (40%), 1:1, vol/vol) for 4 h at room temperature. To this solution 20 µL of 1 M Tris buffer (pH= 7.5) were added, the mixture was dried in a SpeedVac, redissolved in 200 µL of distilled water, and the product was purified by RP-HPLC (Gemini, 5u, C18, 110 Å column; 100*10.0 mm) with a gradient of aqueous triethylammonium acetate buffer (100 mM, pH= 8) and methanol (20% - 70% of methanol over 13 min).

4.1.1. Synthesis of DNA-aldehyde conjugate **DNA-1**

DNA strand (250 nmol, 5`-GTC TTG CCG AAT TC-3`) was coupled with 2-(4-formylphenoxy)acetic acid **22** (4.5 mg, 25 µmol, 100 eq.) to furnish **DNA-1** according to the procedure for coupling of carboxylic acids to amino-modified DNA. For analysis, an aliquot of ca. 10 nmol of the conjugate was deprotected and cleaved from the CPG with 500 µL of AMA (AMA= aqueous ammonia (30%)/ aqueous methylamine

(40%), 1:1, vol/vol) for 4 h at room temperature. Then, 20 µL of 1 M Tris buffer (pH= 7.5) were added, the product was dried in a SpeedVac, redissolved in 100 µL of distilled water, and purified by RP-HPLC (Gemini, 5u, C18, 110 Å column; 100*10.0 mm) with a gradient of aqueous triethylammonium acetate buffer (100 mM, pH= 8) and methanol (20% - 70% of methanol over 13 min). After purification, fractions containing the desired product were collected, evaporated, and co-evaporated with 3 x 200 µL of ethanol in a SpeedVac, and then precipitated twice from ethanol.

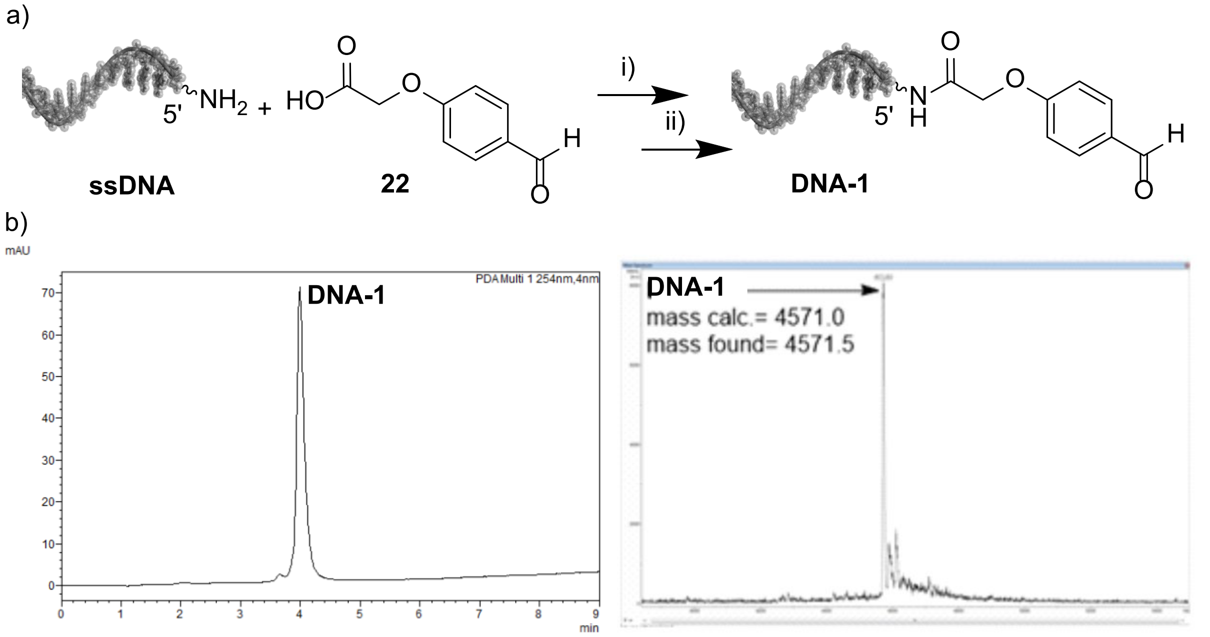

Figure S9. Synthesis of the DNA-aldehyde conjugate **DNA-1**. a) Scheme of the synthesis of **DNA-1**. b) HPLC trace of the aldehyde **DNA-1** (left hand trace) and MALDI-MS spectrum of the aldehyde **DNA-1** (right hand spectrum). Reagents and conditions: i) HATU, DIPEA, dry DMF, room temperature, 4 h; ii) AMA (aqueous ammonia (30%)/ aqueous methylamine (40%), 1:1, vol/vol), 4 h, room temperature. ss= single stranded.

4.2. Procedure for micelle-mediated synthesis of DNA-hexahydro-1*H*-pyrrolo[3,2-c]quinoline conjugates **DNA-11a,18-21**[3]

To a solution of single stranded (ss) DNA-aldehyde conjugate **DNA-1** or double stranded (ds) DNA-aldehyde conjugate **DNA-2-5** (500 pmol) in distilled water were added 4-*tert*-butylaniline **9a** (1 µmol, 2000 eq.) dissolved in 1.5 µL of ethyl acetate (taken from a stock solution: 100 µmol dissolved in 150 µL of ethyl acetate), *N*-Boc-2,3-dihydro-1*H*-pyrrole **10a** (1 µmol, 2000 eq.) dissolved in 1 µL of ethyl acetate (taken from a stock solution: 100 µmol dissolved in 100 µL of ethyl acetate), and copolymer micelle **I/II** (25 nmol, 50 eq.) dissolved in 16 µL of distilled water taken from an aqueous stock solution (1.25 µmol in 800 µL). The reaction mixtures were filled with distilled water to a volume of 50 µL giving a final concentration of 0.5 mM of copolymer **I/II**. The reaction mixtures were shaken at temperatures and reaction times given in Tables S8-S13. Then, 70 µL of distilled water were added and the reaction mixtures were extracted with ethyl acetate (6 x 200 µL). The aqueous solution was evaporated in a SpeedVac, the residue was redissolved in 45 µL of distilled water. All coupling products **DNA-11a,18-21** were analyzed by RP-HPLC (Phenomenex, Gemini; 5 µm, C18, 110 Å, 100*4.6 mm) with a gradient of aqueous triethylammonium acetate buffer (10 mM, pH= 8) and methanol (10% - 60% of methanol over 9 min, followed by 60% - 100% of methanol over 1 min), and by MALDI-MS analysis. The conversion was estimated based on the area under the curve of the product peak versus the starting material peak in the HPLC-trace and by MALDI-MS.

4.3. Copolymer micelle **I/II**-mediated Povarov reaction of aldehyde conjugate **DNA-1**, *tert*-butylaniline **9a**, and *N*-Boc-2,3-dihydro-1*H*-pyrrole **10a** to DNA-conjugate **DNA-11a** at room temperature

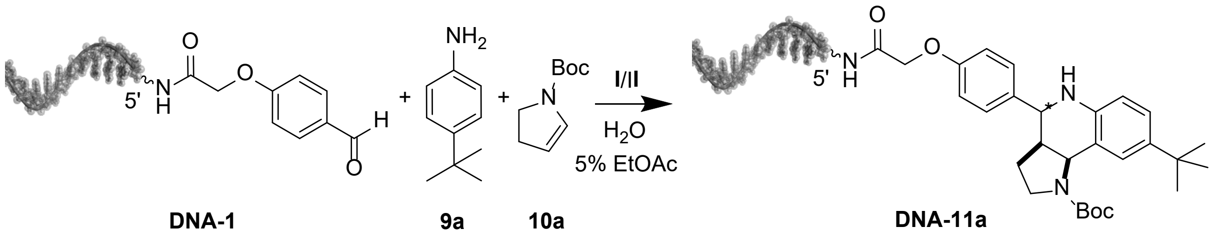

Scheme S6. Reaction scheme of the copolymer micelle **I/II**-mediated Povarov reaction to **DNA-11a**.

Table S8. Impact of copolymer micelle **I/II** design on synthesis of DNA-hexahydro-1*H*-pyrrolo[3,2-*c*]quinoline conjugate **DNA-11a**. Reaction conditions: 2000 eq. of **9a** and 2000 eq. of **10a**, 50 eq. of **I/II**, 0.5 mM **I/II**, room temperature.

No.	copolymer name	copolymer composition	t [h]	**DNA-11a** [%][a]
1	**IA**	DMA_{62}-(MMA_{25}-SPA_2)	0.5	47
2[b]	**IA**	DMA_{62}-(MMA_{25}-SPA_2)	1	58(10)
3	**IA**	DMA_{62}-(MMA_{25}-SPA_2)	2	75(7)
4	**IA**	DMA_{62}-(MMA_{25}-SPA_2)	4	93
5	**IB**	DMA_{62}-(EA_{23}-SPA_2)	0.5	45
6[b]	**IB**	DMA_{62}-(EA_{23}-SPA_2)	1	58(7)
7	**IB**	DMA_{62}-(EA_{23}-SPA_2)	2	77(5)
8	**IB**	DMA_{62}-(EA_{23}-SPA_2)	4	92
9	**IC$_a$**	DMA_{65}-(BA_{33}-SPA_2)	0.5	27(16)
10[b]	**IC$_a$**	DMA_{65}-(BA_{33}-SPA_2)	1	45(17)
11	**IC$_a$**	DMA_{65}-(BA_{33}-SPA_2)	2	47(24)
12	**IC$_a$**	DMA_{65}-(BA_{33}-SPA_2)	4	64(21)
13	**IC$_b$**	DMA_{63}-(BA_{27}-SPA_2)	0.5	30(12)
14[b]	**IC$_b$**	DMA_{63}-(BA_{27}-SPA_2)	1	54(11)
15	**IC$_b$**	DMA_{63}-(BA_{27}-SPA_2)	2	69(11)
16	**IC$_b$**	DMA_{63}-(BA_{27}-SPA_2)	4	84(8)
17	**IC$_c$**	DMA_{132}-(BA_{23}-SPA_2)	0.5	33(16)

18[b]	**IC_c**	DMA_{132}-(BA_{23}-SPA_2)	1	48(17)
19	**IC_c**	DMA_{132}-(BA_{23}-SPA_2)	2	74(12)
20	**IC_c**	DMA_{132}-(BA_{23}-SPA_2)	4	85(10)
21	**ID**	DMA_{62}-(OA_{26}-SPA_2)	0.5	28(19)
22[b]	**ID**	DMA_{62}-(OA_{26}-SPA_2)	1	53(11)
23	**ID**	DMA_{62}-(OA_{26}-SPA_2)	2	65(9)
24	**ID**	DMA_{62}-(OA_{26}-SPA_2)	4	81(8)
25	**IIA**	(DMA_{62}-SPA_2)-MMA_{14}	0.5	39(12)
26[b]	**IIA**	(DMA_{62}-SPA_2)-MMA_{14}	1	66(10)
27	**IIA**	(DMA_{62}-SPA_2)-MMA_{14}	2	76(8)
28	**IIA**	(DMA_{62}-SPA_2)-MMA_{14}	4	90(5)
29	**IIB**	(DMA_{60}-SPA_2)-EA_{23}	0.5	40(10)
30[b]	**IIB**	(DMA_{60}-SPA_2)-EA_{23}	1	64(12)
31	**IIB**	(DMA_{60}-SPA_2)-EA_{23}	2	77(12)
32	**IIB**	(DMA_{60}-SPA_2)-EA_{23}	4	92
33	**IIC_a**	(DMA_{63}-SPA_2)-BA_{33}	0.5	45
34[b]	**IIC_a**	(DMA_{63}-SPA_2)-BA_{33}	1	70(8)
35	**IIC_a**	(DMA_{63}-SPA_2)-BA_{33}	2	83(5)
36	**IIC_a**	(DMA_{63}-SPA_2)-BA_{33}	4	93
37	**IIC_b**	(DMA_{100}-SPA_2)-BA_{34}	0.5	35(25)
38[b]	**IIC_b**	(DMA_{100}-SPA_2)-BA_{34}	1	44(32)
39	**IIC_b**	(DMA_{100}-SPA_2)-BA_{34}	2	59(31)
40	**IIC_b**	(DMA_{100}-SPA_2)-BA_{34}	4	66(30)
41	**IID**	(DMA_{62}-SPA_2)-OA_{26}	0.5	39(16)
42[b]	**IID**	(DMA_{62}-SPA_2)-OA_{26}	1	63(13)
43	**IID**	(DMA_{62}-SPA_2)-OA_{26}	2	70(12)
44	**IID**	(DMA_{62}-SPA_2)-OA_{26}	4	87(6)
45	**IIE**	(DMA_{62}-SPA_2)-DDA_{12}	0.5	29(23)
46[b]	**IIE**	(DMA_{62}-SPA_2)-DDA_{12}	1	62(8)
47	**IIE**	(DMA_{62}-SPA_2)-DDA_{12}	2	69(11)
48	**IIE**	(DMA_{62}-SPA_2)-DDA_{12}	4	80(10)
49	**III**	DMA_{72}-BA_{13}	4	46(16)
50	-	-	0.5	8(4)
51	-	-	1	16(8)
52	-	-	2	24(14)

53	-	-	4	43(19)
54[c]	-	-	4	46(28)

[a] HPLC analysis of the crude, missing percentage to 100%: mainly **DNA-1**; [b] experiments were set up in triplicates, see Table S10; [c] experiment **No. 54** was set up without EtOAc cosolvent. Conversions in parentheses show those of a later eluting side product.

4.3.1. Head-to head comparison of copolymer micelles **I/II** in synthesis of DNA-hexahydro-1*H*-pyrrolo[3,2-*c*]quinoline conjugate **DNA-11a** at different reaction times

Table S9. Impact of copolymer micelle **I/II** design on synthesis of DNA-hexahydro-1*H*-pyrrolo[3,2-*c*]quinoline conjugate **DNA-11a** at 0.5 hour reaction time. Reaction conditions: 2000 eq. of **9a** and 2000 eq. of **10a**, 50 eq. of **I/II**, 0.5 mM **I/II**, room temperature.

No.	copolymer name	copolymer composition	**DNA-11a** [%][a]
1	**IA**	DMA_{62}-(MMA_{25}-SPA_{2})	47
2	**IB**	DMA_{62}-(EA_{23}-SPA_{2})	45
3	**IC$_a$**	DMA_{65}-(BA_{33}-SPA_{2})	27(16)
4	**IC$_b$**	DMA_{63}-(BA_{27}-SPA_{2})	30(12)
5	**IC$_c$**	DMA_{132}-(BA_{23}-SPA_{2})	33(16)
6	**ID**	DMA_{62}-(OA_{26}-SPA_{2})	28(19)
7	**IIA**	(DMA_{62}-SPA_{2})-MMA_{14}	39(12)
8	**IIB**	(DMA_{60}-SPA_{2})-EA_{23}	40(10)
9	**IIC$_a$**	(DMA_{63}-SPA_{2})-BA_{33}	45
10	**IIC$_b$**	(DMA_{100}-SPA_{2})-BA_{34}	35(25)
11	**IID**	(DMA_{62}-SPA_{2})-OA_{26}	39(16)
12	**IIE**	(DMA_{62}-SPA_{2})-DDA_{12}	29(23)

[a] HPLC analysis of the crude, missing percentage to 100%: mainly **DNA-1**. Conversions in parentheses show those of a later eluting side product.

Table S10. Impact of copolymer micelle **I/II** design on synthesis of DNA-hexahydro-1*H*-pyrrolo[3,2-*c*]quinoline conjugate **DNA-11a** at 1 hour reaction time. Reaction conditions: 2000 eq. of **9a** and 2000 eq. of **10a**, 50 eq. of **I/II**, 0.5 mM **I/II**, room temperature.

No.	copolymer name	copolymer composition	**DNA-11a** 1x [%][a]	**DNA-11a** 2x [%][a]	**DNA-11a** 3x [%][a]	**DNA-11a** average [%][a]
1	**IA**	DMA_{62}-(MMA_{25}-SPA_2)	57(13)	61(8)	56(9)	58(10)
2	**IB**	DMA_{62}-(EA_{23}-SPA_2)	57(6)	61(7)	56(7)	58(7)
3	**IC_a**	DMA_{65}-(BA_{33}-SPA_2)	48(15)	50(11)	37(25)	45(17)
4	**IC_b**	DMA_{63}-(BA_{27}-SPA_2)	54(10)	57(11)	51(13)	54(11)
5	**IC_c**	DMA_{132}-(BA_{23}-SPA_2)	54(11)	48(20)	43(19)	48(17)
6	**ID**	DMA_{62}-(OA_{26}-SPA_2)	54(9)	52(13)	54(10)	53(11)
7	**IIA**	(DMA_{62}-SPA_2)-MMA_{14}	65(8)	67(11)	65(10)	66(10)
8	**IIB**	(DMA_{60}-SPA_2)-EA_{23}	64(13)	64(12)	65(12)	64(12)
9	**IIC_a**	(DMA_{63}-SPA_2)-BA_{33}	68(11)	70(5)	71(8)	70(8)
10	**IIC_b**	(DMA_{100}-SPA_2)-BA_{34}	44(31)	46(31)	42(33)	44(32)
11	**IID**	(DMA_{62}-SPA_2)-OA_{26}	64(12)	61(13)	63(14)	63(13)
12	**IIE**	(DMA_{62}-SPA_2)-DDA_{12}	64(8)	61(11)	59(6)	61(8)

[a] HPLC analysis of the crude, missing percentage to 100%: mainly **DNA-1**. Conversions in parentheses show those of a later eluting side product.

Table S11. Impact of copolymer micelle **I/II** design on synthesis of DNA-hexahydro-1*H*-pyrrolo[3,2-*c*]quinoline conjugate **DNA-11a** at 2 hour reaction time. Reaction conditions: 2000 eq. of **9a** and 2000 eq. of **10a**, 50 eq. of **I/II**, 0.5 mM **I/II**, room temperature.

No.	copolymer name	copolymer composition	**DNA-11a** [%][a]
1	**IA**	DMA_{62}-(MMA_{25}-SPA_2)	75(7)
2	**IB**	DMA_{62}-(EA_{23}-SPA_2)	77(5)
3	**IC$_a$**	DMA_{65}-(BA_{33}-SPA_2)	47(24)
4	**IC$_b$**	DMA_{63}-(BA_{27}-SPA_2)	69(11)
5	**IC$_c$**	DMA_{132}-(BA_{23}-SPA_2)	74(12)
6	**ID**	DMA_{62}-(OA_{26}-SPA_2)	65(9)
7	**IIA**	(DMA_{62}-SPA_2)-MMA_{14}	76(8)
8	**IIB**	(DMA_{60}-SPA_2)-EA_{23}	77(12)
9	**IIC$_a$**	(DMA_{63}-SPA_2)-BA_{33}	83(5)
10	**IIC$_b$**	(DMA_{100}-SPA_2)-BA_{34}	59(31)
11	**IID**	(DMA_{62}-SPA_2)-OA_{26}	70(12)
12	**IIE**	(DMA_{62}-SPA_2)-DDA_{12}	69(11)

[a] HPLC analysis of the crude, missing percentage to 100%: mainly **DNA-1**. Conversions in parentheses show those of a later eluting side product.

Table S12. Impact of copolymer micelle **I/II** design on synthesis of DNA-hexahydro-1*H*-pyrrolo[3,2-*c*]quinoline conjugate **DNA-11a** at 4 hour reaction time. Reaction conditions: 2000 eq. of **9a** and 2000 eq. of **10a**, 50 eq. of **I/II**, 0.5 mM **I/II**, room temperature.

No.	copolymer name	copolymer composition	**DNA-11a** [%][a]
1	**IA**	DMA_{62}-(MMA_{25}-SPA_2)	93
2	**IB**	DMA_{62}-(EA_{23}-SPA_2)	92
3	**IC$_a$**	DMA_{65}-(BA_{33}-SPA_2)	64(21)
4	**IC$_b$**	DMA_{63}-(BA_{27}-SPA_2)	84(8)
5	**IC$_c$**	DMA_{132}-(BA_{23}-SPA_2)	85(10)
6	**ID**	DMA_{62}-(OA_{26}-SPA_2)	81(8)
7	**IIA**	(DMA_{62}-SPA_2)-MMA_{14}	90(5)
8	**IIB**	(DMA_{60}-SPA_2)-EA_{23}	92
9	**IIC$_a$**	(DMA_{63}-SPA_2)-BA_{33}	93
10	**IIC$_b$**	(DMA_{100}-SPA_2)-BA_{34}	66(30)
11	**IID**	(DMA_{62}-SPA_2)-OA_{26}	87(6)
12	**IIE**	(DMA_{62}-SPA_2)-DDA_{12}	80(10)

[a] HPLC analysis of the crude, missing percentage to 100%: mainly **DNA-1**. Conversions in parentheses show those of a later eluting side product.

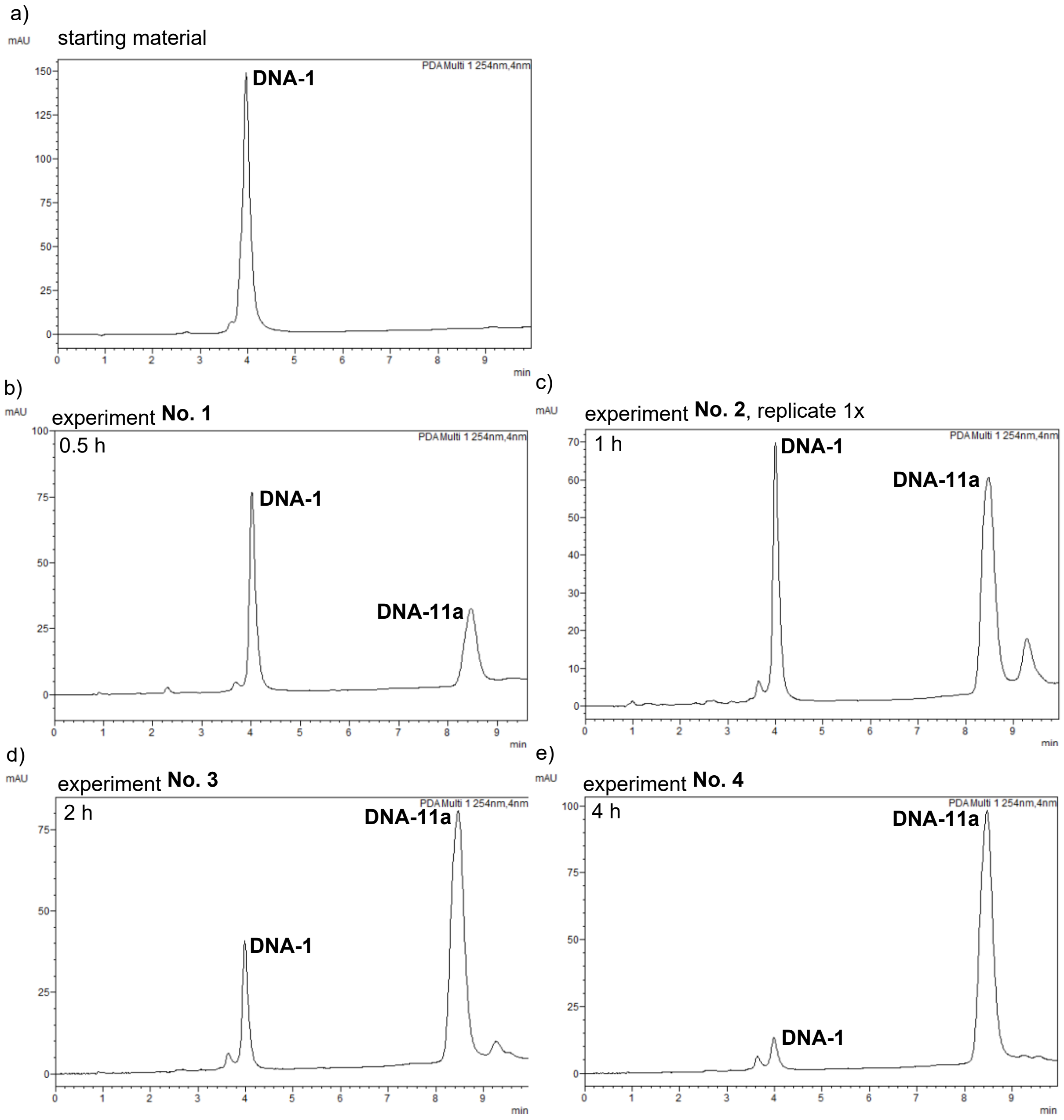

Figure S10. Kinetics of the copolymer **IA**-mediated Povarov reaction of the oligonucleotide-aldehyde conjugate **DNA-1**, 4-*tert*-butylaniline **9a**, and *N*-Boc-2,3-dihydro-1*H*-pyrrole **10a** at 25 °C, for reaction conditions see Table S8. HPLC traces show oligonucleotide-aldehyde conjugate **DNA-1** and experiments **No. 1** - **No. 4**.

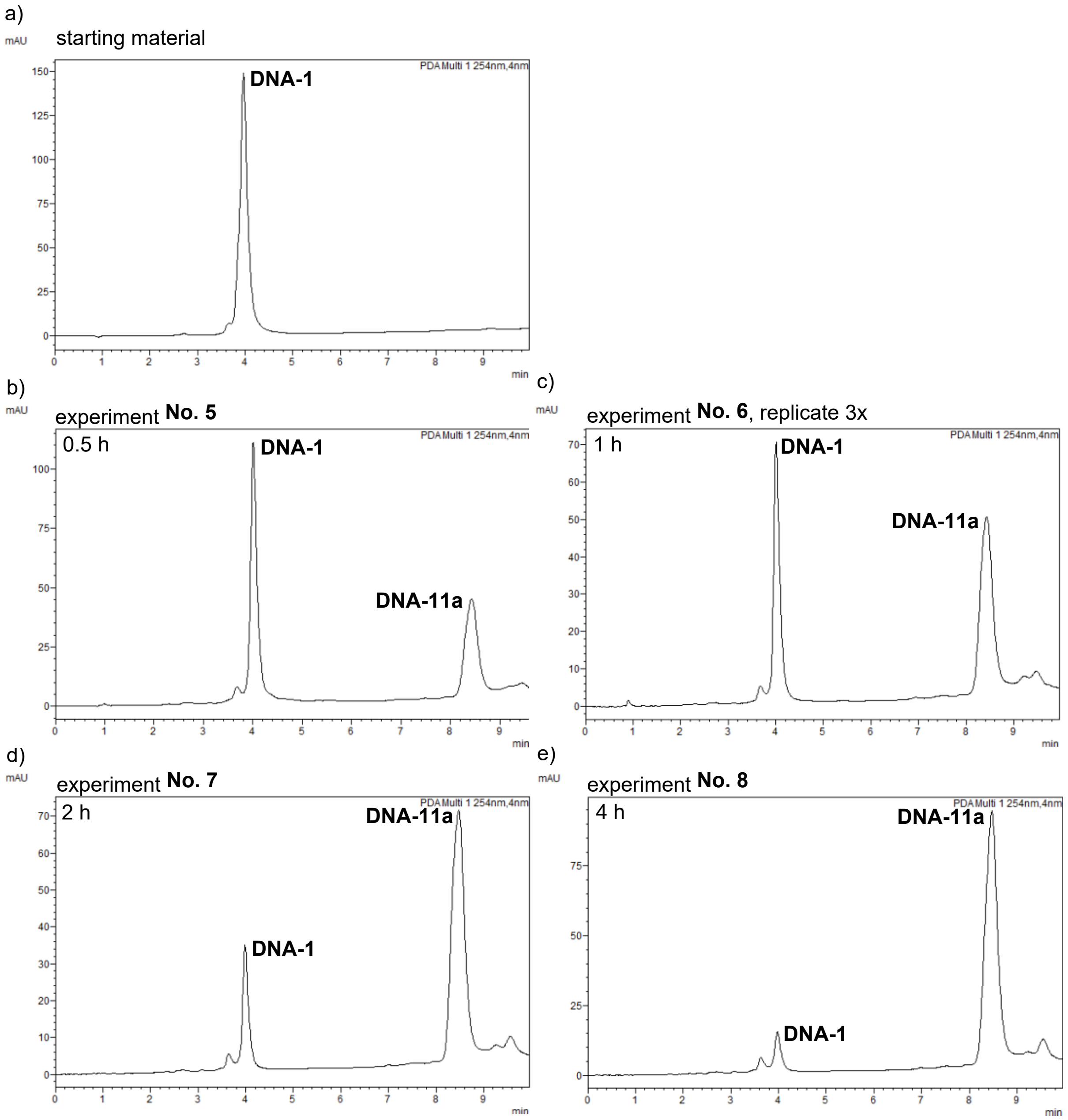

Figure S11. Kinetics of the copolymer **IB**-mediated Povarov reaction of the oligonucleotide-aldehyde conjugate **DNA-1**, 4-*tert*-butylaniline **9a**, and *N*-Boc-2,3-dihydro-1*H*-pyrrole **10a** at 25 °C, for reaction conditions see Table S8. HPLC traces show oligonucleotide-aldehyde conjugate **DNA-1** and experiments **No. 5** - **No. 8**.

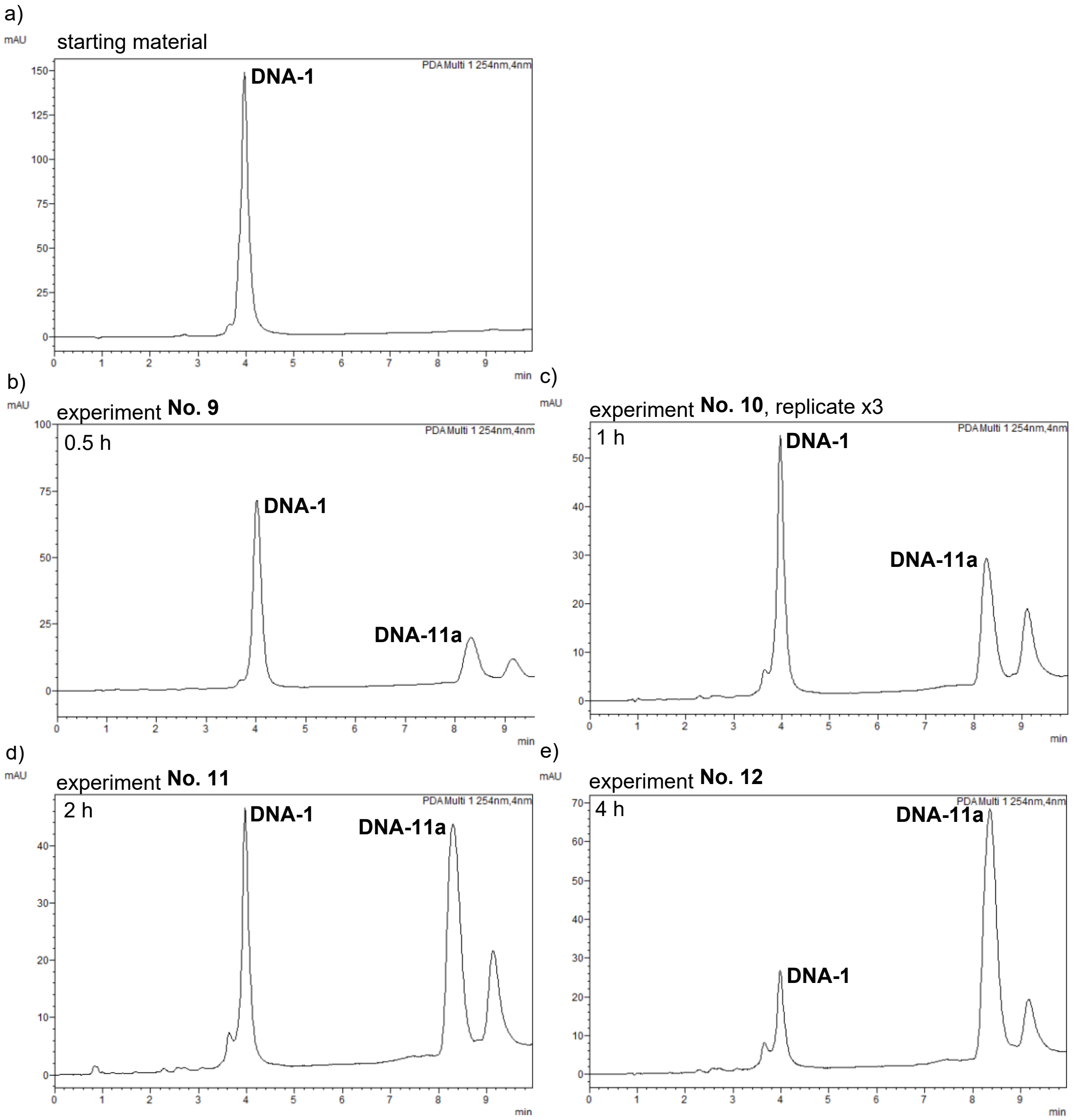

Figure S12. Kinetics of the copolymer **IC$_a$**-mediated Povarov reaction of the oligonucleotide-aldehyde conjugate **DNA-1**, 4-*tert*-butylaniline **9a**, and *N*-Boc-2,3-dihydro-1*H*-pyrrole **10a** at 25 °C, for reaction conditions see Table S8. HPLC traces show oligonucleotide-aldehyde conjugate **DNA-1** and experiments **No. 9** - **No. 12**.

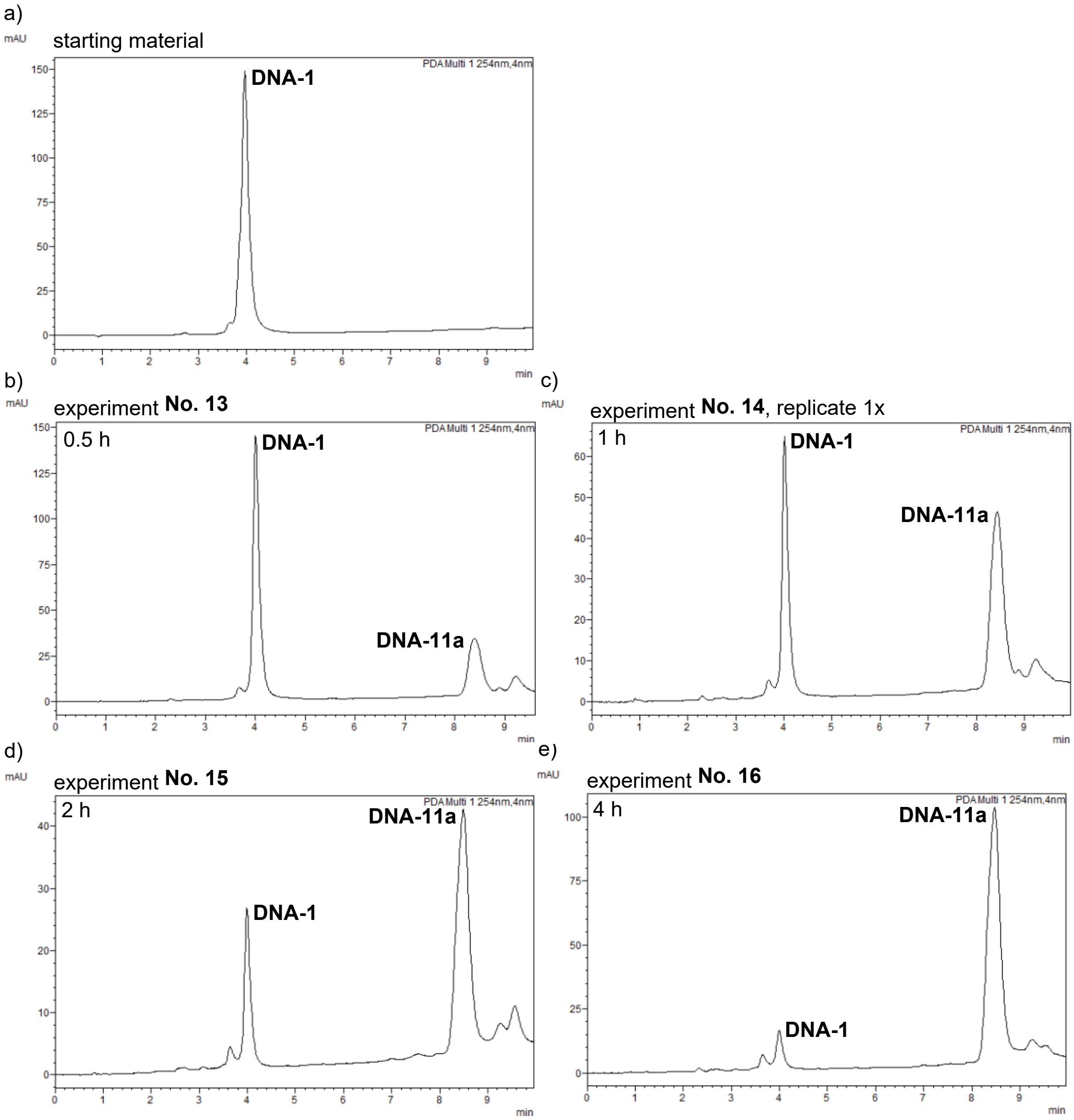

Figure S13. Kinetics of the copolymer **IC$_b$**-mediated Povarov reaction of the oligonucleotide-aldehyde conjugate **DNA-1**, 4-*tert*-butylaniline **9a**, and *N*-Boc-2,3-dihydro-1*H*-pyrrole **10a** at 25 °C, for reaction conditions see Table S8. HPLC traces show oligonucleotide-aldehyde conjugate **DNA-1** and experiments **No. 13** - **No. 16**.

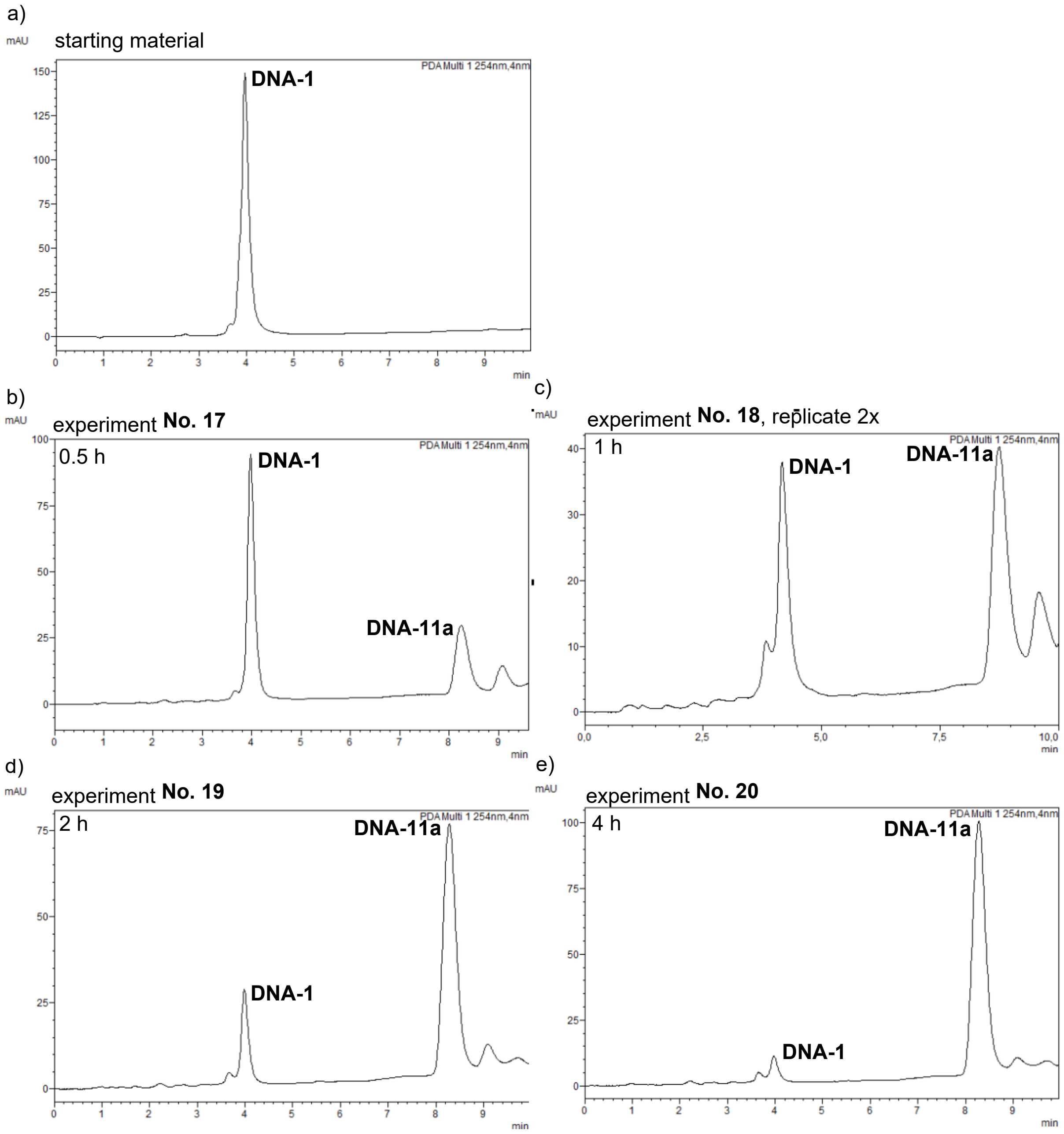

Figure S14. Kinetics of the copolymer **IC$_c$**-mediated Povarov reaction of the oligonucleotide-aldehyde conjugate **DNA-1**, 4-*tert*-butylaniline **9a**, and *N*-Boc-2,3-dihydro-1*H*-pyrrole **10a** at 25 °C, for reaction conditions see Table S8. HPLC traces show oligonucleotide-aldehyde conjugate **DNA-1** and experiments **No. 17** - **No. 20**.

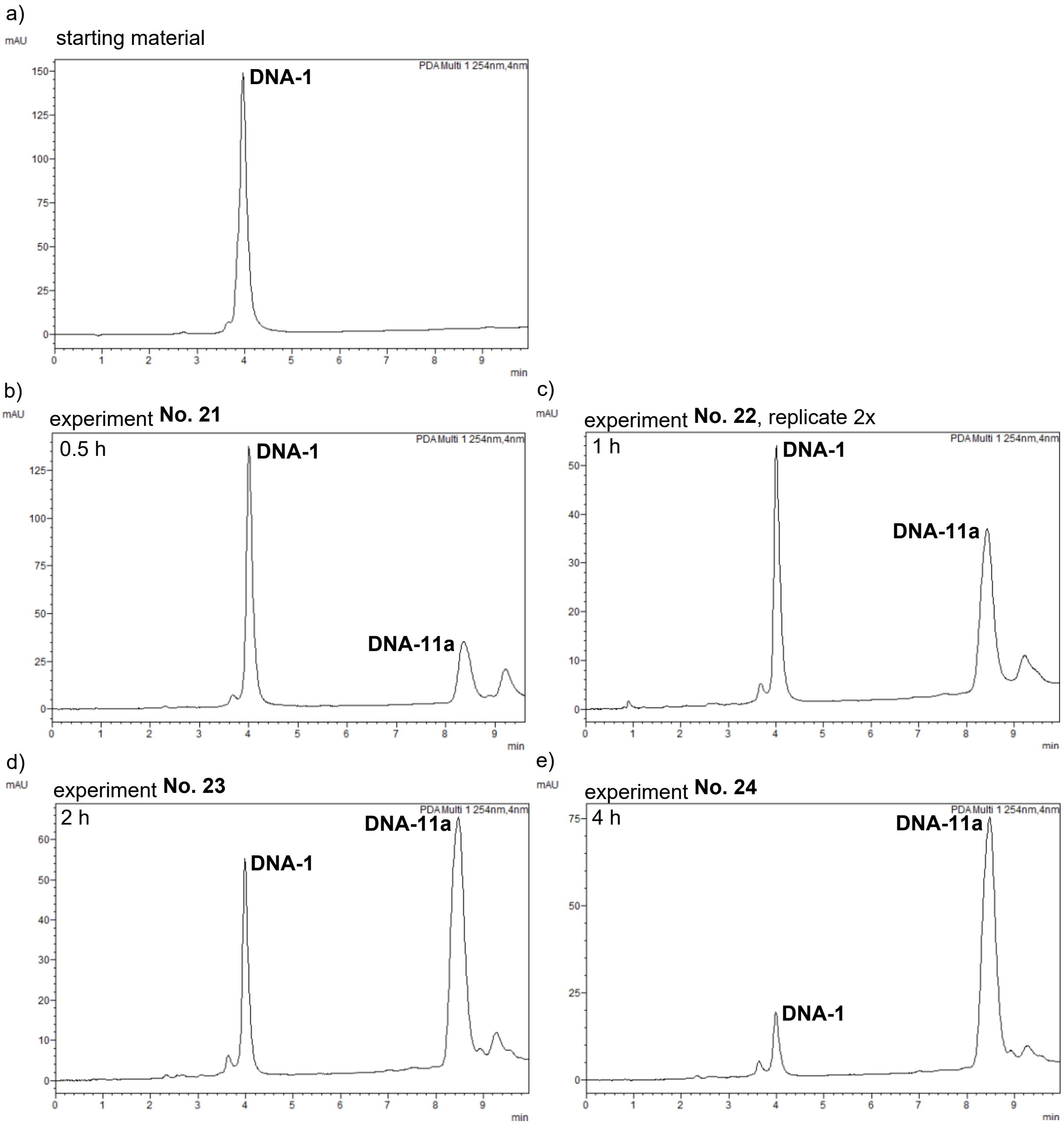

Figure S15. Kinetics of the copolymer **ID**-mediated Povarov reaction of the oligonucleotide-aldehyde conjugate **DNA-1**, 4-*tert*-butylaniline **9a**, and *N*-Boc-2,3-dihydro-1*H*-pyrrole **10a** at 25 °C, for reaction conditions see Table S8. HPLC traces show oligonucleotide-aldehyde conjugate **DNA-1** and experiments **No. 21** - **No. 24**.

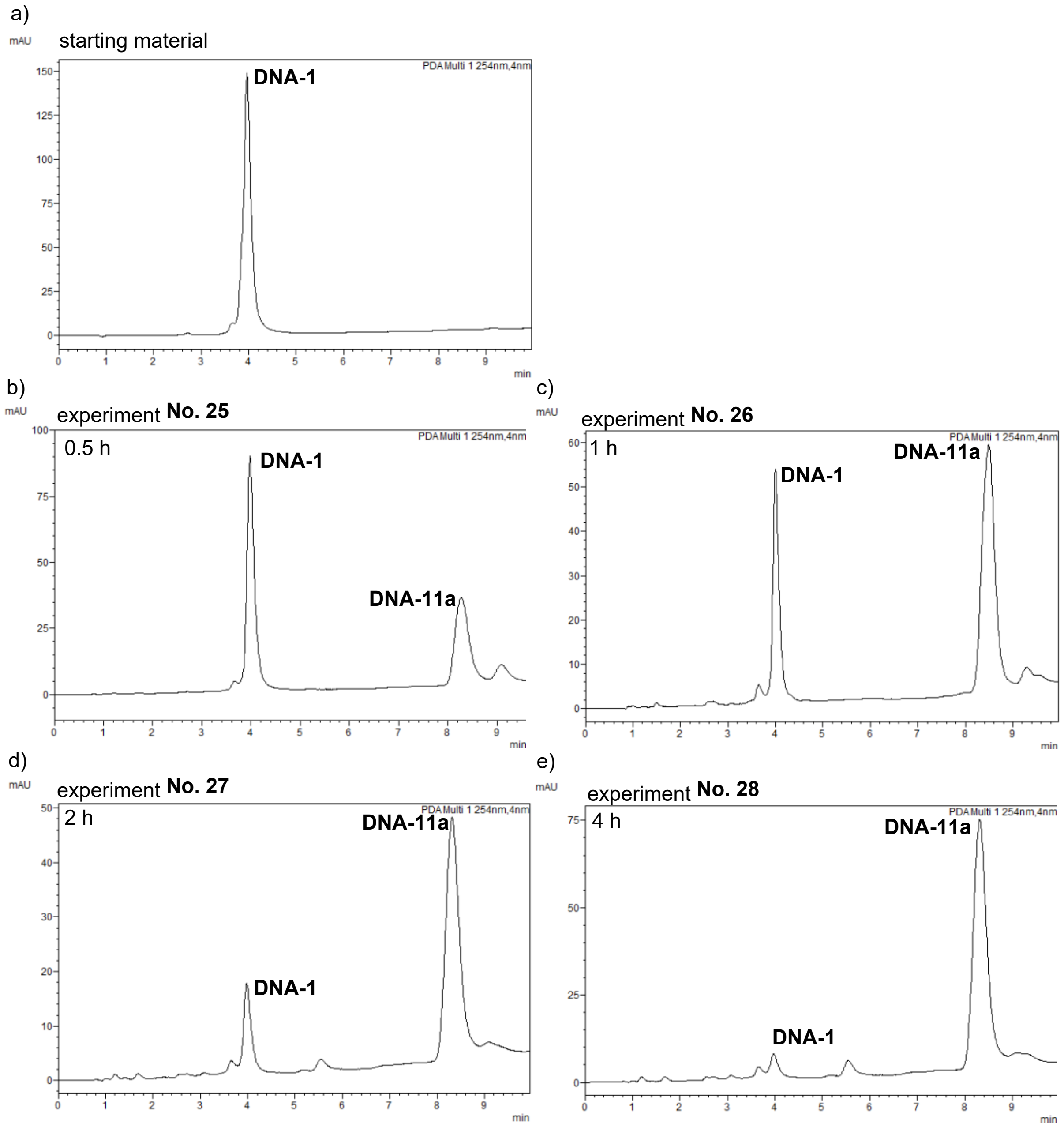

Figure S16. Kinetics of the copolymer **IIA**-mediated Povarov reaction of the oligonucleotide-aldehyde conjugate **DNA-1**, 4-*tert*-butylaniline **9a**, and *N*-Boc-2,3-dihydro-1*H*-pyrrole **10a** at 25 °C, for reaction conditions see Table S8. HPLC traces show oligonucleotide-aldehyde conjugate **DNA-1** and experiments **No. 25** - **No. 28**.

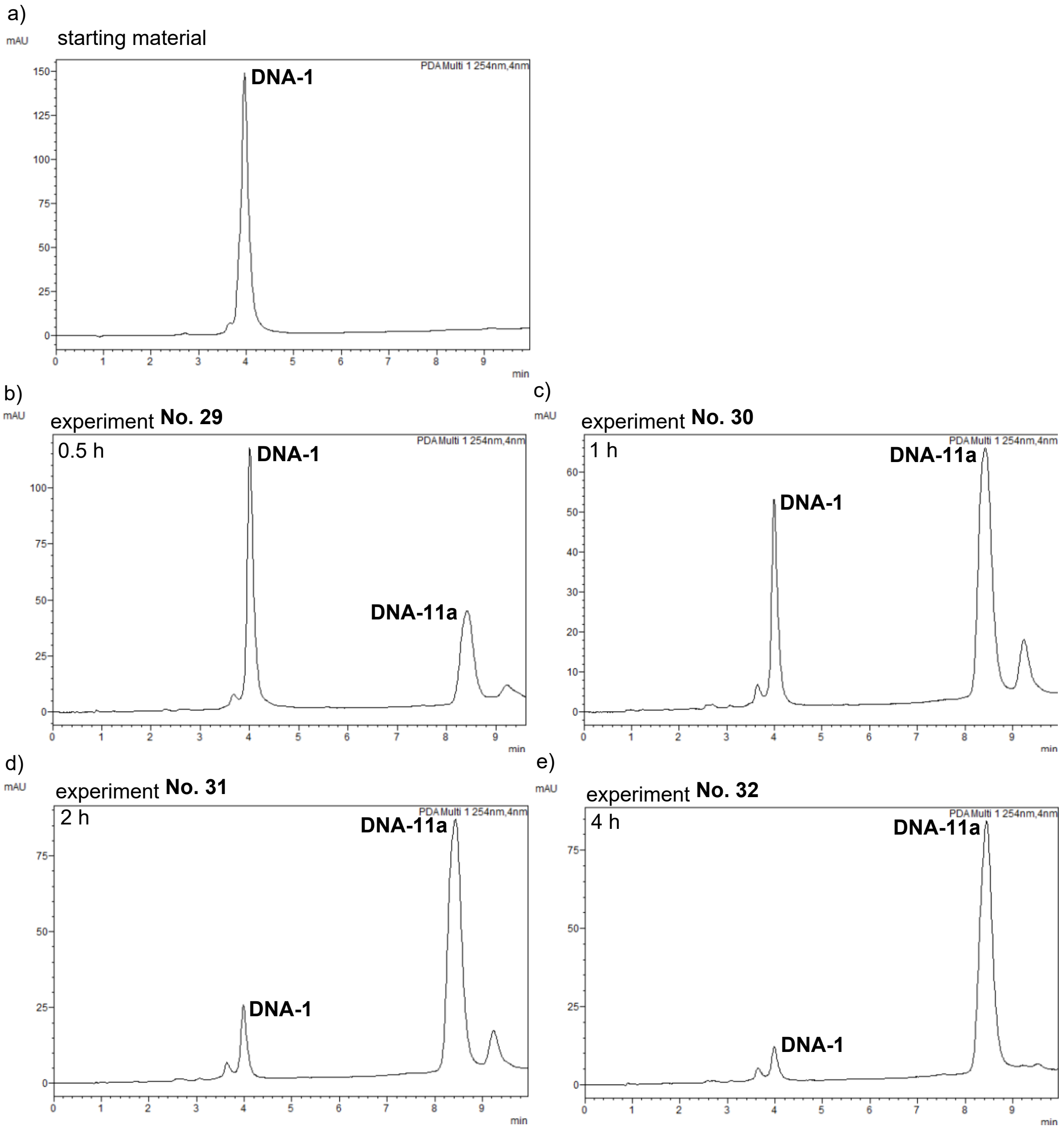

Figure S17. Kinetics of the copolymer **IIB**-mediated Povarov reaction of the oligonucleotide-aldehyde conjugate **DNA-1**, 4-*tert*-butylaniline **9a**, and *N*-Boc-2,3-dihydro-1*H*-pyrrole **10a** at 25 °C, for reaction conditions see Table S8. HPLC traces show oligonucleotide-aldehyde conjugate **DNA-1** and experiments **No. 29**- **No. 32**.

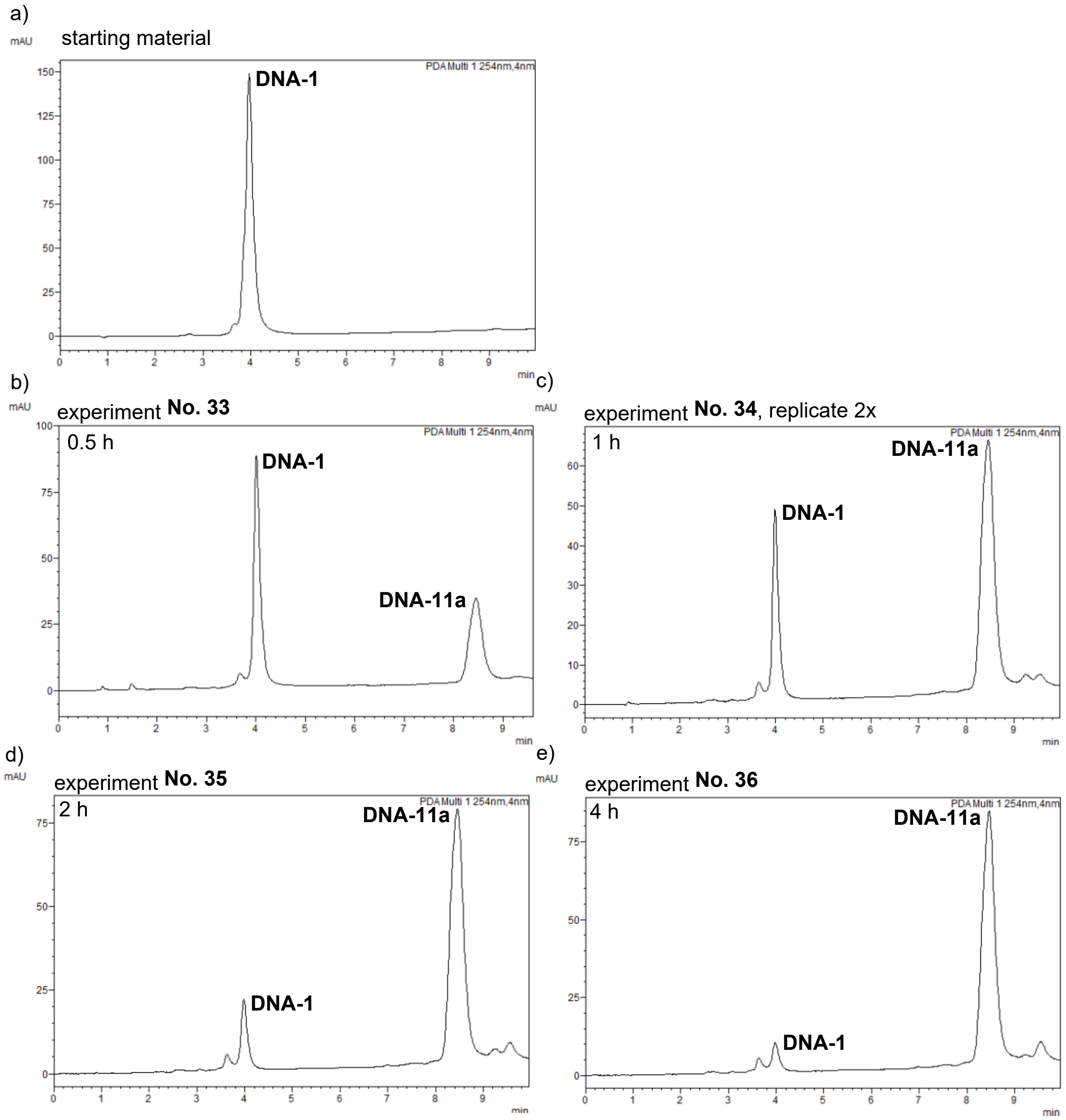

Figure S18. Kinetics of the copolymer **IIC$_a$**-mediated Povarov reaction of the oligonucleotide-aldehyde conjugate **DNA-1**, 4-*tert*-butylaniline **9a**, and *N*-Boc-2,3-dihydro-1*H*-pyrrole **10a** at 25 °C, for reaction conditions see Table S8. HPLC traces show oligonucleotide-aldehyde conjugate **DNA-1** and experiments **No. 33** - **No. 36**.

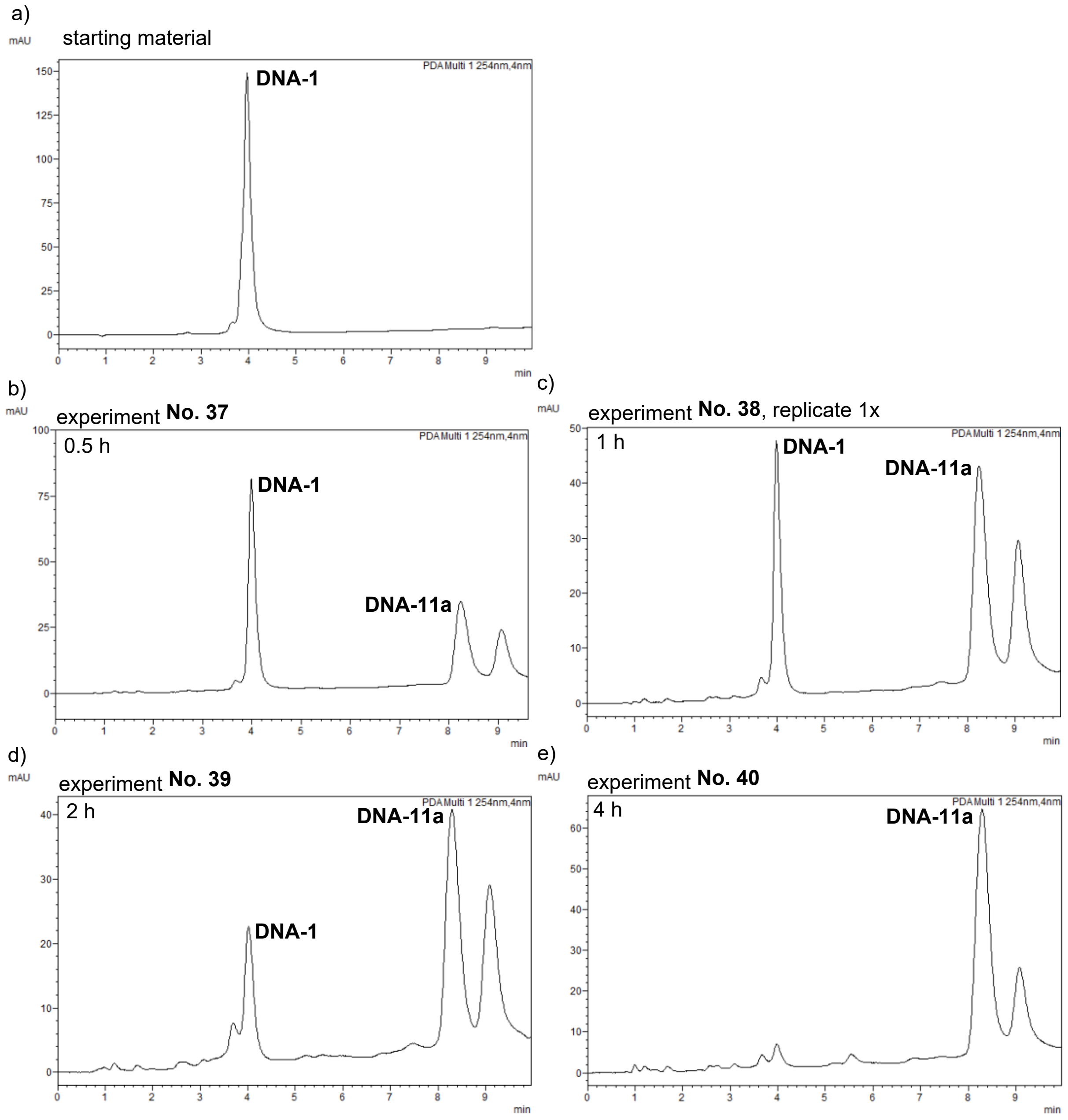

Figure S19. Kinetics of the copolymer **IIC$_b$**-mediated Povarov reaction of the oligonucleotide-aldehyde conjugate **DNA-1**, 4-*tert*-butylaniline **9a**, and *N*-Boc-2,3-dihydro-1*H*-pyrrole **10a** at 25 °C, for reaction conditions see Table S8. HPLC traces show oligonucleotide-aldehyde conjugate **DNA-1** and experiments **No. 37** - **No. 40**.

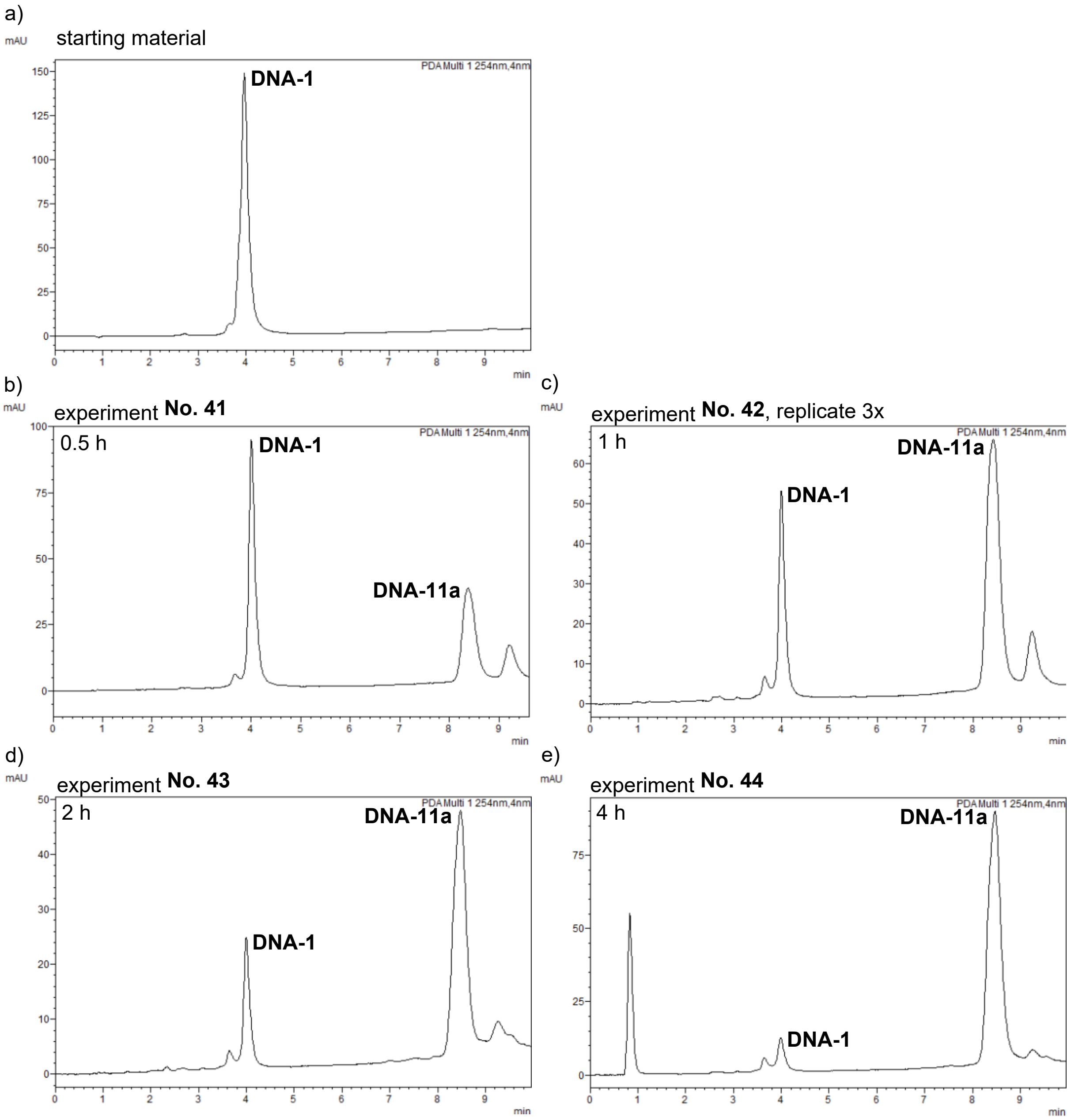

Figure S20. Kinetics of the copolymer **IID**-mediated Povarov reaction of the oligonucleotide-aldehyde conjugate **DNA-1**, 4-*tert*-butylaniline **9a**, and *N*-Boc-2,3-dihydro-1*H*-pyrrole **10a** at 25 °C, for reaction conditions see Table S8. HPLC traces show oligonucleotide-aldehyde conjugate **DNA-1** and experiments **No. 41** - **No. 44**.

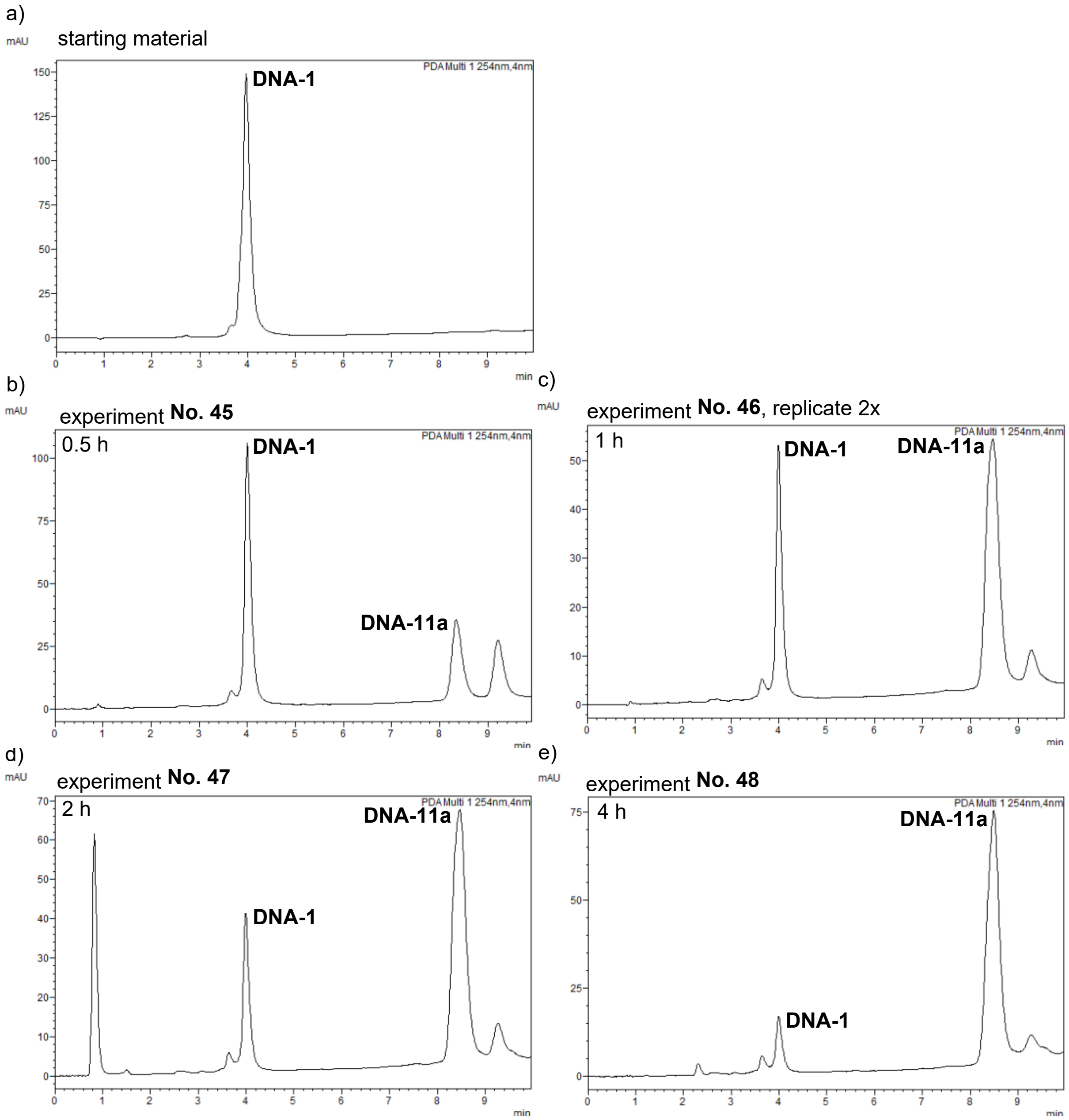

Figure S21. Kinetics of the copolymer **IIE**-mediated Povarov reaction of the oligonucleotide-aldehyde conjugate **DNA-1**, 4-*tert*-butylaniline **9a**, and *N*-Boc-2,3-dihydro-1*H*-pyrrole **10a** at 25 °C, for reaction conditions see Table S8. HPLC traces show oligonucleotide-aldehyde conjugate **DNA-1** and experiments **No. 45** - **No. 48**.

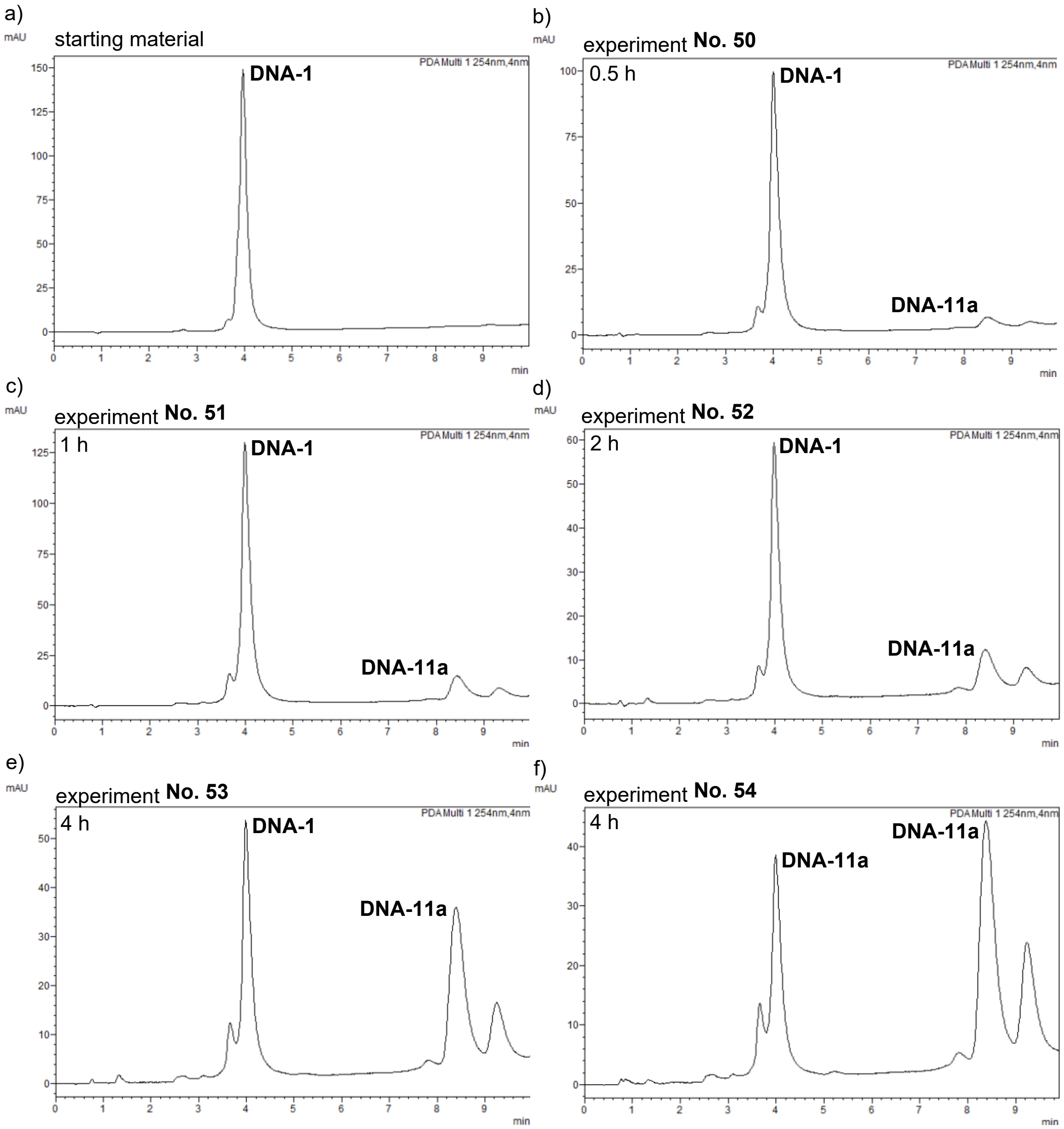

Figure S22. Kinetics of the Povarov reaction of the oligonucleotide-aldehyde conjugate **DNA-1**, 4-*tert*-butylaniline **9a**, and *N*-Boc-2,3-dihydro-1*H*-pyrrole **10a** at 25 °C, in the absence of the copolymer micelle, for reaction conditions see Table S8. HPLC traces show oligonucleotide-aldehyde conjugate **DNA-1** and experiments **No. 50** - **No. 54**.

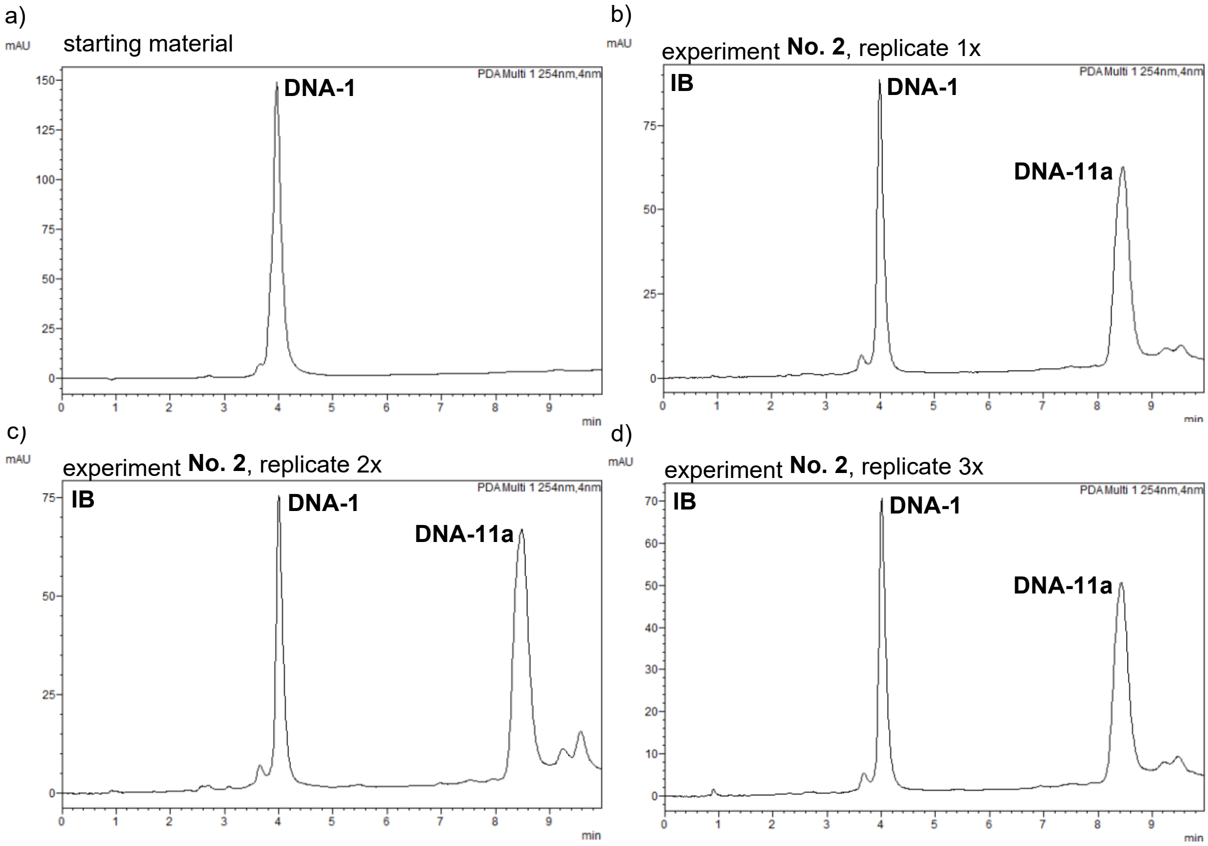

Figure S23. Copolymer **IB**-mediated Povarov reaction of the oligonucleotide-aldehyde conjugate **DNA-1**, aniline **9a**, and *N*-Boc-2,3-dihydro-1*H*-pyrrole **10a** at 25 °C, for reaction conditions see Table S10. HPLC traces show: a) oligonucleotide-aldehyde conjugate **DNA-1** and b)-c) experiment **No. 2** in triplicate.

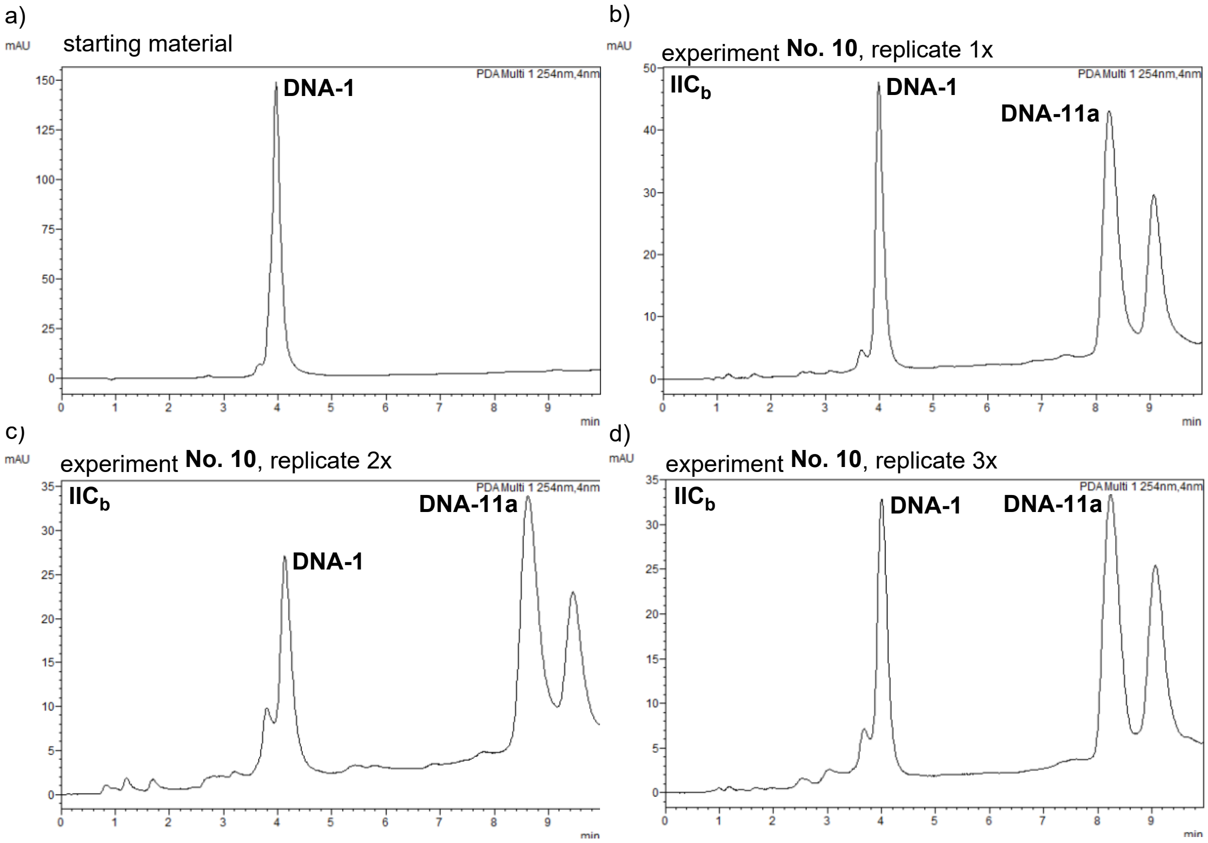

Figure S24. Copolymer **IIC$_b$**-mediated Povarov reaction of the oligonucleotide-aldehyde conjugate **DNA-1**, aniline **9a** and *N*-Boc-2,3-dihydro-1*H*-pyrrole **10a** at 25 °C, for reaction conditions see Table S10. HPLC traces show: a) oligonucleotide-aldehyde conjugate **DNA-1** and b)-c) experiment **No. 10** in triplicate.

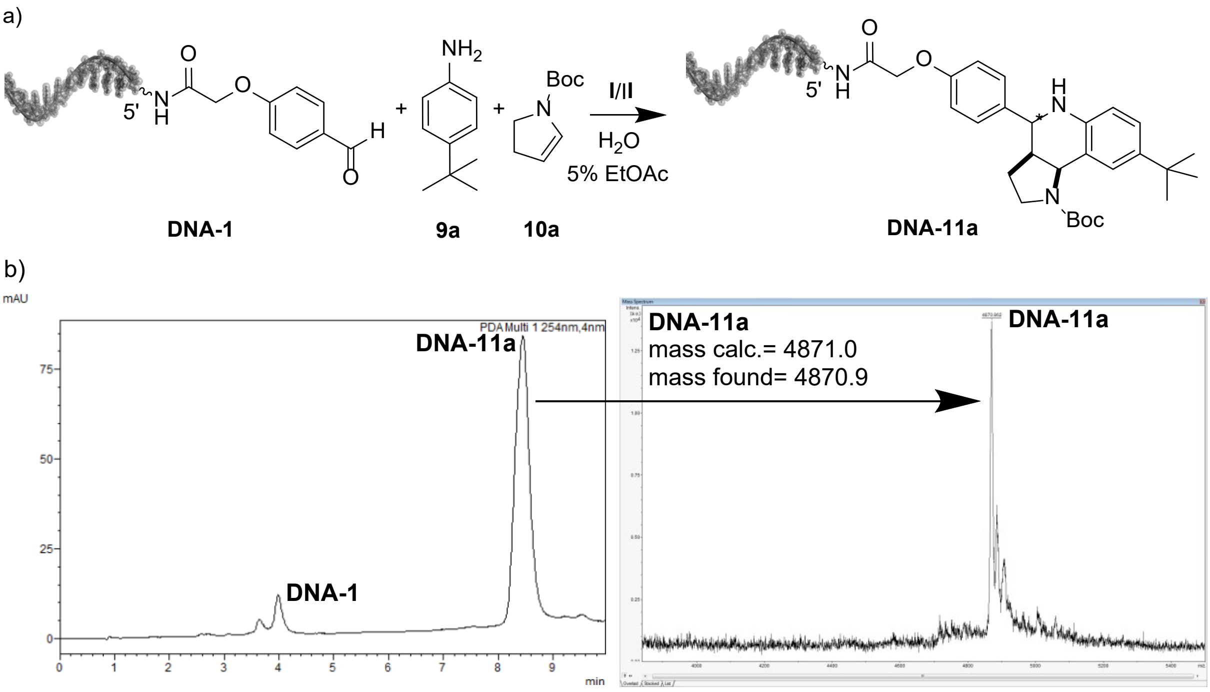

Figure S25. MALDI-MS analysis of the isolated product **DNA-11a**. a) Reaction scheme of the synthesis of **DNA-11a**; conditions: 50 eq. of copolymer **I/II** (0.5 mM), 2000 eq. of reactant **9a** and 2000 eq. of reactant **10a**, 25 °C, 18 h. b) HPLC trace of the crude reaction mixture (left hand) from Fig. S17e (Table S8, **No. 32**) and MALDI-MS spectrum of the isolated product **DNA-11a** (right hand).

4.4. Exploring sulfonic acid un-substituted copolymer micelle **III** for the synthesis of **DNA-11a**

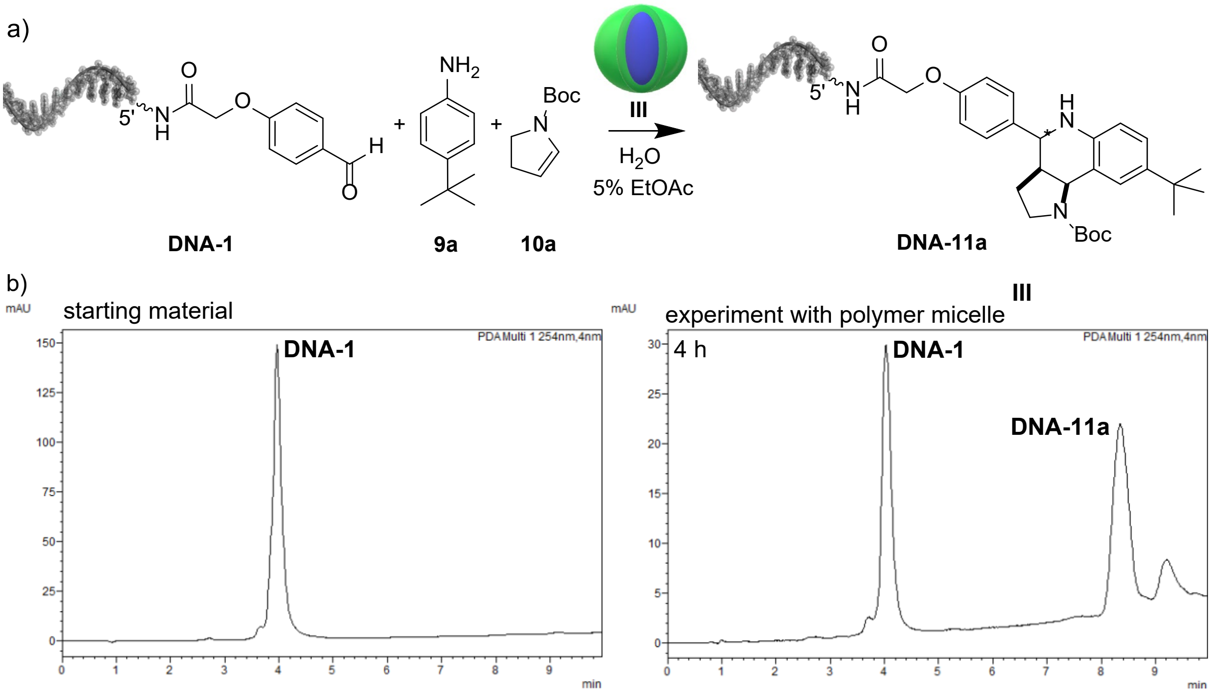

Figure S26. Exploring sulfonic acid un-substituted copolymer micelle **III** for the synthesis of **DNA-11a**. a) Reaction scheme of the synthesis of **DNA-11a**; conditions: 50 eq. of copolymer **III** (0.5 mM), 2000 eq. of reactant **9a** and 2000 eq. of **10a**, 25 °C, 4 h. b) HPLC traces show oligonucleotide-aldehyde conjugate **DNA-1** (left hand trace) and experiment **No. 49** (Table S8) with copolymer micelle **III** (right hand trace).

4.5. Kinetics of the copolymer micelle **I/II**-mediated Povarov reaction of aldehyde conjugate **DNA-1**, *tert*-butylaniline **9a**, and *N*-Boc-2,3-dihydro-1*H*-pyrrole **10a** to DNA-conjugate **DNA-11a** at elevated temperatures

Table S13. Impact of copolymer micelle **I/II** design on synthesis of DNA-hexahydro-1*H*-pyrrolo[3,2-c]quinoline conjugate **DNA-11a** at elevated temperatures. Reaction conditions: 2000 eq. of **9a** and 2000 eq. of **10a**, 50 eq. of **I/II**, 0.5 mM **I/II**, 1 h.

No.	copolymer name	copolymer composition	*T* [° C]	**DNA-11a** [%][a]
1[b]	**IB**	DMA_{62}-(EA_{23}-SPA_2)	25	58(7)
2	**IB**	DMA_{62}-(EA_{23}-SPA_2)	40	72(7)
3	**IB**	DMA_{62}-(EA_{23}-SPA_2)	50	77(6)
4	**IB**	DMA_{62}-(EA_{23}-SPA_2)	60	81(4)
5[b]	**IC$_a$**	DMA_{65}-(BA_{33}-SPA_2)	25	45(17)
6	**IC$_a$**	DMA_{65}-(BA_{33}-SPA_2)	40	43(21)
7	**IC$_a$**	DMA_{65}-(BA_{33}-SPA_2)	50	45(18)
8	**IC$_a$**	DMA_{65}-(BA_{33}-SPA_2)	60	48(14)
9[b]	**IC$_c$**	DMA_{132}-(BA_{23}-SPA_2)	25	48(17)
10	**IC$_c$**	DMA_{132}-(BA_{23}-SPA_2)	40	56(25)
11	**IC$_c$**	DMA_{132}-(BA_{23}-SPA_2)	50	71(8)
12	**IC$_c$**	DMA_{132}-(BA_{23}-SPA_2)	60	77(8)
13[b]	**ID**	DMA_{62}-(OA_{26}-SPA_2)	25	53(11)
14	**ID**	DMA_{62}-(OA_{26}-SPA_2)	40	67(10)
15	**ID**	DMA_{62}-(OA_{26}-SPA_2)	50	68(8)
16	**ID**	DMA_{62}-(OA_{26}-SPA_2)	60	71(7)
17[b]	**IIC$_a$**	(DMA_{63}-SPA_2)-BA_{33}	25	70(8)
18	**IIC$_a$**	(DMA_{63}-SPA_2)-BA_{33}	40	79(6)
19	**IIC$_a$**	(DMA_{63}-SPA_2)-BA_{33}	50	79(6)
20	**IIC$_a$**	(DMA_{63}-SPA_2)-BA_{33}	60	89
21[b]	**IIC$_b$**	(DMA_{100}-SPA_2)-BA_{34}	25	44(32)
22	**IIC$_b$**	(DMA_{100}-SPA_2)-BA_{34}	40	50(30)
23	**IIC$_b$**	(DMA_{100}-SPA_2)-BA_{34}	50	59(21)
24	**IIC$_b$**	(DMA_{100}-SPA_2)-BA_{34}	60	65(21)
25[b]	**IID**	(DMA_{62}-SPA_2)-OA_{26}	25	63(13)

26	**IID**	(DMA_{62}-SPA_2)-OA_{26}	40	76(6)
27	**IID**	(DMA_{62}-SPA_2)-OA_{26}	50	78(6)
28	**IID**	(DMA_{62}-SPA_2)-OA_{26}	60	86
29	**IIE**[b]	(DMA_{62}-SPA_2)-DDA_{12}	25	62(8)
30	**IIE**	(DMA_{62}-SPA_2)-DDA_{12}	40	74(5)
31	**IIE**	(DMA_{62}-SPA_2)-DDA_{12}	50	76(4)
32	**IIE**	(DMA_{62}-SPA_2)-DDA_{12}	60	84
33	**III**	DMA_{72}-BA_{13}	25	13(7)
34	**III**	DMA_{72}-BA_{13}	40	21(8)
35	**III**	DMA_{72}-BA_{13}	50	32(5)
36	**III**	DMA_{72}-BA_{13}	60	34
37	-	-	25	16(8)
38	-	-	40	20(8)
39	-	-	50	29(8)
40	-	-	60	36

[a] HPLC analysis of the crude, missing percentage to 100%: mainly **DNA-1**; [b] experiments were set up in triplicates, see Table S10. Conversions in parentheses show those of a later eluting side product.

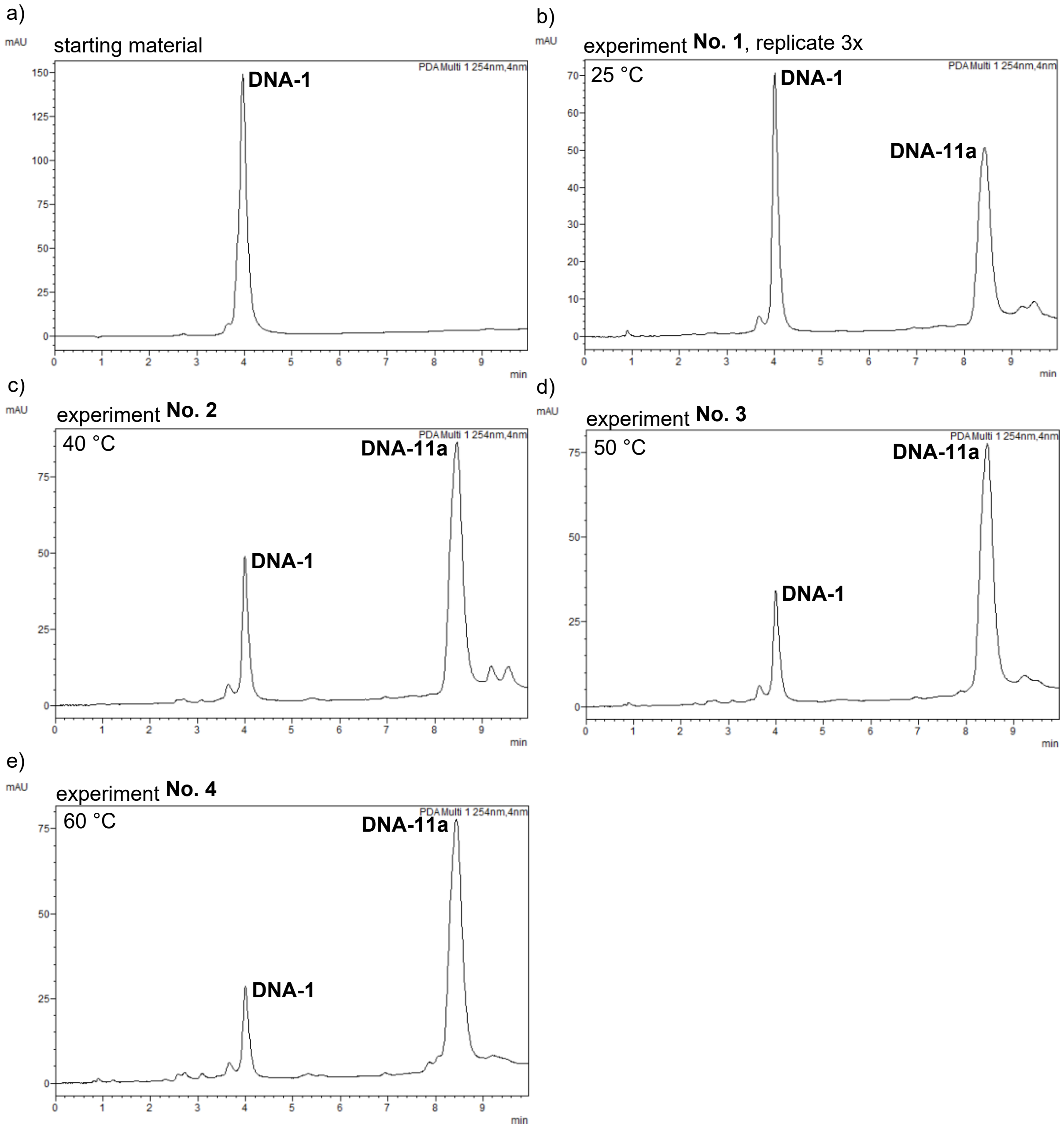

Figure S27. Effect of temperature on the copolymer **IB**-mediated Povarov reaction of the oligonucleotide-aldehyde conjugate **DNA-1**, 4-*tert*-butylaniline **9a**, and *N*-Boc-2,3-dihydro-1*H*-pyrrole **10a** after 1 hour, for reaction conditions see Table S13. HPLC traces show oligonucleotide-aldehyde conjugate **DNA-1** and experiments **No. 1** - **No. 4**.

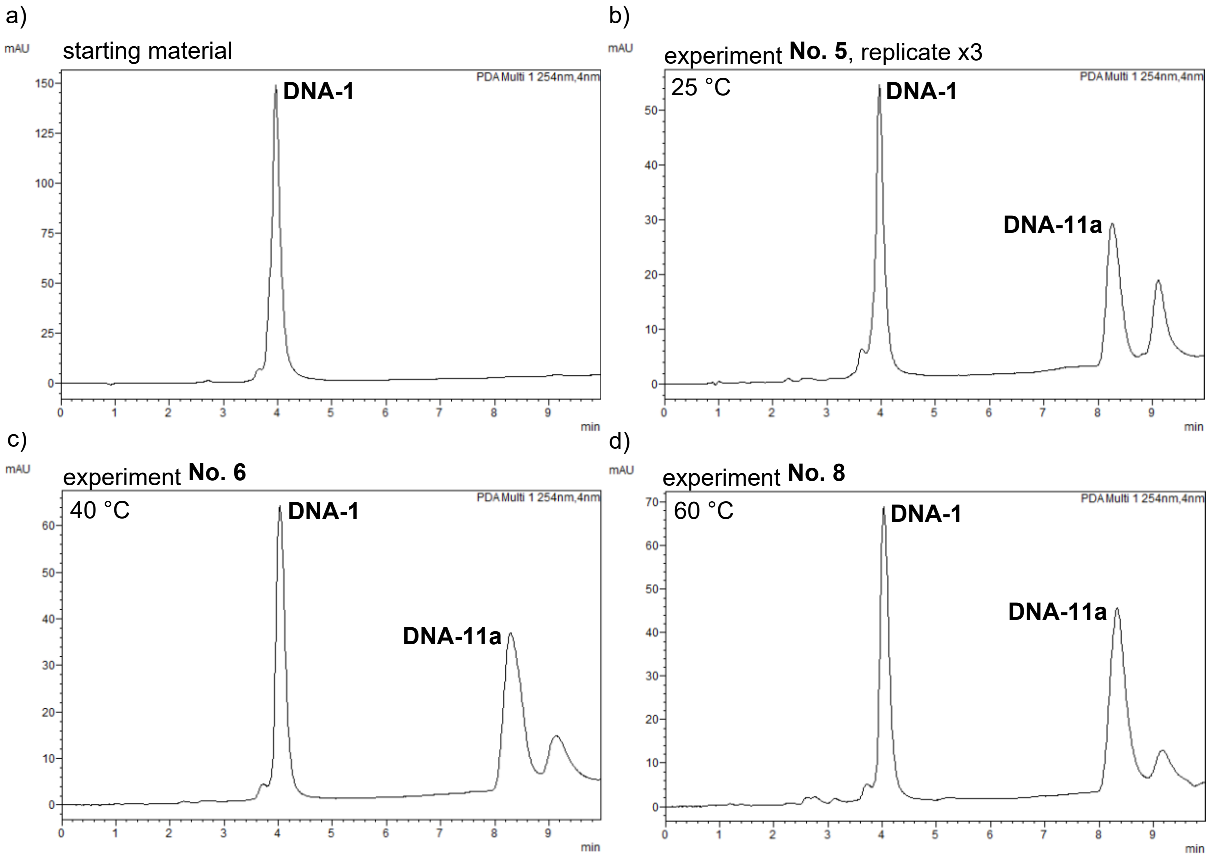

Figure S28. Effect of temperature on the copolymer **IC$_a$**-mediated Povarov reaction of the oligonucleotide-aldehyde conjugate **DNA-1**, 4-*tert*-butylaniline **9a**, and *N*-Boc-2,3-dihydro-1*H*-pyrrole **10a** after 1 hour, for reaction conditions see Table S13. HPLC traces show oligonucleotide-aldehyde conjugate **DNA-1** and experiments **No. 5**, **No. 6** and **No. 8**.

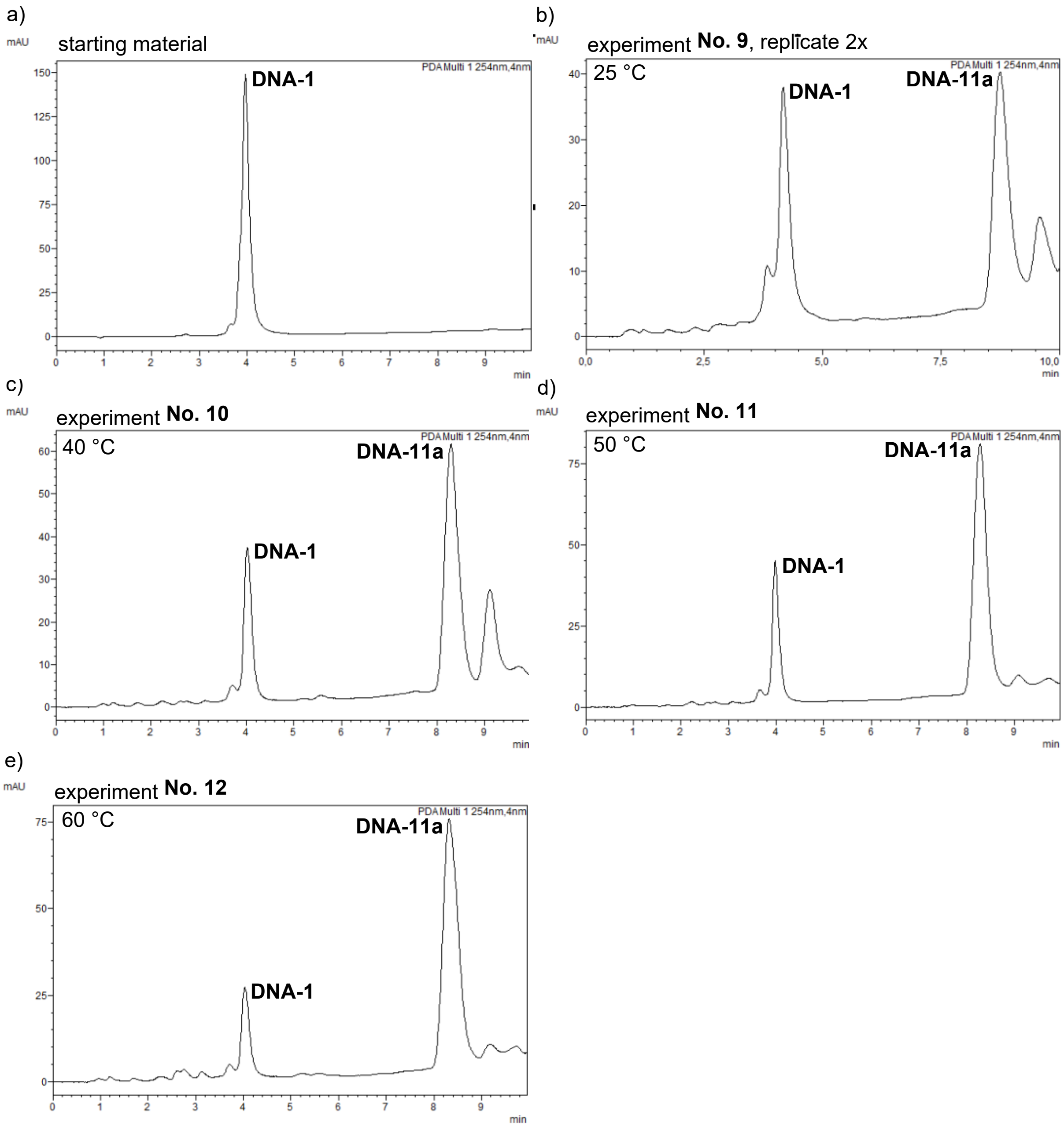

Figure S29. Effect of temperature on the copolymer **IC$_c$**-mediated Povarov reaction of the oligonucleotide-aldehyde conjugate **DNA-1**, 4-*tert*-butylaniline **9a**, and *N*-Boc-2,3-dihydro-1*H*-pyrrole **10a** after 1 hour, for reaction conditions see Table S13. HPLC traces show oligonucleotide-aldehyde conjugate **DNA-1** and experiments **No. 9** - **No. 12**.

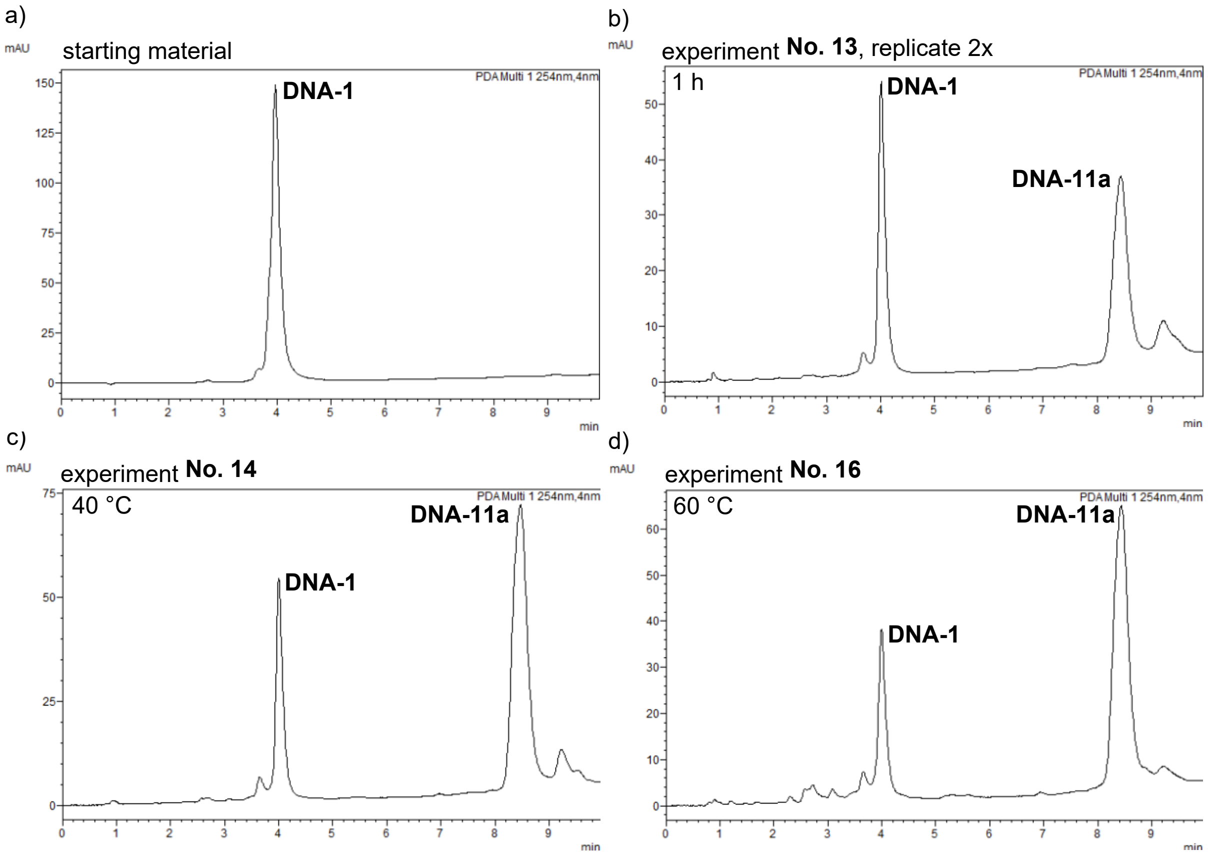

Figure S30. Effect of temperature on the copolymer **ID**-mediated Povarov reaction of the oligonucleotide-aldehyde conjugate **DNA-1**, 4-*tert*-butylaniline **9a**, and *N*-Boc-2,3-dihydro-1*H*-pyrrole **10a** after 1 hour, for reaction conditions see Table S13. HPLC traces show oligonucleotide-aldehyde conjugate **DNA-1** and experiments **No. 13, No. 14** and **No. 16**.

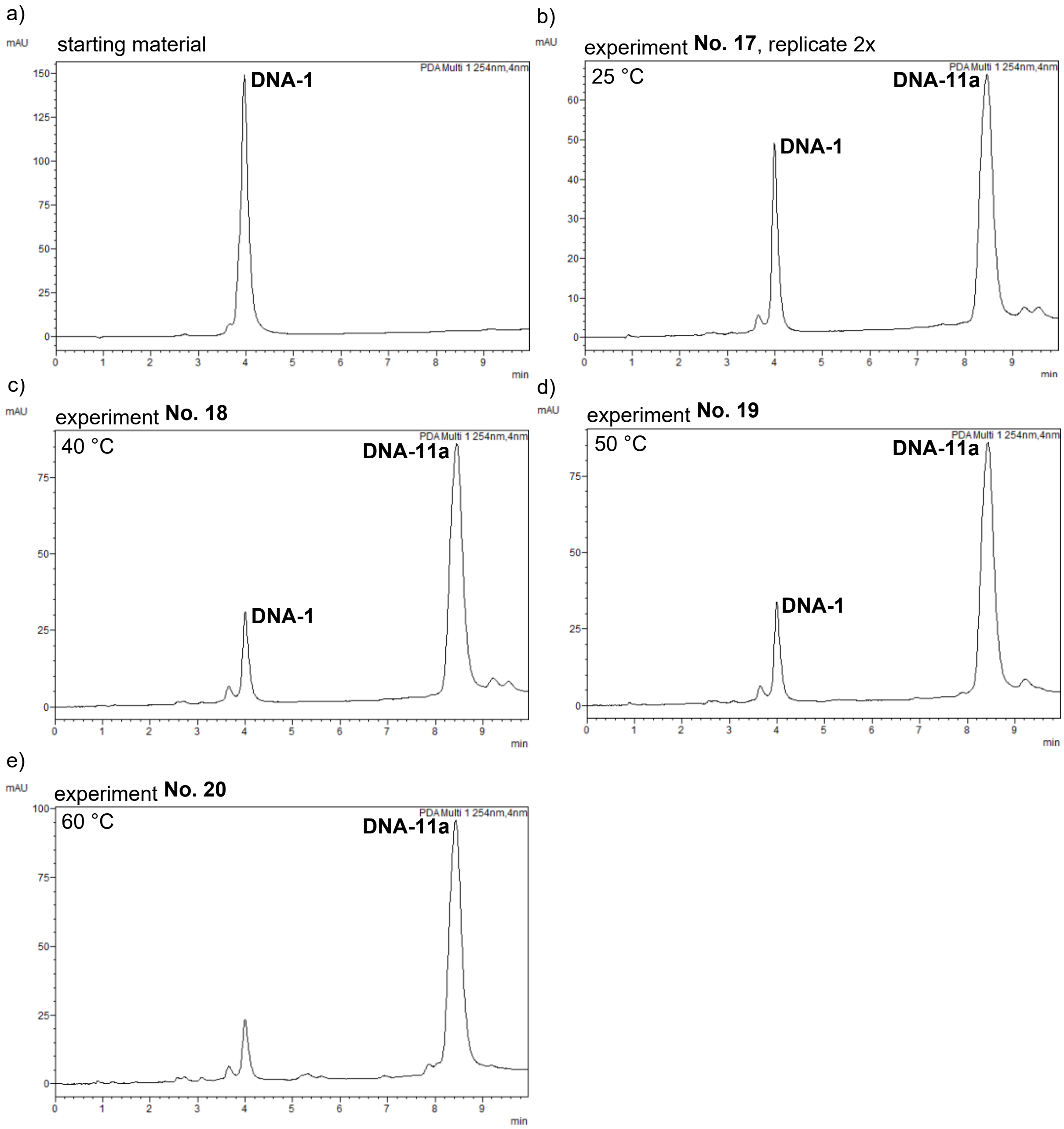

Figure S31. Effect of temperature on the copolymer **IIC$_a$**-mediated Povarov reaction of the oligonucleotide-aldehyde conjugate **DNA-1**, 4-*tert*-butylaniline **9a**, and *N*-Boc-2,3-dihydro-1*H*-pyrrole **10a** after 1 hour, for reaction conditions see Table S13. HPLC traces show oligonucleotide-aldehyde conjugate **DNA-1** and experiments **No. 17** - **No. 20**.

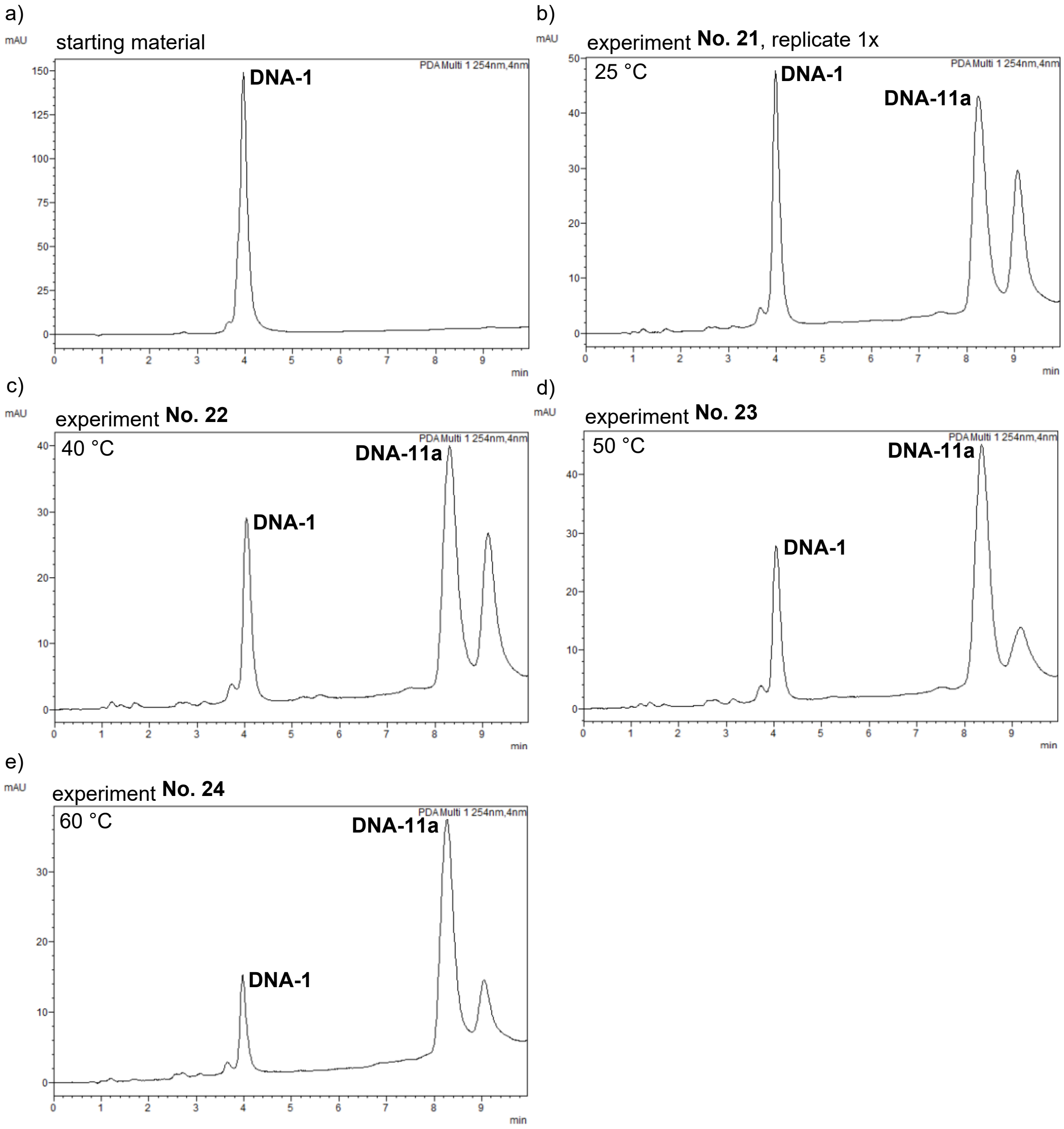

Figure S32. Effect of temperature on the copolymer **IIC**$_{\mathbf{b}}$-mediated Povarov reaction of the oligonucleotide-aldehyde conjugate **DNA-1**, 4-*tert*-butylaniline **9a**, and *N*-Boc-2,3-dihydro-1*H*-pyrrole **10a** after 1 hour, for reaction conditions see Table S13. HPLC traces show oligonucleotide-aldehyde conjugate **DNA-1** and experiments **No. 21** - **No. 24**.

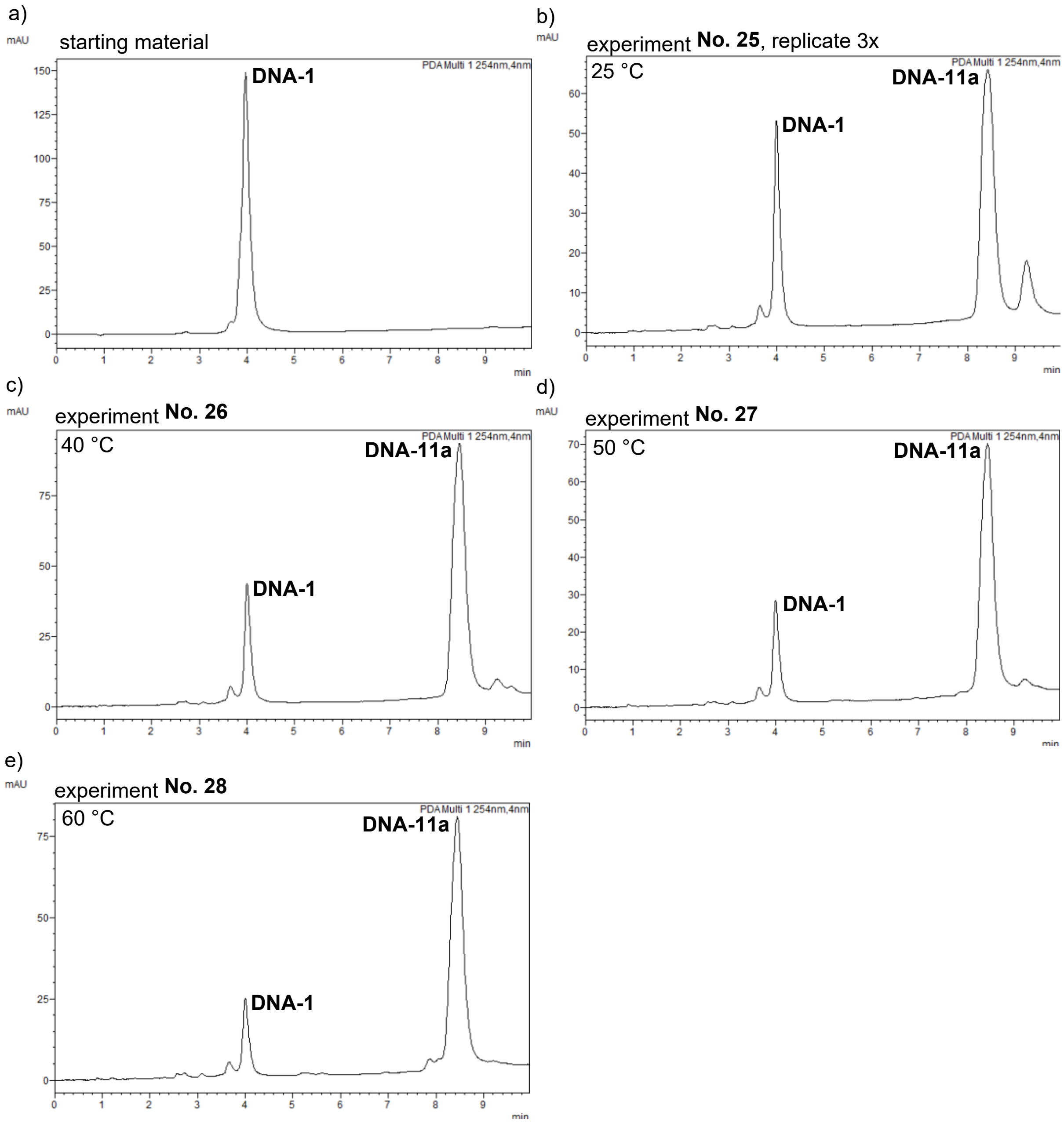

Figure S33. Effect of temperature on the copolymer **IID**-mediated Povarov reaction of the oligonucleotide-aldehyde conjugate **DNA-1**, 4-*tert*-butylaniline **9a**, and *N*-Boc-2,3-dihydro-1*H*-pyrrole **10a** after 1 hour, for reaction conditions see Table S13. HPLC traces show oligonucleotide-aldehyde conjugate **DNA-1** and experiments **No. 25** - **No. 28**.

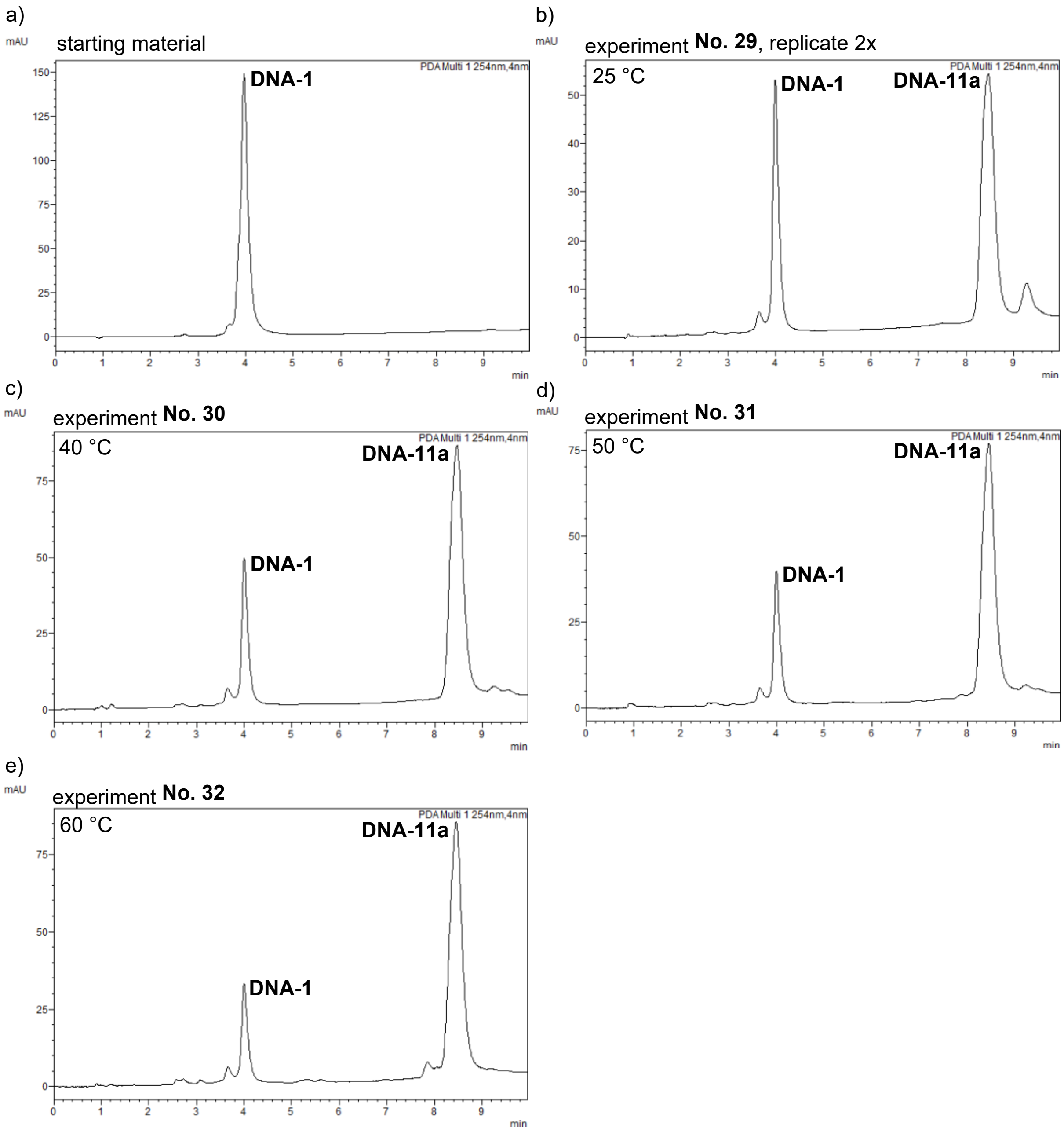

Figure S34. Effect of temperature on the copolymer **IIE**-mediated Povarov reaction of the oligonucleotide-aldehyde conjugate **DNA-1**, 4-*tert*-butylaniline **9a**, and *N*-Boc-2,3-dihydro-1*H*-pyrrole **10a** after 1 hour, for reaction conditions see Table S13. HPLC traces show oligonucleotide-aldehyde conjugate **DNA-1** and experiments **No. 29** - **No. 32**.

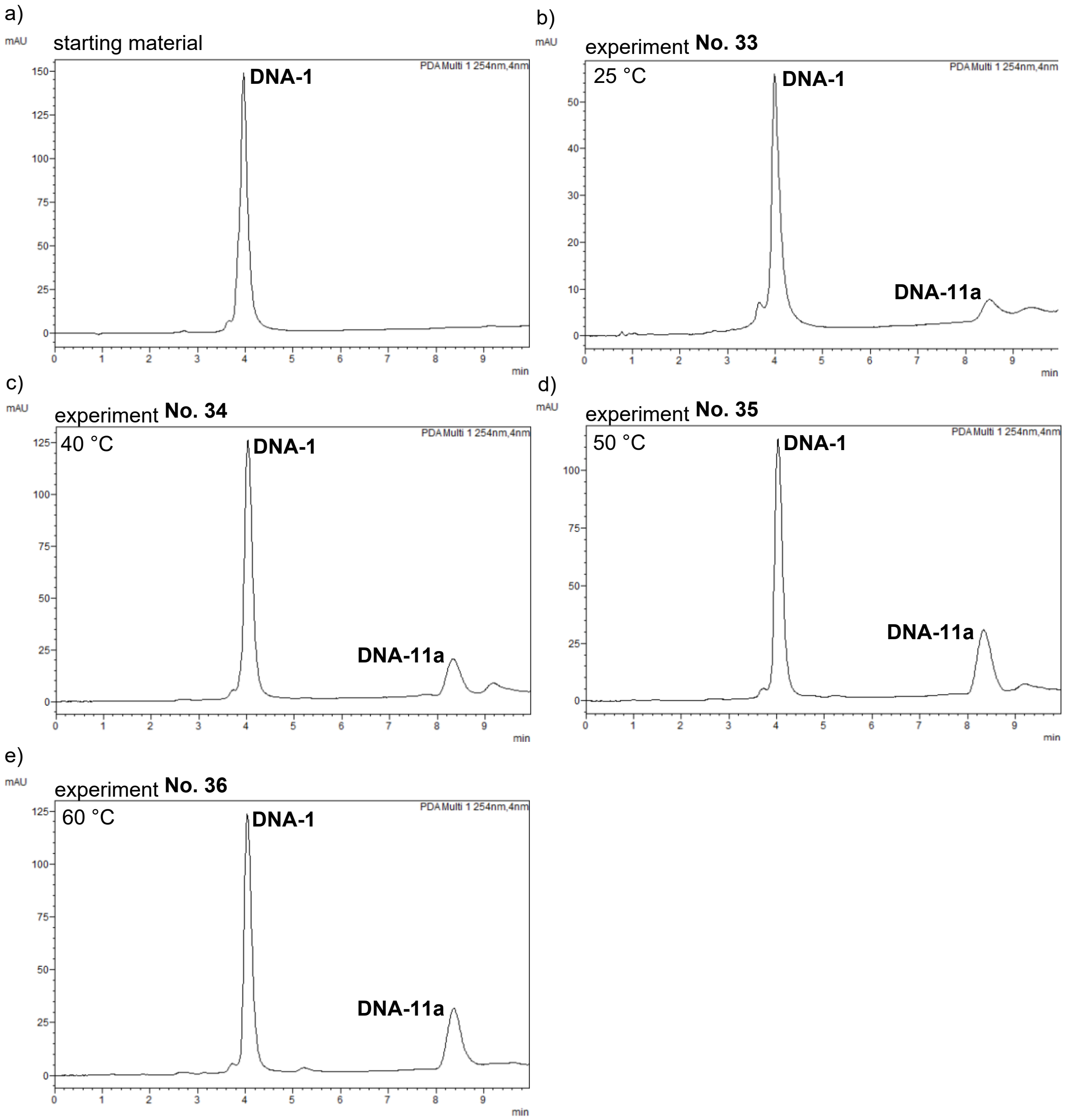

Figure S35. Effect of temperature on the copolymer **III**-mediated Povarov reaction of the oligonucleotide-aldehyde conjugate **DNA-1**, 4-*tert*-butylaniline **9a**, and *N*-Boc-2,3-dihydro-1*H*-pyrrole **10a** after 1 hour, for reaction conditions see Table S13. HPLC traces show oligonucleotide-aldehyde conjugate **DNA-1** and experiments **No. 33** - **No. 36**.

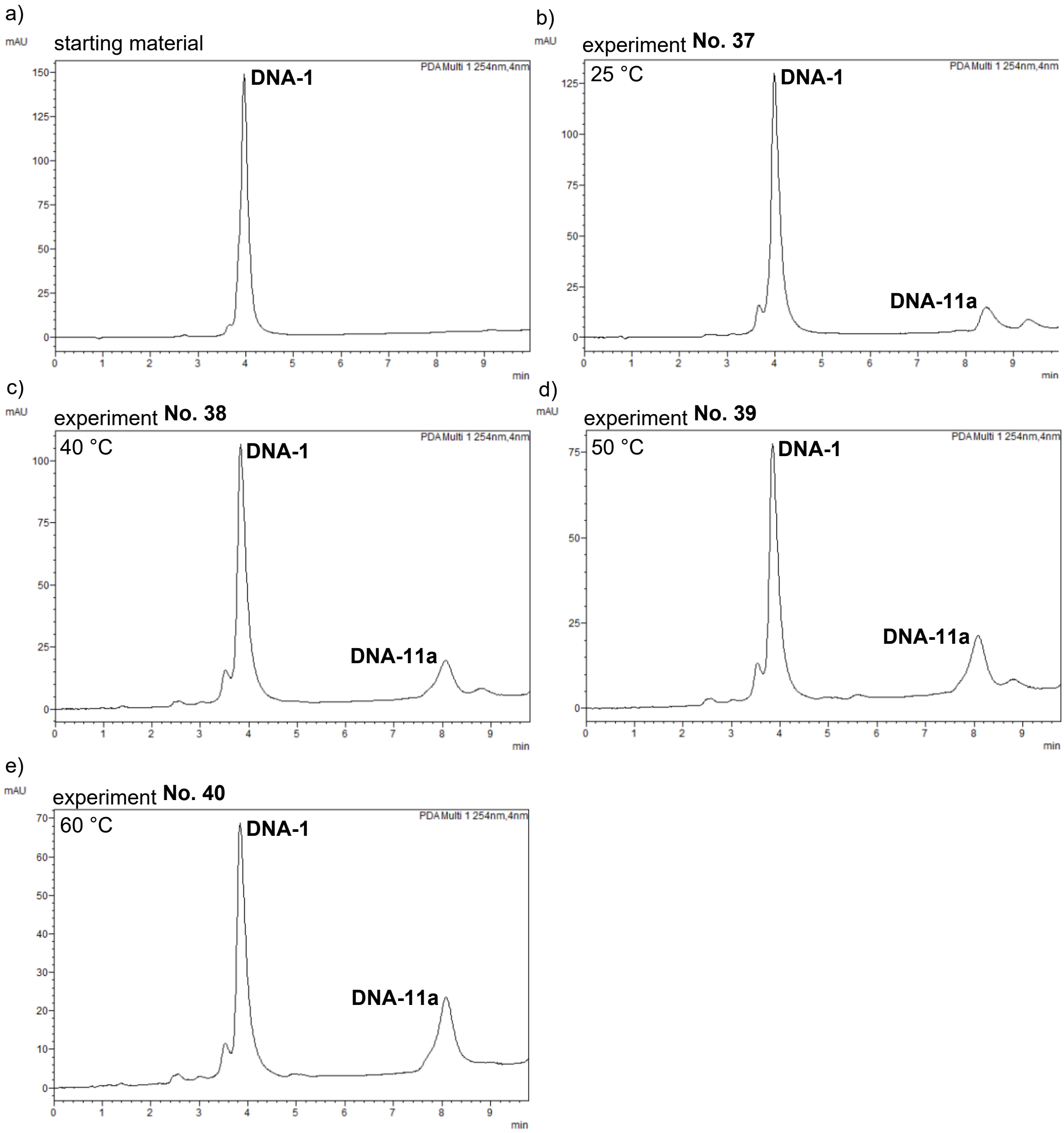

Figure S36. Effect of temperature on the Povarov reaction of the oligonucleotide-aldehyde conjugate **DNA-1**, 4-*tert*-butylaniline **9a**, and *N*-Boc-2,3-dihydro-1*H*-pyrrole **10a** after 1 hour, in the absence of the copolymer micelle, for reaction conditions see Table S13. HPLC traces show oligonucleotide-aldehyde conjugate **DNA-1** and experiments **No. 37** - **No. 40**.

4.6. Procedure for micelle-mediated synthesis of DNA-hexahydro-1*H*-pyrrolo[3,2-c]quinoline conjugates **DNA-11b** [3]

To a solution of single stranded (ss) DNA-aldehyde conjugate **DNA-1** (500 pmol) in distilled water were added aniline **9b** (125 nmol, 250 eq.) dissolved in 1.5 µL of ethyl acetate (taken from a stock solution: 12,5 µmol dissolved in 150 µL of ethyl acetate), *N*-Boc-2,3-dihydro-1*H*-pyrrole **10a** (125 nmol, 250 eq.) dissolved in 1 µL of ethyl acetate (taken from a stock solution: 12,5 µmol dissolved in 100 µL of ethyl acetate), and copolymer micelle **I/II** (6.25 nmol, 12.5 eq.) dissolved in 4 µL of distilled water taken from an aqueous stock solution (1.25 µmol in 800 µL). The reaction mixtures were filled with distilled water to a volume of 50 µL giving a final concentration of 0.125 mM of copolymer **I/II**. The reaction mixtures were shaken at room temperature for 18 hours (Table S14). The reactions were set up in triplicates. Then, 70 µL of distilled water were added and the reaction mixtures were extracted with ethyl acetate (6 x 200 µL). The aqueous solution was evaporated in a SpeedVac, the residue was redissolved in 45 µL of distilled water. All coupling products **DNA-11b** were analyzed by RP-HPLC (Phenomenex, Gemini; 5 µm, C18, 110 Å, 100*4.6 mm) with a gradient of aqueous triethylammonium acetate buffer (10 mM, pH= 8) and methanol (10% - 60% of methanol over 9 min), and by MALDI-MS analysis. The conversion was estimated based on the area under the curve of the product peak versus the starting material peak in the HPLC-trace and by MALDI-MS.

4.7. Copolymer micelle **I/II**-mediated Povarov reaction of aldehyde conjugate **DNA-1**, aniline **9b**, and *N*-Boc-2,3-dihydro-1*H*-pyrrole **10a** to DNA-conjugate **DNA-11b** at room temperature

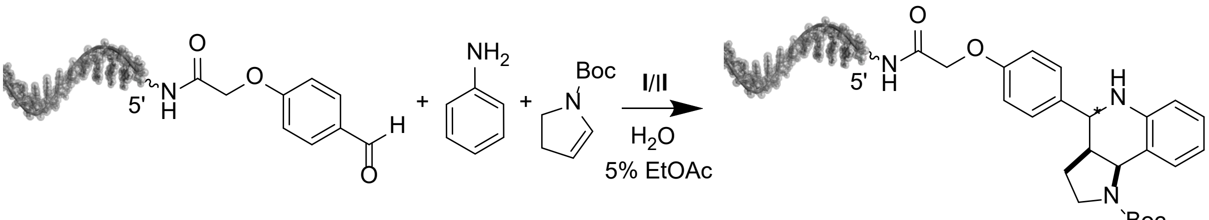

DNA-1 **9b** **10a** **DNA-11b**

Scheme S7. Reaction scheme of the copolymer micelle **I/II**-mediated Povarov reaction to **DNA-11b**.

Table S14. Impact of copolymer micelle **I/II** design on synthesis of DNA-hexahydro-1*H*-pyrrolo[3,2-*c*]quinoline conjugate **DNA-11b**. Reaction conditions: 250 eq. of **9b** and 250 eq. of **10a**, 12.5 eq. of **I/II**, 0.125 mM **I/II**, room temperature, 18 h.

No.	copolymer name	copolymer composition	**DNA-11b** 1x [%][a]	**DNA-11b** 2x [%][a]	**DNA-11b** 3x [%][a]	**DNA-11b** average [%][a]
1	**IA**	DMA_{62}-(MMA_{25}-SPA_2)	81	83	82	82
2	**IB**	DMA_{62}-(EA_{23}-SPA_2)	84	84	84	84
3	**IC$_a$**	DMA_{65}-(BA_{33}-SPA_2)	60(22)	57(20)	62(19)	60(20)
4	**IC$_b$**	DMA_{63}-(BA_{27}-SPA_2)	67(9)	70(11)	73(10)	70(10)
5	**IC$_c$**	DMA_{132}-(BA_{23}-SPA_2)	69(17)	68(17)	67(18)	68(17)
6	**ID**	DMA_{62}-(OA_{26}-SPA_2)	61(16)	63(17)	64(17)	63(17)
7	**IIA**	(DMA_{62}-SPA_2)-MMA_{14}	76(9)	77(9)	78(9)	77(9)
8	**IIB**	(DMA_{60}-SPA_2)-EA_{23}	71(11)	76(10)	77(10)	75(10)
9	**IIC$_a$**	(DMA_{63}-SPA_2)-BA_{33}	84	84	85	84
10	**IIC$_b$**	(DMA_{100}-SPA_2)-BA_{34}	76(11)	76(8)	76(10)	76(10)
11	**IID**	(DMA_{62}-SPA_2)-OA_{26}	74(10)	75(9)	72(11)	74(10)
12	**IIE**	(DMA_{62}-SPA_2)-DDA_{12}	73(11)	75(10)	73(10)	74(10)
13	-	-	48	-	-	48

[a] HPLC analysis of the crude, missing percentage to 100%: mainly **DNA-1**. Conversions in parentheses show those of a later eluting side product.

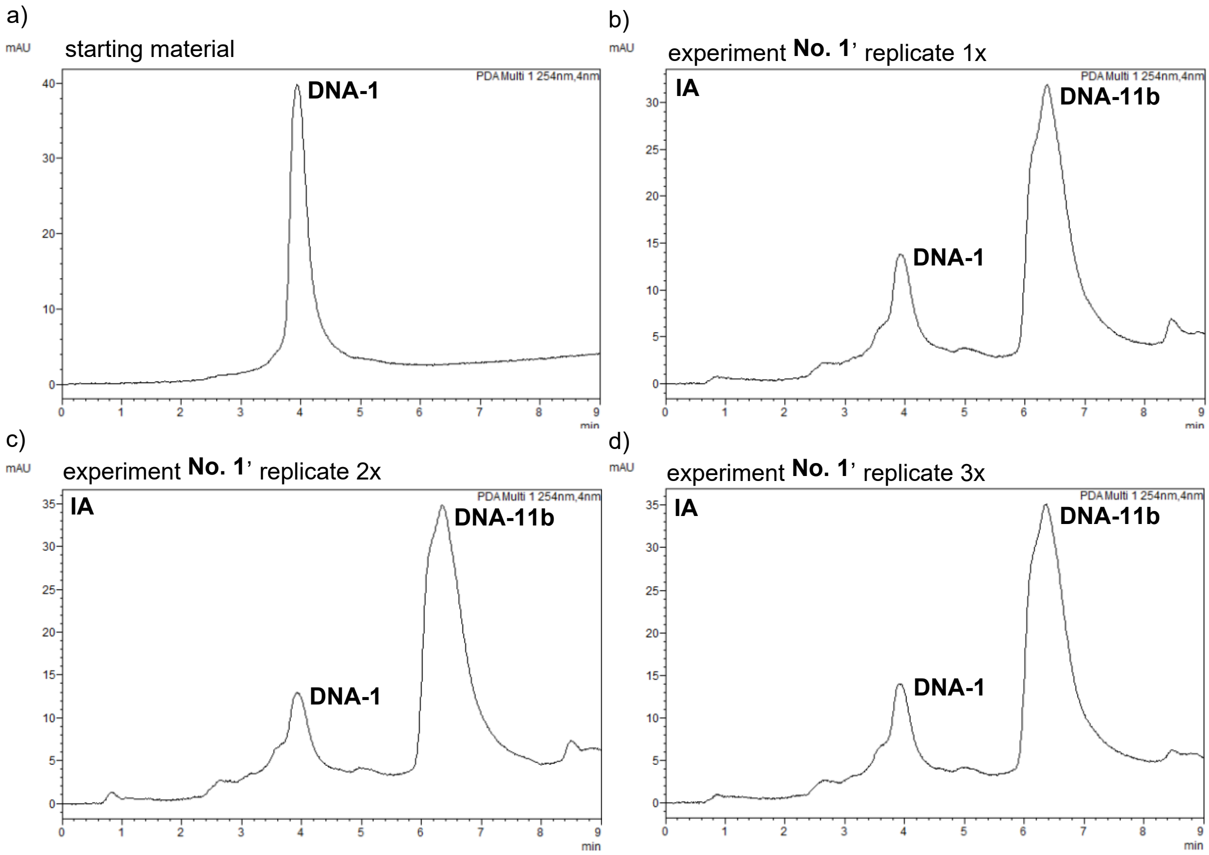

Figure S37. Copolymer **IA**-mediated Povarov reaction of the oligonucleotide-aldehyde conjugate **DNA-1**, aniline **9b**, and *N*-Boc-2,3-dihydro-1*H*-pyrrole **10a** at 25 °C, for reaction conditions see Table S14. HPLC traces show: a) oligonucleotide-aldehyde conjugate **DNA-1** and b)-d) experiment **No. 1** in triplicate.

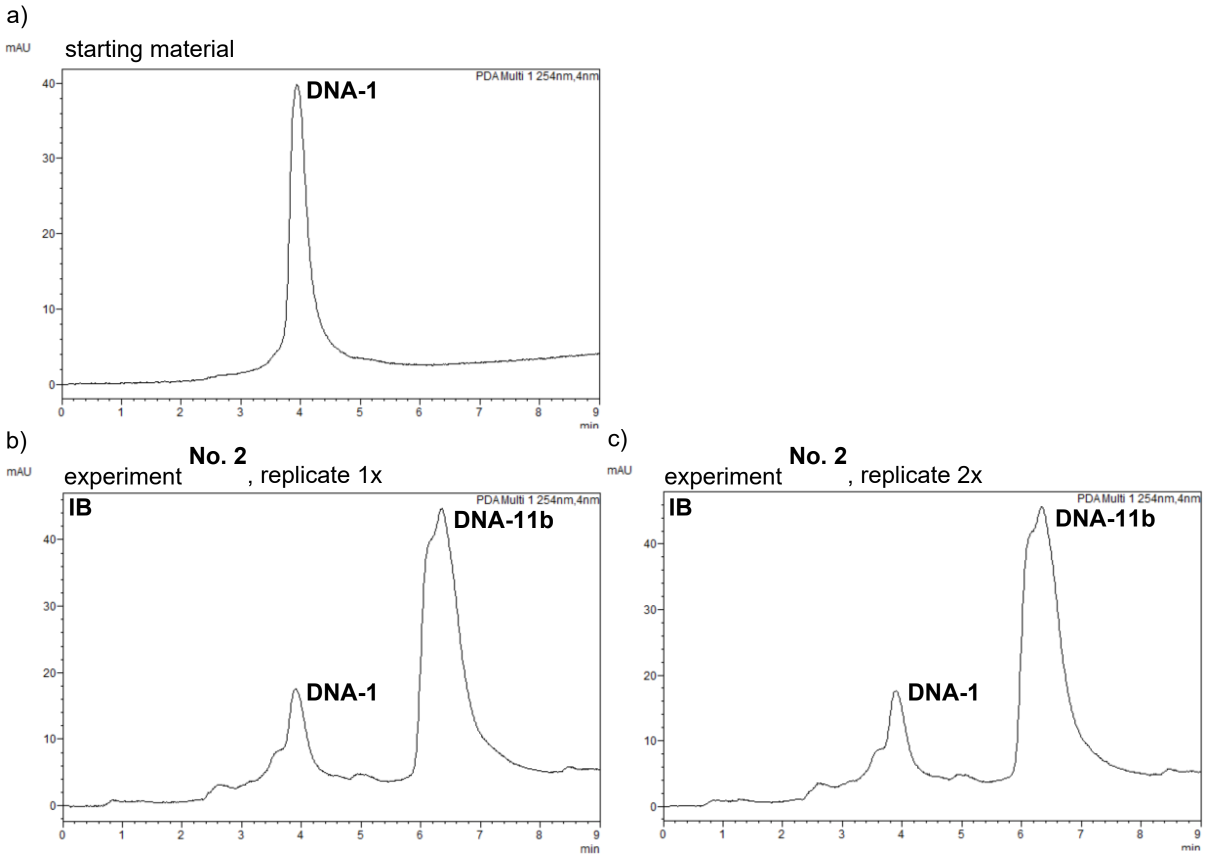

Figure S38. Copolymer **IB**-mediated Povarov reaction of the oligonucleotide-aldehyde conjugate **DNA-1**, aniline **9b**, and *N*-Boc-2,3-dihydro-1*H*-pyrrole **10a** at 25 °C, for reaction conditions see Table S14. HPLC traces show: a) oligonucleotide-aldehyde conjugate **DNA-1** and b)-c) experiment **No. 2** in duplicate.

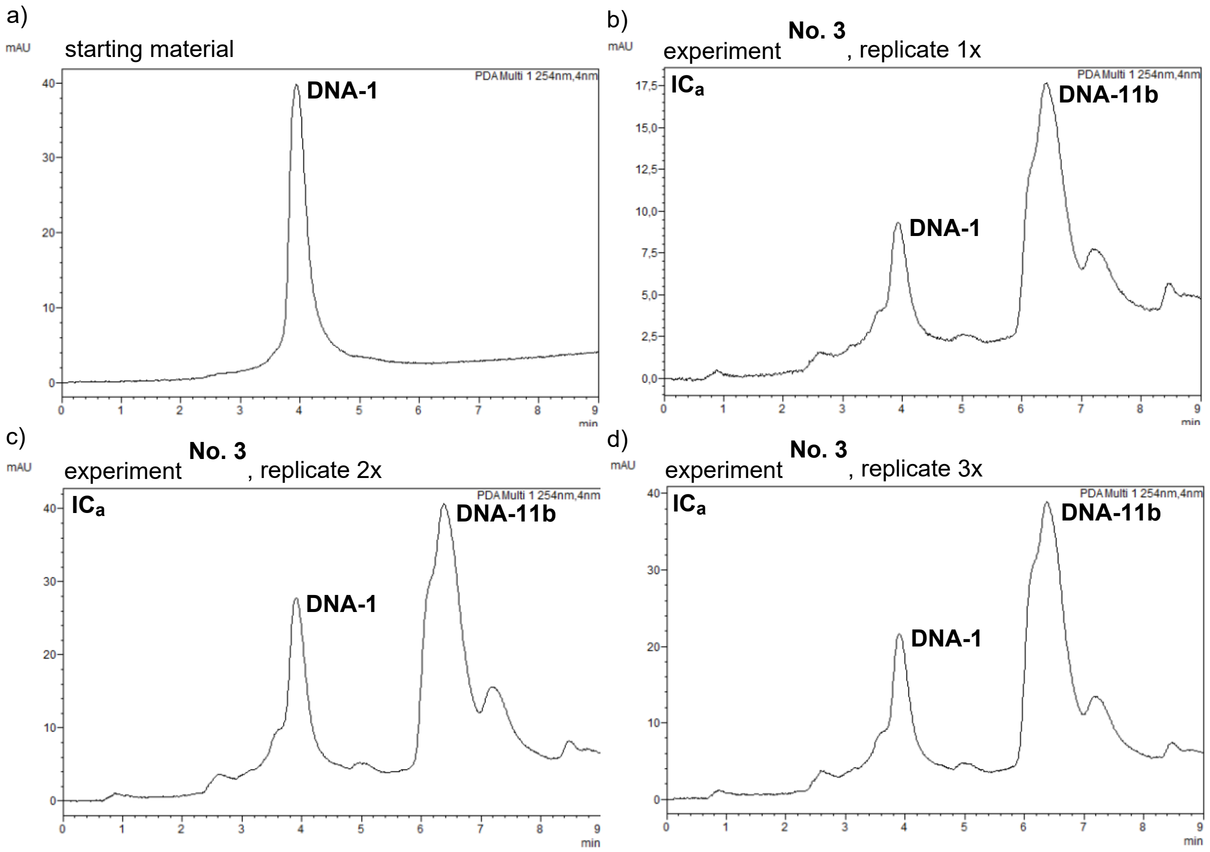

Figure S39. Copolymer **IC**$_a$-mediated Povarov reaction of the oligonucleotide-aldehyde conjugate **DNA-1**, aniline **9b**, and *N*-Boc-2,3-dihydro-1*H*-pyrrole **10a** at 25 °C, for reaction conditions see Table S14. HPLC traces show: a) oligonucleotide-aldehyde conjugate **DNA-1** and b)-d) experiment **No. 3** in triplicate.

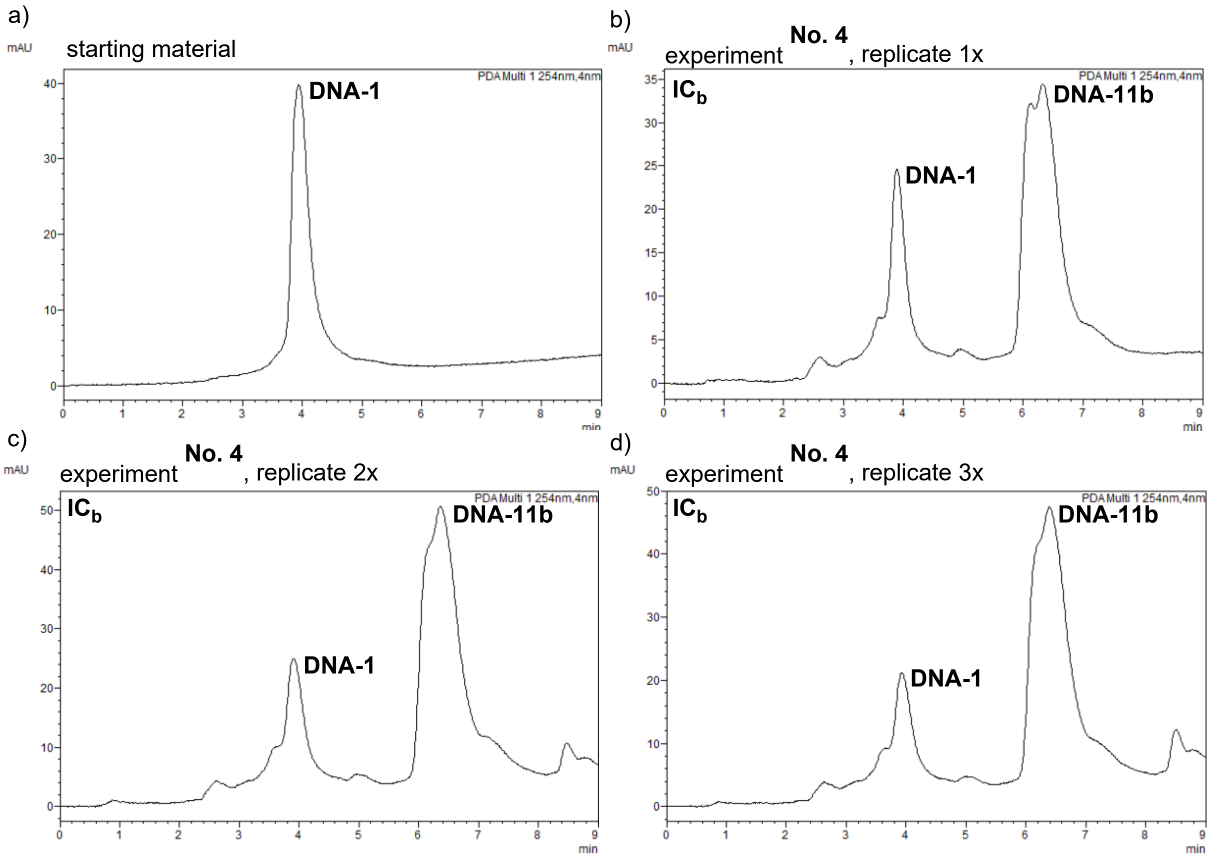

Figure S40. Copolymer **IC$_b$**-mediated Povarov reaction of the oligonucleotide-aldehyde conjugate **DNA-1**, aniline **9b**, and *N*-Boc-2,3-dihydro-1*H*-pyrrole **10a** at 25 °C, for reaction conditions see Table S14. HPLC traces show: a) oligonucleotide-aldehyde conjugate **DNA-1** and b)-d) experiment **No. 4** in triplicate.

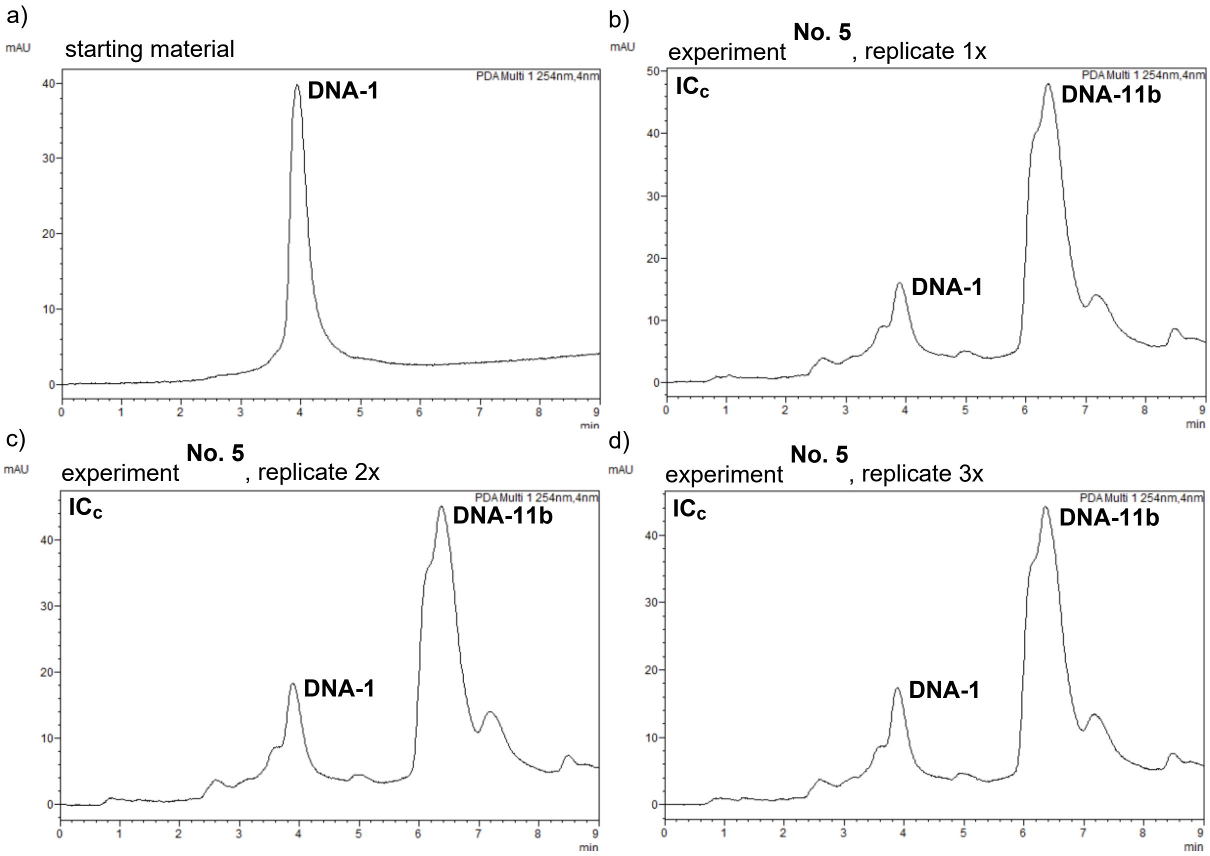

Figure S41. Copolymer **IC**$_c$-mediated Povarov reaction of the oligonucleotide-aldehyde conjugate **DNA-1**, aniline **9b**, and *N*-Boc-2,3-dihydro-1*H*-pyrrole **10a** at 25 °C, for reaction conditions see Table S14. HPLC traces show: a) oligonucleotide-aldehyde conjugate **DNA-1** and b)-d) experiment **No. 5** in triplicate.

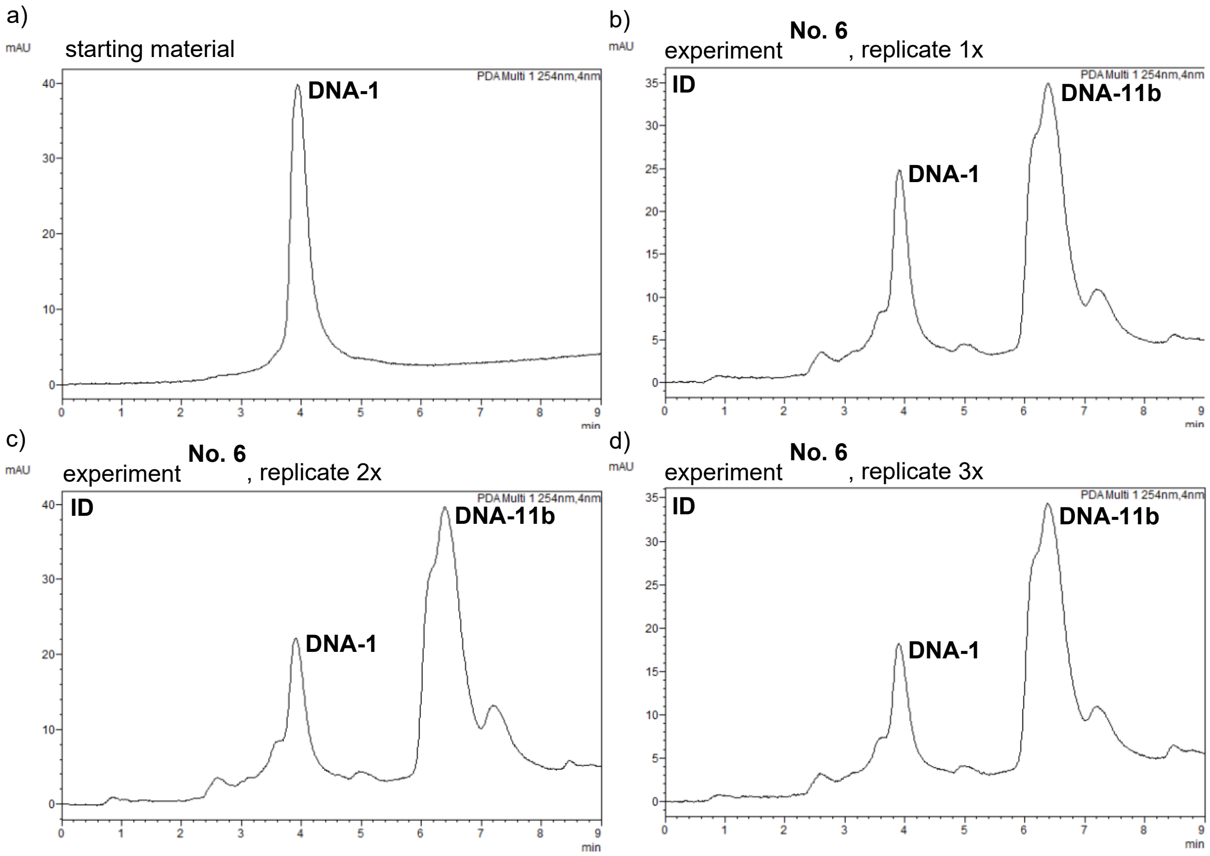

Figure S42. Copolymer **ID**-mediated Povarov reaction of the oligonucleotide-aldehyde conjugate **DNA-1**, aniline **9b**, and *N*-Boc-2,3-dihydro-1*H*-pyrrole **10a** at 25 °C, for reaction conditions see Table S14. HPLC traces show: a) oligonucleotide-aldehyde conjugate **DNA-1** and b)-d) experiment **No. 6** in triplicate.

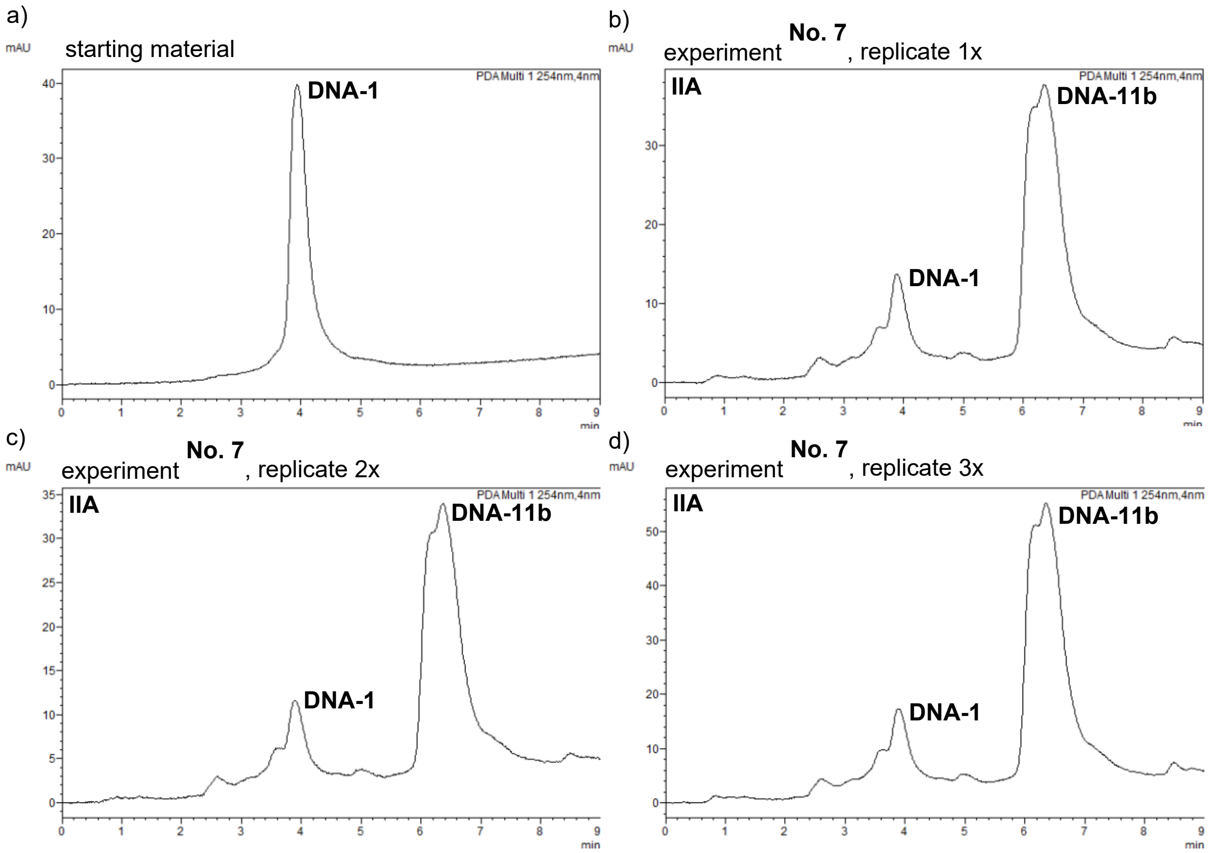

Figure S43. Copolymer **IIA**-mediated Povarov reaction of the oligonucleotide-aldehyde conjugate **DNA-1**, aniline **9b**, and *N*-Boc-2,3-dihydro-1*H*-pyrrole **10a** at 25 °C, for reaction conditions see Table S14. HPLC traces show: a) oligonucleotide-aldehyde conjugate **DNA-1** and b)-d) experiment **No. 7** in triplicate.

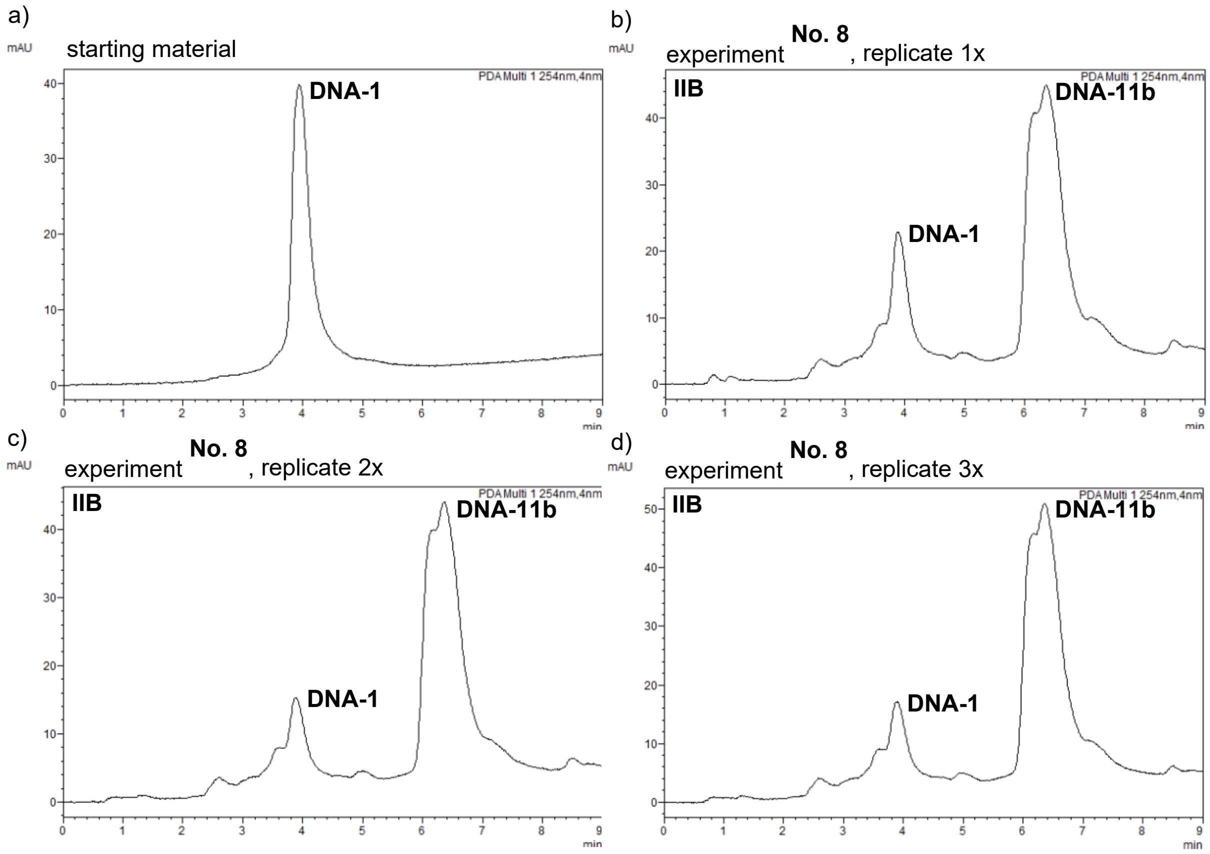

Figure S44. Copolymer **IIB**-mediated Povarov reaction of the oligonucleotide-aldehyde conjugate **DNA-1**, aniline **9b**, and *N*-Boc-2,3-dihydro-1*H*-pyrrole **10a** at 25 °C, for reaction conditions see Table S14. HPLC traces show: a) oligonucleotide-aldehyde conjugate **DNA-1** and b)-d) experiment **No. 8** in triplicate.

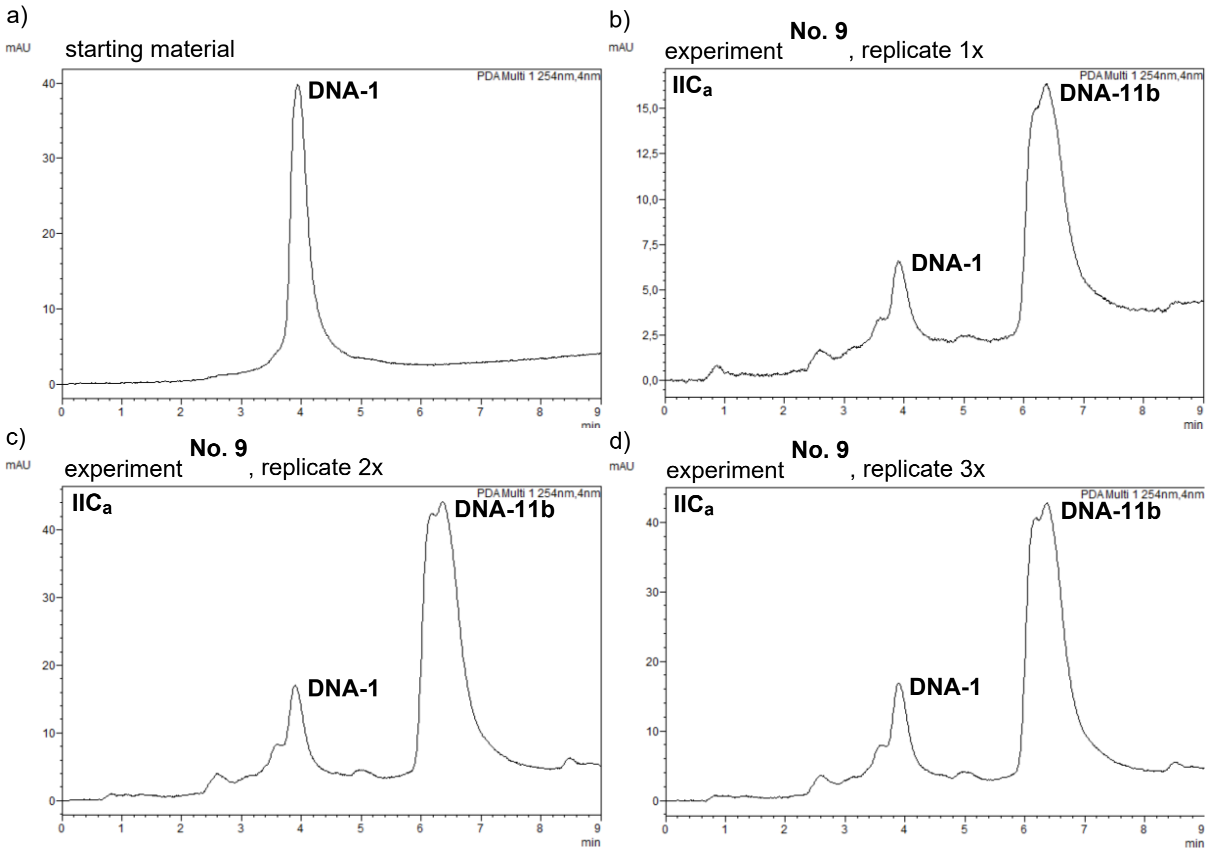

Figure S45. Copolymer **IIC$_a$**-mediated Povarov reaction of the oligonucleotide-aldehyde conjugate **DNA-1**, aniline **9b**, and *N*-Boc-2,3-dihydro-1*H*-pyrrole **10a** at 25 °C, for reaction conditions see Table S14. HPLC traces show: a) oligonucleotide-aldehyde conjugate **DNA-1** and b)-d) experiment **No. 9** in triplicate.

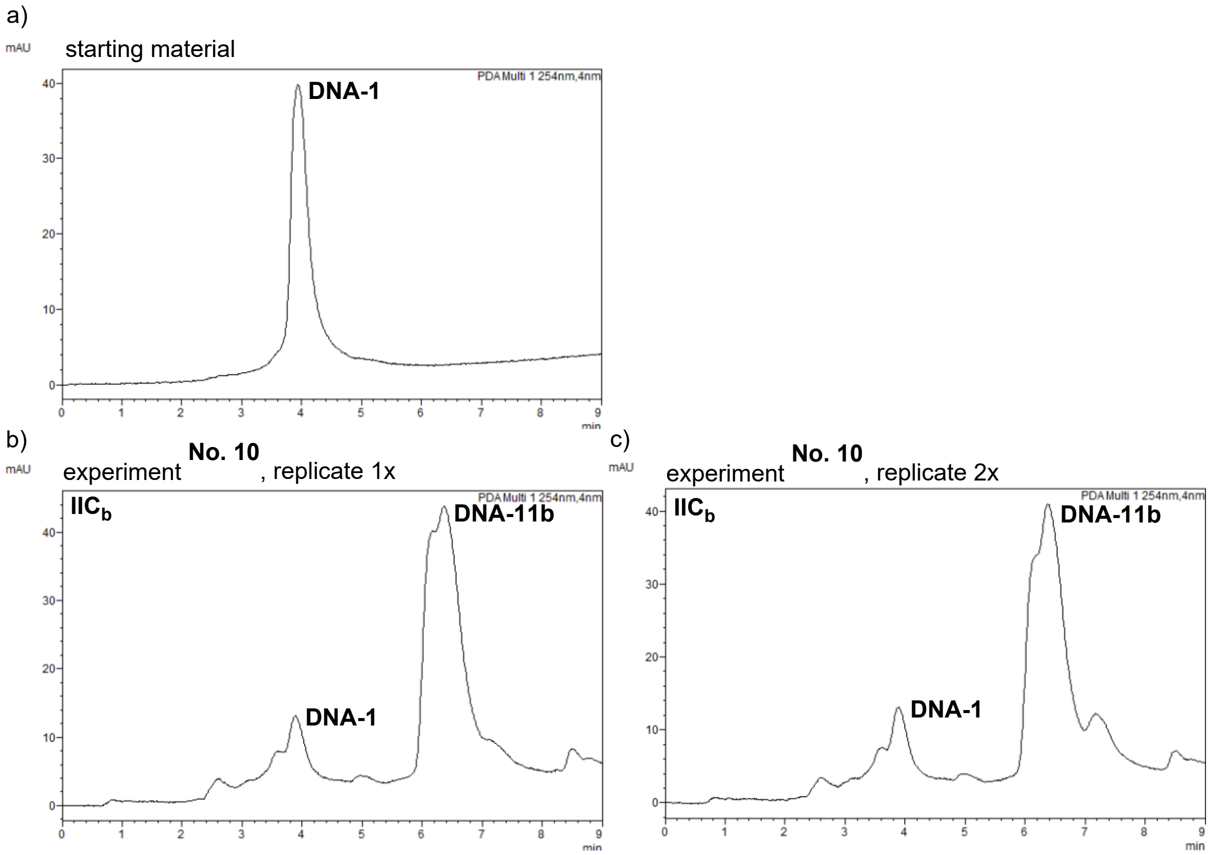

Figure S46. Copolymer **IIC$_b$**-mediated Povarov reaction of the oligonucleotide-aldehyde conjugate **DNA-1**, aniline **9b**, and *N*-Boc-2,3-dihydro-1*H*-pyrrole **10a** at 25 °C, for reaction conditions see Table S14. HPLC traces show: a) oligonucleotide-aldehyde conjugate **DNA-1** and b)-c) experiment **No. 10** in duplicate.

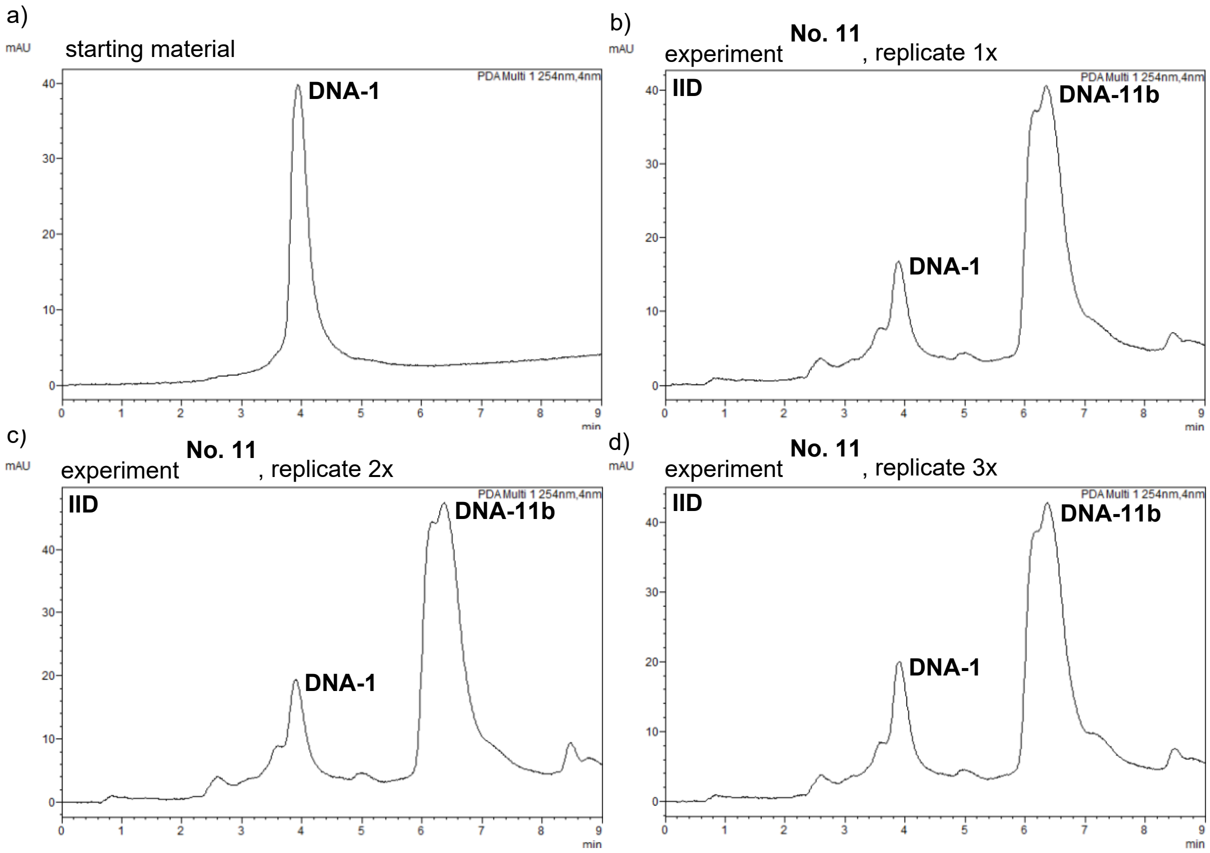

Figure S47. Copolymer **IID**-mediated Povarov reaction of the oligonucleotide-aldehyde conjugate **DNA-1**, aniline **9b**, and *N*-Boc-2,3-dihydro-1*H*-pyrrole **10a** at 25 °C, for reaction conditions see Table S14. HPLC traces show: a) oligonucleotide-aldehyde conjugate **DNA-1** and b)-d) experiment **No. 11** in triplicate.

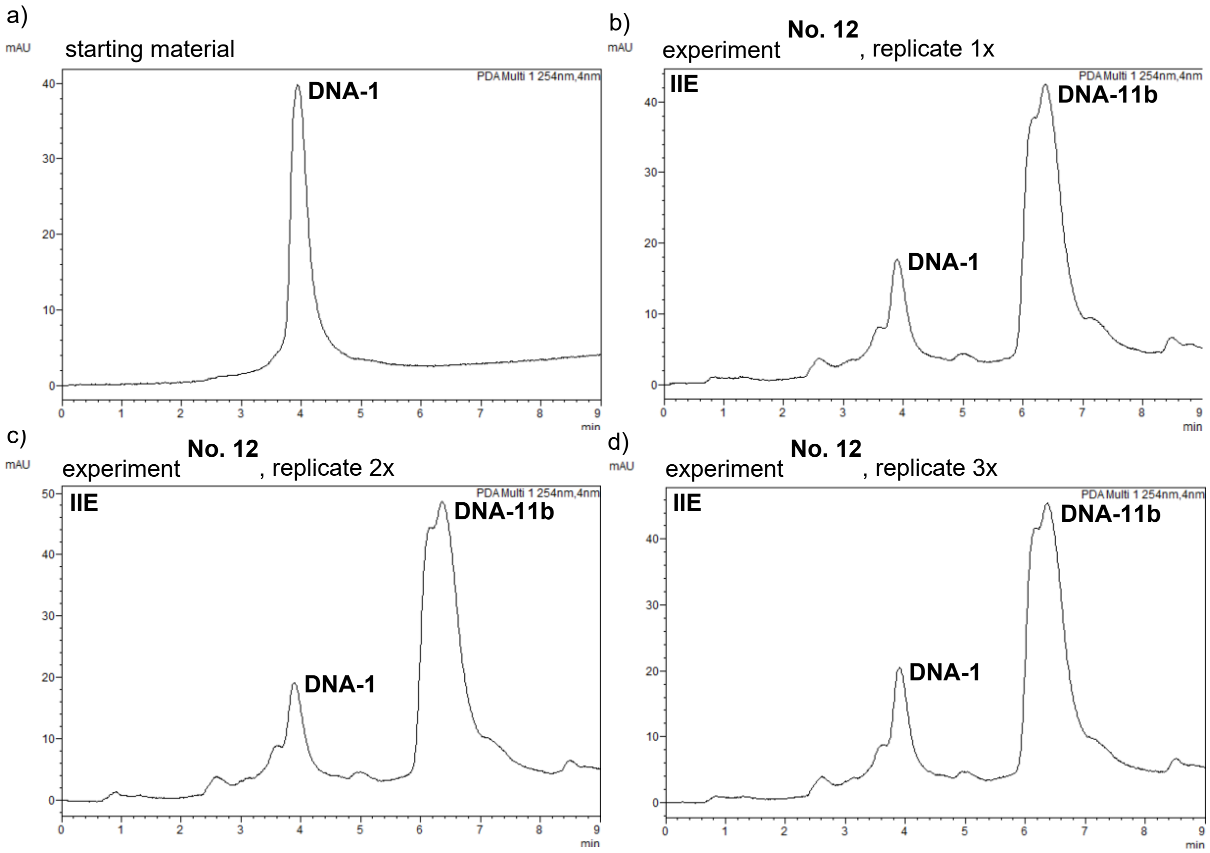

Figure S48. Copolymer **IIE**-mediated Povarov reaction of the oligonucleotide-aldehyde conjugate **DNA-1**, aniline **9b**, and *N*-Boc-2,3-dihydro-1*H*-pyrrole **10a** at 25 °C, for reaction conditions see Table S14. HPLC traces show: a) oligonucleotide-aldehyde conjugate **DNA-1** and b)-d) experiment **No. 12** in triplicate.

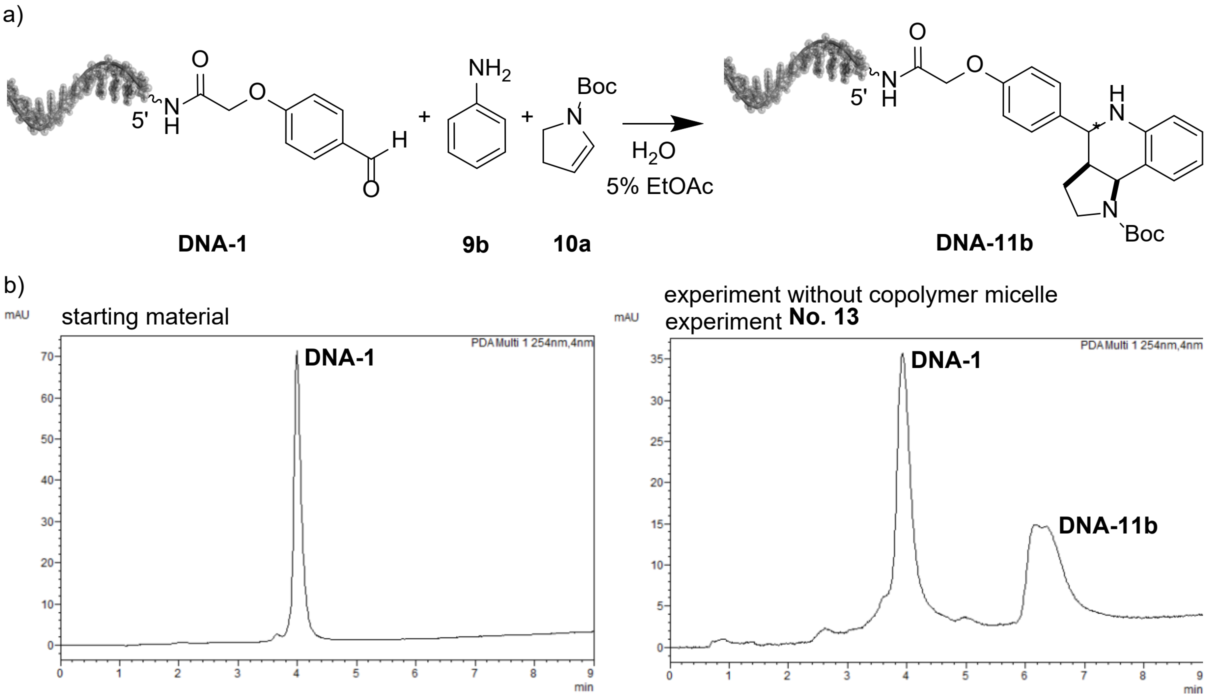

Figure S49. Povarov reaction of the oligonucleotide-aldehyde conjugate **DNA-1**, aniline **9b**, and *N*-Boc-2,3-dihydro-1*H*-pyrrole **10a** at 25 °C, in the absence of the copolymer micelle. a) Reaction scheme of the synthesis of **DNA-11b**; for reaction conditions see Table S14. b) HPLC traces show oligonucleotide-aldehyde conjugate **DNA-1** (left hand trace) and experiment **No. 13** (Table S14) (right hand trace).

a)

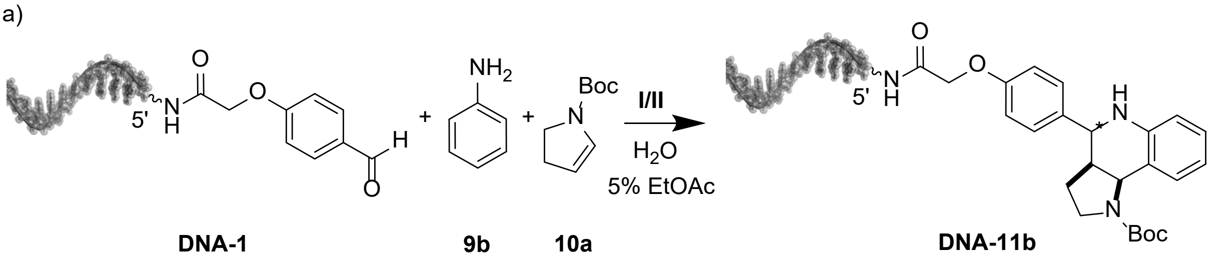

b)

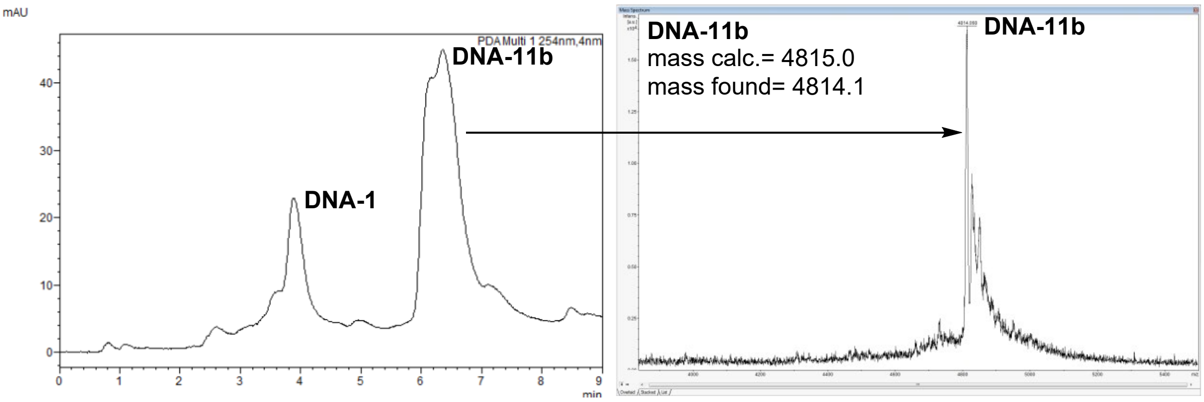

Figure S50. MALDI-MS analysis of the isolated product **DNA-11b**. a) Reaction scheme of the synthesis of **DNA-11b**; conditions: 12.5 eq. of copolymer **I/II** (0.125 mM), 250 eq. of reactant **9b** and 250 eq. of reactant **10a**, 25 °C, 18 h. b) HPLC trace of the crude reaction mixture (left hand) from Fig. S44b (Table S14, **No. 8**) and MALDI-MS spectrum of the isolated product **DNA-11b** (right hand).

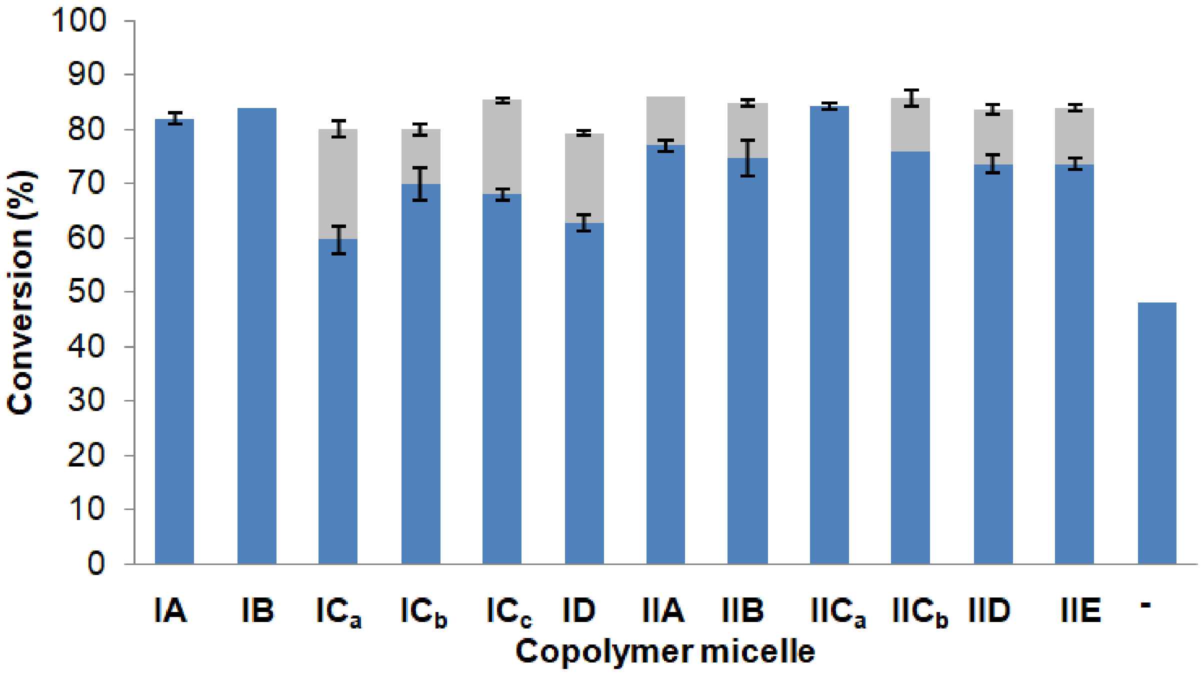

Figure S51. Investigations in the copolymer micelle structure-catalytic activity relationships for copolymer micelle-mediated Povarov reaction to **DNA-11b**. Head-to-head comparison of micelles **IA**-**ID** and **IIA**-**IIE** in the micelle-mediated Povarov reaction to **DNA-11b** (Table S14). Reaction conditions: 250 eq. aniline **9b** and 250 eq. olefin **10a**, 12.5 eq. (0.125 mM) **I/II**, room temperature, 18 hours. Blue: product **DNA-11b**, grey: later eluting side product.

4.8. Procedure for micelle-mediated synthesis of DNA-hexahydro-1*H*-pyrano[3,2-*c*]quinoline conjugates **DNA-12a** and **DNA-12c** [3]

To a solution of single stranded (ss) DNA-aldehyde conjugate **DNA-1** (500 pmol) in distilled water were added *tert*-butylaniline **9a** or 3,5-difluoroaniline **9c** (4 µmol, 8000 eq.) dissolved in 2.5 µL of ethyl acetate (taken from a stock solution: 400 µmol dissolved in 250 µL of ethyl acetate), 3,4-dihydro-2*H*-pyran **10b** (4 µmol, 8000 eq.), and copolymer micelle **I/II** (25 nmol, 50 eq.) dissolved in 16 µL of distilled water taken from an aqueous stock solution (1.25 µmol in 800 µL). The reaction mixtures were filled with distilled water to a volume of 50 µL giving a final concentration of 0.5 mM of copolymer **I/II**. The reaction mixtures were shaken at room temperature for 18 hours (Tables S15, S16). The reactions were set up in triplicates. Then, 70 µL of distilled water were added and the reaction mixtures were extracted with ethyl acetate (6 x 200 µL). The aqueous solution was evaporated in a SpeedVac, the residue was redissolved in 45 µL of distilled water. All coupling products **DNA-12** were analyzed by RP-HPLC (Phenomenex, Gemini; 5 µm, C18, 110 Å, 100*4.6 mm) with a gradient of aqueous triethylammonium acetate buffer (10 mM, pH= 8) and methanol (10% - 60% of methanol over 9 min, followed by 60% - 100% of methanol over 1 min), and by MALDI-MS analysis. The conversion was estimated based on the area under the curve of the product peak versus the starting material peak in the HPLC-trace and by MALDI-MS.

4.9. Copolymer micelle **I/II**-mediated Povarov reaction of aldehyde conjugate **DNA-1**, 3,5-difluoroaniline **9c**, and 3,4-dihydro-2*H*-pyran **10b** to DNA-conjugate **DNA-12a**

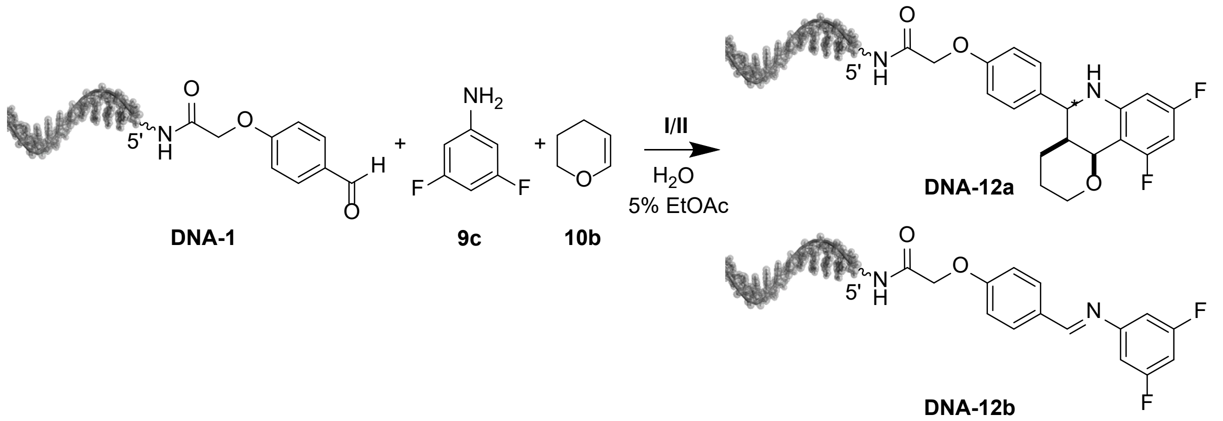

Scheme S8. Reaction scheme of the copolymer micelle **I/II**-mediated Povarov reaction to **DNA-12a**.

Table S15. Impact of copolymer micelle **I/II** design on synthesis of DNA-hexahydro-1*H*-pyrano[3,2-*c*]quinoline conjugate **DNA-12a**. Reaction conditions: 8000 eq. of **9c** and 8000 eq. of **10b**, 50 eq. of **I/II**, 0.5 mM **I/II**, room temperature, 18 hours.

No.	copolymer name	copolymer composition	**DNA-12a** 1x [%][a]	**DNA-12a** 2x [%][a]	**DNA-12a** 3x [%][a]	**DNA-12a** average [%][a]
1	**IA**	DMA_{62}-(MMA_{25}-SPA_2)	24(56)	26(55)	25(55)	25(55)
2	**IB**	DMA_{62}-(EA_{23}-SPA_2)	34(37)	35(37)	36(31)	35(35)
3	**IC$_a$**	DMA_{65}-(BA_{33}-SPA_2)	36(41)	34(47)	31(50)	34(47)
4	**IC$_b$**	DMA_{63}-(BA_{27}-SPA_2)	37(41)	36(47)	35(45)	36(44)
5	**IC$_a$**	DMA_{132}-(BA_{23}-SPA_2)	45(50)	43(51)	45(49)	44(50)
6	**ID**	DMA_{62}-(OA_{26}-SPA_2)	41(36)	39(37)	38(39)	37(39)
7	**IIA**	(DMA_{62}-SPA_2)-MMA_{14}	53(32)	60(31)	60(32)	58(32)
8	**IIB**	(DMA_{60}-SPA_2)-EA_{23}	53(30)	47(29)	52(34)	51(31)
9	**IIC$_a$**	(DMA_{63}-SPA_2)-BA_{33}	50(26)	55(24)	50(30)	52(27)
10	**IIC$_b$**	(DMA_{100}-SPA_2)-BA_{34}	63(33)	62(33)	61(34)	62(33)
11	**IID**	(DMA_{62}-SPA_2)-OA_{26}	53(33)	53(29)	53(27)	53(30)
12	**IIE**	(DMA_{62}-SPA_2)-DDA_{12}	43(28)	42(28)	40(31)	42(29)
13	-	-	0	0	0	0

[a] HPLC analysis of the crude, missing percentage to 100%: mainly **DNA-1**. Conversions in parentheses show those of imine intermediate **DNA-12b**.

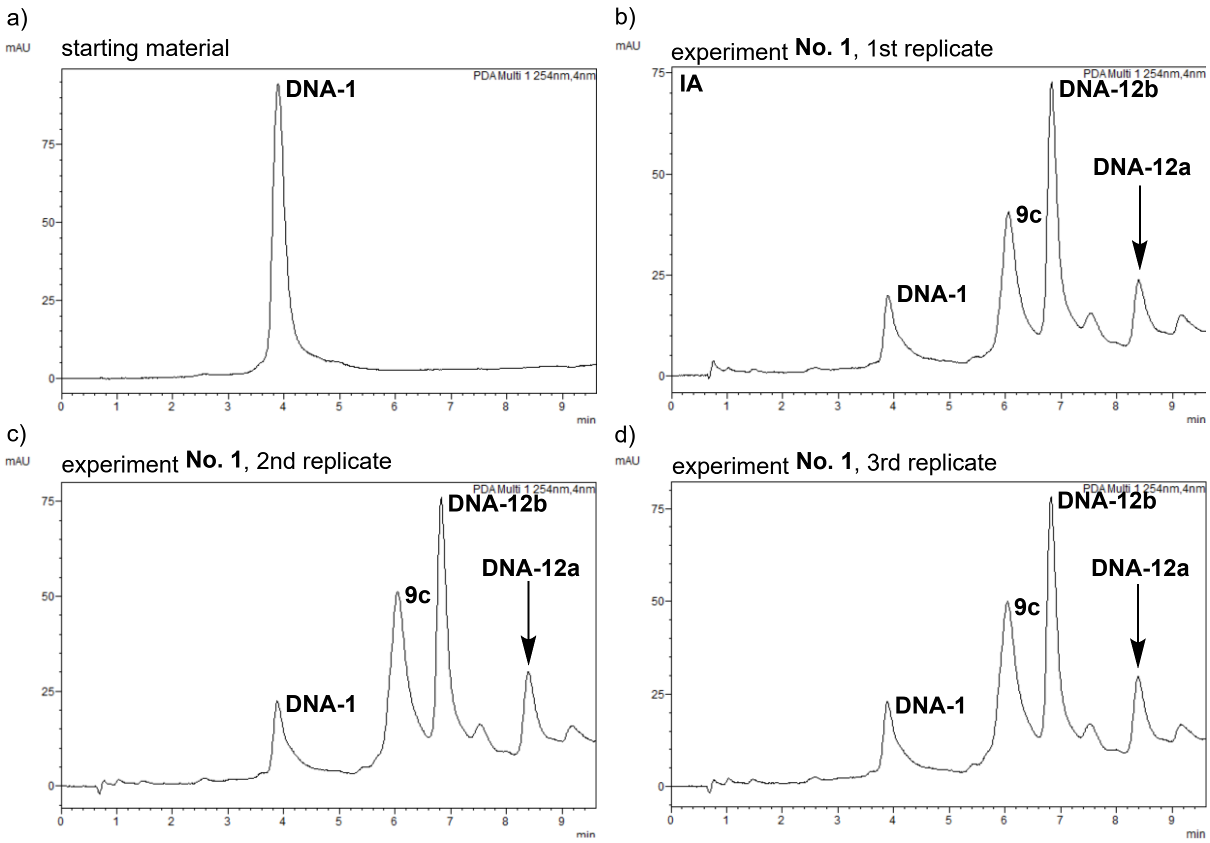

Figure S52. Copolymer **IA**-mediated Povarov reaction of the oligonucleotide-aldehyde conjugate **DNA-1**, aniline **9c**, and 3,4-dihydro-2*H*-pyran **10b** at 25 °C, for reaction conditions see Table S15. HPLC traces show: a) oligonucleotide-aldehyde conjugate **DNA-1** and b)-d) experiment **No. 1** in triplicate.

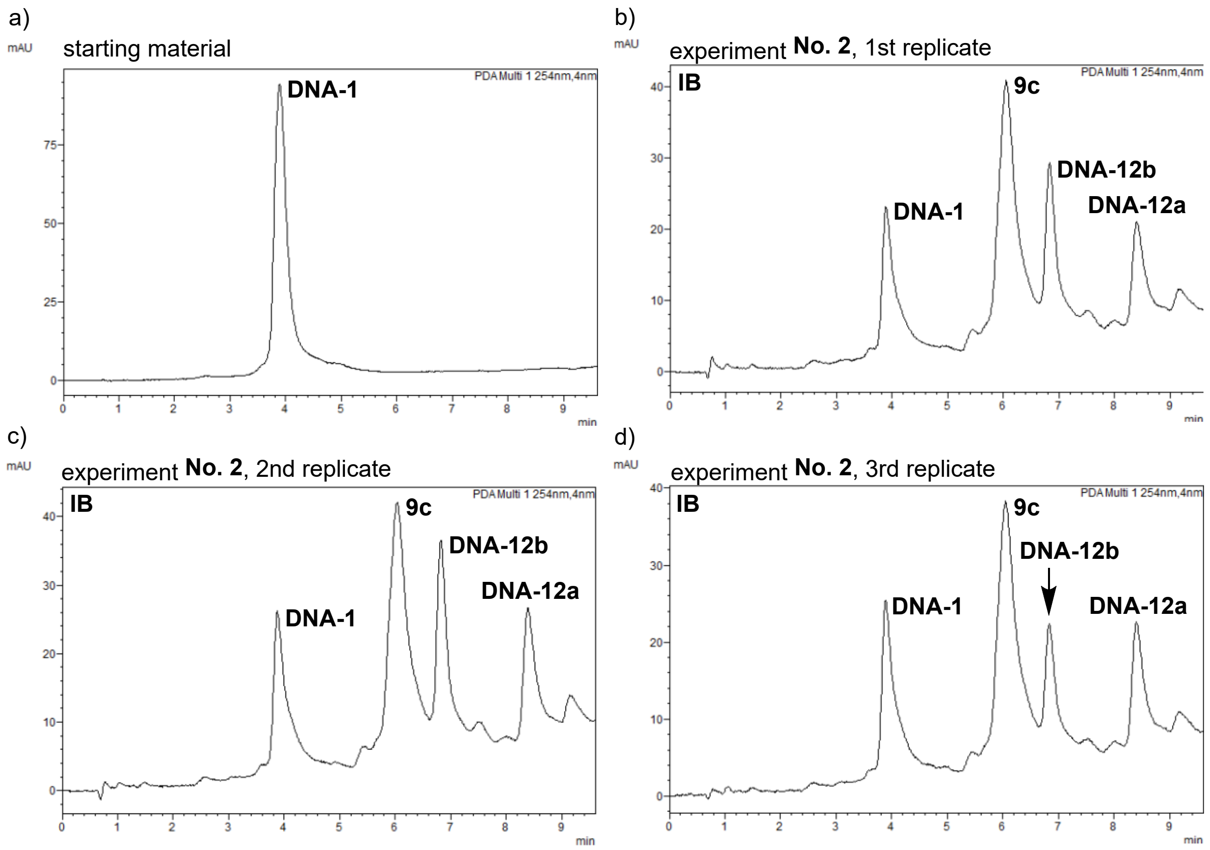

Figure S53. Copolymer **IB**-mediated Povarov reaction of the oligonucleotide-aldehyde conjugate **DNA-1**, aniline **9c**, and 3,4-dihydro-2*H*-pyran **10b** at 25 °C, for reaction conditions see Table S15. HPLC traces show: a) oligonucleotide-aldehyde conjugate **DNA-1** and b)-d) experiment **No. 2** in triplicate.

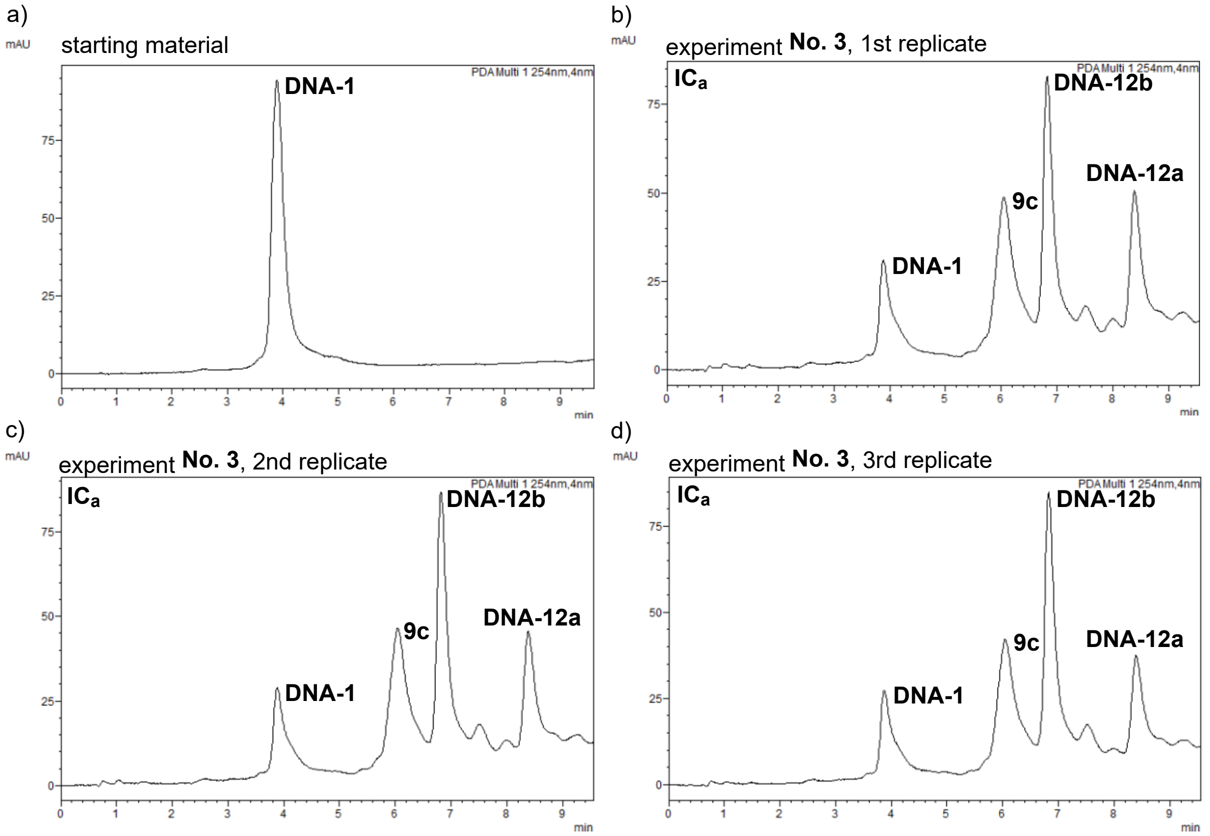

Figure S54. Copolymer **IC$_a$**-mediated Povarov reaction of the oligonucleotide-aldehyde conjugate **DNA-1**, aniline **9c**, and 3,4-dihydro-2*H*-pyran **10b** at 25 °C, for reaction conditions see Table S15. HPLC traces show: a) oligonucleotide-aldehyde conjugate **DNA-1** and b)-d) experiment **No. 3** in triplicate.

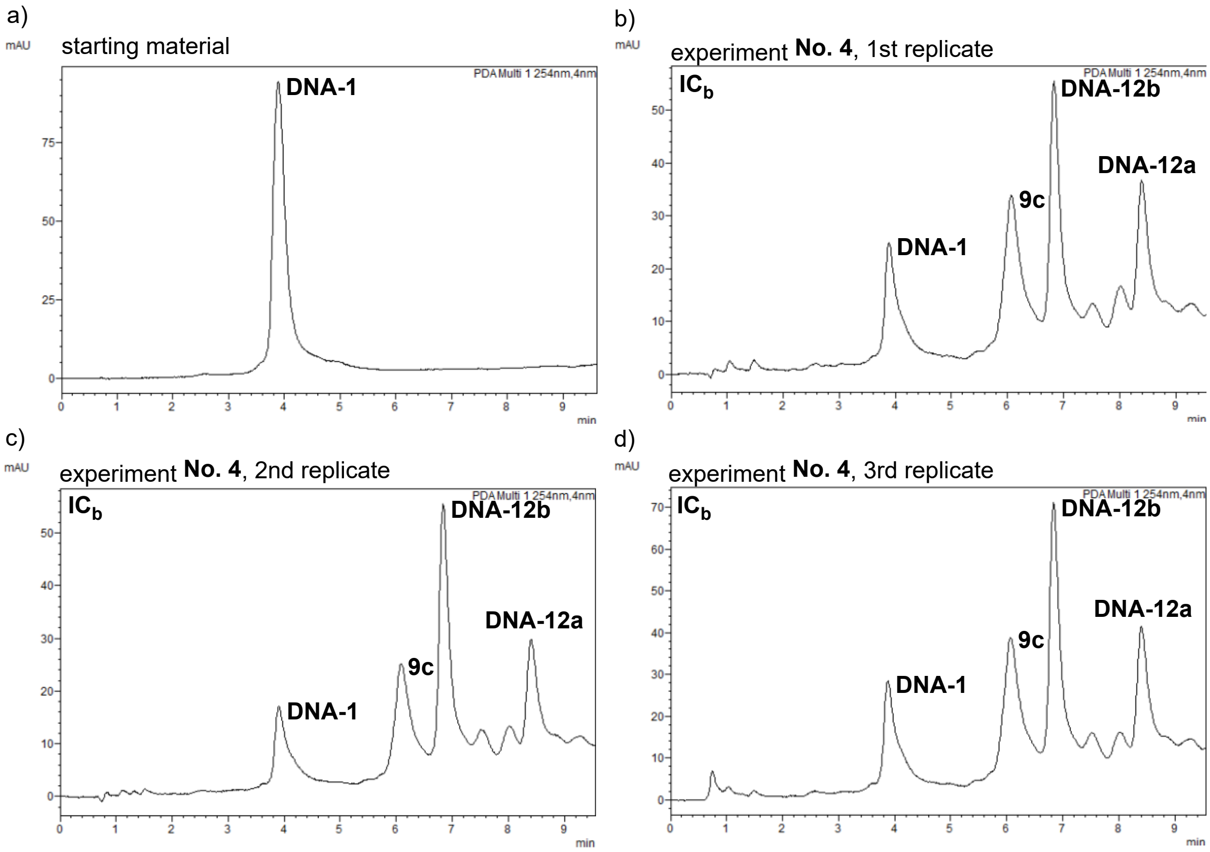

Figure S55. Copolymer **IC$_b$**-mediated Povarov reaction of the oligonucleotide-aldehyde conjugate **DNA-1**, aniline **9c**, and 3,4-dihydro-2*H*-pyran **10b** at 25 °C, for reaction conditions see Table S15. HPLC traces show: a) oligonucleotide-aldehyde conjugate **DNA-1** and b)-d) experiment **No. 4** in triplicate.

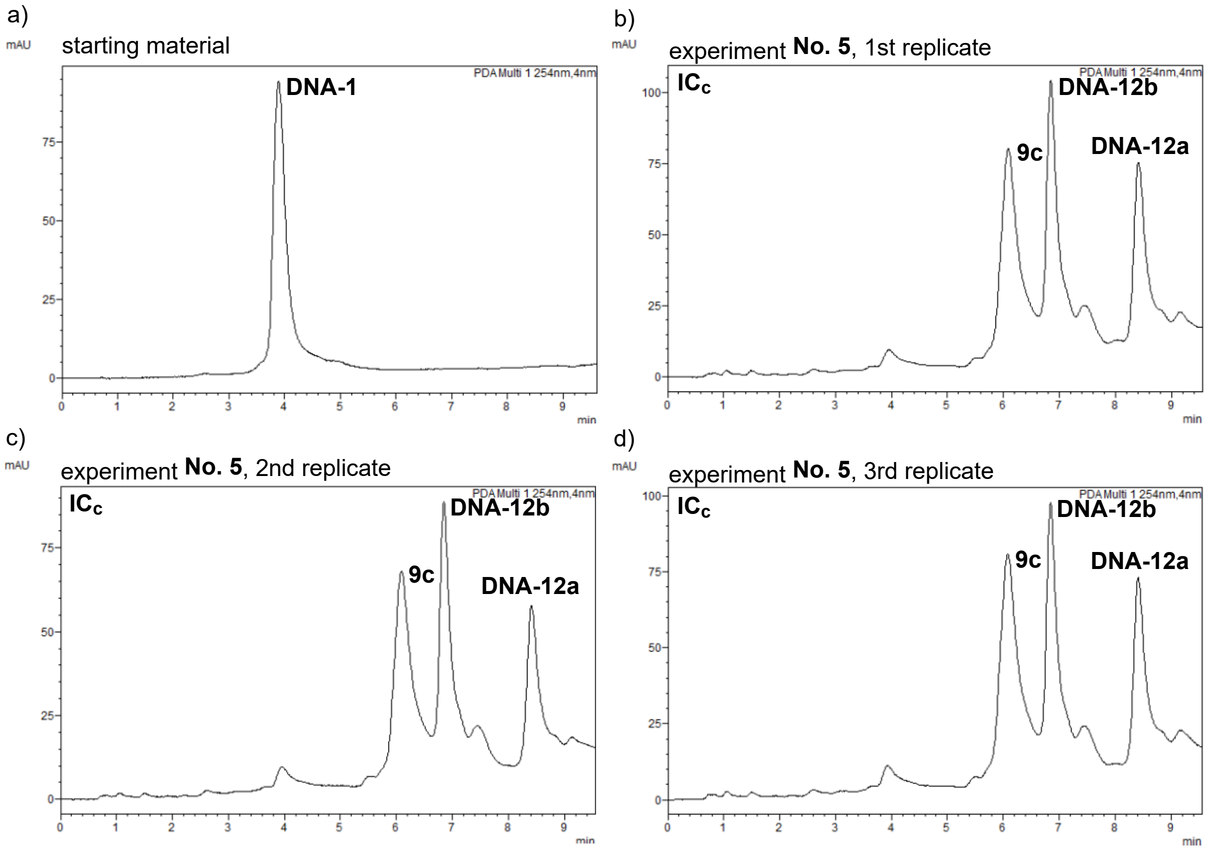

Figure S56. Copolymer **IC$_c$**-mediated Povarov reaction of the oligonucleotide-aldehyde conjugate **DNA-1**, aniline **9c**, and 3,4-dihydro-2*H*-pyran **10b** at 25 °C, for reaction conditions see Table S15. HPLC traces show: a) oligonucleotide-aldehyde conjugate **DNA-1** and b)-d) experiment **No. 5** in triplicate.

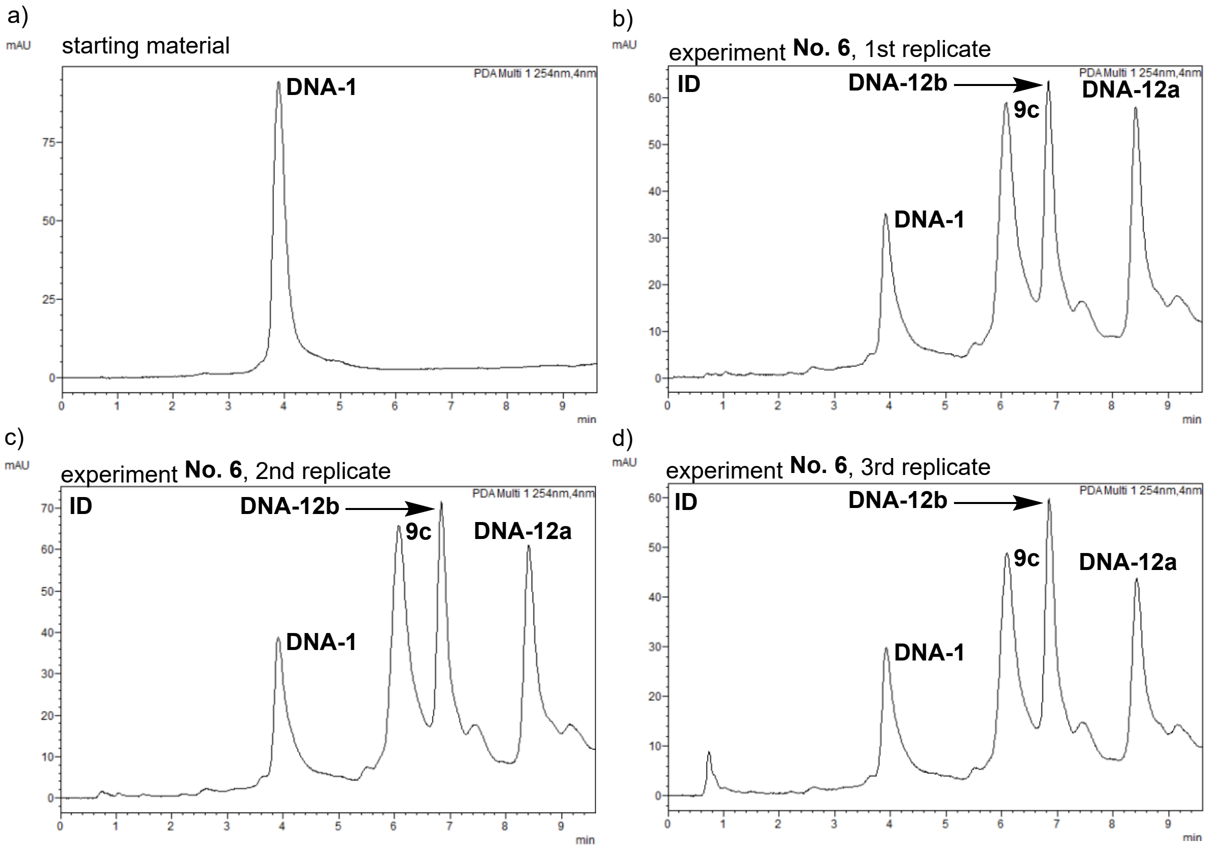

Figure S57. Copolymer **ID**-mediated Povarov reaction of the oligonucleotide-aldehyde conjugate **DNA-1**, aniline **9c**, and 3,4-dihydro-2*H*-pyran **10b** at 25 °C, for reaction conditions see Table S15. HPLC traces show: a) oligonucleotide-aldehyde conjugate **DNA-1** and b)-d) experiment **No. 6** in triplicate.

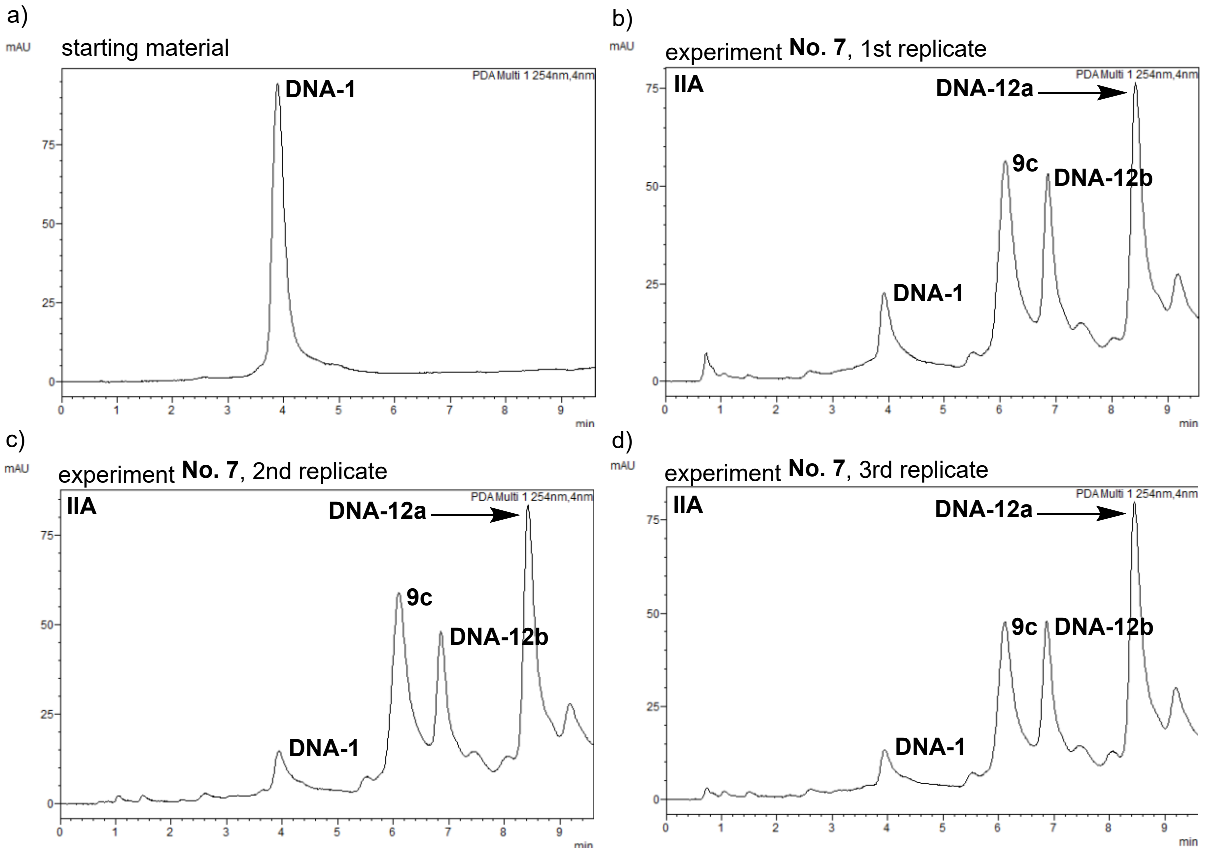

Figure S58. Copolymer **IIA**-mediated Povarov reaction of the oligonucleotide-aldehyde conjugate **DNA-1**, aniline **9c**, and 3,4-dihydro-2*H*-pyran **10b** at 25 °C, for reaction conditions see Table S15. HPLC traces show: a) oligonucleotide-aldehyde conjugate **DNA-1** and b)-d) experiment **No. 7** in triplicate.

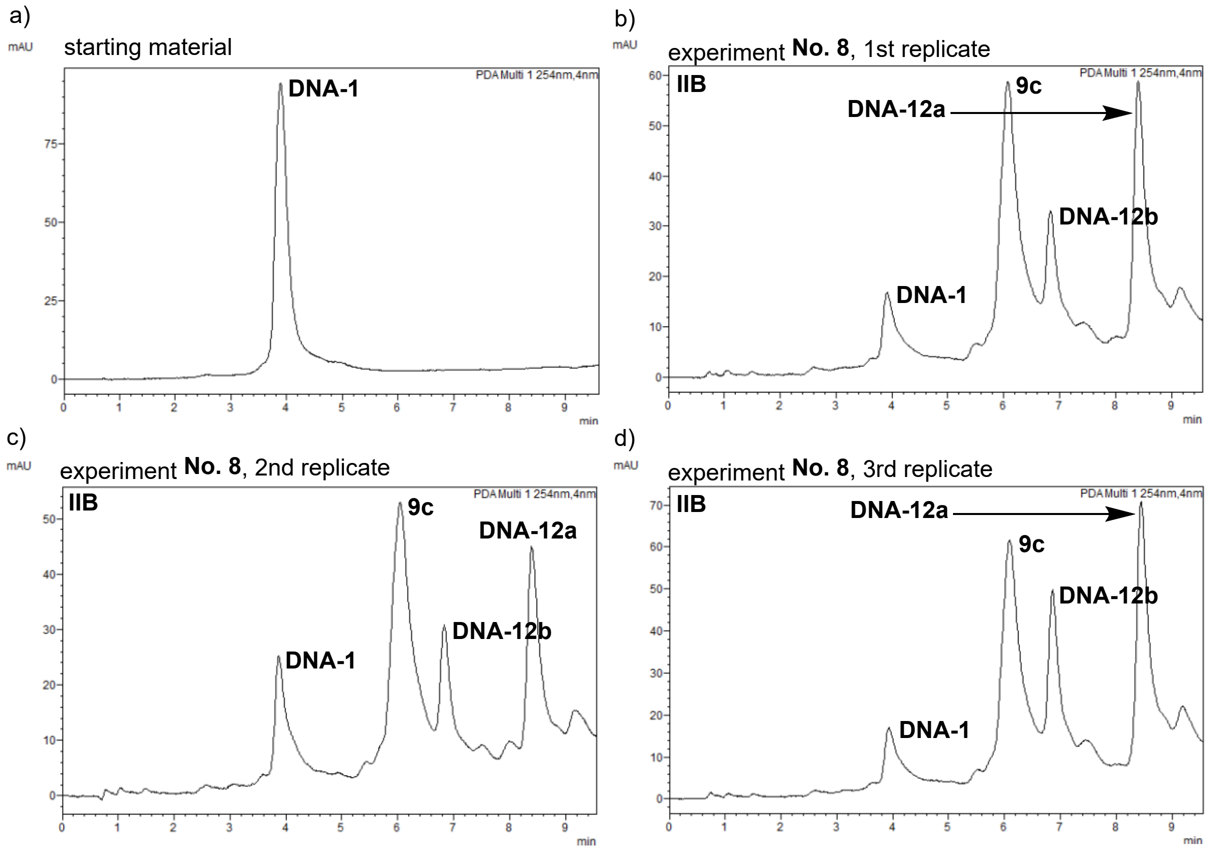

Figure S59. Copolymer **IIB**-mediated Povarov reaction of the oligonucleotide-aldehyde conjugate **DNA-1**, aniline **9c**, and 3,4-dihydro-2*H*-pyran **10b** at 25 °C, for reaction conditions see Table S15. HPLC traces show: a) oligonucleotide-aldehyde conjugate **DNA-1** and b)-d) experiment **No. 8** in triplicate.

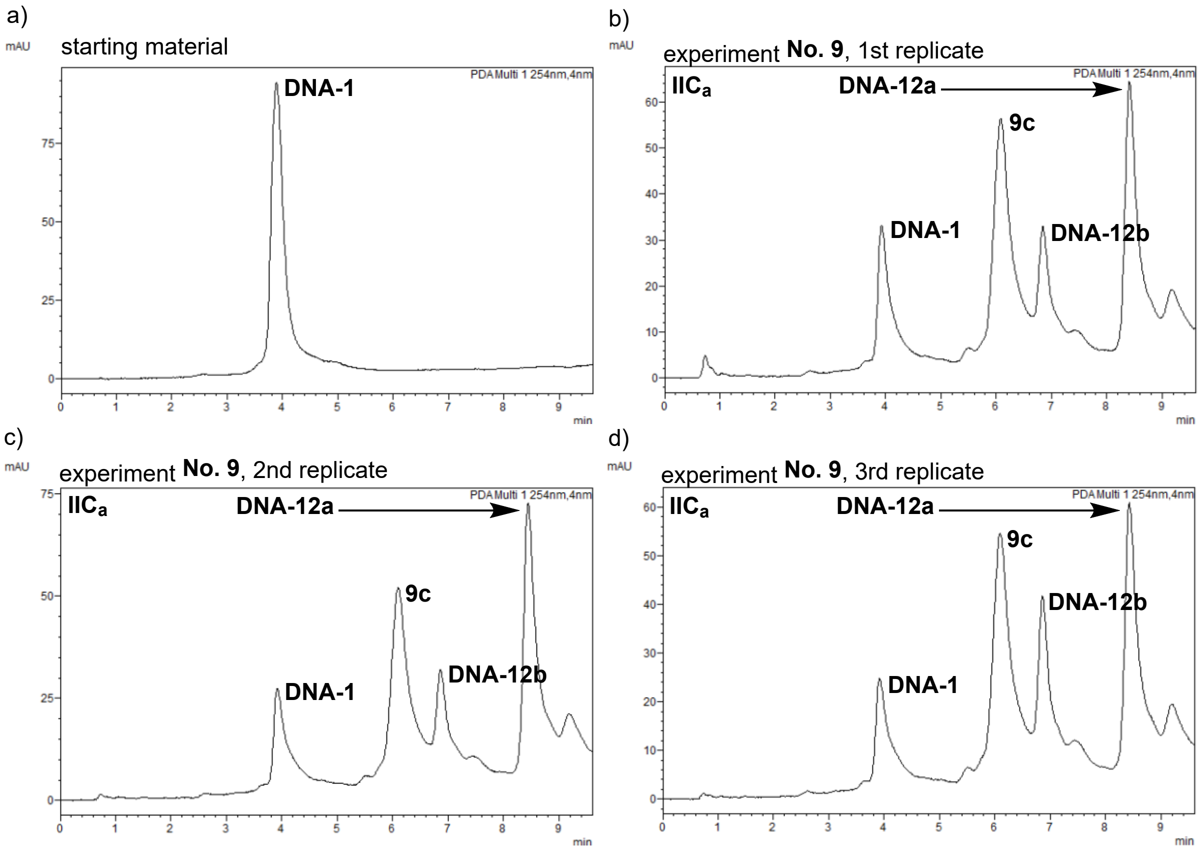

Figure S60. Copolymer **IIC**$_a$-mediated Povarov reaction of the oligonucleotide-aldehyde conjugate **DNA-1**, aniline **9c**, and 3,4-dihydro-2*H*-pyran **10b** at 25 °C, for reaction conditions see Table S16. HPLC traces show: a) oligonucleotide-aldehyde conjugate **DNA-1** and b)-d) experiment **No. 9** in triplicate.

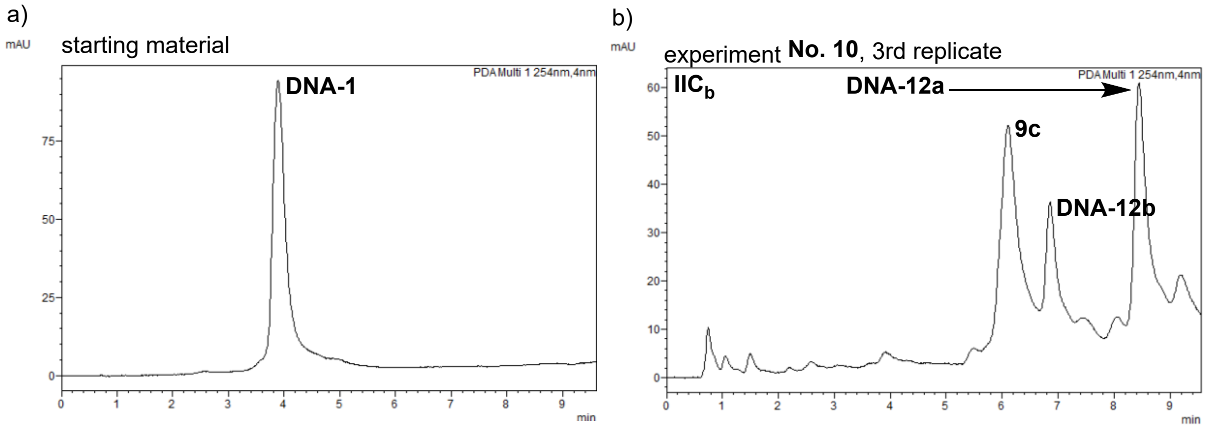

Figure S61. Copolymer **IIC**$_b$-mediated Povarov reaction of the oligonucleotide-aldehyde conjugate **DNA-1**, aniline **9c**, and 3,4-dihydro-2*H*-pyran **10b** at 25 °C, for reaction conditions see Table S15. HPLC traces show: a) oligonucleotide-aldehyde conjugate **DNA-1** and b) experiment **No. 10** (replicate 3x).

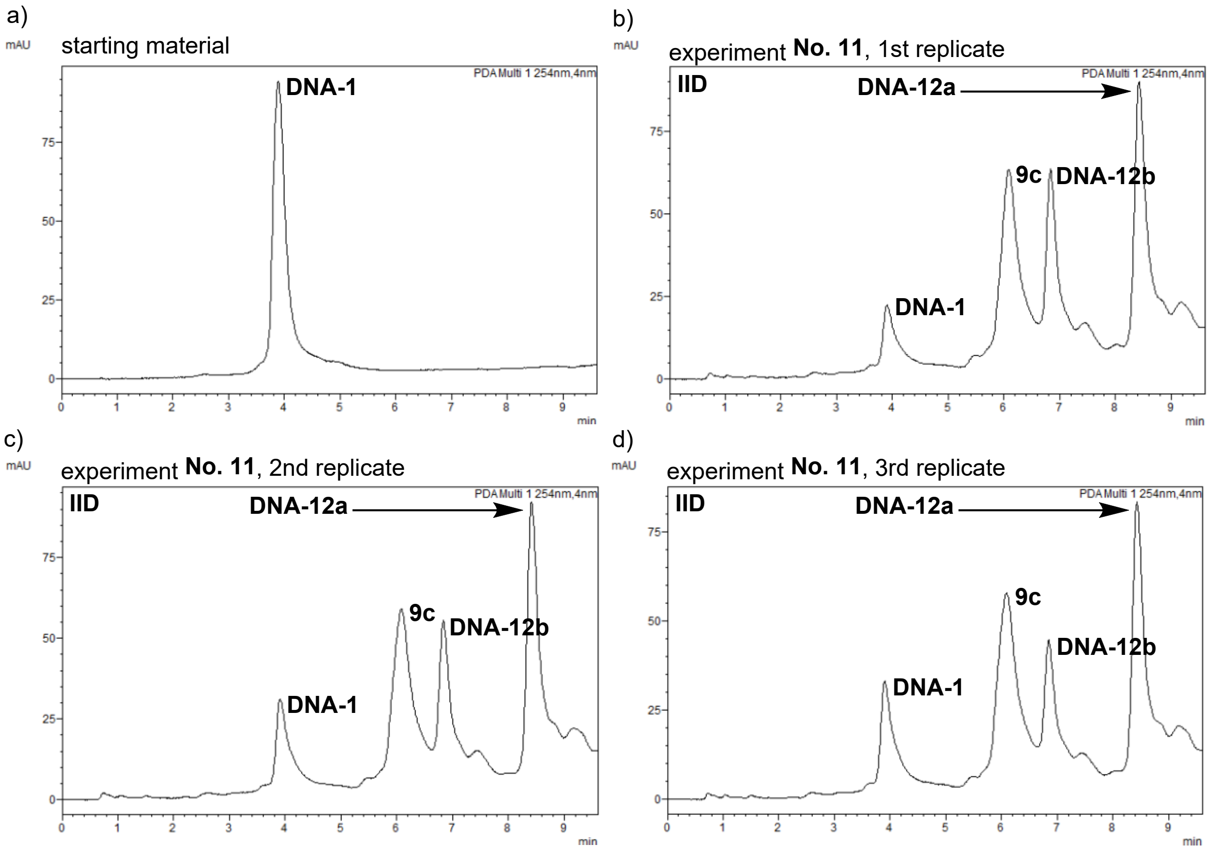

Figure S62. Copolymer **IID**-mediated Povarov reaction of the oligonucleotide-aldehyde conjugate **DNA-1**, aniline **9c**, and 3,4-dihydro-2*H*-pyran **10b** at 25 °C, for reaction conditions see Table S15. HPLC traces show: a) oligonucleotide-aldehyde conjugate **DNA-1** and b)-d) experiment **No. 11** in triplicate.

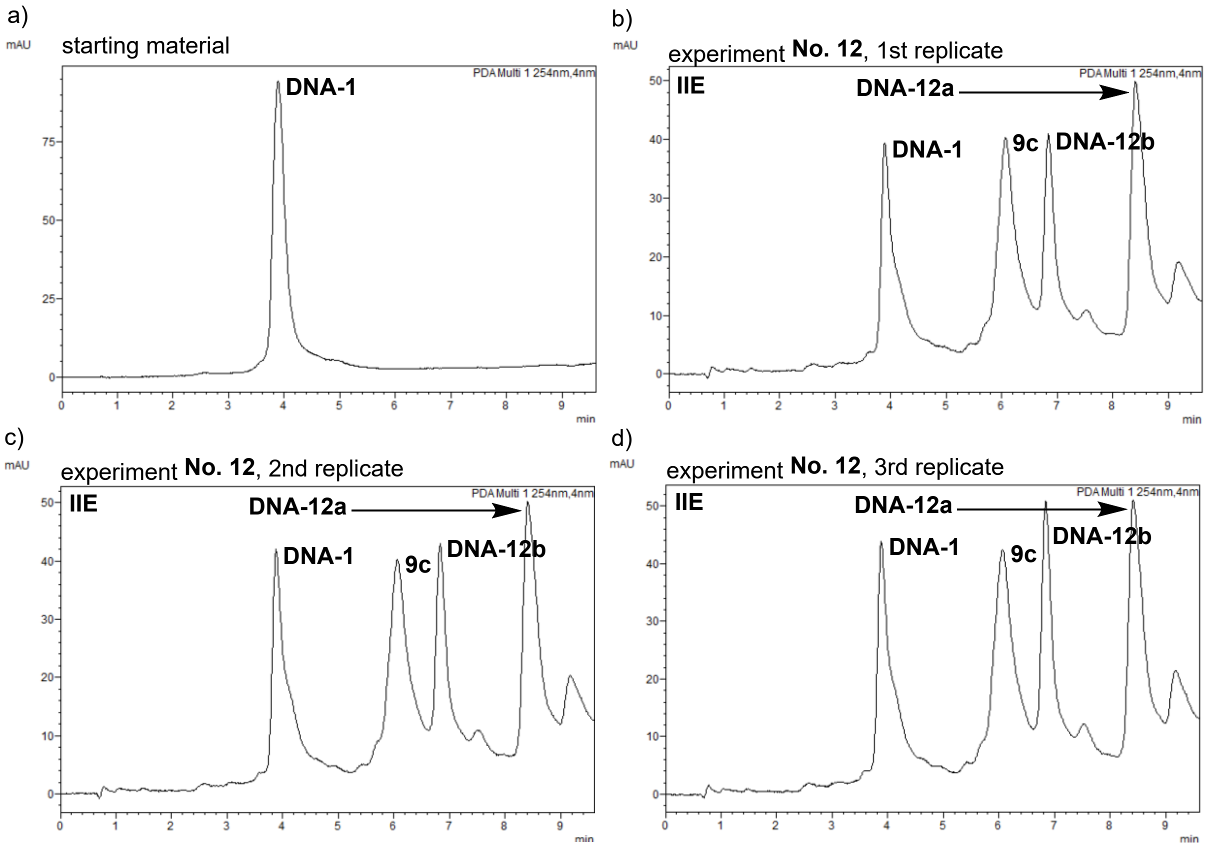

Figure S63. Copolymer **IIE**-mediated Povarov reaction of the oligonucleotide-aldehyde conjugate **DNA-1**, aniline **9c**, and 3,4-dihydro-2*H*-pyran **10b** at 25 °C, for reaction conditions see Table S15. HPLC traces show: a) oligonucleotide-aldehyde conjugate **DNA-1** and b)-d) experiment **No. 12** in triplicate.

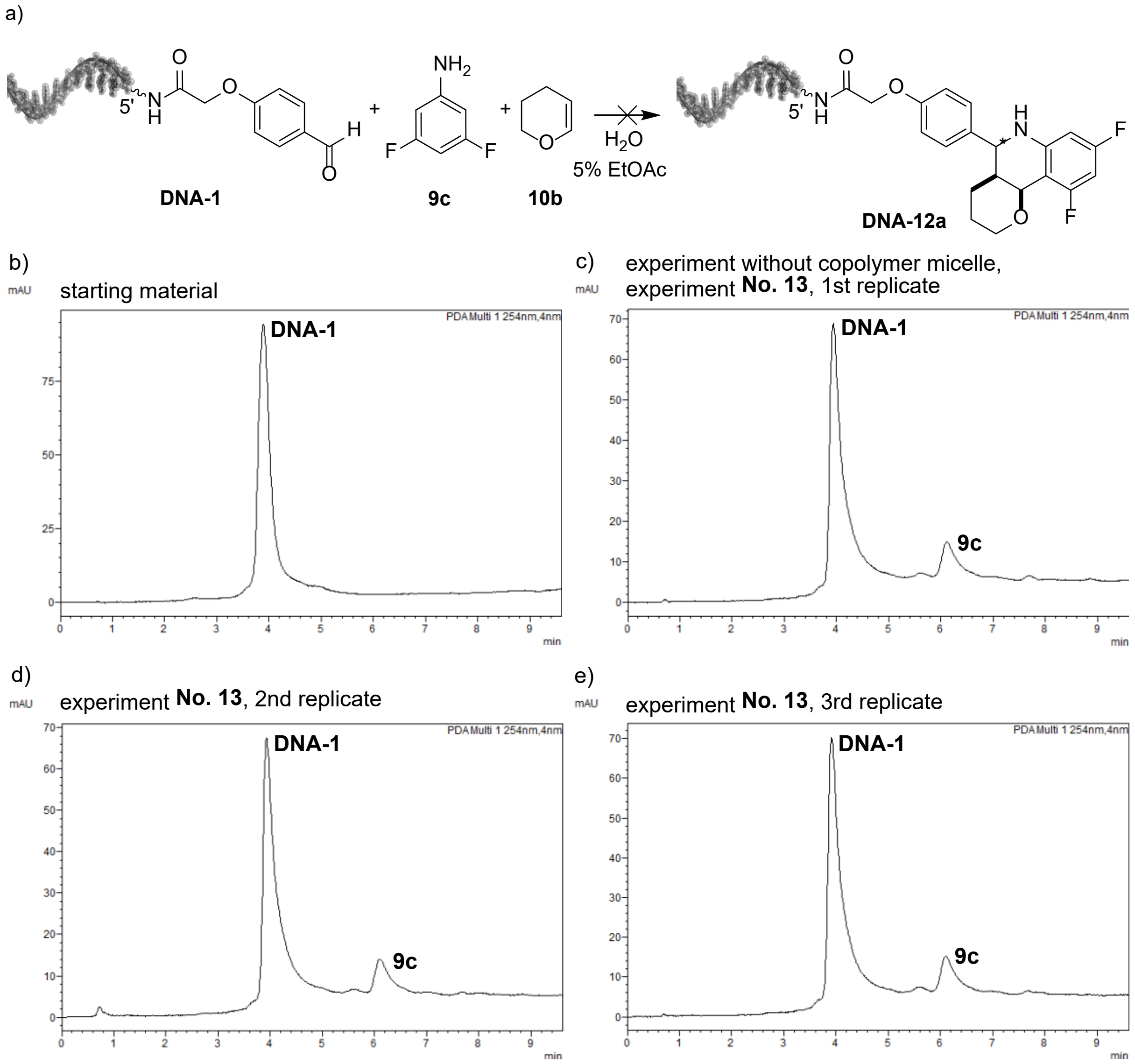

Figure S64. Povarov reaction of the oligonucleotide-aldehyde conjugate **DNA-1**, 3,5-difluoroaniline **9c**, and 3,4-dihydro-2*H*-pyran **10b** at 25 °C, in the absence of the copolymer micelle. a) Reaction scheme of the synthesis of **DNA-12a**; for reaction conditions see Table S15. HPLC traces show: b) oligonucleotide-aldehyde conjugate **DNA-1** and c)-e) experiment **No. 13** (Table S15) in triplicate.

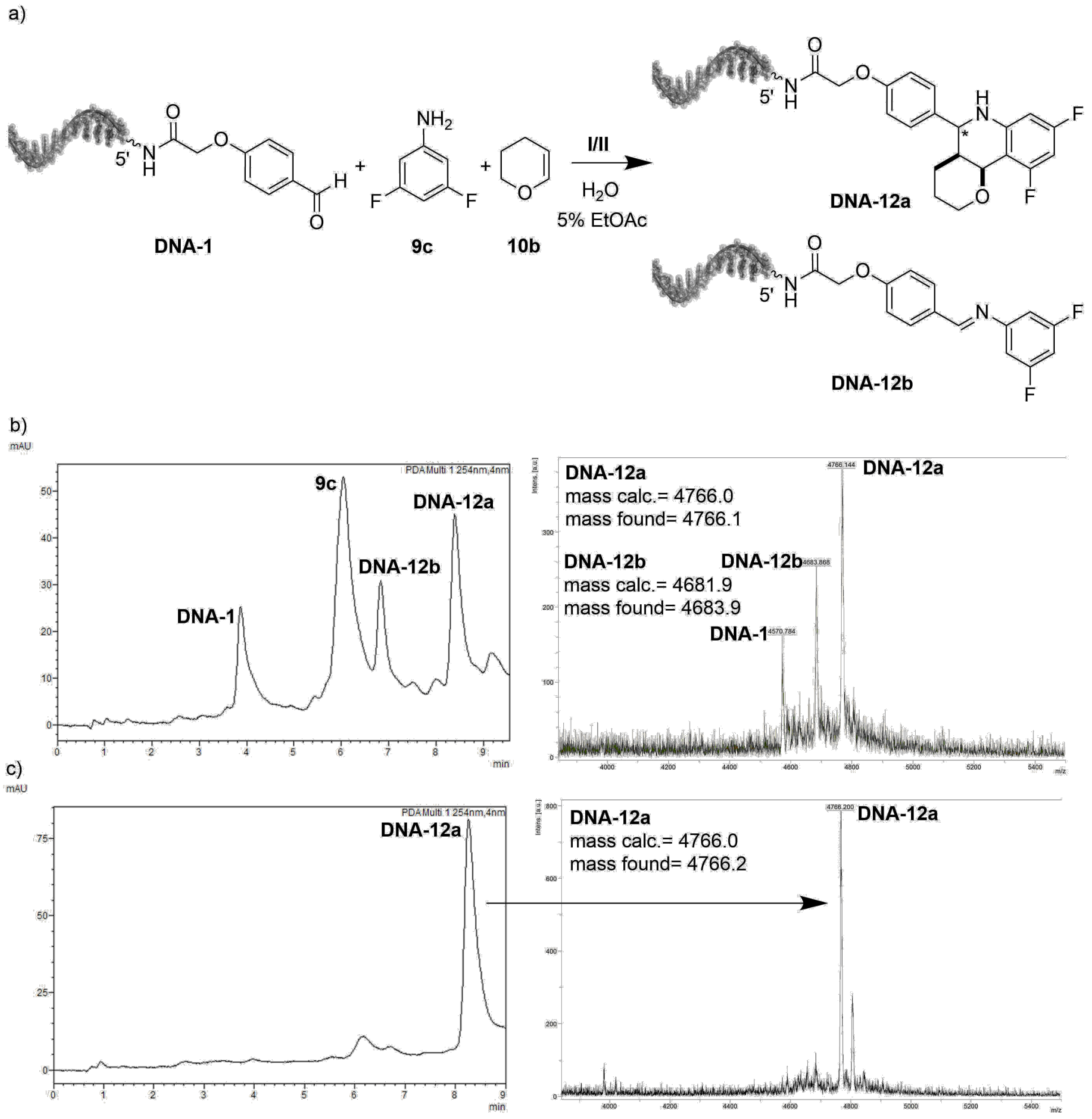

Figure S65. MALDI-MS analysis of the crude and of the isolated product **DNA-12a**. a) Reaction scheme of the synthesis of **DNA-12a** and **DNA-12b**; conditions: 50 eq. of copolymer **I/II** (0. 5 mM), 8000 eq. of reactant **9c** and 8000 eq. of reactant **10b**, 25 °C, 18 h. b) HPLC trace of the crude reaction mixture (left hand) from Fig. S59c (Table S16, **No. 8**, replicate 2x) and MALDI-MS spectrum of the corresponding reaction mixture (right hand). c) HPLC trace of the isolated product **DNA-12a** (left hand) and MALDI-MS spectrum of the isolated product **DNA-12a** (right hand).

4.10. Copolymer micelle **I/II**-mediated Povarov reaction of aldehyde conjugate **DNA-1**, *tert*-butylaniline **9a**, and 3,4-dihydro-2*H*-pyran **10b** to DNA-conjugate **DNA-12c**

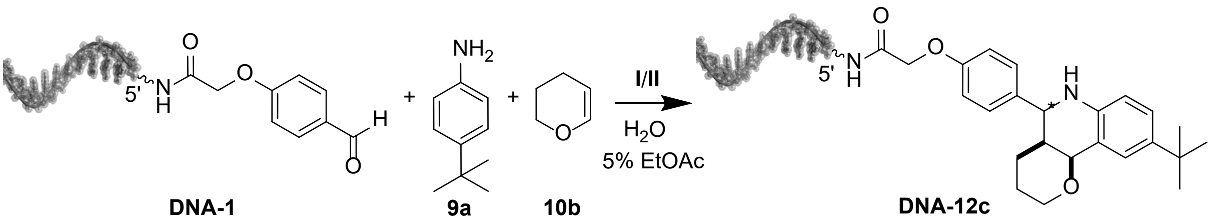

Scheme S9. Reaction scheme of the copolymer micelle **I/II**-mediated Povarov reaction to **DNA-12c**.

Table S16. Impact of copolymer micelle **I/II** design on synthesis of DNA-hexahydro-1*H*-pyrano[3,2-*c*]quinoline conjugate **DNA-12c**. Reaction conditions: 8000 eq. of **9a** and 8000 eq. of **10b**, 50 eq. of **I/II**, 0.5 mM **I/II**, room temperature, 18 hours.

No.	copolymer name	copolymer composition	**DNA-12c** 1x [%][a]	**DNA-12c** 2x [%][a]	**DNA-12c** 3x [%][a]	**DNA-12c** average [%][a]
1	**IA**	DMA_{62}-(MMA_{25}-SPA_2)	< 10	< 10	< 10	< 10
2	**IB**	DMA_{62}-(EA_{23}-SPA_2)	< 10	< 10	< 10	< 10
3	**IC$_a$**	DMA_{65}-(BA_{33}-SPA_2)	11	13	13	12
4	**IC$_b$**	DMA_{63}-(BA_{27}-SPA_2)	19	18	19	19
5	**IC$_c$**	DMA_{132}-(BA_{23}-SPA_2)	56	54	55	55
6	**ID**	DMA_{62}-(OA_{26}-SPA_2)	< 10	< 10	< 10	< 10
7	**IIA**	(DMA_{62}-SPA_2)-MMA_{14}	< 10	< 10	< 10	< 10
8	**IIB**	(DMA_{60}-SPA_2)-EA_{23}	< 10	< 10	< 10	< 10
9	**IIC$_a$**	(DMA_{63}-SPA_2)-BA_{33}	< 10	< 10	< 10	< 10
10	**IIC$_b$**	(DMA_{100}-SPA_2)-BA_{34}	44	41	44	43
11	**IID**	(DMA_{62}-SPA_2)-OA_{26}	< 10	< 10	< 10	< 10
12	**IIE**	(DMA_{62}-SPA_2)-DDA_{12}	< 10	< 10	< 10	< 10
13	-	-	0	0	0	0

[a] HPLC analysis of the crude, missing percentage to 100%: mainly **DNA-1**.

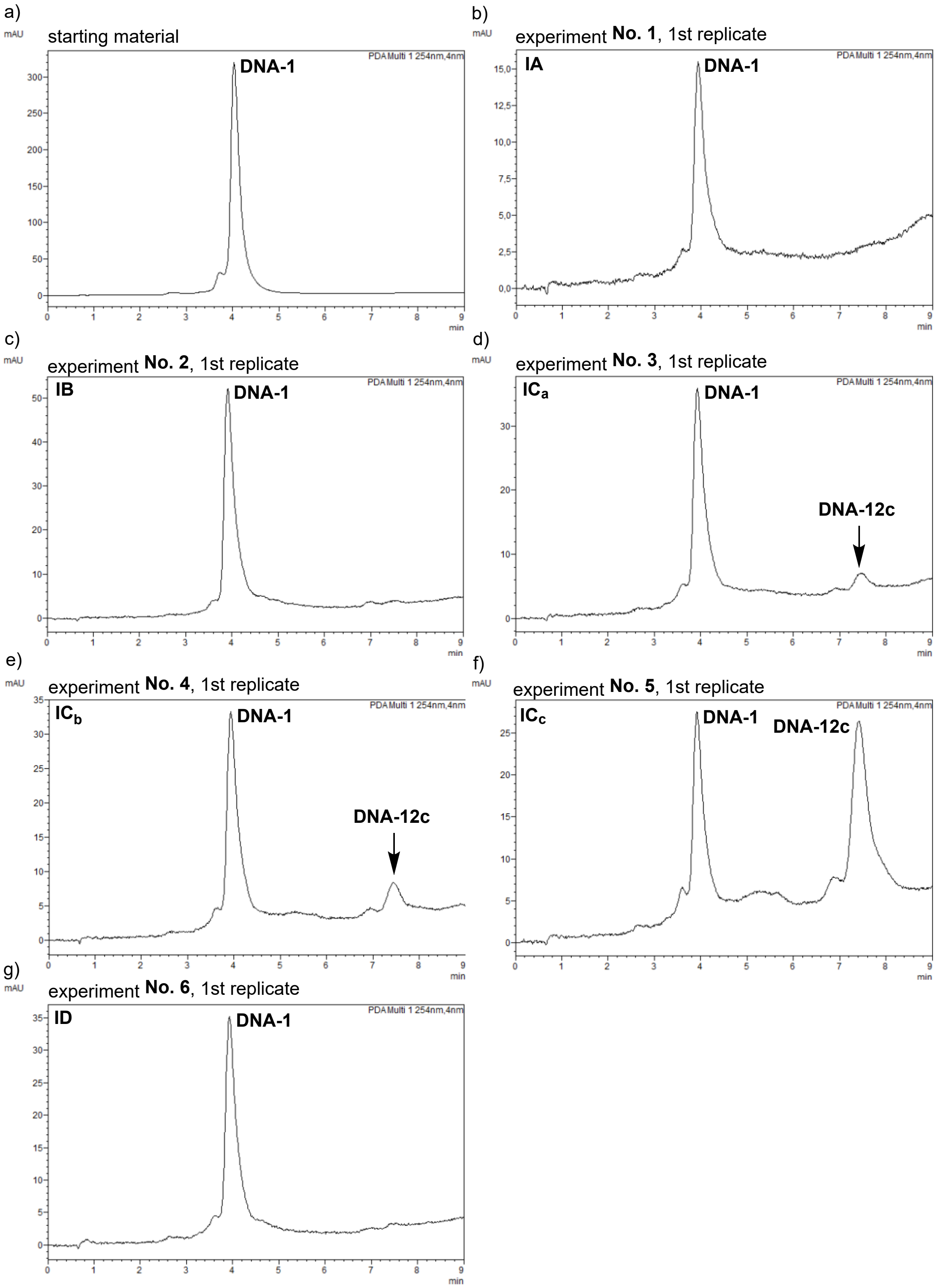

Figure S66. Impact of copolymer micelle **I**/**II** design on copolymer micelle **I**/**II**-mediated Povarov reaction of DNA-aldehyde conjugate **DNA-1**, *tert*-butylaniline **9a**, and 3,4-dihydro-2*H*-pyran **10b** at 25 °C, for reaction conditions see Table S16. HPLC traces show oligonucleotide-aldehyde conjugate **DNA-1** and experiments **No. 1** - **No. 6**.

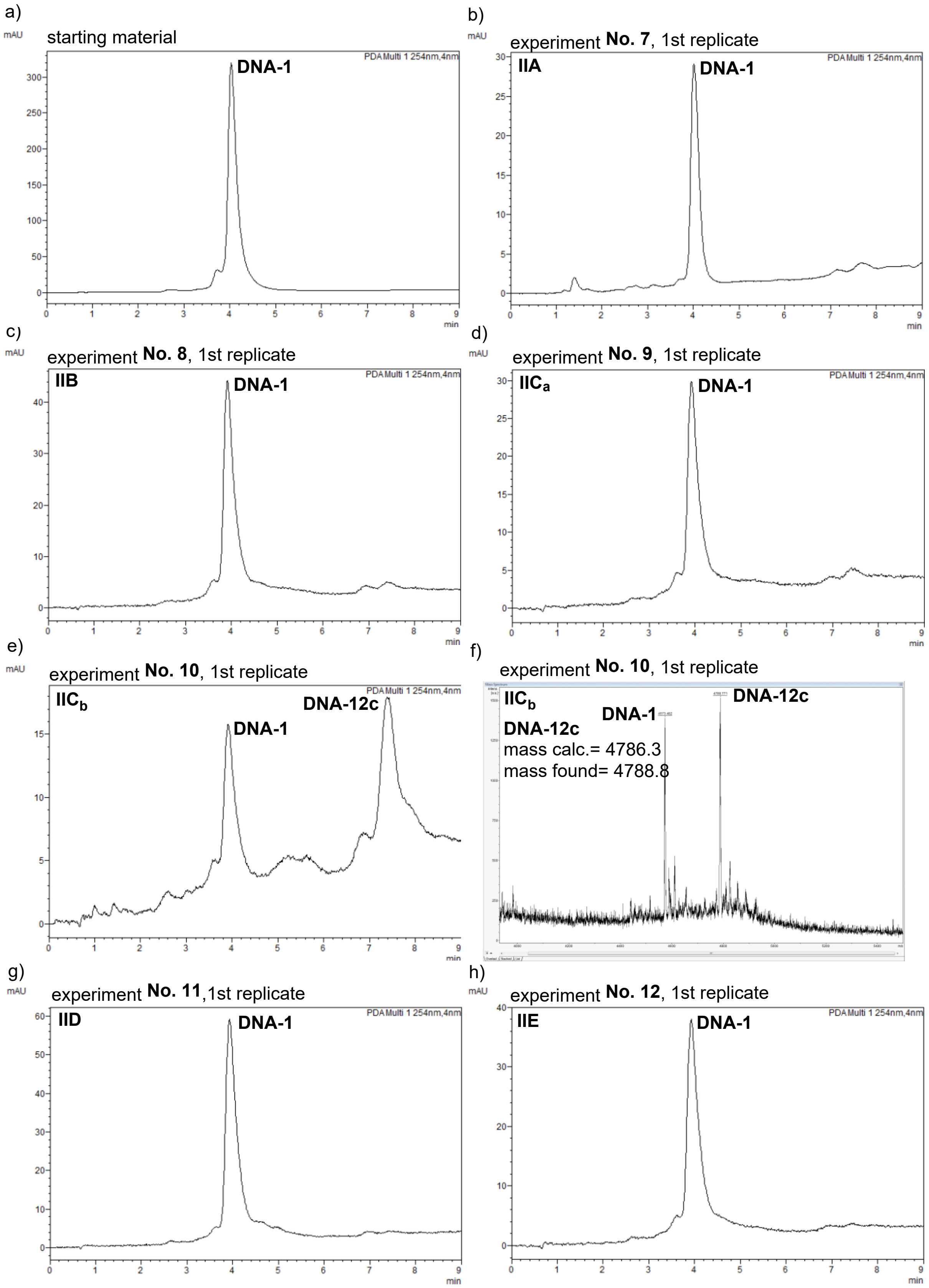

Figure S67. Impact of copolymer micelle **I**/**II** design on copolymer micelle **I**/**II**-mediated Povarov reaction of DNA-aldehyde conjugate **DNA-1**, *tert*-butylaniline **9a**, and 3,4-dihydro-2*H*-pyran **10b** at 25 °C, for reaction conditions see Table S16. HPLC traces show oligonucleotide-aldehyde conjugate **DNA-1** and experiments **No. 7** - **No. 12**. MALDI-MS spectrum shows experiment **No. 10**.

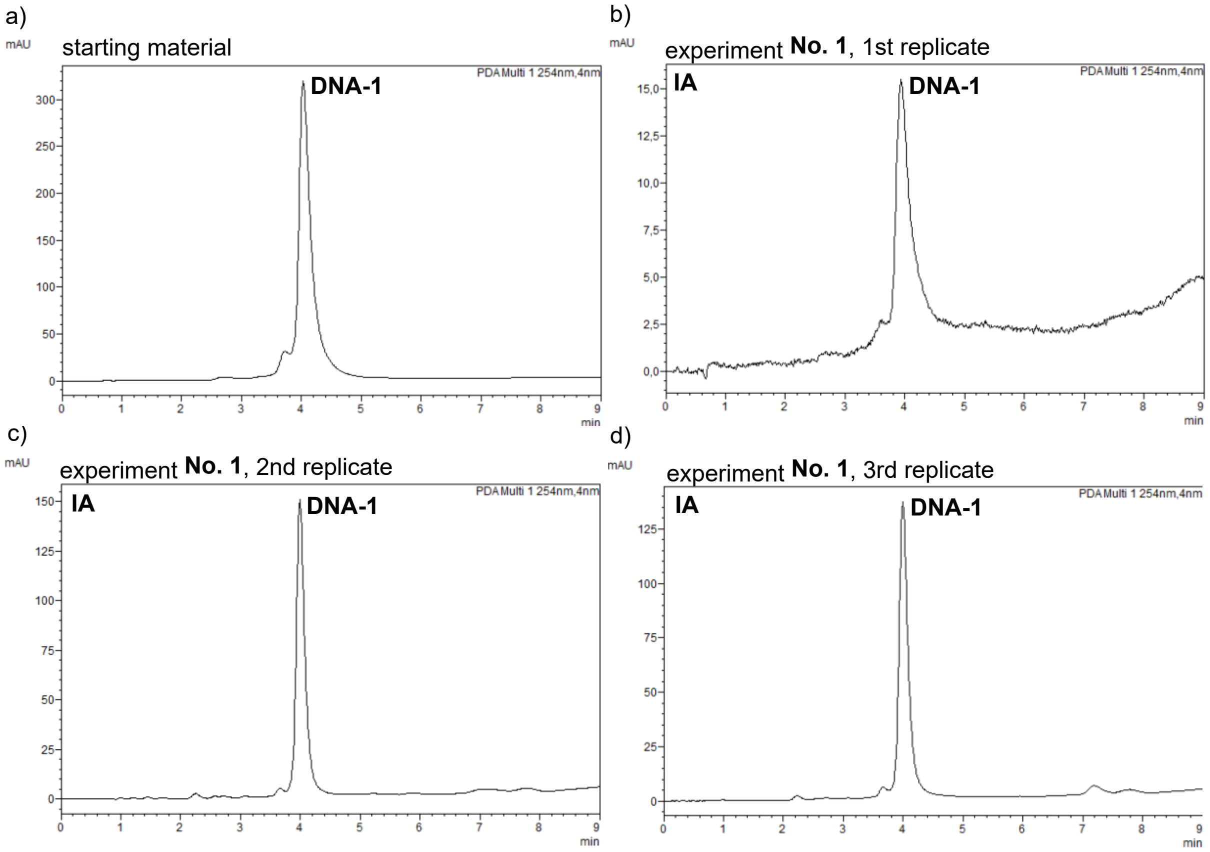

Figure S68. Copolymer **IA**-mediated Povarov reaction of the oligonucleotide-aldehyde conjugate **DNA-1**, aniline **9a**, and 3,4-dihydro-2*H*-pyran **10b** at 25 °C, for reaction conditions see Table S16. HPLC traces show: a) oligonucleotide-aldehyde conjugate **DNA-1** and b)-c) experiment **No. 1** in triplicate.

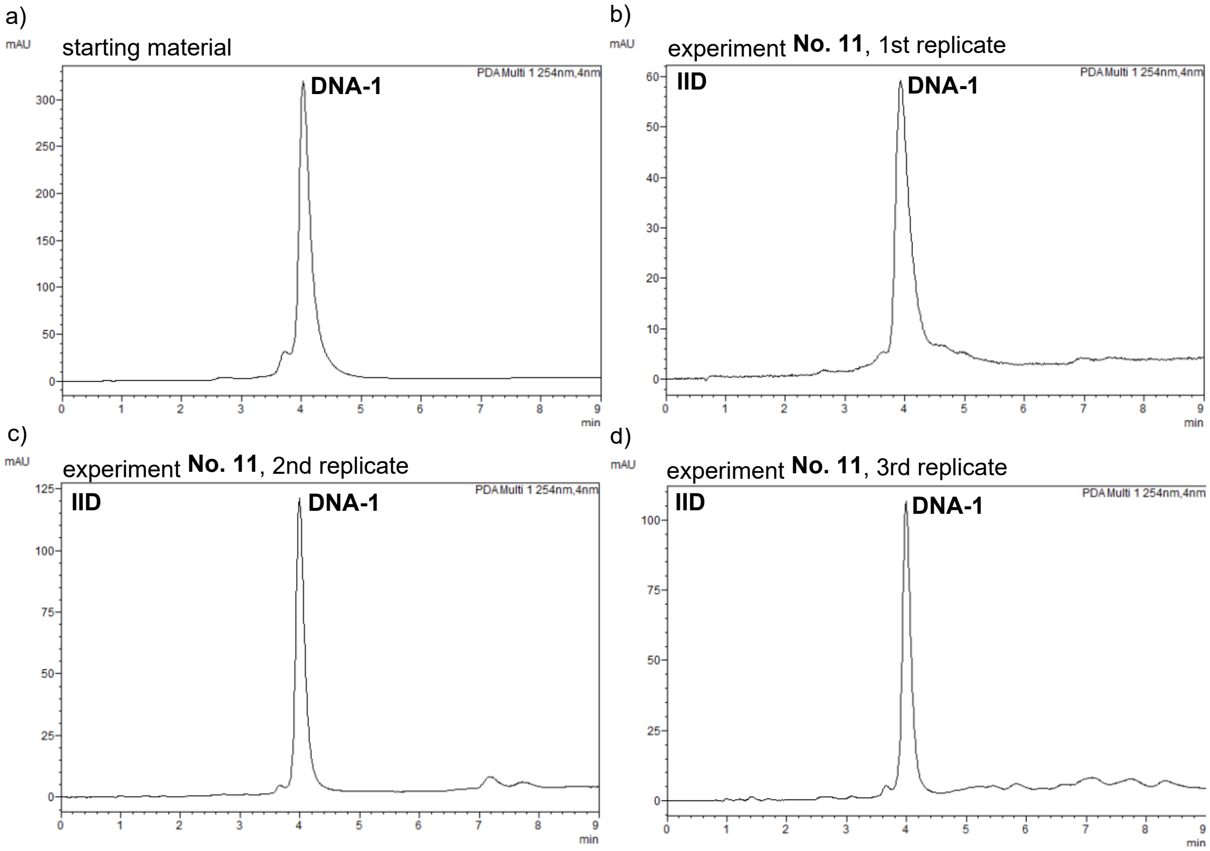

Figure S69. Copolymer **IID**-mediated Povarov reaction of the oligonucleotide-aldehyde conjugate **DNA-1**, aniline **9a**, and 3,4-dihydro-2*H*-pyran **10b** at 25 °C, for reaction conditions see Table S16. HPLC traces show: a) oligonucleotide-aldehyde conjugate **DNA-1** and b)-c) experiment **No. 11** in triplicate.

a)

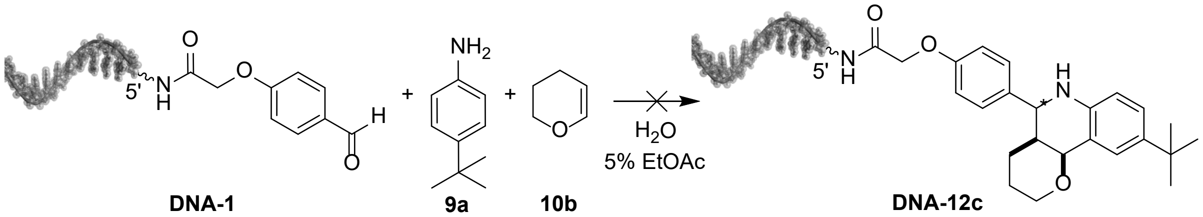

b)

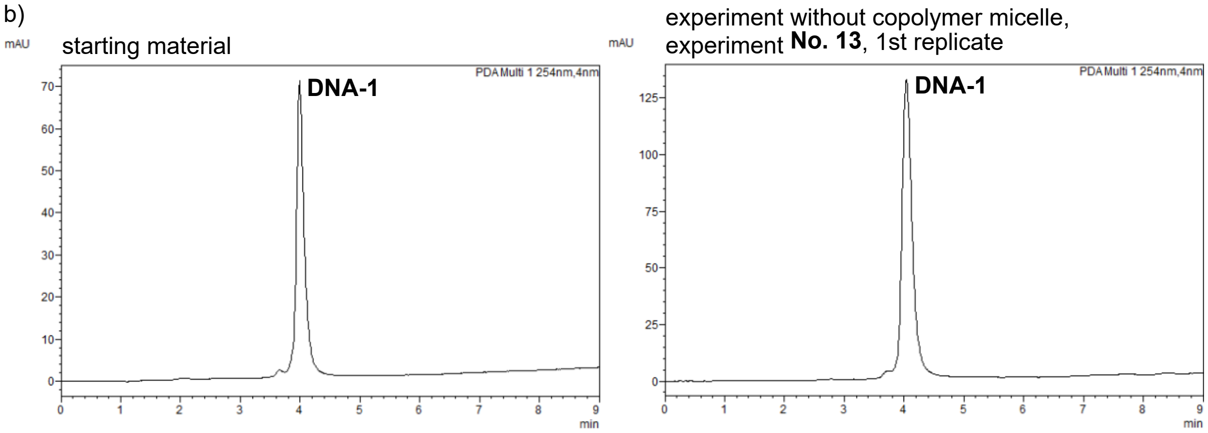

Figure S70. Povarov reaction of the oligonucleotide-aldehyde conjugate **DNA-1**, *tert*-butylaniline **9a**, and 3,4-dihydro-2*H*-pyran **10b** at 25 °C, in the absence of the copolymer micelle. a) Reaction scheme of the synthesis of **DNA-12c**; for reaction conditions see Table S16. b) HPLC traces show oligonucleotide-aldehyde conjugate **DNA-1** (left hand trace) and experiment **No. 13** (Table S16) (right hand trace).

4.11. Micellar Brønsted acid-mediated synthesis of DNA-hexahydro-1*H*-pyrrolo-[3,2-*c*]quinoline conjugates **DNA-11a** and **-18** in *aqueous* NaCl solution

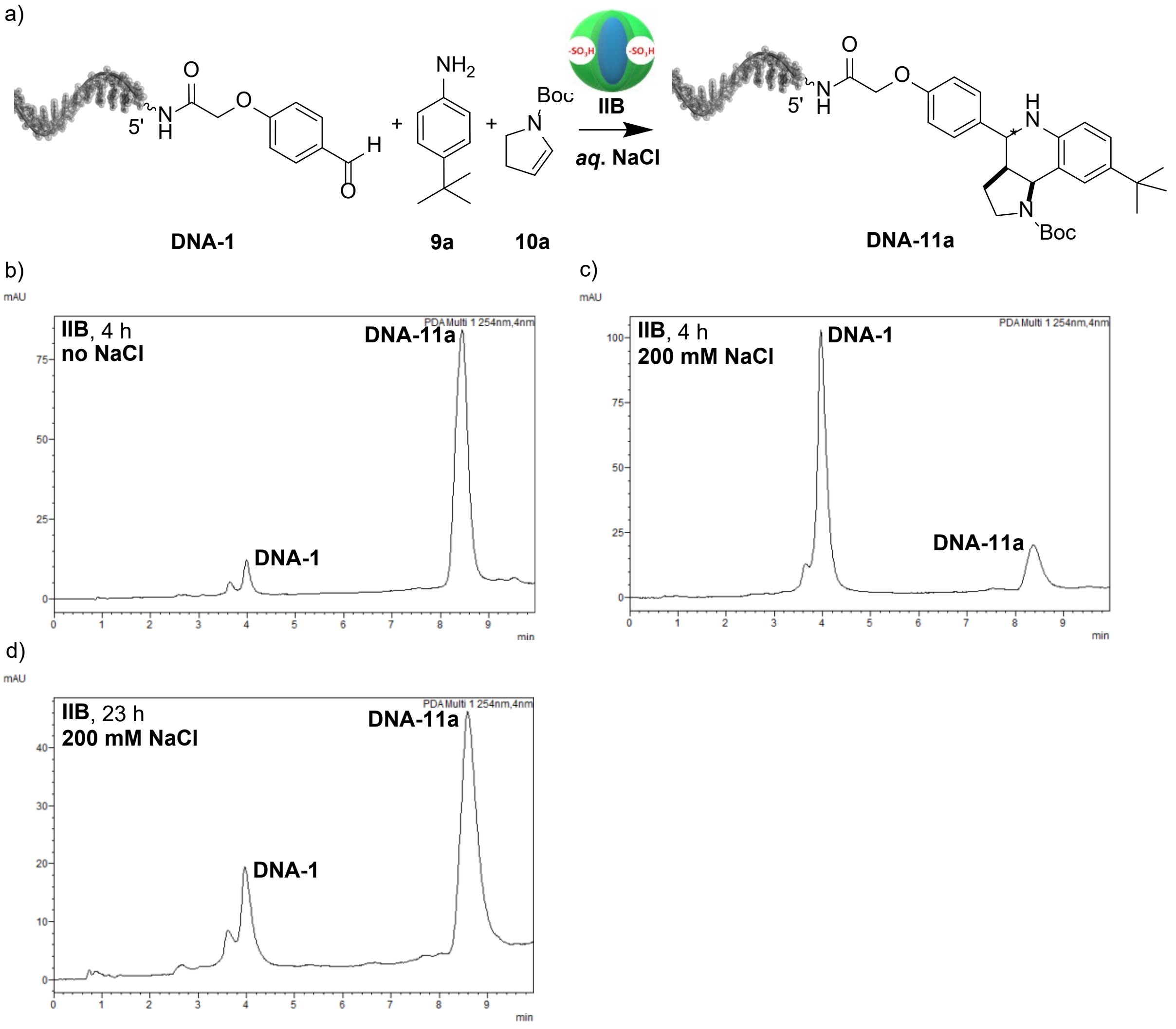

Figure S71. Effect of *aqueous* NaCl solution on copolymer micelle **IIB**-mediated Povarov reaction of the oligonucleotide-aldehyde conjugate **DNA-1**, 4-*tert*-butylaniline **9a**, and *N*-Boc-2,3-dihydro-1*H*-pyrrole **10a**. a) Reaction scheme for copolymer **IIB**-mediated synthesis of **DNA-11a** in an *aqueous* NaCl solution; b) HPLC trace of an experiment performed in water (5% EtOAc) for 4 hours (Table S8, **No. 32**); c) HPLC trace of an experiment performed in 200 mM NaCl solution for 4 hours; d) HPLC trace of an experiment performed in 200 mM NaCl solution for 23 hours.

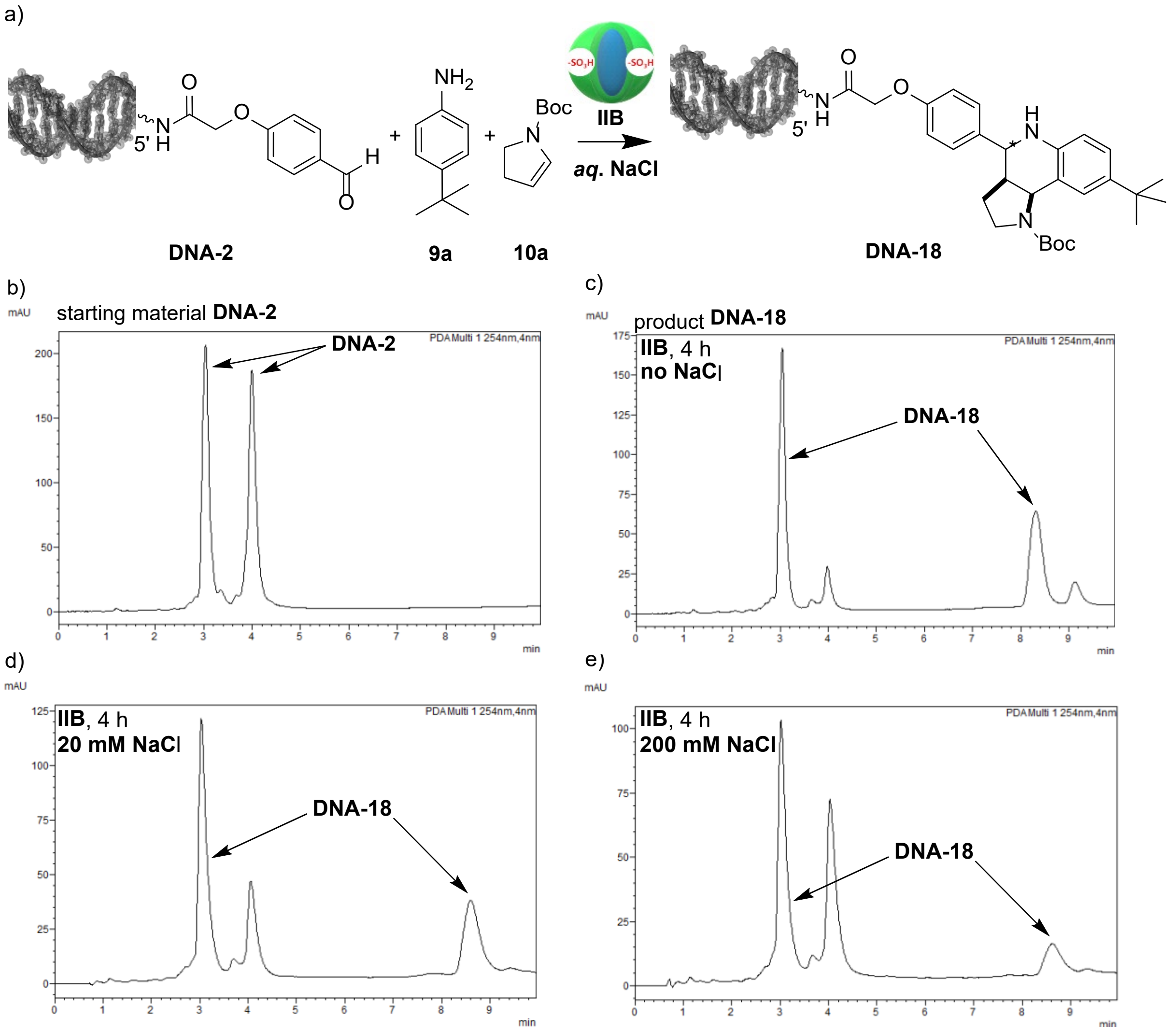

Figure S72. Effect of *aqueous* NaCl solution on copolymer micelle **IIB**-mediated Povarov reaction of the double stranded (ds) oligonucleotide-aldehyde conjugate **DNA-2**, 4-*tert*-butylaniline **9a**, and *N*-Boc-2,3-dihydro-1*H*-pyrrole **10a**. a) Reaction scheme for copolymer **IIB**-mediated synthesis of **DNA-18** in an *aqueous* NaCl solution; b) HPLC trace of starting material double stranded (ds) oligonucleotide-aldehyde conjugate **DNA-2**; c) HPLC trace of an experiment performed in water (5% EtOAc) for 4 hours; d) HPLC trace of an experiment performed in 20 mM NaCl solution for 4 hours; e) HPLC trace of an experiment performed in 200 mM NaCl solution for 4 hours.

5. Micellar Brønsted acid-mediated cleavage of the Boc-protective group from DNA-(Boc)glycine conjugate **DNA-13**

5.1. Synthesis of DNA-(Boc)glycine conjugate **DNA-13**

DNA strand (250 nmol, 5`-GTC TTG CCG AAT TC-3`) was coupled with (*tert*-butoxycarbonyl)glycine **23** (4.5 mg, 25 µmol, 100 eq.) to furnish **DNA-13** according to the procedure for coupling of carboxylic acids to amino-modified DNA.

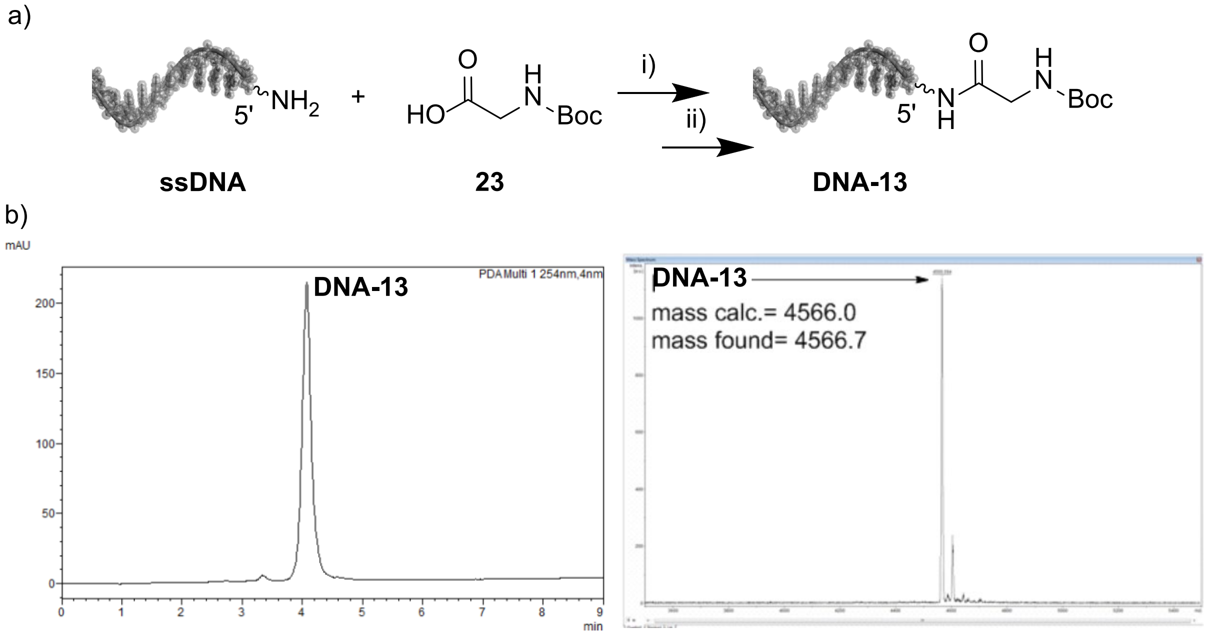

Figure S73. Synthesis of the DNA-(Boc)glycine conjugate **DNA-13**. a) Scheme of the synthesis of **DNA-13**. b) HPLC trace of the DNA-(Boc)glycine conjugate **DNA-13** (left hand trace) and MALDI-MS spectrum of the DNA-(Boc)glycine conjugate **DNA-13** (right hand spectrum). Reagents and conditions: i) HATU, DIPEA, dry DMF, room temperature, 4 h; ii) AMA (aqueous ammonia (30%)/ aqueous methylamine (40%), 1:1, vol/vol), 4 h, room temperature. ss= single stranded.

5.2. Copolymer micelle **I/II**-mediated cleavage of Boc protective group from DNA-(Boc)glycine conjugate **DNA-13**

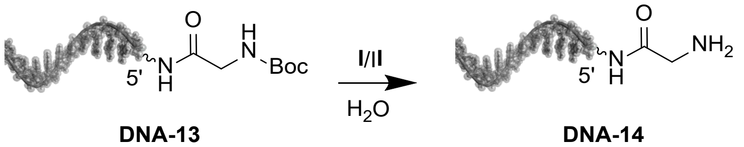

Scheme S10. Copolymer micelle **I/II**-mediated cleavage of Boc protective group from DNA-(Boc)glycine conjugate **DNA-13**.

Protocol:[3] To a solution of single stranded (ss) DNA-(Boc)glycine conjugate **DNA-13** (500 pmol) in distilled water was added copolymer micelle **I/II** (12.5 nmol, 25 eq.) dissolved in 8 µL of distilled water taken from an aqueous stock solution (1.25 µmol in 800 µL). The reaction mixtures were filled with distilled water to a volume of 50 µL giving a final concentration of 0.25 mM of copolymer **I/II**. The reaction mixtures were shaken at 50 °C at reaction times given in Table S17. Then, 70 µL of distilled water were added and the reaction mixtures were extracted with ethyl acetate (6 x 200 µL). The aqueous solution was evaporated in a SpeedVac, the residue was redissolved in 45 µL of distilled water, and analyzed by RP-HPLC (Phenomenex, Gemini; 5 µm, C18, 110 Å, 100*4.6 mm) with a gradient of aqueous triethylammonium acetate buffer (10 mM, pH= 8) and methanol (10% - 60% of methanol over 9 min), and by MALDI-MS to analyze product formation rate and product identity.

Table S17. Impact of copolymer micelle **I/II** design on cleavage of Boc protective group from DNA-(Boc)glycine conjugate **DNA-13**. Reaction conditions: 25 eq. of **I/II**, 0.25 mM **I/II**, 50 °C.

No.	copolymer name	copolymer composition	*t* [h]	**DNA-14**				
				(1x) [%][a]	(2x) [%][a]	(3x) [%][a]	average [%][a]	average depurination [%][a]
1	**IA**	DMA_{62}-(MMA_{25}-SPA_2)	4	35	45	43	41	22
2	**IA**	DMA_{62}-(MMA_{25}-SPA_2)	8	62	67	63	64	49
3	**IB**	DMA_{62}-(EA_{23}-SPA_2)	4	37	36	35	36	21
4	**IB**	DMA_{62}-(EA_{23}-SPA_2)	8	56	56	56	56	40
5	**IC$_a$**	DMA_{65}-(BA_{33}-SPA_2)	4	25	26	33	28	14
6	**IC$_a$**	DMA_{65}-(BA_{33}-SPA_2)	8	48	51	54	51	31
7	**IC$_b$**	DMA_{63}-(BA_{27}-SPA_2)	4	44	44	42	43	24
8	**IC$_b$**	DMA_{63}-(BA_{27}-SPA_2)	8	64	64	64	64	46
9	**IC$_c$**	DMA_{132}-(BA_{23}-SPA_2)	4	47	47	47	47	26
10	**IC$_c$**	DMA_{132}-(BA_{23}-SPA_2)	8	68	70	71	70	50
11	**ID**	DMA_{62}-(OA_{26}-SPA_2)	4	25	29	16	23	12
12	**ID**	DMA_{62}-(OA_{26}-SPA_2)	8	45	46	37	43	26
13	**IIA**	(DMA_{62}-SPA_2)-MMA_{14}	4	45	42	44	44	24
14	**IIA**	(DMA_{62}-SPA_2)-MMA_{14}	8	75	69	71	72	65
15	**IIB**	(DMA_{60}-SPA_2)-EA_{23}	4	40	39	42	40	22
16	**IIB**	(DMA_{60}-SPA_2)-EA_{23}	8	63	63	60	62	42
17	**IIC$_a$**	(DMA_{63}-SPA_2)-BA_{33}	4	33	33	33	33	17
18	**IIC$_a$**	(DMA_{63}-SPA_2)-BA_{33}	8	55	61	63	60	41
19	**IIC$_b$**	(DMA_{100}-SPA_2)-BA_{34}	4	57	55	57	56	44
20	**IIC$_b$**	(DMA_{100}-SPA_2)-BA_{34}	8	88	84	87	86	96
21	**IID**	(DMA_{62}-SPA_2)-OA_{26}	4	29	26	30	28	15
22	**IID**	(DMA_{62}-SPA_2)-OA_{26}	8	50	49	51	50	30
23	**IIE**	(DMA_{62}-SPA_2)-DDA_{12}	4	24	21	26	24	11
24	**IIE**	(DMA_{62}-SPA_2)-DDA_{12}	8	44	42	46	44	23
25	**III**	DMA_{72}-BA_{13}	4	0	-	-	-	0
26	**III**	DMA_{72}-BA_{13}	8	< 5	-	-	-	< 5

[a] HPLC and MALDI-MS analysis of the crude, conversions in parentheses show DNA degradation.

Table S18. Impact of copolymer micelle **I/II** design on DNA depurination during cleavage of Boc protective group from DNA-(Boc)glycine conjugate **DNA-13**: ranking micelles **I/II** from the lowest to the highest level of DNA depurination they produce. Reaction conditions: 25 eq. of **I/II**, 0.25 mM **I/II**, 50 °C.

	DNA depurination ranking (depurination range)[a]	copolymer name	copolymer composition
4 h	**1** (0-15 %)	**IIE**	$(DMA_{62}\text{-}SPA_{2})\text{-}DDA_{12}$
		**ID**	$DMA_{62}\text{-}(OA_{26}\text{-}SPA_{2})$
		**IC$_a$**	$DMA_{65}\text{-}(BA_{33}\text{-}SPA_{2})$
		**IID**	$(DMA_{62}\text{-}SPA_{2})\text{-}OA_{26}$
	**2** (16-20 %)	**IIC$_a$**	$(DMA_{63}\text{-}SPA_{2})\text{-}BA_{33}$
	**3** (21-25 %)	**IB**	$DMA_{62}\text{-}(EA_{23}\text{-}SPA_{2})$
		**IIB**	$(DMA_{60}\text{-}SPA_{2})\text{-}EA_{23}$
		**IA**	$DMA_{62}\text{-}(MMA_{25}\text{-}SPA_{2})$
		**IC$_b$**	$DMA_{63}\text{-}(BA_{27}\text{-}SPA_{2})$
		**IIA**	$(DMA_{62}\text{-}SPA_{2})\text{-}MMA_{14}$
	**4** (26-30 %)	**IC$_c$**	$DMA_{132}\text{-}(BA_{23}\text{-}SPA_{2})$
	**5** (41-45 %)	**IIC$_b$**	$(DMA_{100}\text{-}SPA_{2})\text{-}BA_{34}$
8 h	**1** (21-25 %)	**IIE**	$(DMA_{62}\text{-}SPA_{2})\text{-}DDA_{12}$
	**2** (26-30 %)	**ID**	$DMA_{62}\text{-}(OA_{26}\text{-}SPA_{2})$
		**IID**	$(DMA_{62}\text{-}SPA_{2})\text{-}OA_{26}$
	**3** (31-35 %)	**IC$_a$**	$DMA_{65}\text{-}(BA_{33}\text{-}SPA_{2})$
	**4** (36-40 %)	**IB**	$DMA_{62}\text{-}(EA_{23}\text{-}SPA_{2})$
	**5** (41-45 %)	**IIC$_a$**	$(DMA_{63}\text{-}SPA_{2})\text{-}BA_{33}$
		**IIB**	$(DMA_{60}\text{-}SPA_{2})\text{-}EA_{23}$
	**6** (46-50 %)	**IC$_b$**	$DMA_{63}\text{-}(BA_{27}\text{-}SPA_{2})$
		**IA**	$DMA_{62}\text{-}(MMA_{25}\text{-}SPA_{2})$
		**IC$_c$**	$DMA_{132}\text{-}(BA_{23}\text{-}SPA_{2})$
	**7** (61-65 %)	**IIA**	$(DMA_{62}\text{-}SPA_{2})\text{-}MMA_{14}$
	**8** (96-100 %)	**IIC$_b$**	$(DMA_{100}\text{-}SPA_{2})\text{-}BA_{34}$

[a] Based on the data from Table S17.

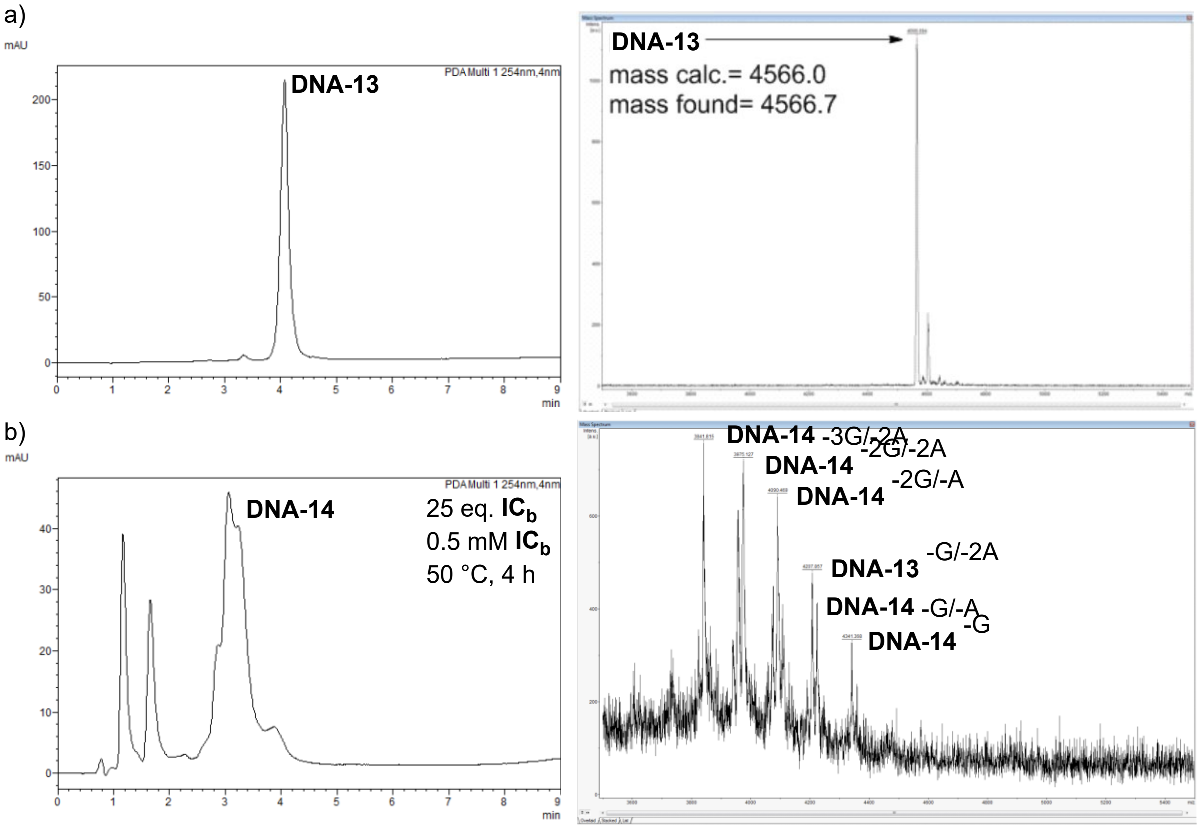

Figure S74. Comparison of analytical data of intact and depurinated DNA. a) HPLC trace and MALDI-MS spectrum of intact DNA-(Boc)glycine conjugate **DNA-13** compared to b) copolymer **IC$_b$**-mediated cleavage of Boc protective group from DNA-(Boc)glycine conjugate **DNA-13**. HPLC trace and MALDI-MS spectrum show depurinated crude product **DNA-14**. Reaction conditions: 25 eq. of copolymer **IC$_b$** (0.5 mM), 50 °C, 4 hours.

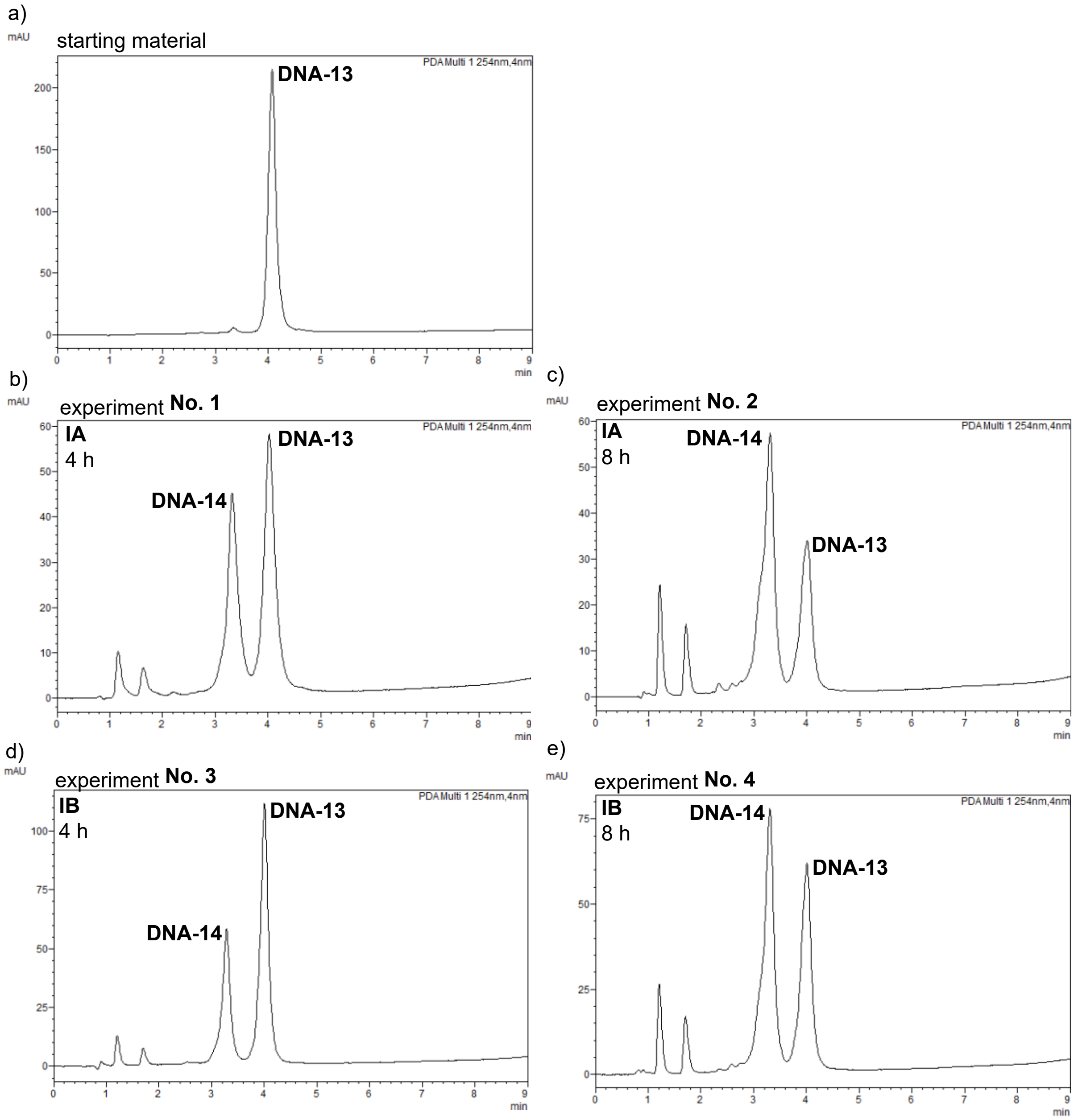

Figure S75. Kinetics of the copolymer **IA** (experiment 3x) and **IB** (experiment 3x)-mediated cleavage of Boc protective group from DNA-(Boc)glycine conjugate **DNA-13**, for reaction conditions see Table S17. HPLC traces show starting material **DNA-13** and experiments **No. 1** - **No. 4**.

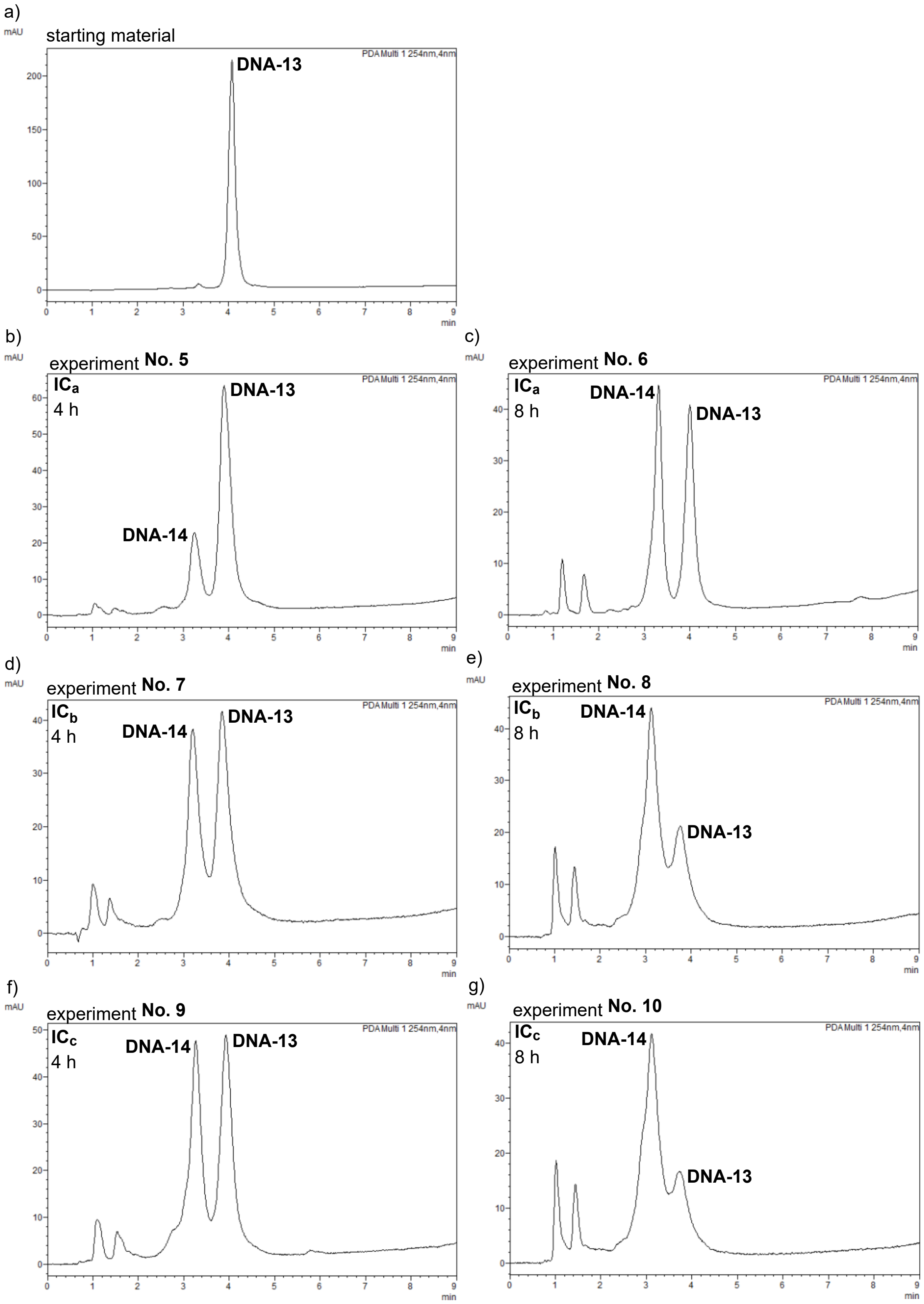

Figure S76. Kinetics of the copolymer **IC$_a$** (experiment 2x), **IC$_b$** (experiment 2x), and **IC$_c$** (experiment 2x)-mediated cleavage of Boc protective group from DNA-(Boc)glycine conjugate **DNA-13**, for reaction conditions see Table S17. HPLC traces show starting material **DNA-13** and experiments **No. 5** - **No. 10**.

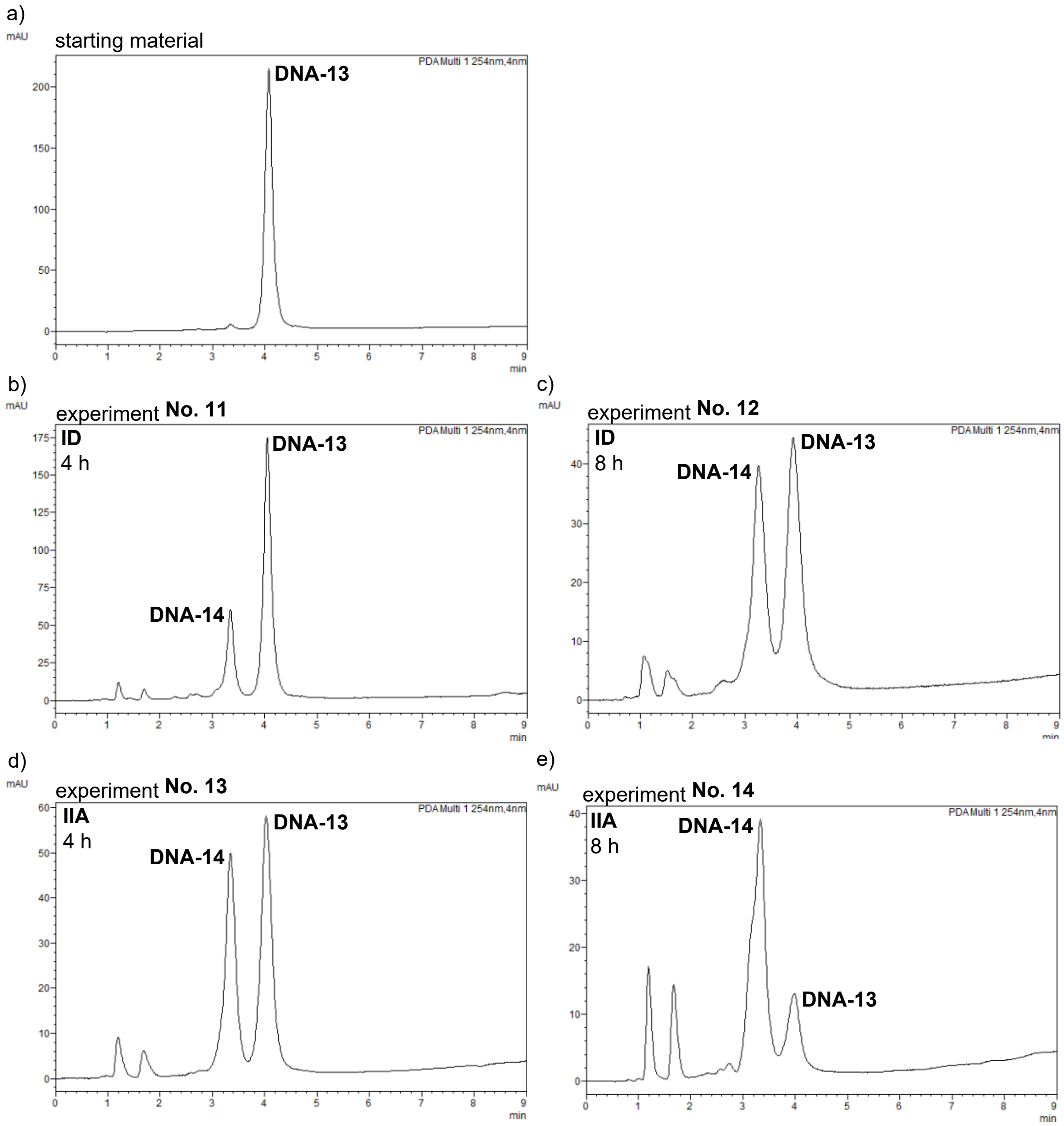

Figure S77. Kinetics of the copolymer **ID** (experiment 1x) and **IIA** (experiment 1x)-mediated cleavage of Boc protective group from DNA-(Boc)glycine conjugate **DNA-13**, for reaction conditions see Table S17. HPLC traces show starting material **DNA-13** and experiments **No. 11** - **No. 14**.

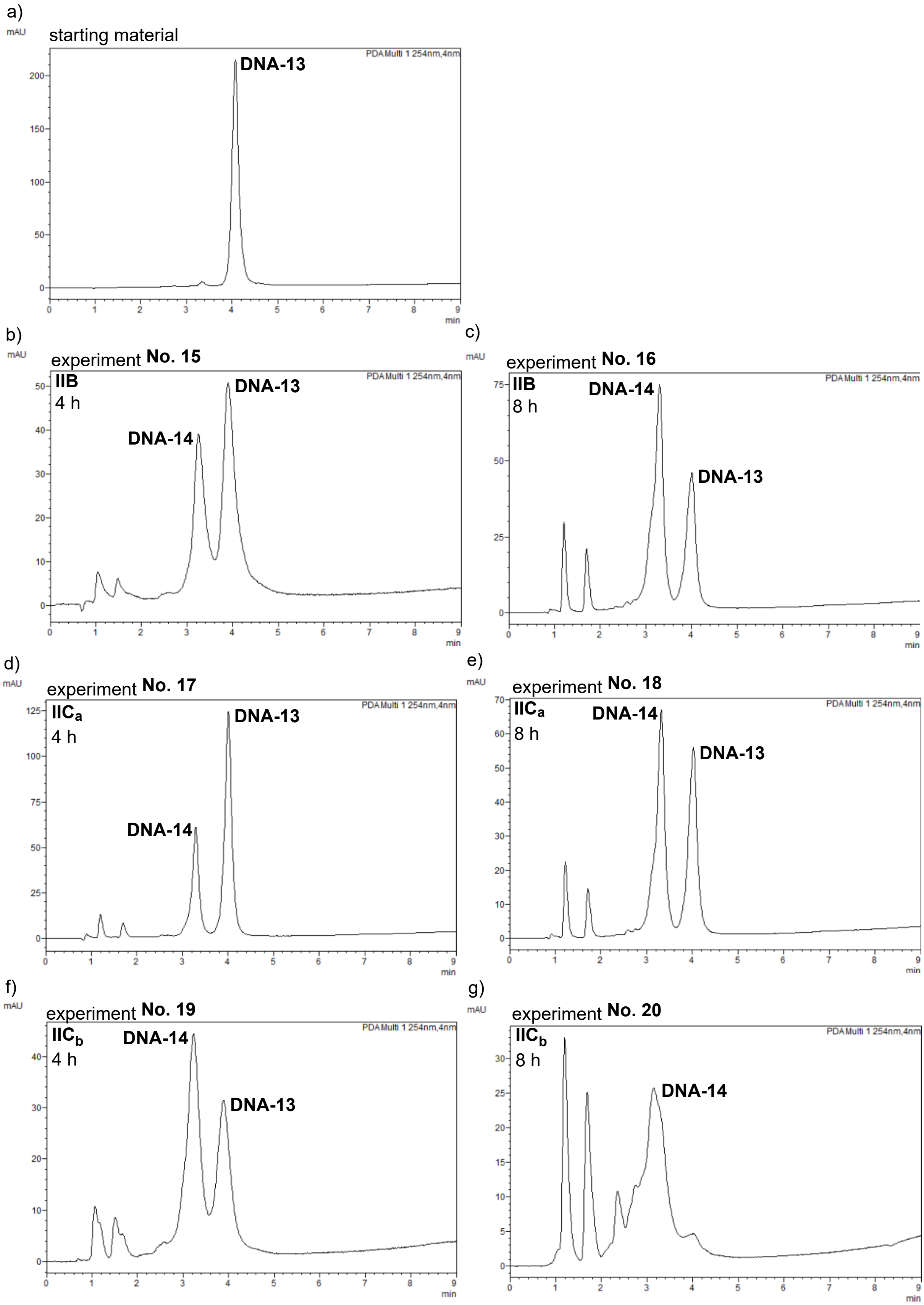

Figure S78. Kinetics of the copolymer **IIB** (experiment 1x), **IIC$_a$** (experiment 1x), and **IIC$_b$** (experiment 1x)-mediated cleavage of Boc protective group from DNA-(Boc)glycine conjugate **DNA-13**, for reaction conditions see Table S17. HPLC traces show starting material **DNA-13** and experiments **No. 15** - **No. 20**.

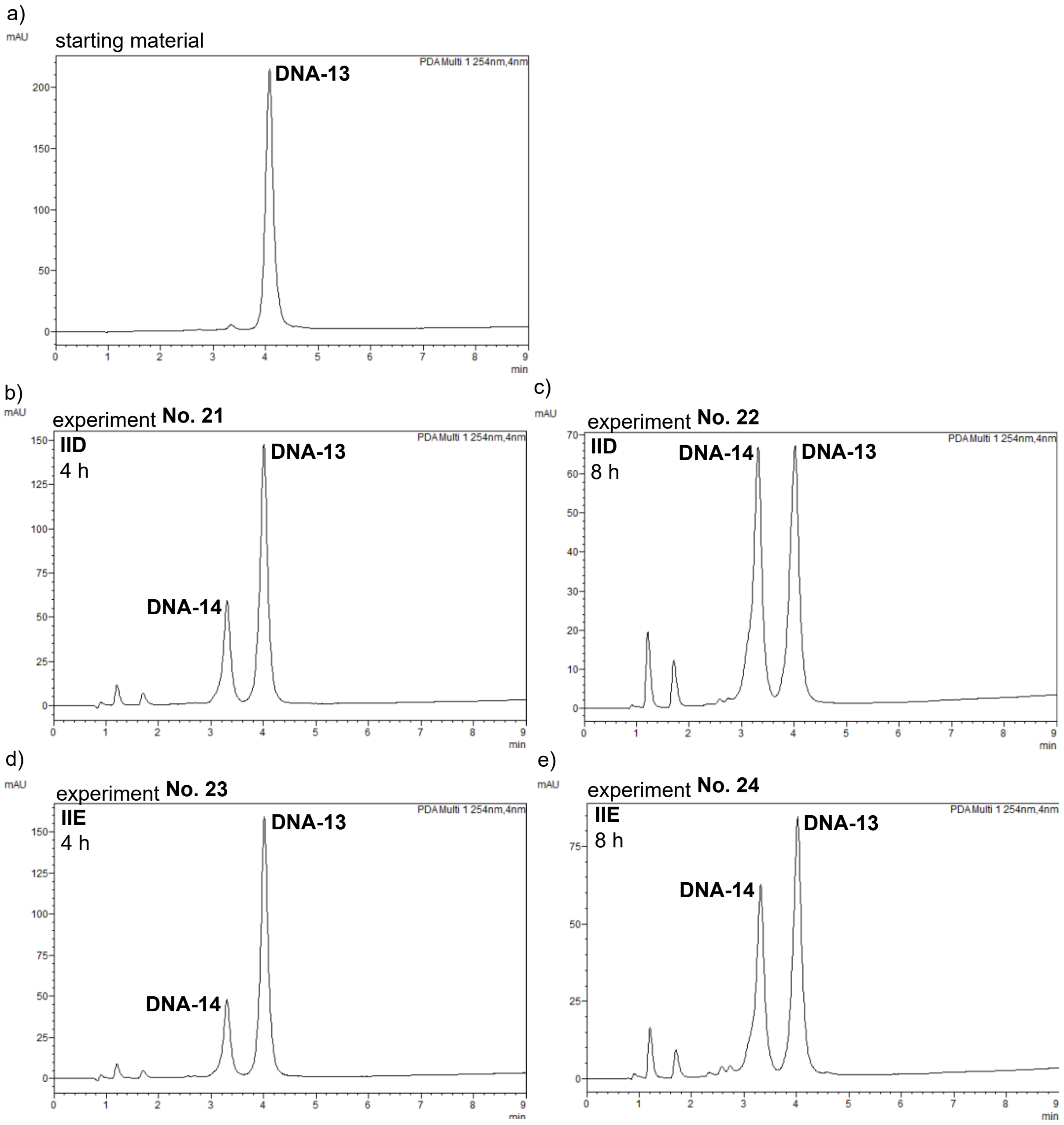

Figure S79. Kinetics of the copolymer **IID** (experiment 1x) and **IIE** (experiment 1x)-mediated cleavage of Boc protective group from DNA-(Boc)glycine conjugate **DNA-13**, for reaction conditions see Table S17. HPLC traces show starting material **DNA-13** and experiments **No. 21** - **No. 24**.

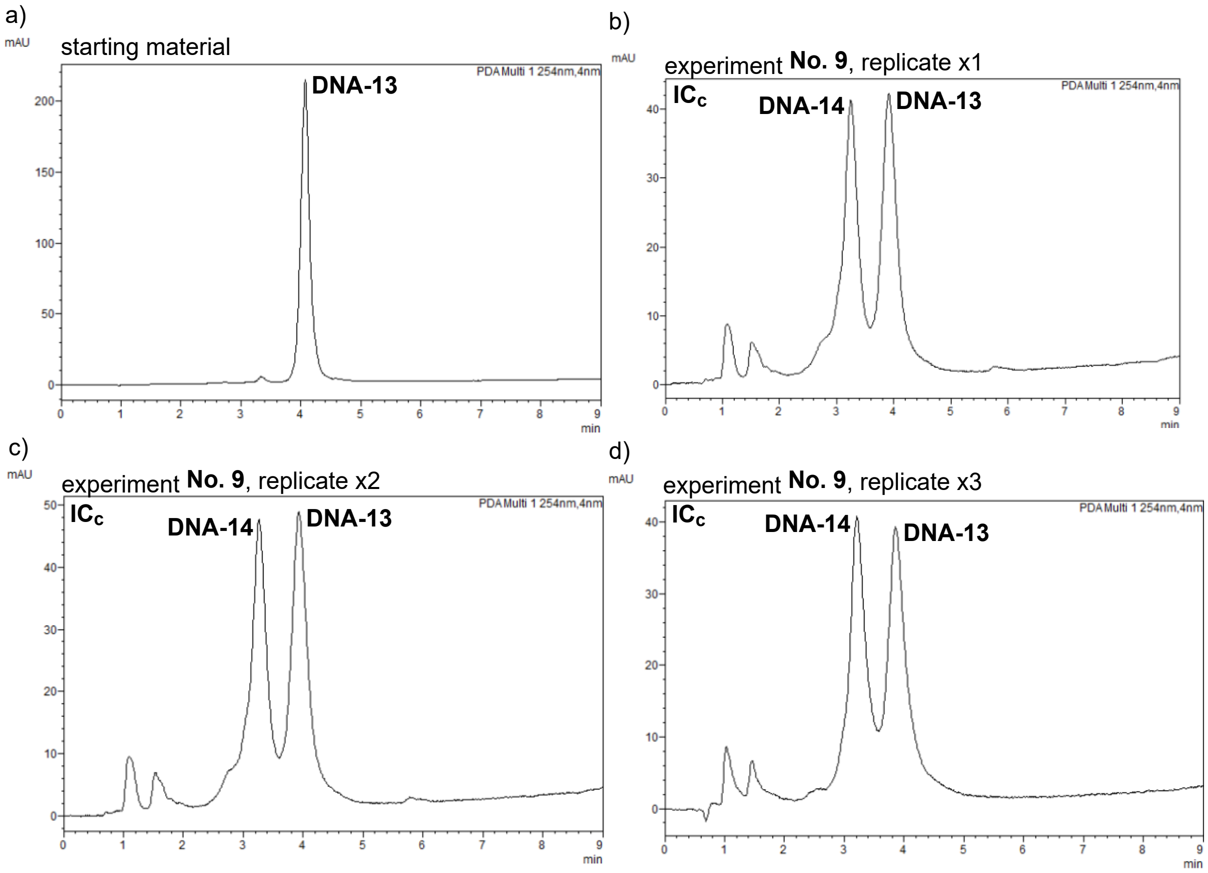

Figure S80. Copolymer **IC$_c$**-mediated cleavage of Boc protective group from DNA-(Boc)glycine conjugate **DNA-13**, for reaction conditions see Table S17. HPLC traces show: a) DNA-(Boc)glycine conjugate **DNA-13** and b)-d) experiment **No. 9** in triplicate.

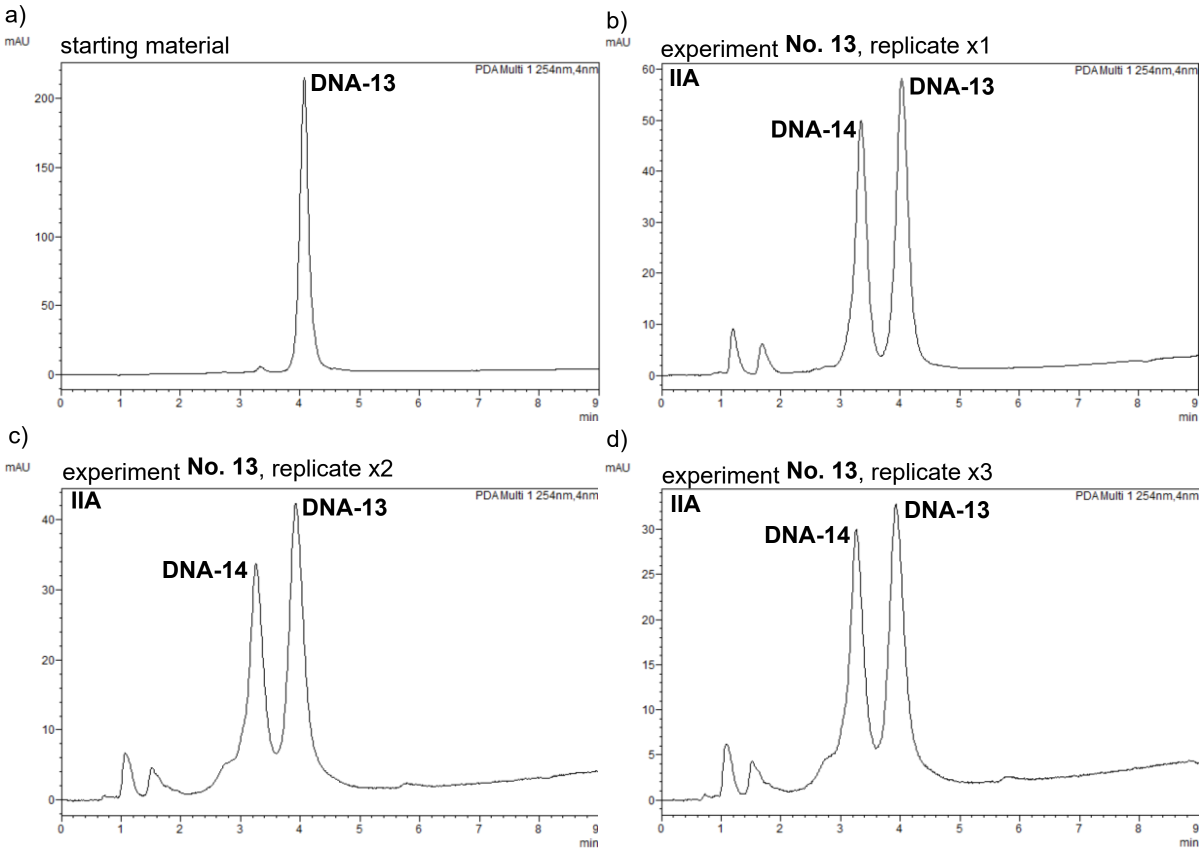

Figure S81. Copolymer **IIA**-mediated cleavage of Boc protective group from DNA-(Boc)glycine conjugate **DNA-13**, for reaction conditions see Table S17. HPLC traces show: a) DNA-(Boc)glycine conjugate **DNA-13** and b)-d) experiment **No. 13** in triplicate.

5.3. Exploring sulfonic acid un-substituted copolymer micelle **III** for cleavage of Boc protective group from DNA-(Boc)glycine conjugate **DNA-13**

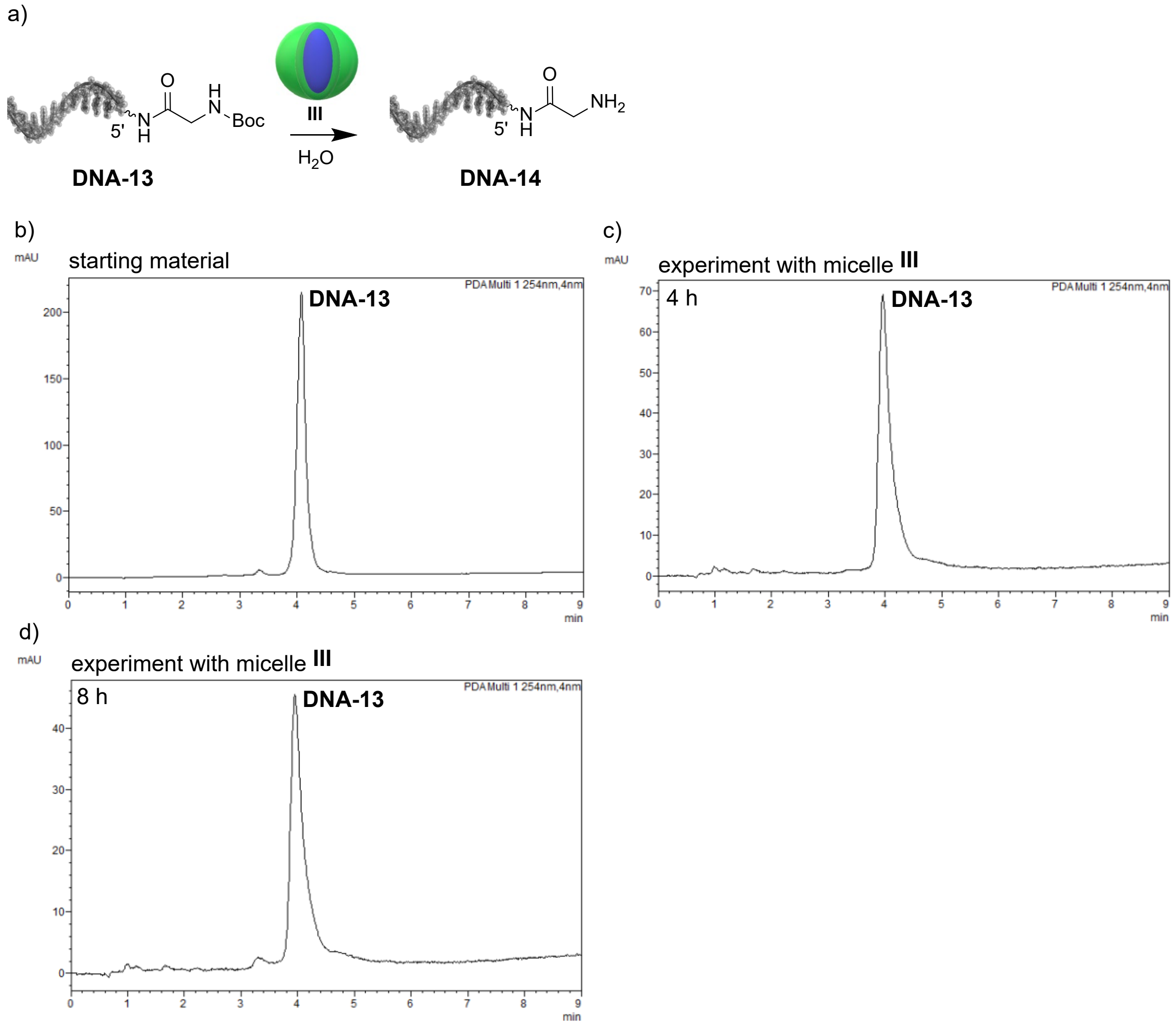

Figure S82. Exploring sulfonic acid un-substituted copolymer micelle **III** for cleavage of Boc protective group from DNA-(Boc)glycine conjugate **DNA-13**. a) Reaction scheme for copolymer **III**-mediated cleavage of Boc protective group from DNA-(Boc)glycine conjugate **DNA-13**; b) HPLC trace of oligonucleotide-aldehyde conjugate **DNA-1**; c) HPLC trace of experiment **No. 25** (Table S17); d) HPLC trace of experiment **No. 26** (Table S17).

5.4. Buffering the copolymer micelle **IC$_b$**-mediated cleavage of Boc protective group from DNA-(Boc)glycine conjugate **DNA-13** by addition of *tert*-butylaniline **9a** as buffering component

Table S19. Addition of *tert*-butylaniline **9a** to copolymer micelle **IC$_b$**-mediated cleavage of Boc-protective group from DNA-(Boc)glycine conjugate **DNA-13**. Reaction conditions: 25 eq. of **IC$_b$**, 0.5 mM **IC$_b$**, 50 °C, 4 h.

No.	aniline **9a** [eq.][a]	aniline **9a** [mM]	**DNA-14** [%][b]	depurination [%][b]
1	12.5	0.25	46	13
2	25	0.5	42	10
3	50	1.0	16	traces
4	100	2.0	8	traces

[a] versus the DNA-(Boc)glycine conjugate **DNA-13**; [b] HPLC analysis of the crude.

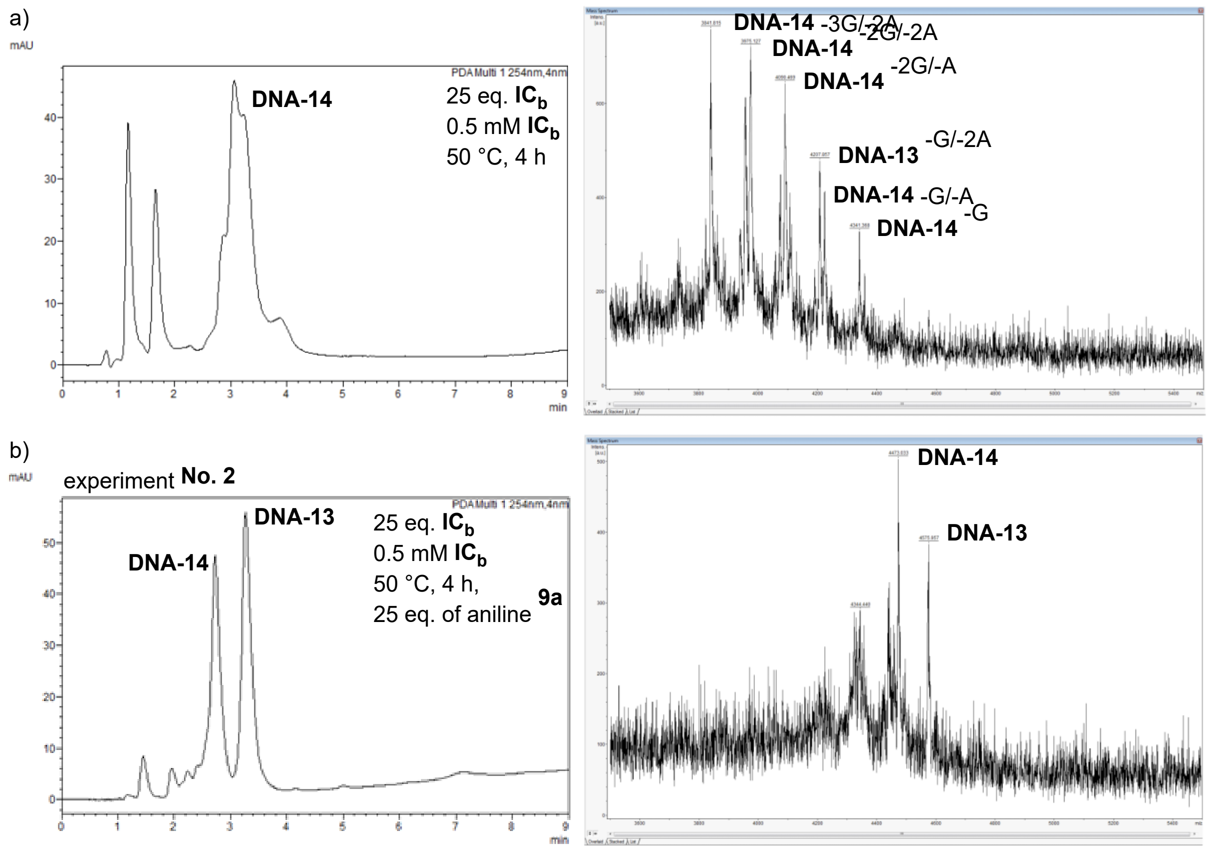

Figure S83. Copolymer micelle **IC$_b$**-mediated cleavage of Boc protective group from DNA-(Boc)glycine conjugate **DNA-13** in the abscence and in the presence of aniline **9a** as buffering component, for reaction conditions see Table S19. HPLC traces and MALDI-MS spectra show **IC$_b$**-mediated cleavage of Boc protective group from DNA-(Boc)glycine conjugate **DNA-13**: a) in the abscence of aniline **9a**, and b) in the presence of aniline **9a** (experiment **No. 2**). Please note that experiment **No. 2** was analyzed at a different gradient of aqueous triethylammonium acetate buffer (10 mM, pH= 8) and methanol (10% - 80% of methanol over 9 min).

6. Extending the scope of the micelle-mediated reactions: Biginelli reaction

6.1. Copolymer micelle **I/II**-mediated Biginelli reaction of aldehyde conjugate **DNA-1**, urea **16**, and ethyl acetoacetate **17** to DNA-dihydropyrimidin-2(1*H*)-one conjugate **DNA-15**: first attempts

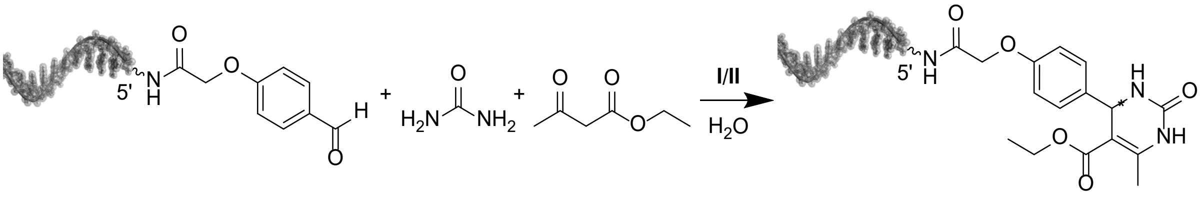

Scheme S11. Reaction scheme of the copolymer micelle **I/II**-mediated Biginelli reaction to **DNA-15**.

Protocol: To a solution of DNA-aldehyde conjugate **DNA-1** (500 pmol) in distilled water were added urea **16** (4 µmol, 8000 eq.) dissolved in 2.5 µL of distilled water (taken from a stock solution: 400 µmol dissolved in 250 µL of distilled water), ethyl acetoacetate **17** (4 µmol, 8000 eq.), and copolymer micelle **I/II** (25 nmol, 50 eq.) dissolved in 16 µL of distilled water taken from an aqueous stock solution (1.25 µmol in 800 µL). The reaction mixtures were filled with distilled water giving a final concentration of 0.5 mM of copolymer **I/II**. The reaction mixtures were shaken at 40 °C for 21 hour or at 40 °C for 63 hours. Then, 70 µL of distilled water were added and the reaction mixtures were thoroughly extracted with ethyl acetate (8 x 200 µL). The aqueous solution was evaporated in a SpeedVac, the residue was redissolved in 45 µL of distilled water, and all coupling products **DNA-15** were analyzed by RP-HPLC (Phenomenex, Gemini; 5 µm, C18, 110 Å, 100*4.6 mm) with a gradient of aqueous triethylammonium acetate buffer (10 mM, pH= 8) and methanol (10% - 60% of methanol over 9 min), and by MALDI-MS analysis. The conversion was estimated based on the area under the curve of the product peak versus the starting material peak in the HPLC-trace (analytical HPLC) and by MALDI-MS.

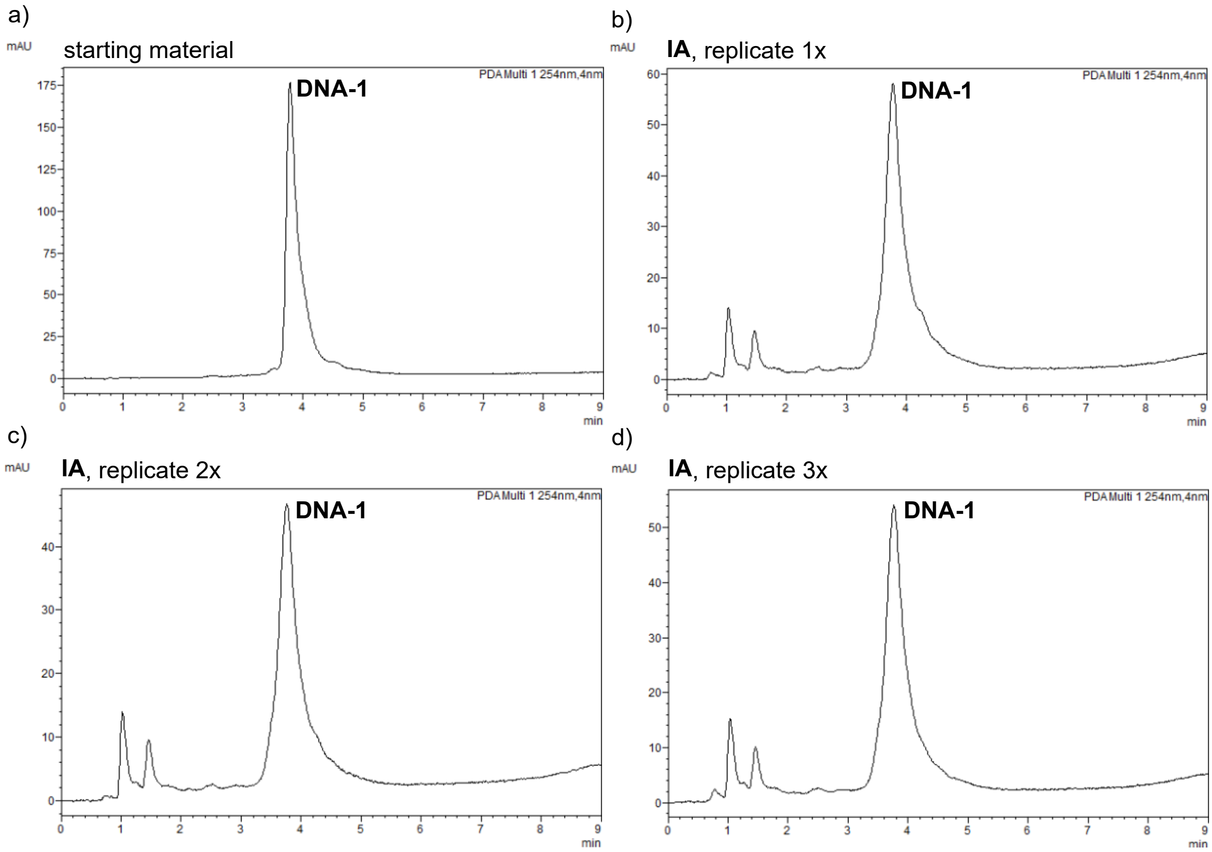

Figure S84. Copolymer **IA**-mediated Biginelli reaction of the oligonucleotide-aldehyde conjugate **DNA-1**, urea **16**, and ethyl acetoacetate **17** at 40 °C for 21 hour. HPLC traces show: a) oligonucleotide-aldehyde conjugate **DNA-1** and b)-d) experiment with copolymer **IA** in triplicate.

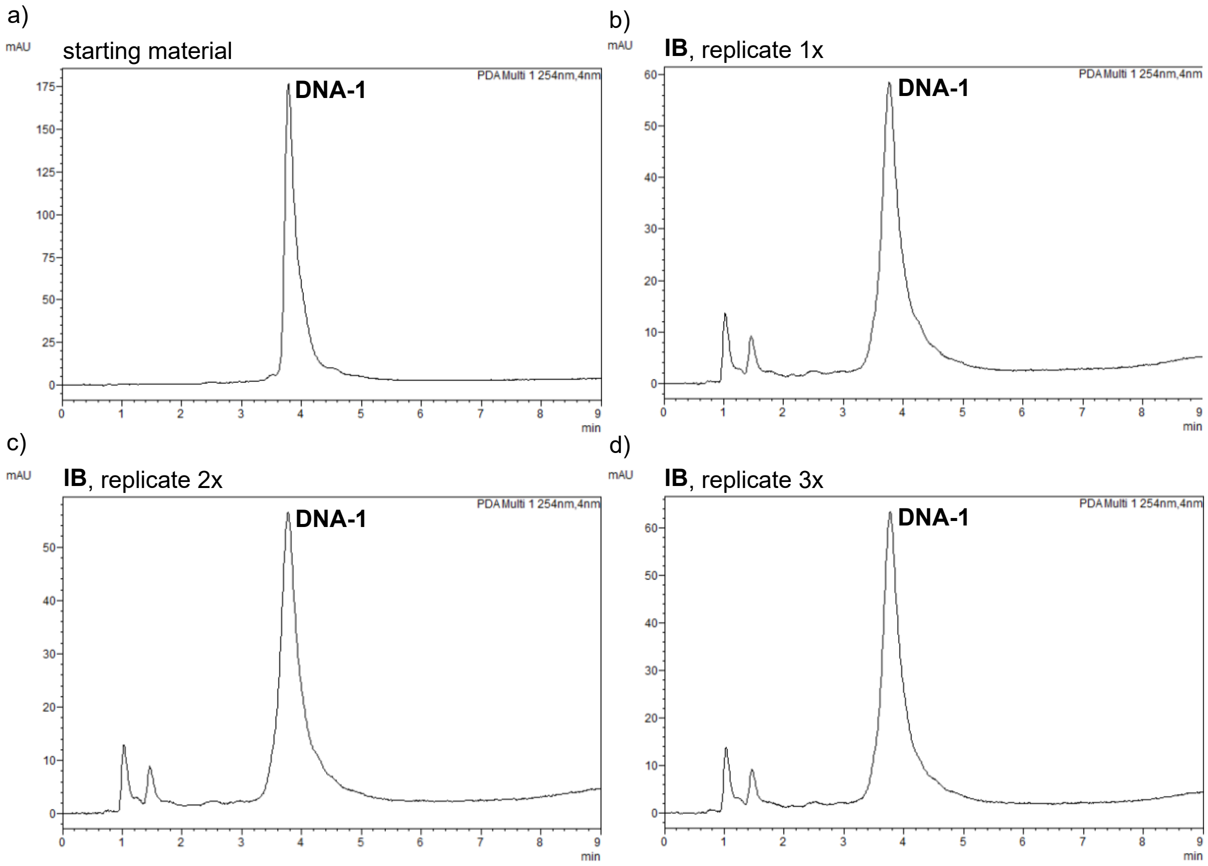

Figure S85. Copolymer **IB**-mediated Biginelli reaction of the oligonucleotide-aldehyde conjugate **DNA-1**, urea **16**, and ethyl acetoacetate **17** at 40 °C for 21 hour. HPLC traces show: a) oligonucleotide-aldehyde conjugate **DNA-1** and b)-d) experiment with copolymer **IB** in triplicate.

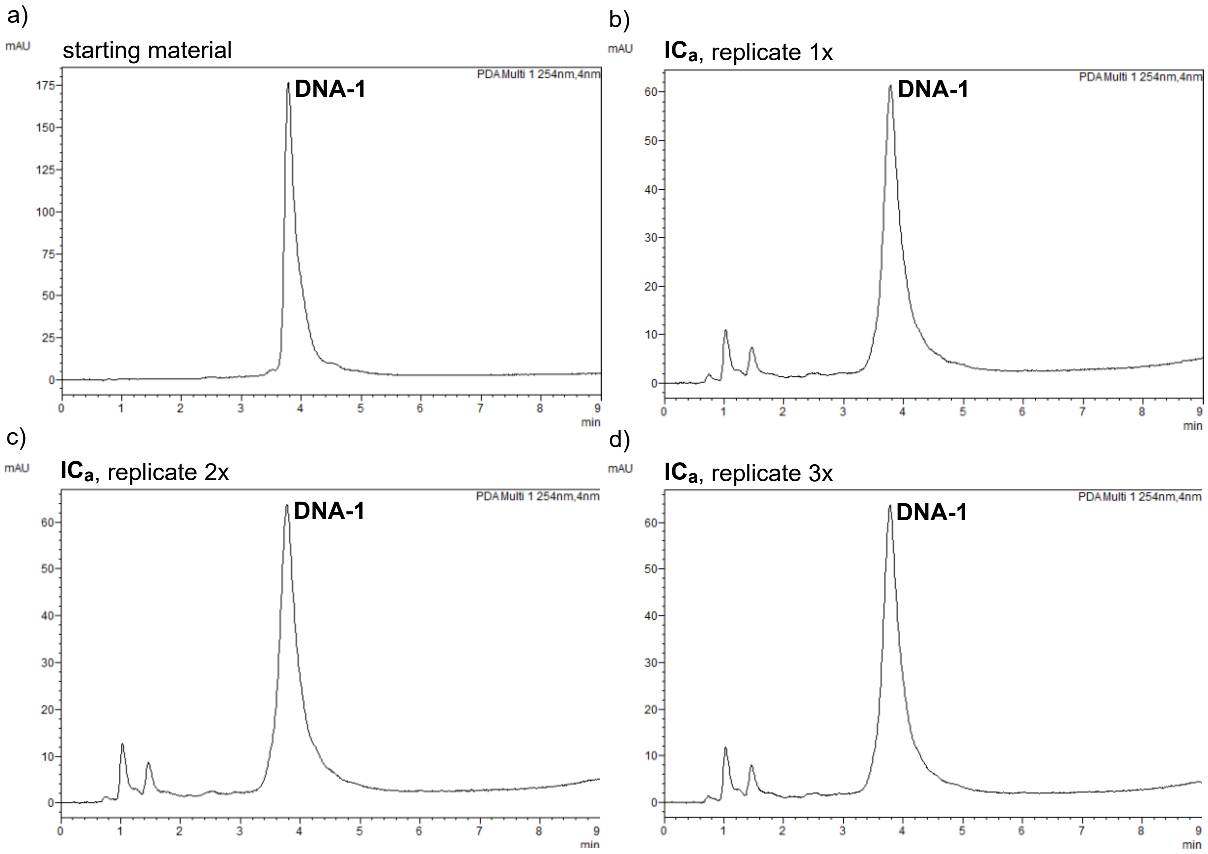

Figure S86. Copolymer **IC**$_a$-mediated Biginelli reaction of the oligonucleotide-aldehyde conjugate **DNA-1**, urea **16**, and ethyl acetoacetate **17** at 40 °C for 21 hour. HPLC traces show: a) oligonucleotide-aldehyde conjugate **DNA-1** and b)-d) experiment with copolymer **IC**$_a$ in triplicate.

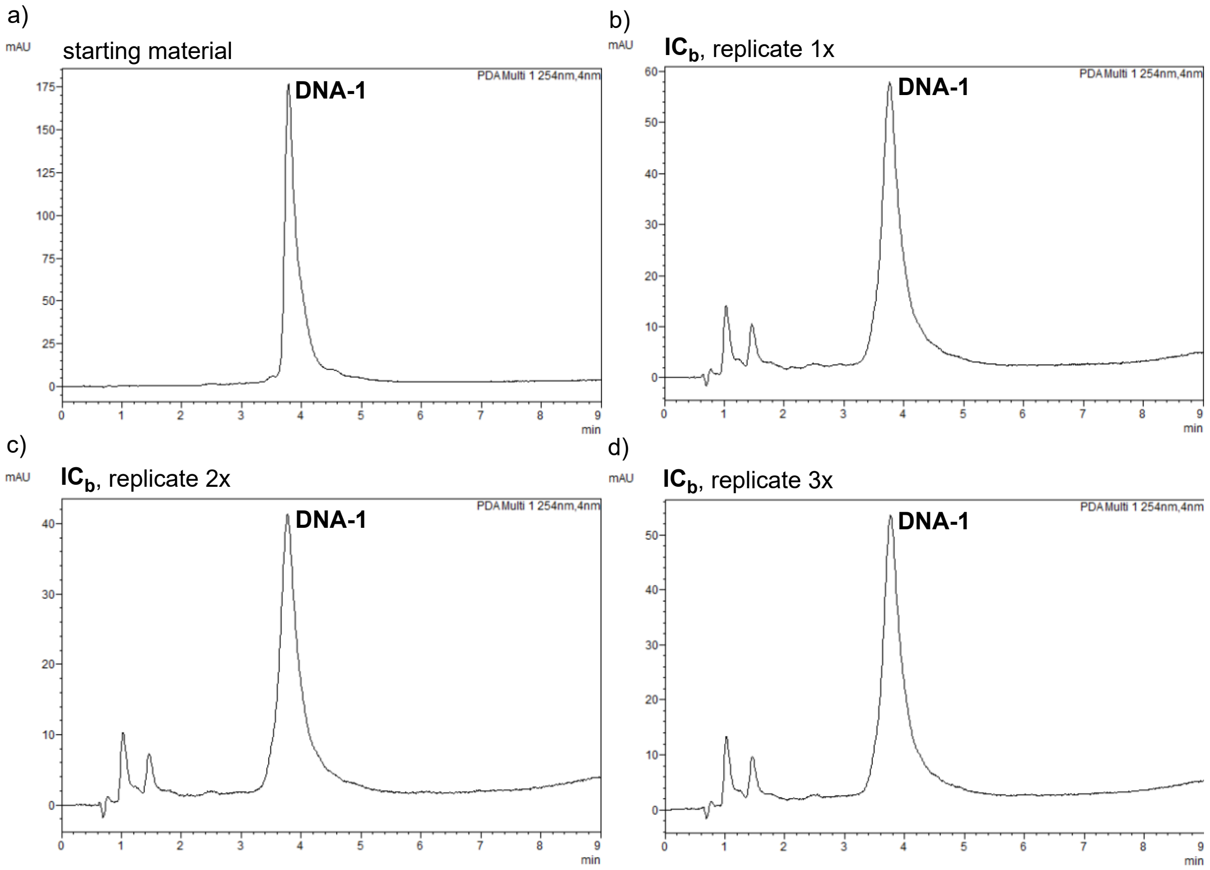

Figure S87. Copolymer **IC$_b$**-mediated Biginelli reaction of the oligonucleotide-aldehyde conjugate **DNA-1**, urea **16**, and ethyl acetoacetate **17** at 40 °C for 21 hour. HPLC traces show: a) oligonucleotide-aldehyde conjugate **DNA-1** and b)-d) experiment with copolymer **IC$_b$** in triplicate.

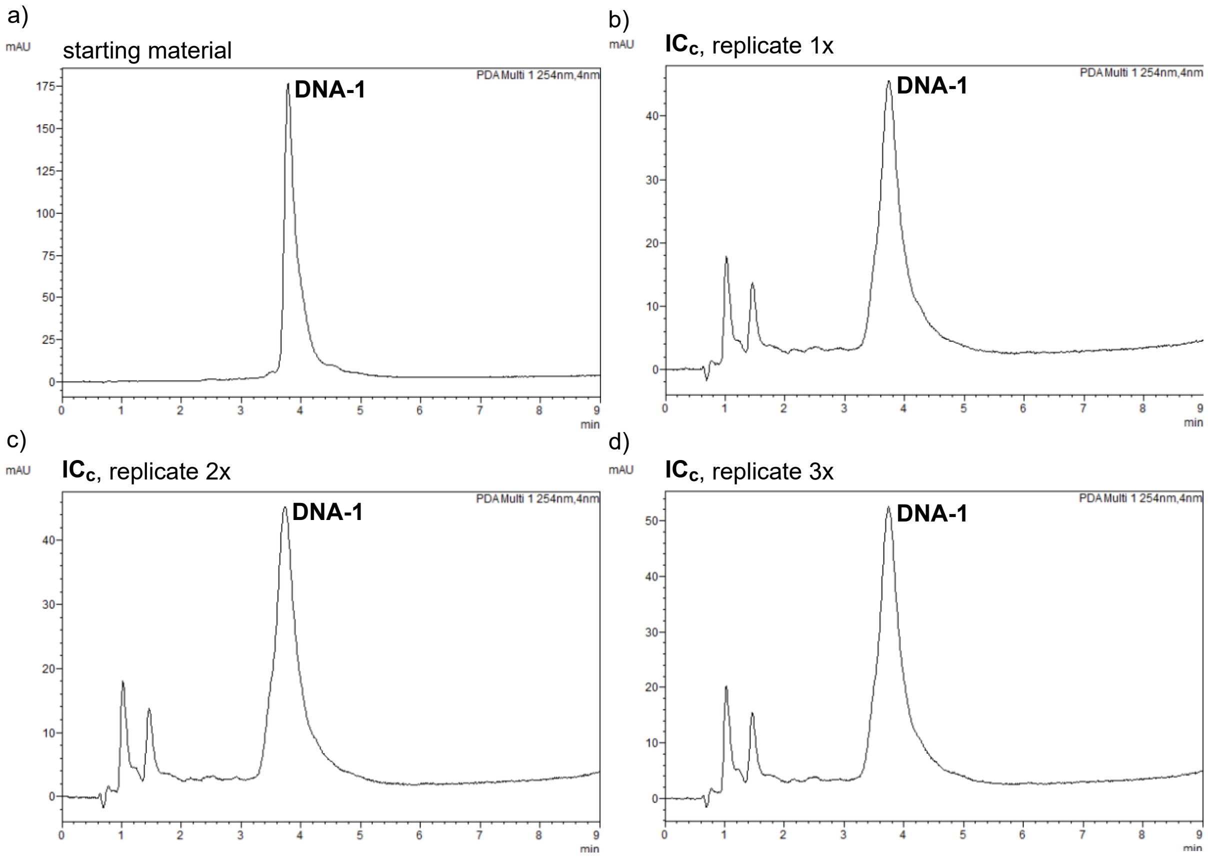

Figure S88. Copolymer **IC**$_c$-mediated Biginelli reaction of the oligonucleotide-aldehyde conjugate **DNA-1**, urea **16**, and ethyl acetoacetate **17** at 40 °C for 21 hour. HPLC traces show: a) oligonucleotide-aldehyde conjugate **DNA-1** and b)-d) experiment with copolymer **IC**$_c$ in triplicate.

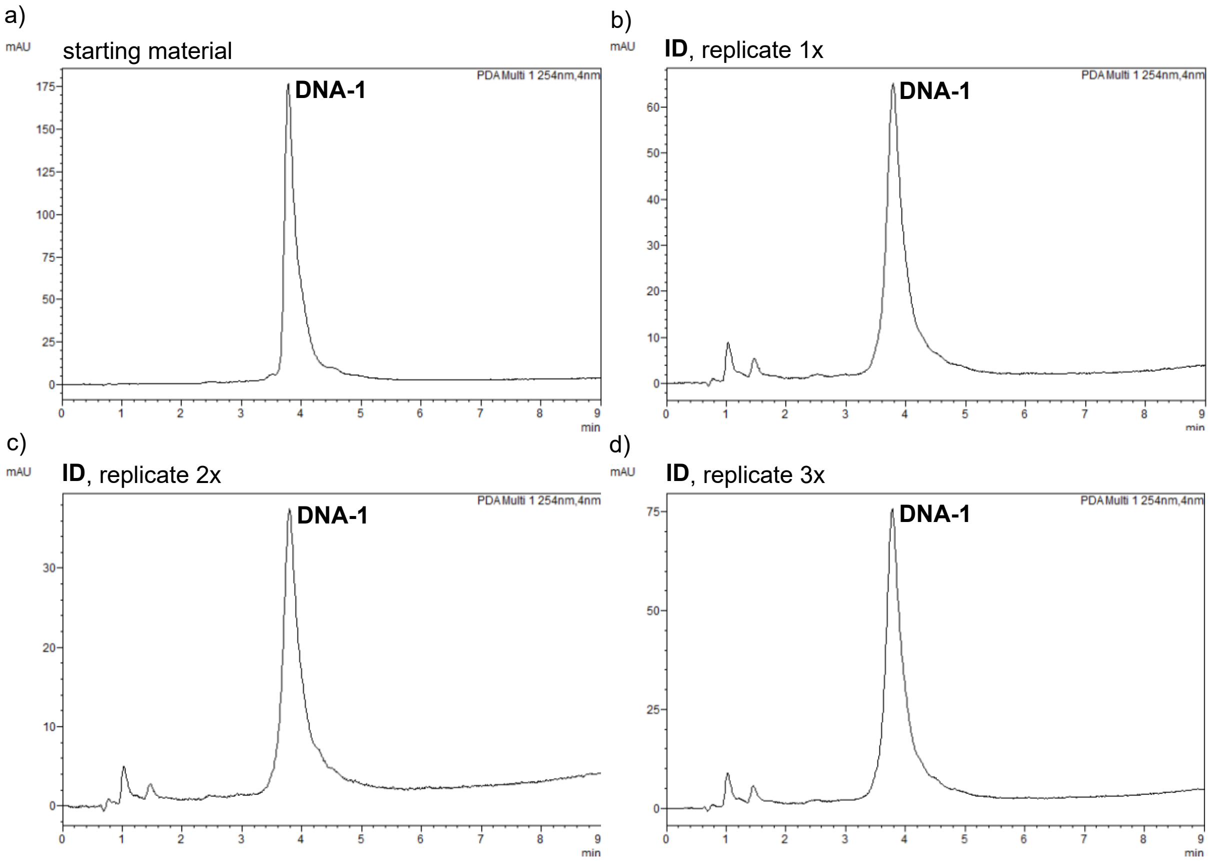

Figure S89. Copolymer **ID**-mediated Biginelli reaction of the oligonucleotide-aldehyde conjugate **DNA-1**, urea **16**, and ethyl acetoacetate **17** at 40 °C for 21 hour. HPLC traces show: a) oligonucleotide-aldehyde conjugate **DNA-1** and b)-d) experiment with copolymer **ID** in triplicate.

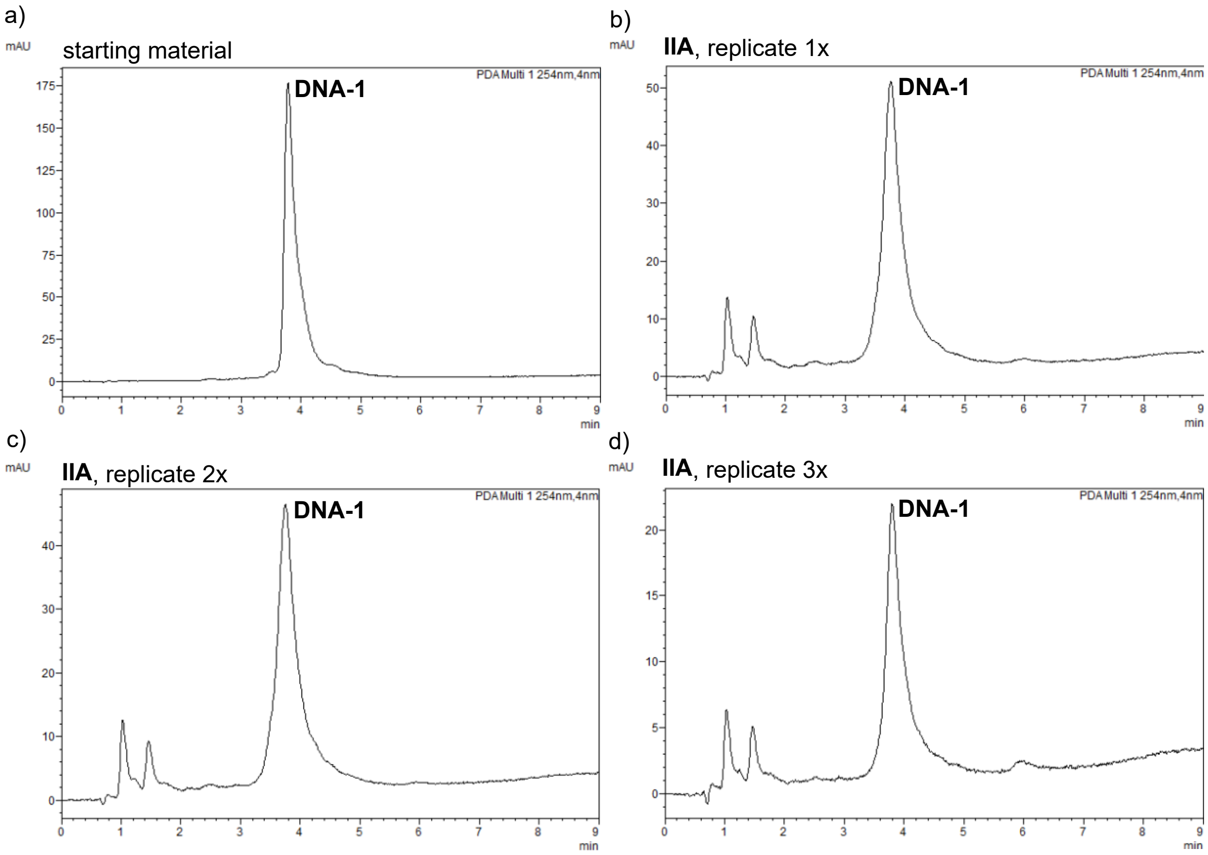

Figure S90. Copolymer **IIA**-mediated Biginelli reaction of the oligonucleotide-aldehyde conjugate **DNA-1**, urea **16**, and ethyl acetoacetate **17** at 40 °C for 21 hour. HPLC traces show: a) oligonucleotide-aldehyde conjugate **DNA-1** and b)-d) experiment with copolymer **IIA** in triplicate.

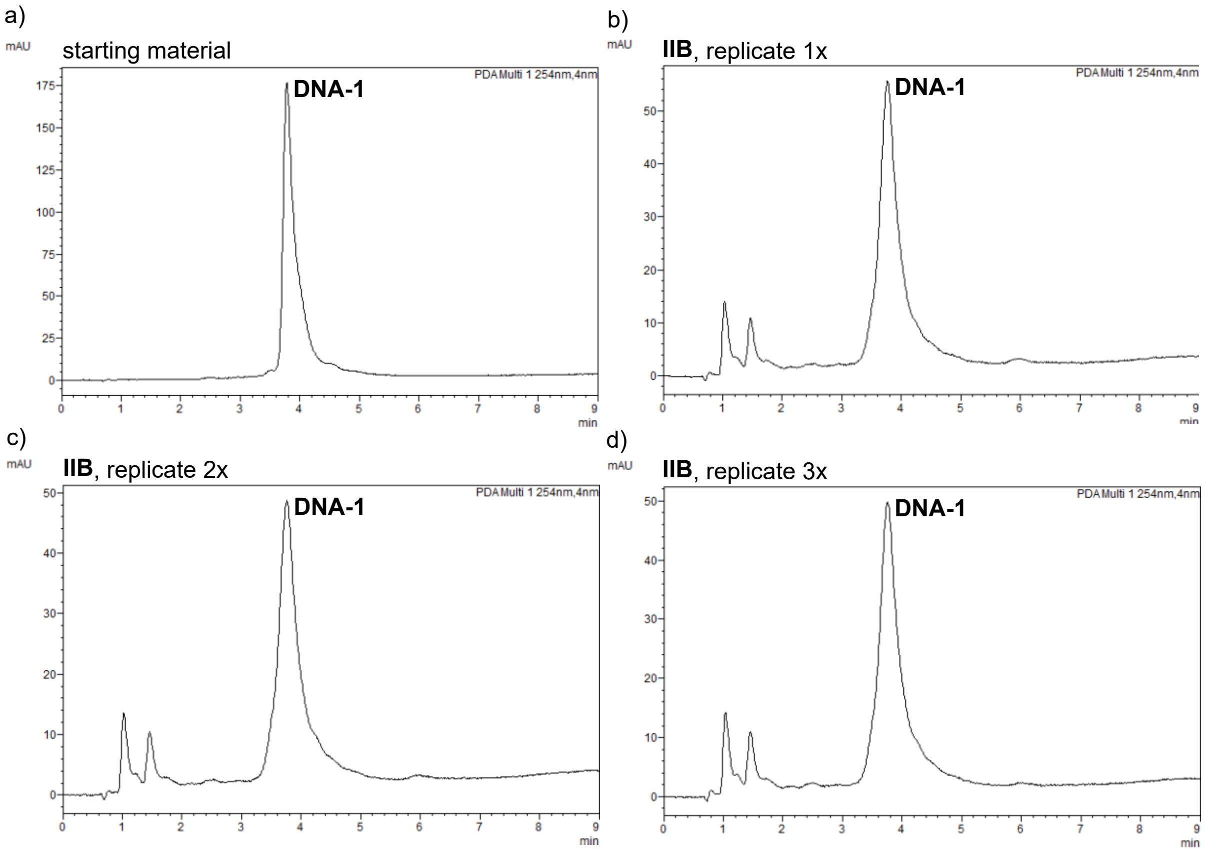

Figure S91. Copolymer **IIB**-mediated Biginelli reaction of the oligonucleotide-aldehyde conjugate **DNA-1**, urea **16**, and ethyl acetoacetate **17** at 40 °C for 21 hour. HPLC traces show: a) oligonucleotide-aldehyde conjugate **DNA-1** and b)-d) experiment with copolymer **IIB** in triplicate.

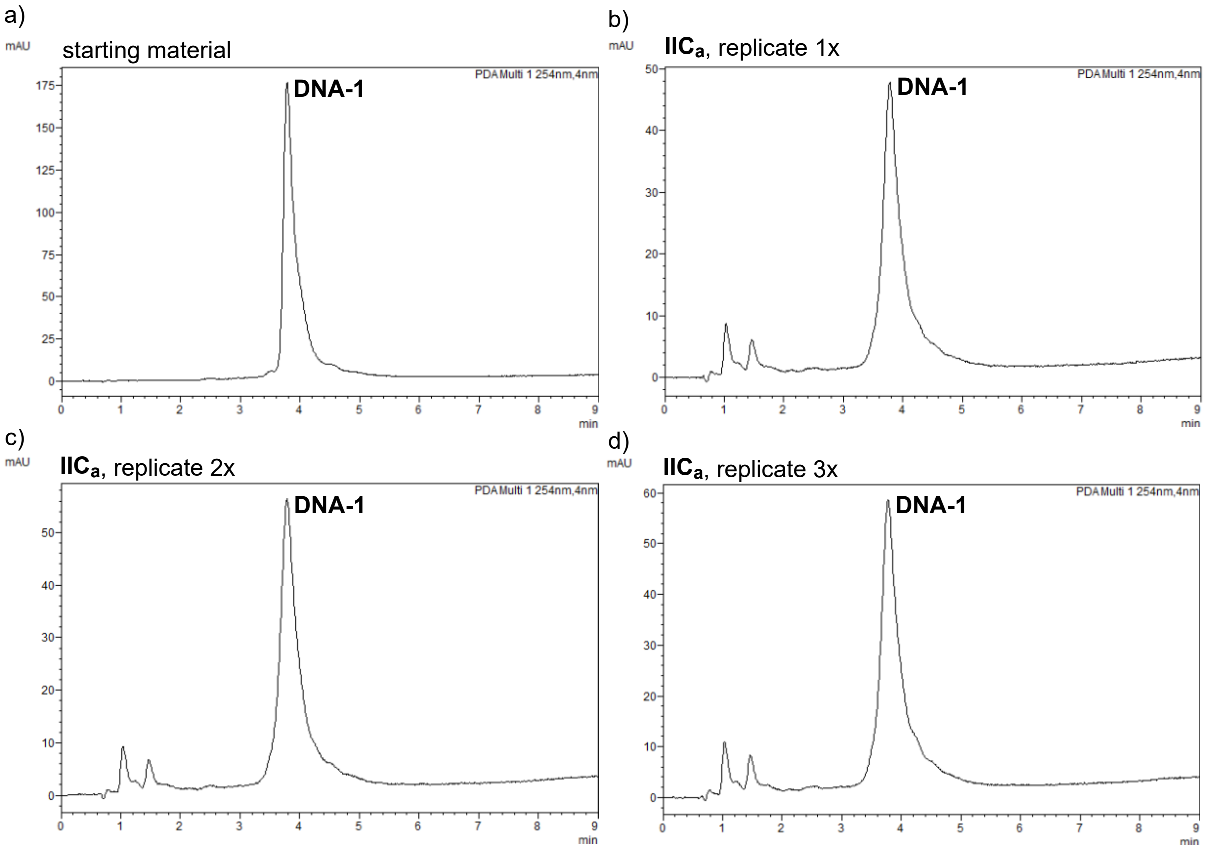

Figure S92. Copolymer **IIC$_a$**-mediated Biginelli reaction of the oligonucleotide-aldehyde conjugate **DNA-1**, urea **16**, and ethyl acetoacetate **17** at 40 °C for 21 hour. HPLC traces show: a) oligonucleotide-aldehyde conjugate **DNA-1** and b)-d) experiment with copolymer **IIC$_a$** in triplicate.

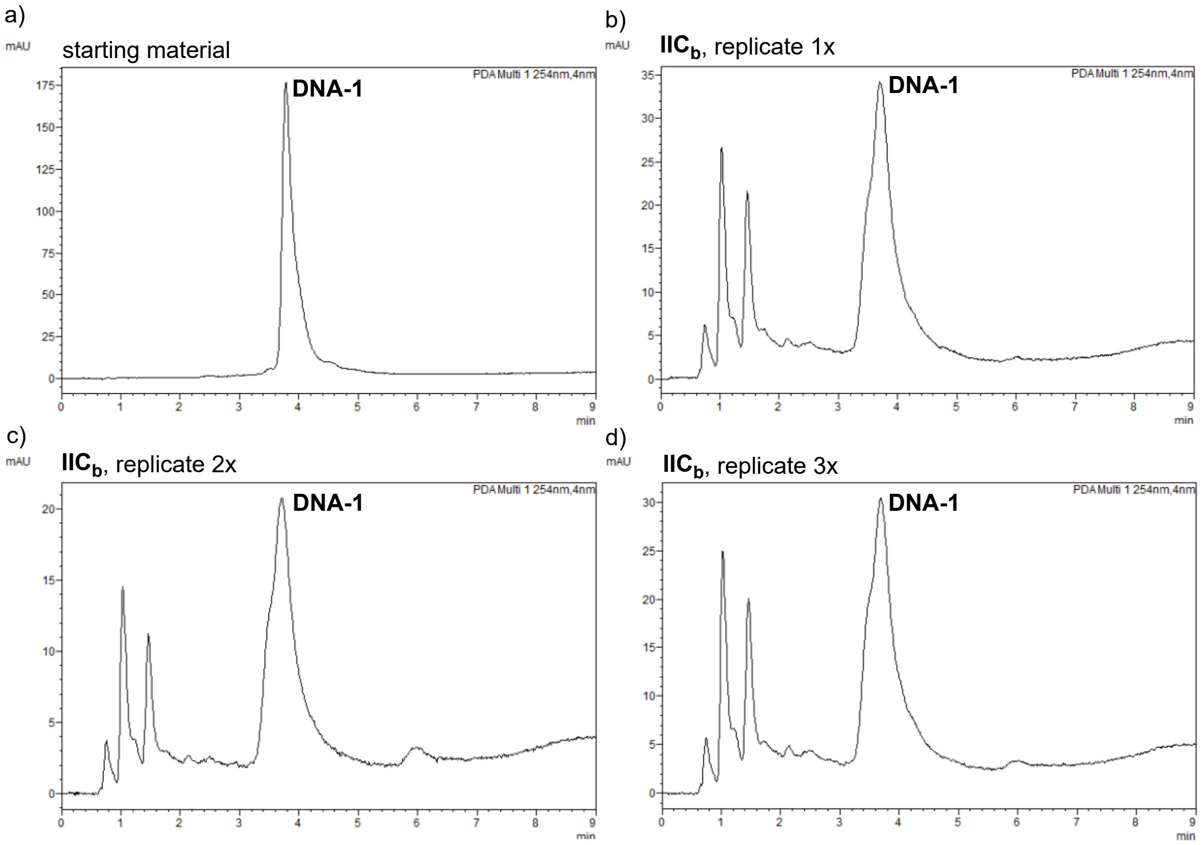

Figure S93. Copolymer **IIC$_b$**-mediated Biginelli reaction of the oligonucleotide-aldehyde conjugate **DNA-1**, urea **16**, and ethyl acetoacetate **17** at 40 °C for 21 hour. HPLC traces show: a) oligonucleotide-aldehyde conjugate **DNA-1** and b)-d) experiment with copolymer **IIC$_b$** in triplicate.

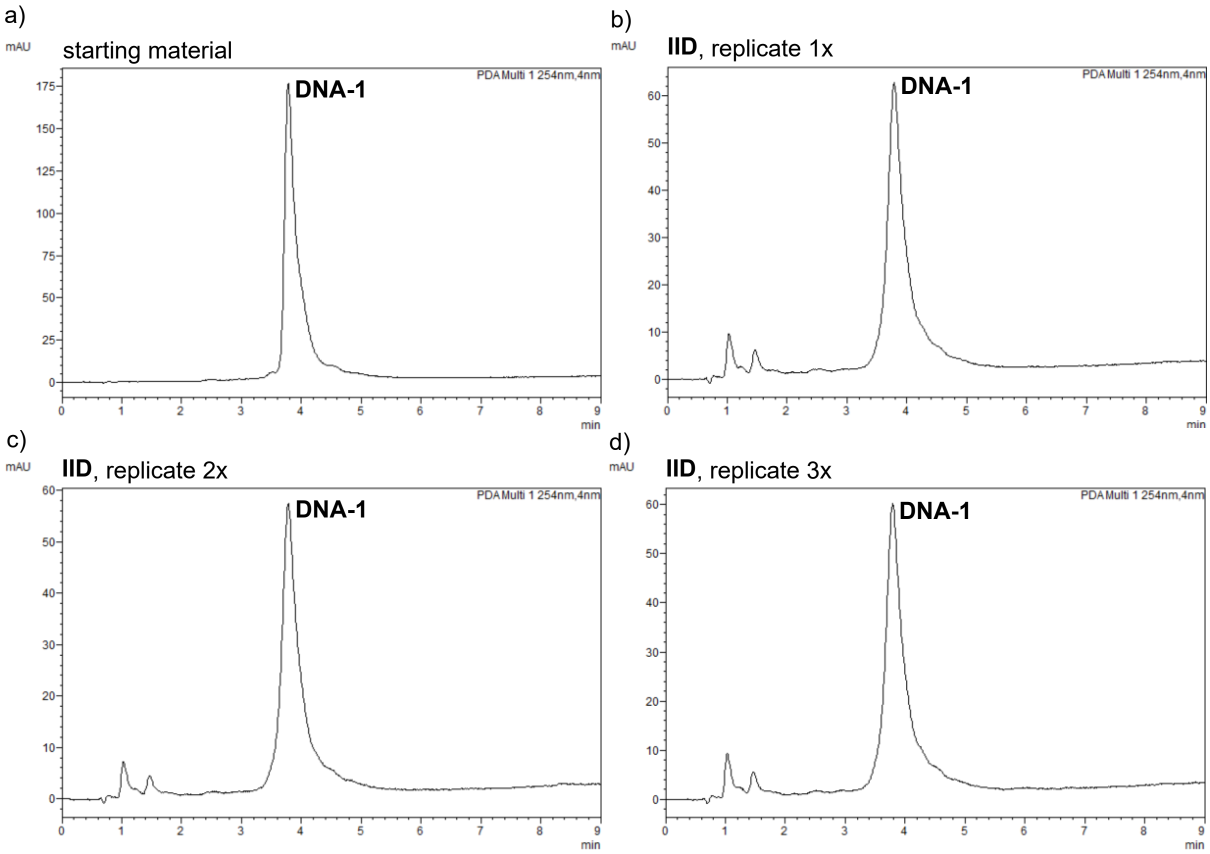

Figure S94. Copolymer **IID**-mediated Biginelli reaction of the oligonucleotide-aldehyde conjugate **DNA-1**, urea **16**, and ethyl acetoacetate **17** at 40 °C for 21 hour. HPLC traces show: a) oligonucleotide-aldehyde conjugate **DNA-1** and b)-d) experiment with copolymer **IID** in triplicate.

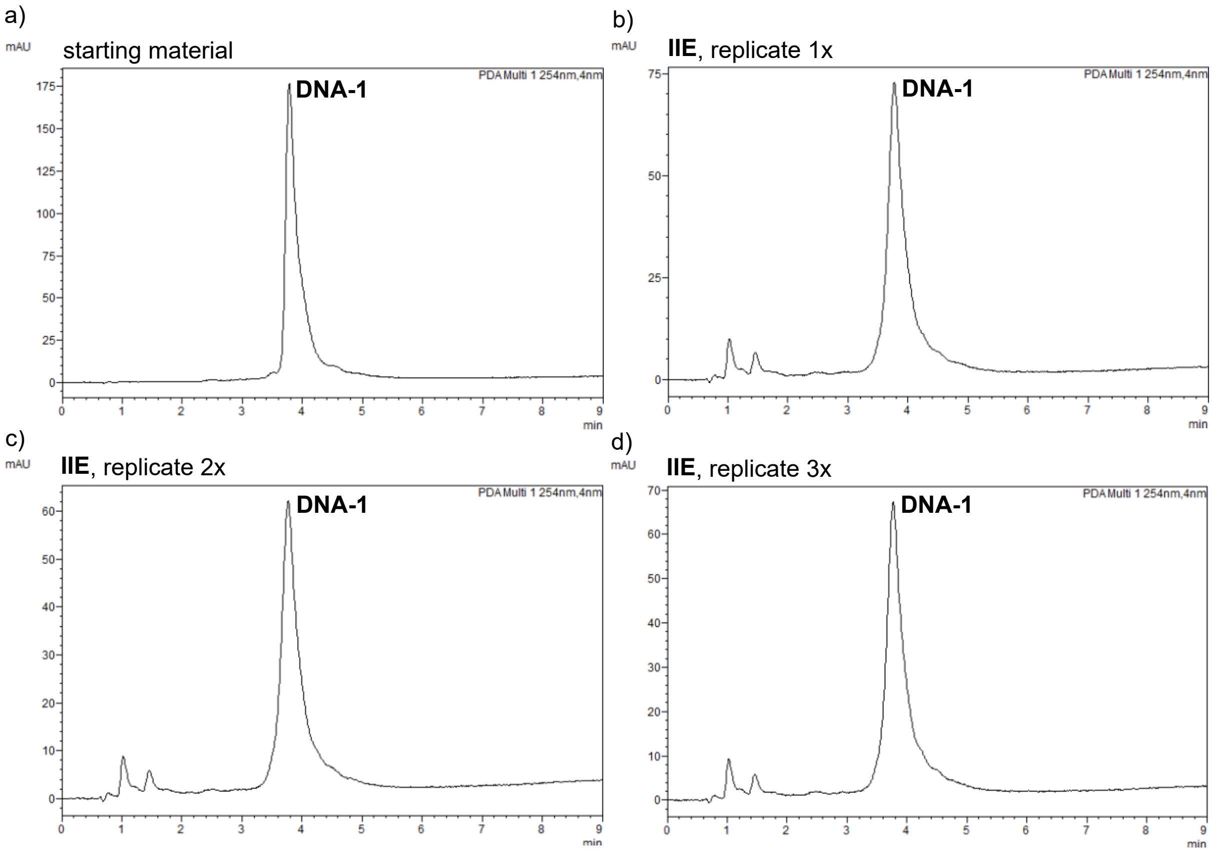

Figure S95. Copolymer **IIE**-mediated Biginelli reaction of the oligonucleotide-aldehyde conjugate **DNA-1**, urea **16**, and ethyl acetoacetate **17** at 40 °C for 21 hour. HPLC traces show: a) oligonucleotide-aldehyde conjugate **DNA-1** and b)-d) experiment with copolymer **IIE** in triplicate.

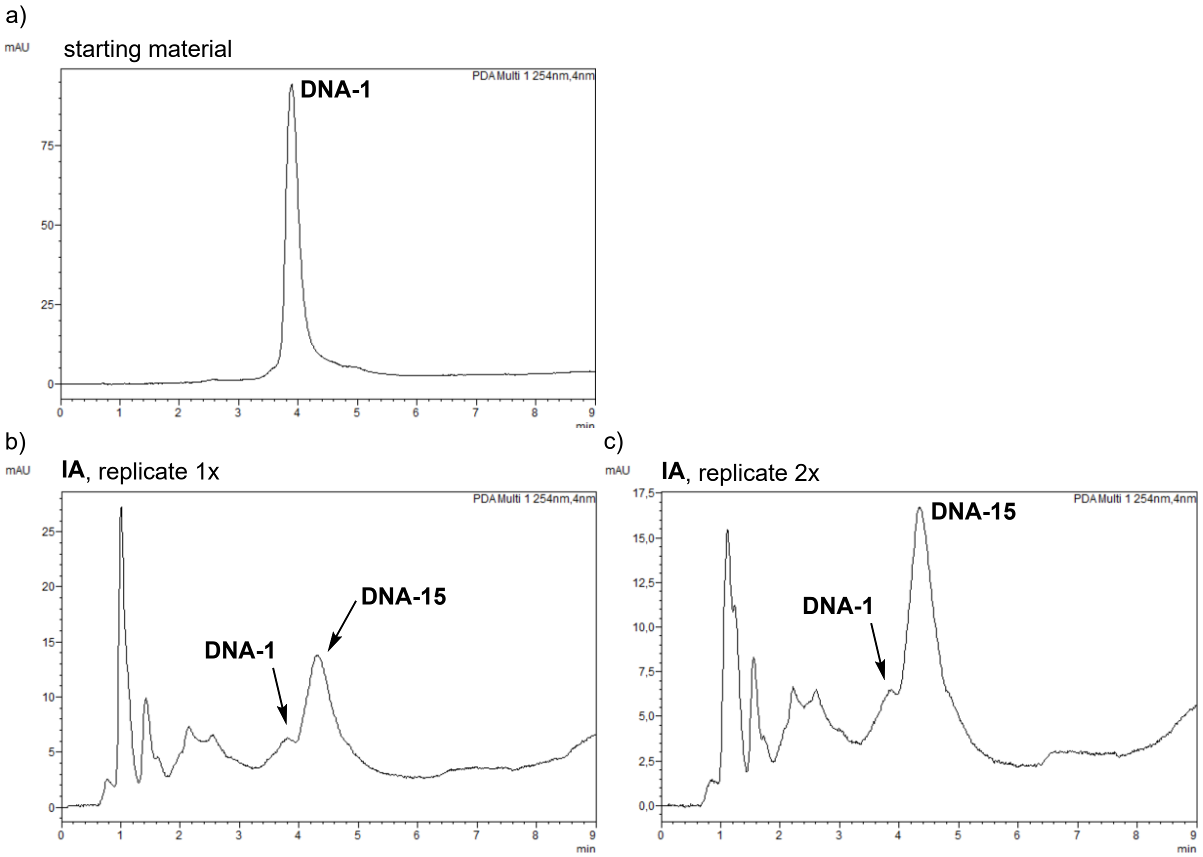

Figure S96. Copolymer **IA**-mediated Biginelli reaction of the oligonucleotide-aldehyde conjugate **DNA-1**, urea **16**, and ethyl acetoacetate **17** at 40 °C for 63 hours. HPLC traces show: a) oligonucleotide-aldehyde conjugate **DNA-1** and b)-c) experiment with copolymer **IA** in duplicate.

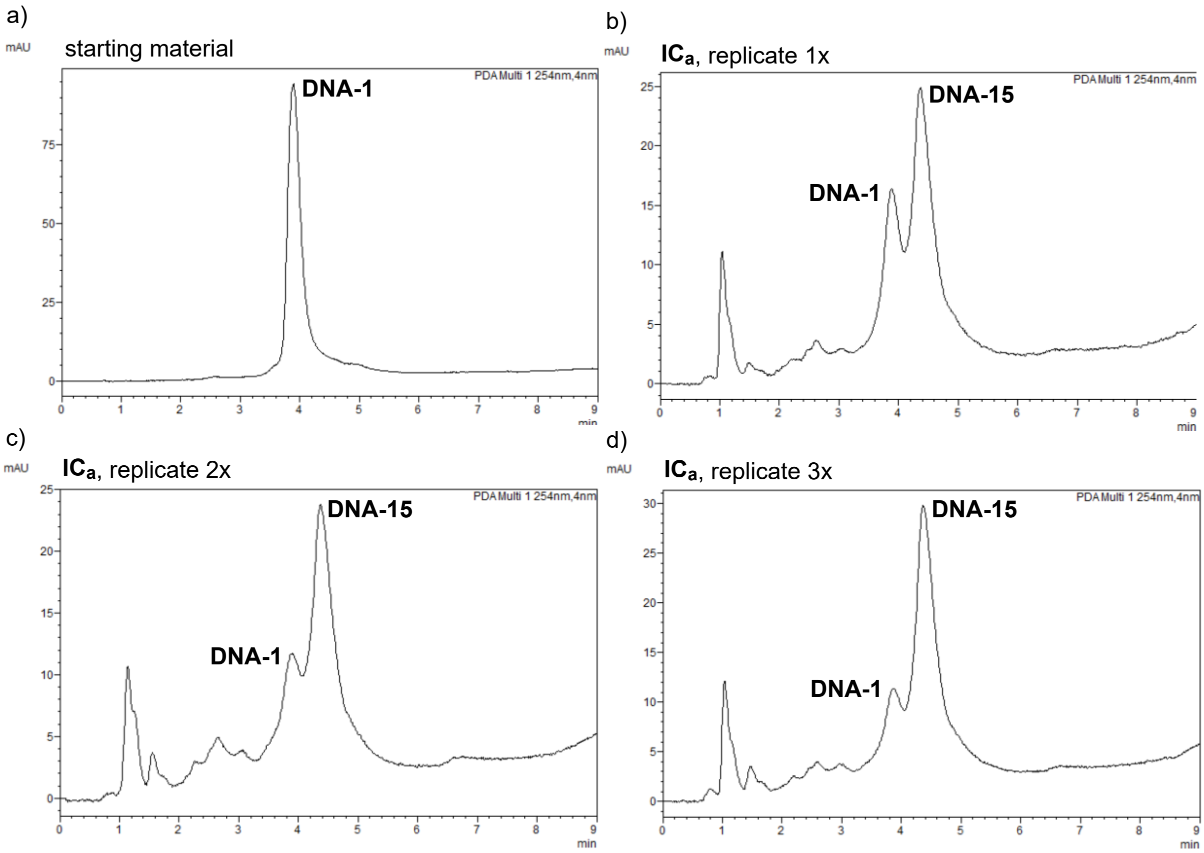

Figure S97. Copolymer **IC$_a$**-mediated Biginelli reaction of the oligonucleotide-aldehyde conjugate **DNA-1**, urea **16**, and ethyl acetoacetate **17** at 40 °C for 63 hours. HPLC traces show: a) oligonucleotide-aldehyde conjugate **DNA-1** and b)-d) experiment with copolymer **IC$_a$** in triplicate.

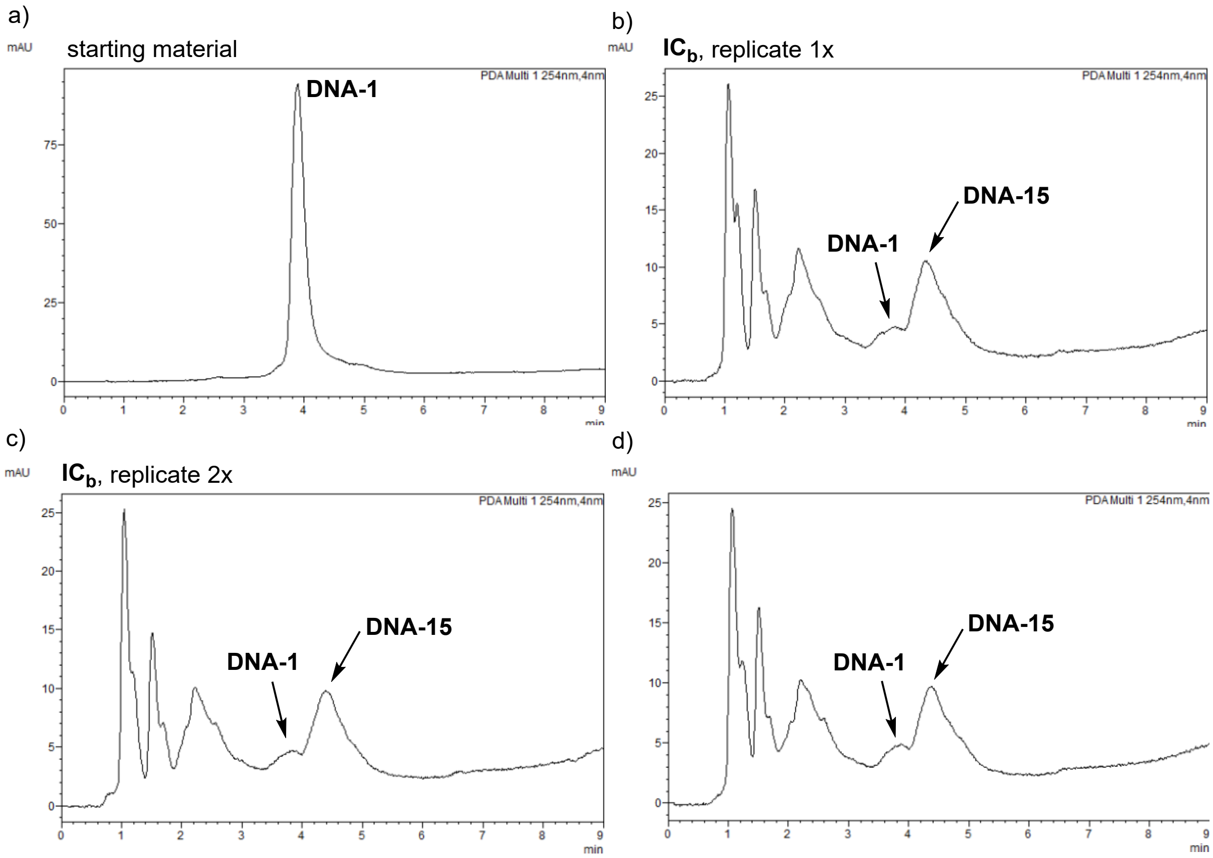

Figure S98. Copolymer **IC$_b$**-mediated Biginelli reaction of the oligonucleotide-aldehyde conjugate **DNA-1**, urea **16**, and ethyl acetoacetate **17** at 40 °C for 63 hours. HPLC traces show: a) oligonucleotide-aldehyde conjugate **DNA-1** and b)-d) experiment with copolymer **IC$_b$** in triplicate.

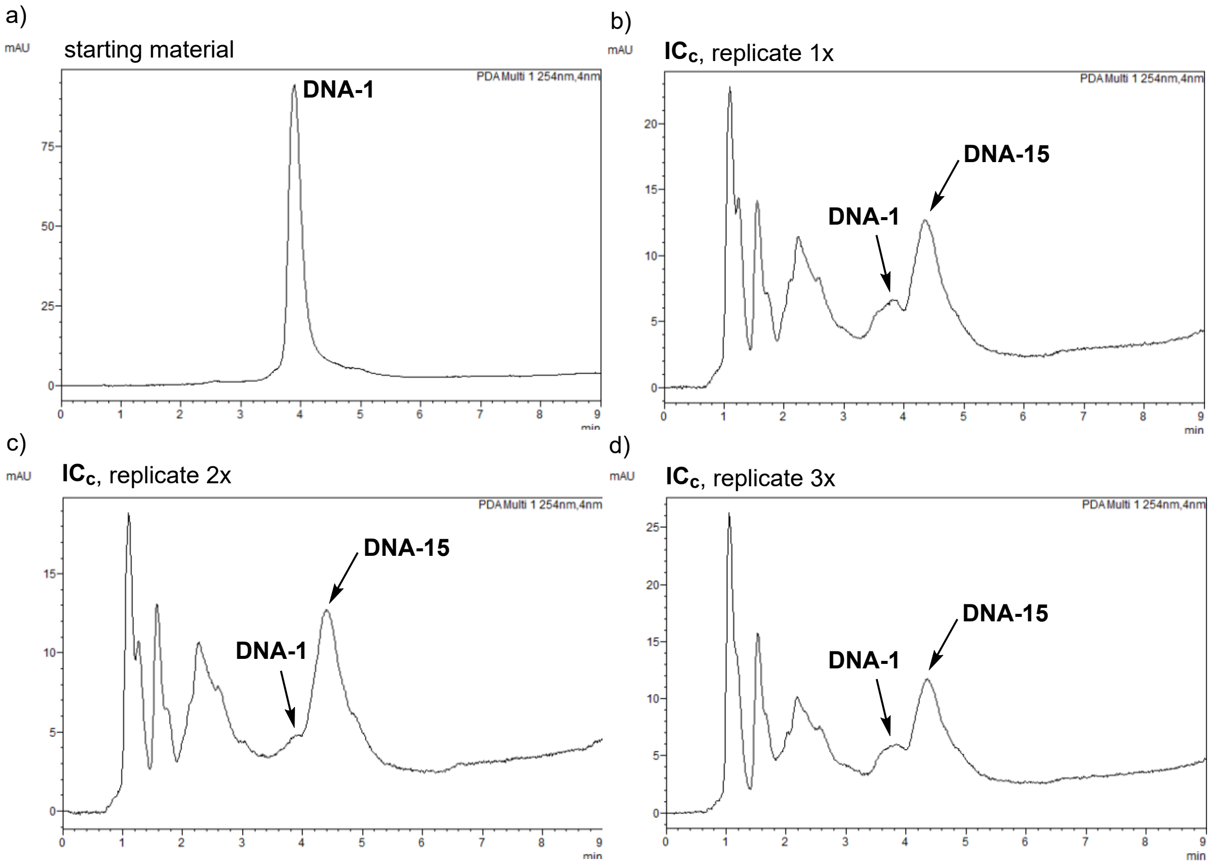

Figure S99. Copolymer **IC**$_c$-mediated Biginelli reaction of the oligonucleotide-aldehyde conjugate **DNA-1**, urea **16**, and ethyl acetoacetate **17** at 40 °C for 63 hours. HPLC traces show: a) oligonucleotide-aldehyde conjugate **DNA-1** and b)-d) experiment with copolymer **IC**$_c$ in triplicate.

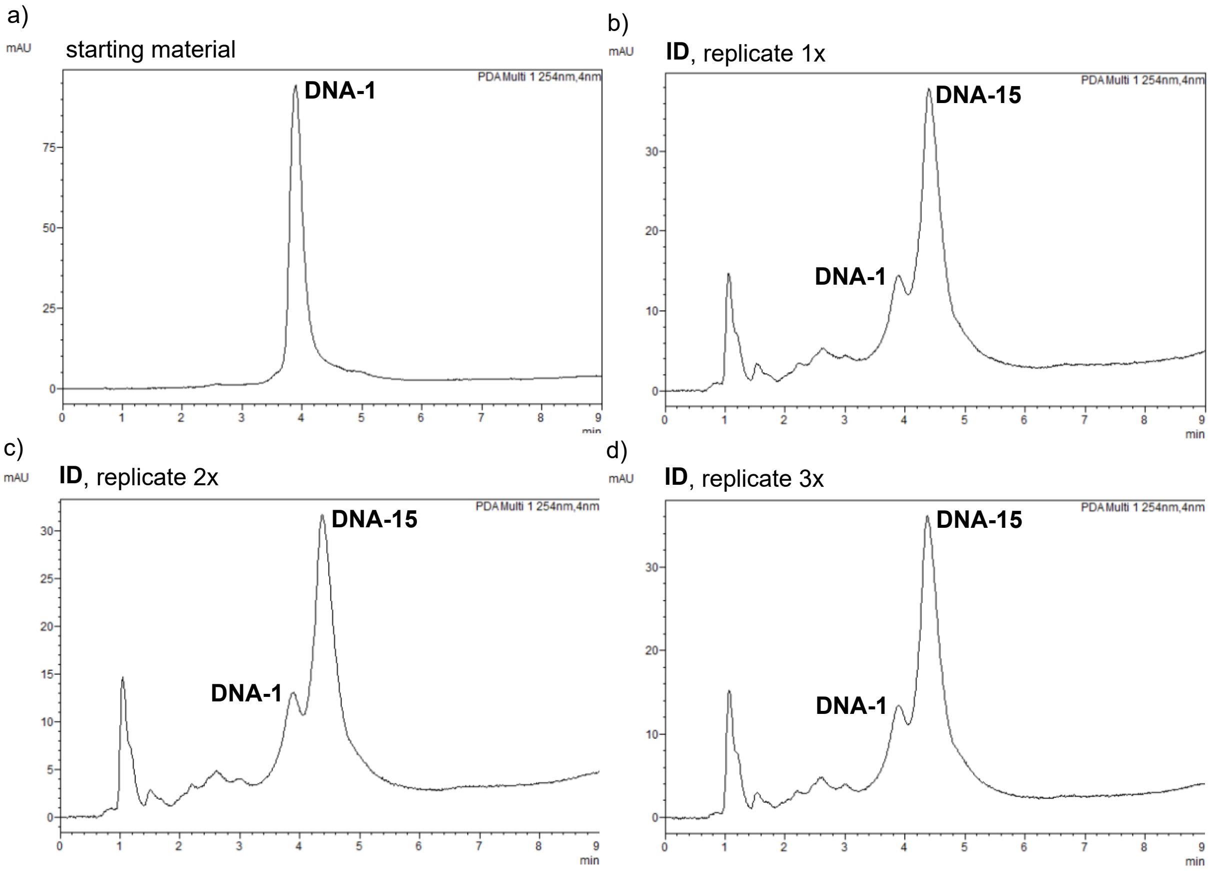

Figure S100. Copolymer **ID**-mediated Biginelli reaction of the oligonucleotide-aldehyde conjugate **DNA-1**, urea **16**, and ethyl acetoacetate **17** at 40 °C for 63 hours. HPLC traces show: a) oligonucleotide-aldehyde conjugate **DNA-1** and b)-d) experiment with copolymer **ID** in triplicate.

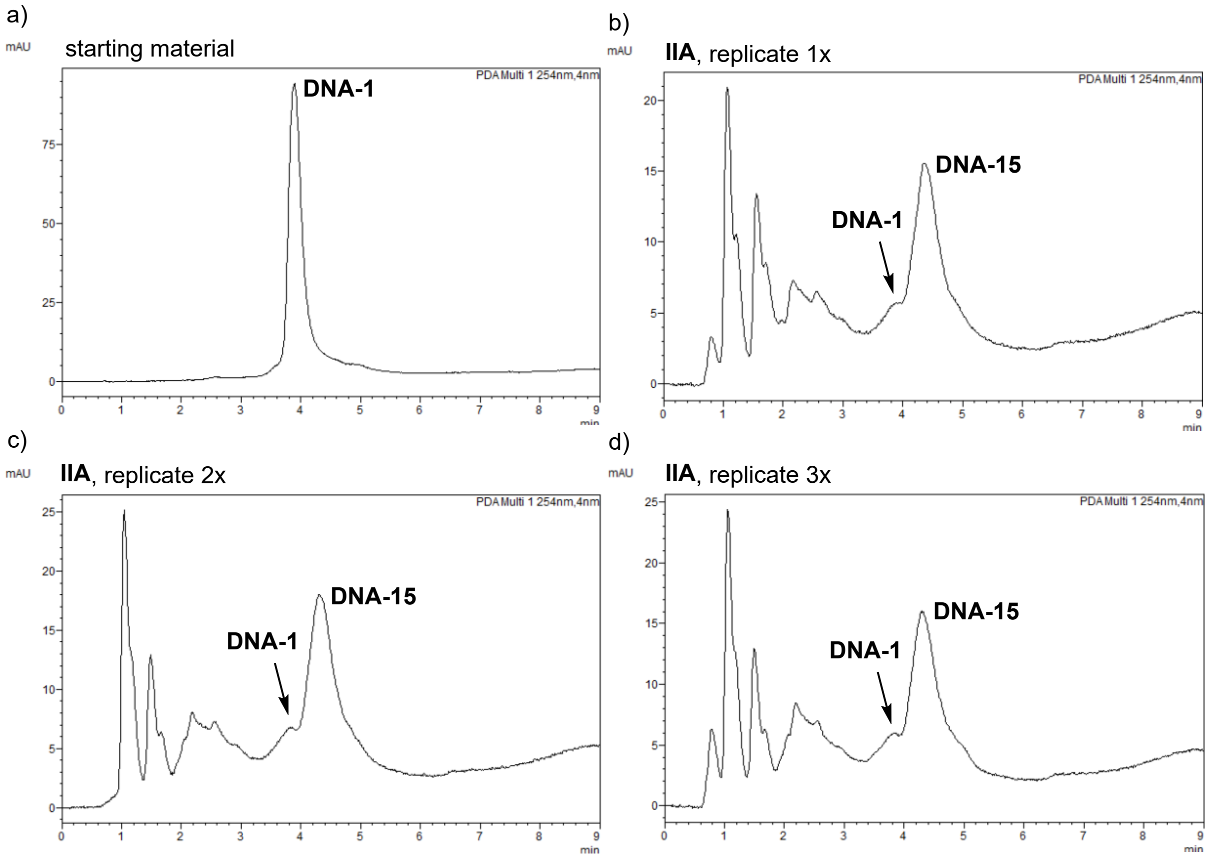

Figure S101. Copolymer **IIA**-mediated Biginelli reaction of the oligonucleotide-aldehyde conjugate **DNA-1**, urea **16**, and ethyl acetoacetate **17** at 40 °C for 63 hours. HPLC traces show: a) oligonucleotide-aldehyde conjugate **DNA-1** and b)-d) experiment with copolymer **IIA** in triplicate.

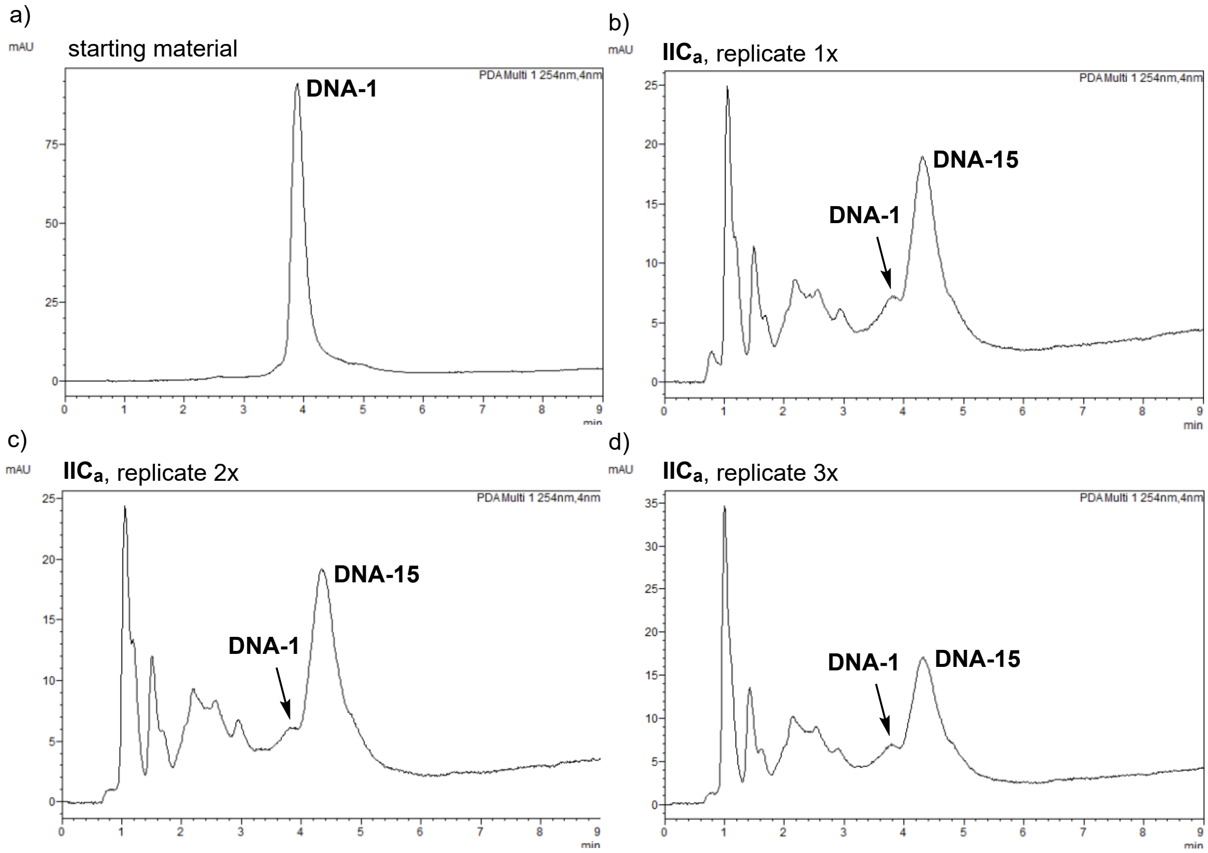

Figure S102. Copolymer **IIC$_a$**-mediated Biginelli reaction of the oligonucleotide-aldehyde conjugate **DNA-1**, urea **16**, and ethyl acetoacetate **17** at 40 °C for 63 hours. HPLC traces show: a) oligonucleotide-aldehyde conjugate **DNA-1** and b)-d) experiment with copolymer **IIC$_a$** in triplicate.

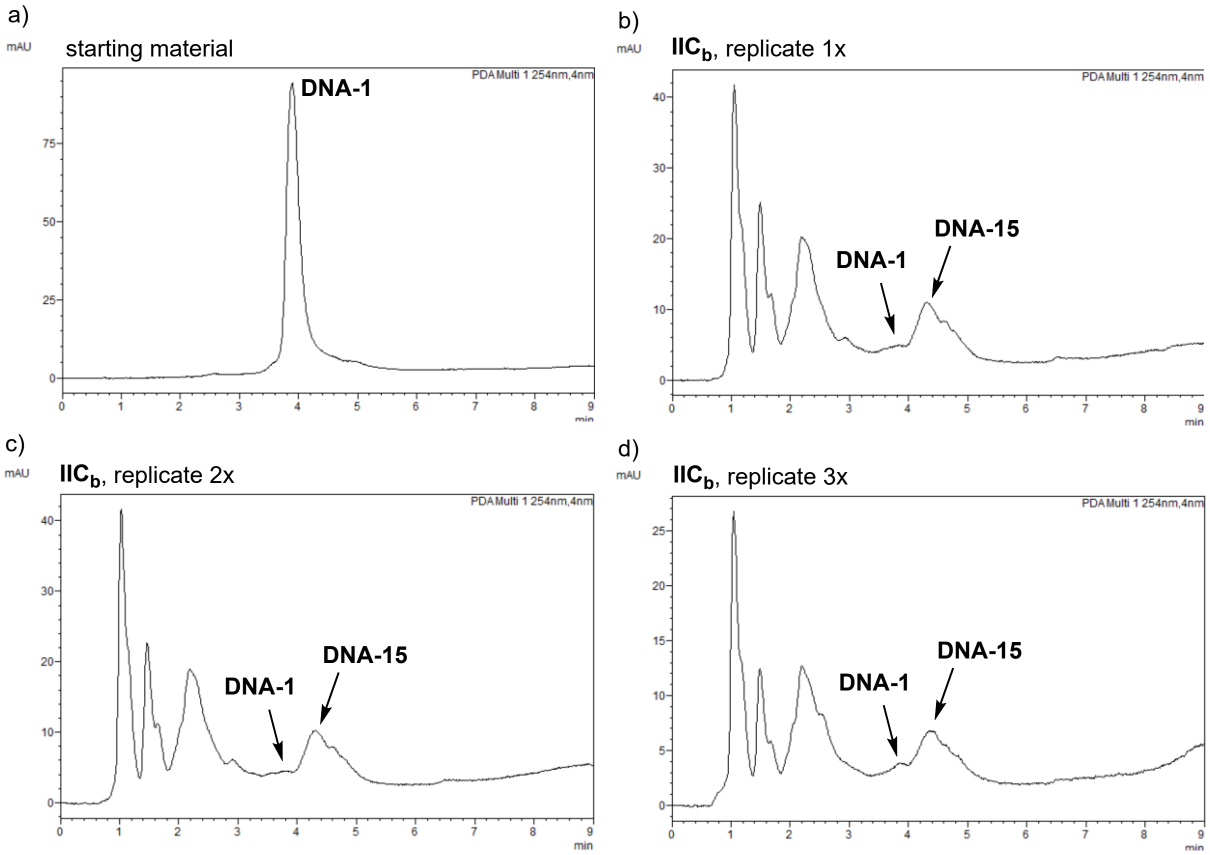

Figure S103. Copolymer **IIC$_b$**-mediated Biginelli reaction of the oligonucleotide-aldehyde conjugate **DNA-1**, urea **16**, and ethyl acetoacetate **17** at 40 °C for 63 hours. HPLC traces show: a) oligonucleotide-aldehyde conjugate **DNA-1** and b)-d) experiment with copolymer **IIC$_b$** in triplicate.

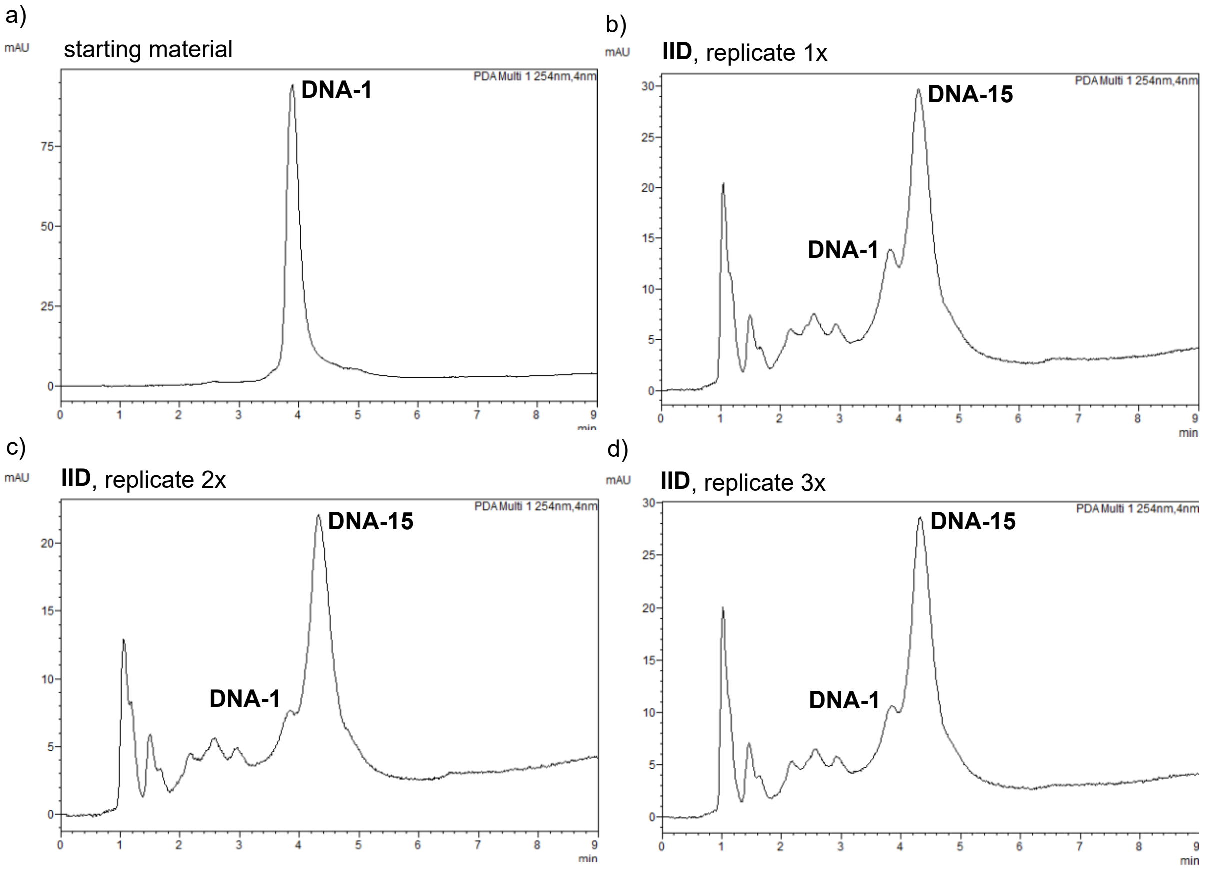

Figure S104. Copolymer **IID**-mediated Biginelli reaction of the oligonucleotide-aldehyde conjugate **DNA-1**, urea **16**, and ethyl acetoacetate **17** at 40 °C for 63 hours. HPLC traces show: a) oligonucleotide-aldehyde conjugate **DNA-1** and b)-d) experiment with copolymer **IID** in triplicate.

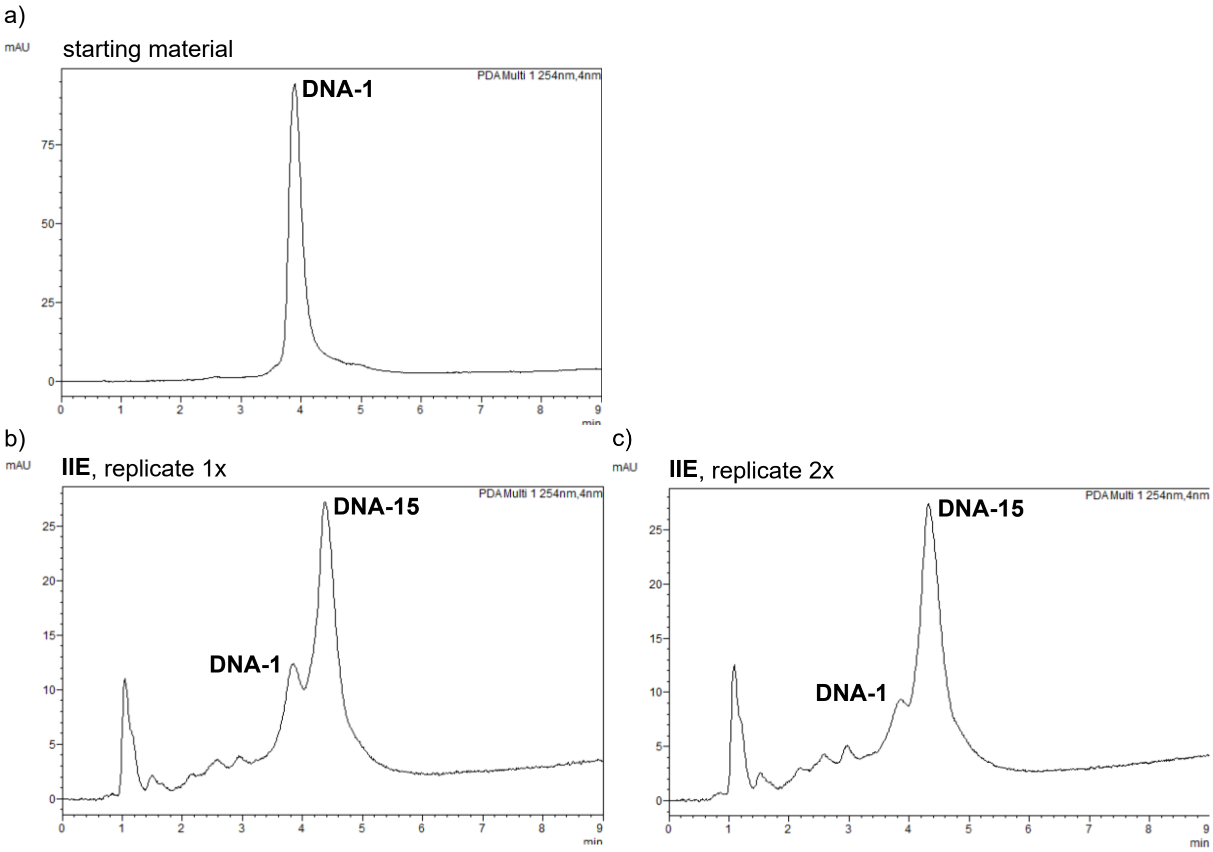

Figure S105. Copolymer **IIE**-mediated Biginelli reaction of the oligonucleotide-aldehyde conjugate **DNA-1**, urea **16**, and ethyl acetoacetate **17** at 40 °C for 63 hours. HPLC traces show: a) oligonucleotide-aldehyde conjugate **DNA-1** and b)-c) experiment with copolymer **IIE** in duplicate.

Table S20. Impact of copolymer micelle **I/II** design on DNA depurination during Biginelli reaction of aldehyde conjugate **DNA-1**, urea **16**, and ethyl acetoacetate **17** to DNA-dihydropyrimidin-2(1*H*)-one conjugate **DNA-15**: ranking micelles **I/II** from the lowest (**1**) to the highest level (**6**) of DNA depurination they produce. Reaction conditions: 8000 eq. of **16**, 8000 eq. of **17**, 50 eq. of **I/II**, 0.5 mM **I/II**, 40 °C, 21 h.

DNA depurination ranking	copolymer name	copolymer composition
**1**	**ID**	DMA_{62}-(OA_{26}-SPA_2)
	**IIE**	(DMA_{62}-SPA_2)-DDA_{12}
	**IID**	(DMA_{62}-SPA_2)-OA_{26}
**2**	**IC$_a$**	DMA_{65}-(BA_{33}-SPA_2)
	**IIC$_a$**	(DMA_{63}-SPA_2)-BA_{33}
**3**	**IB**	DMA_{62}-(EA_{23}-SPA_2)
	**IC$_b$**	DMA_{63}-(BA_{27}-SPA_2)
**4**	**IIA**	(DMA_{62}-SPA_2)-MMA_{14}
	**IA**	DMA_{62}-(MMA_{25}-SPA_2)
	**IIB**	(DMA_{60}-SPA_2)-EA_{23}
**5**	**IC$_c$**	DMA_{132}-(BA_{23}-SPA_2)
**6**	**IIC$_b$**	(DMA_{100}-SPA_2)-BA_{34}

[a] Based on the analysis of HPLC traces from Figures S84-S95.

6.1.1. DNA stability under conditions of copolymer micelle **I**/**II**-mediated Biginelli reaction of aldehyde conjugate **DNA-1**, urea **16**, and ethyl acetoacetate **17** to DNA-dihydropyrimidin-2(1*H*)-one conjugate **DNA-15** at room temperature

a)

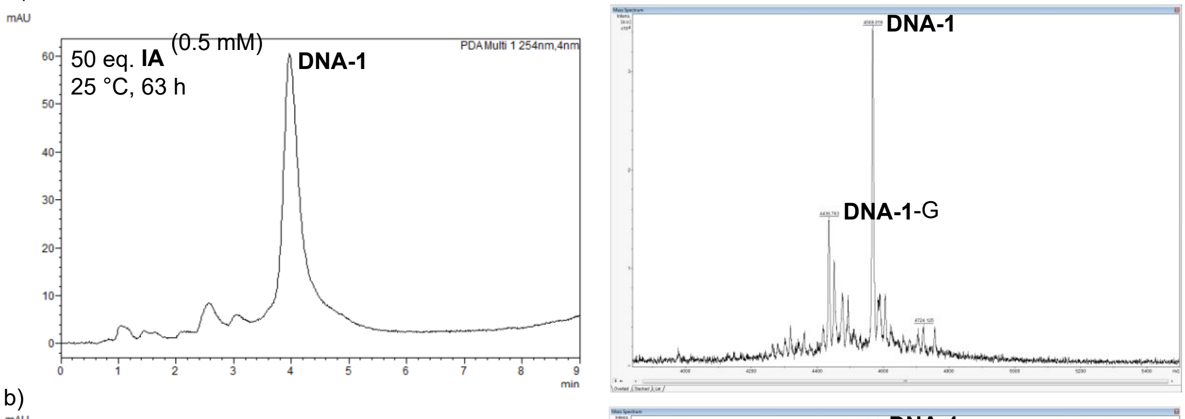

b)

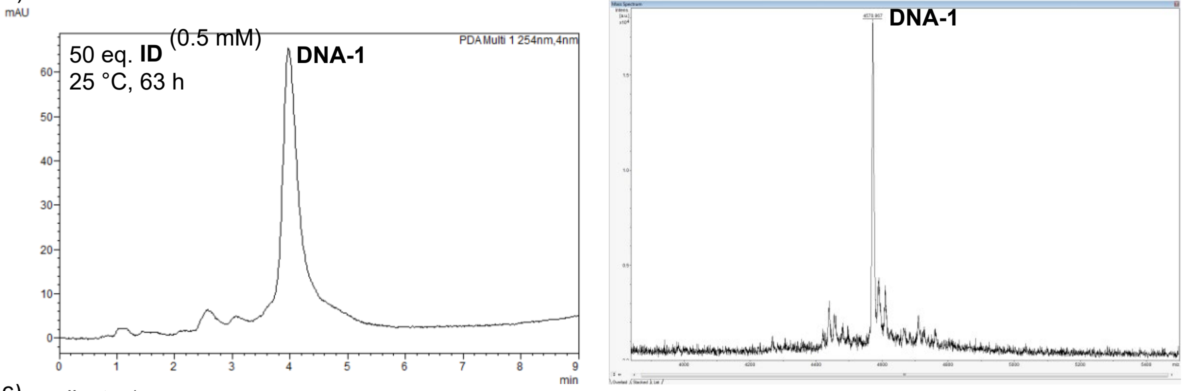

c) replicate 1x

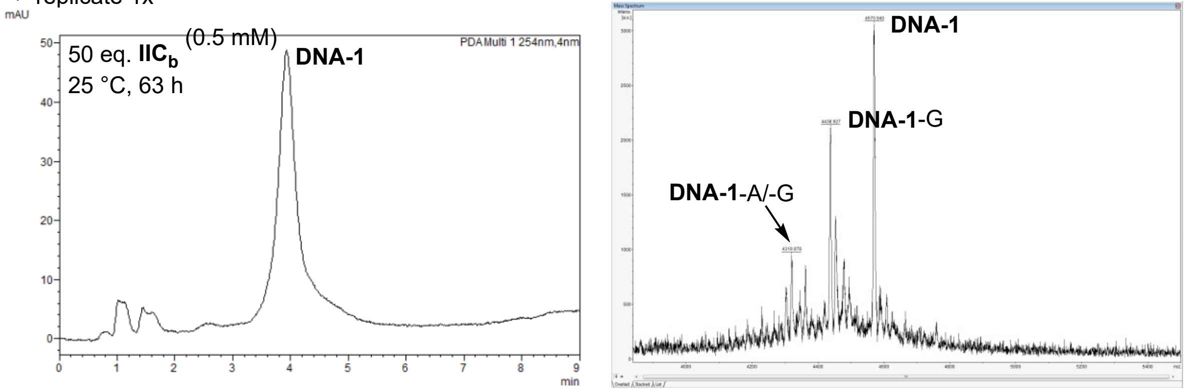

d) replicate 2x

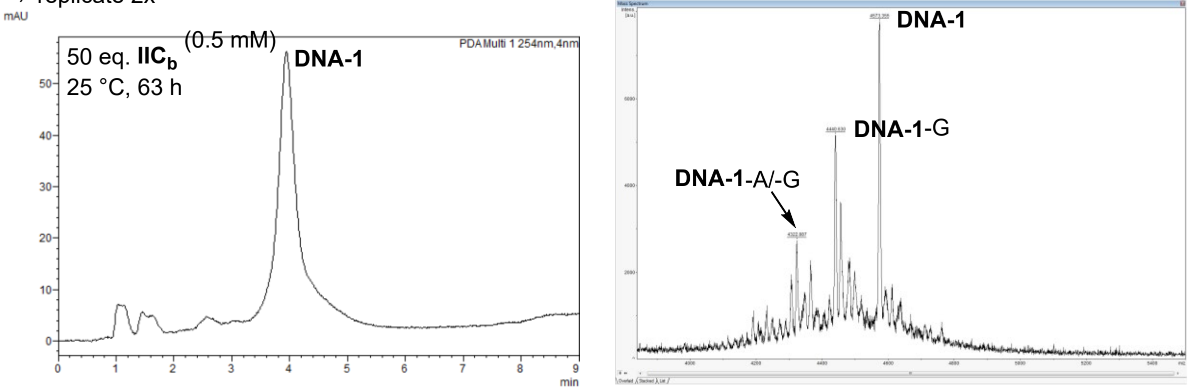

e) replicate 3x

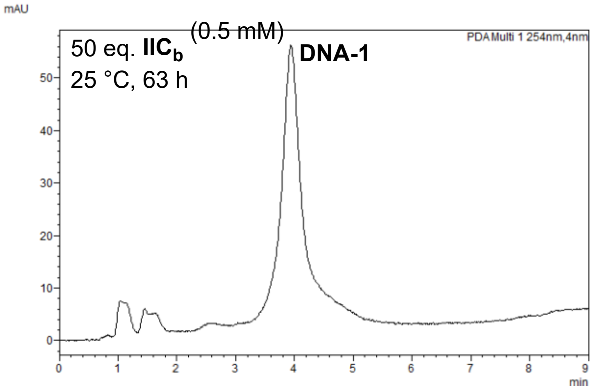

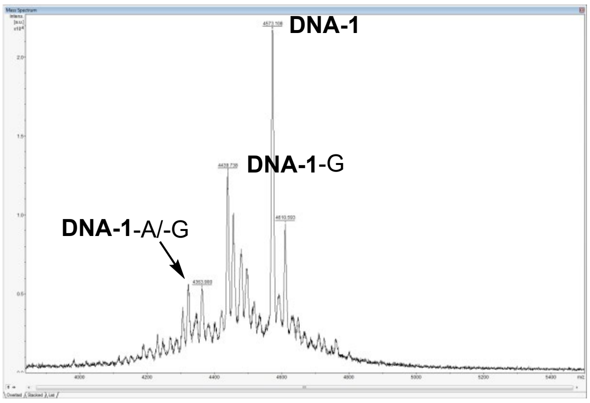

Figure S106. Comparison of different degree of DNA degradation in copolymer micelle **IA/ID/IIC$_b$**-mediated Biginelli reaction of the oligonucleotide-aldehyde conjugate **DNA-1**, urea **16**, and ethyl acetoacetate **17** at 25 °C for 63 hours. HPLC traces (left hand traces) and MALDI-MS spectra (right hand spectra) show: a) experiment with copolymer **IA**, b) experiment with copolymer **ID**, and c)-e) experiment with copolymer **IIC$_b$** in triplicate.

6.2. Optimization of copolymer micelle **ID**-mediated Biginelli reaction of aldehyde conjugate **DNA-1**, urea **16**, and ethyl acetoacetate **17** to DNA-dihydropyrimidin-2(1*H*)-one conjugate **DNA-15**

Table S21. Optimization of copolymer micelle **ID**-mediated Biginelli reaction of the oligonucleotide-aldehyde conjugate **DNA-1**, urea **16**, and ethyl acetoacetate **17** to DNA-dihydropyrimidin-2(1*H*)-one conjugate **DNA-15**.

No.	**16** [eq.][a]	**16** [mM][a]	**17** [eq.][a]	**17** [mM][a]	**ID** [eq.][a]	**ID** [mM]	**T** [°C]	time [h]	**DNA-15** [%][a]
1	8000	80	8000	80	50	0.5	40	63	77*
2	8000	80	8000	80	50	0.5	40	21	-
3	8000	80	8000	80	50	0.5	25	63	-
**4**	**8000**	**80**	**8000**	**80**	**25**	**0.25**	**40**	**63**	**56**
5	16000	160	8000	80	25	0.25	40	63	55
6	8000	80	4000	40	25	0.25	50	21	37*
7	4000	40	8000	80	25	0.25	50	21	40*
8	8000	80	4000	40	25	0.25	80	2	-*
9	4000	40	8000	80	25	0.25	80	2	-*
10	12000	120	8000	80	25	0.25	80	6	-*
11	16000	160	8000	80	25	0.25	80	6	-*

[a] HPLC analysis of the crude, missing percentage to 100%: mainly **DNA-1**. *Significant DNA depurination detected by HPLC and MALDI-MS.

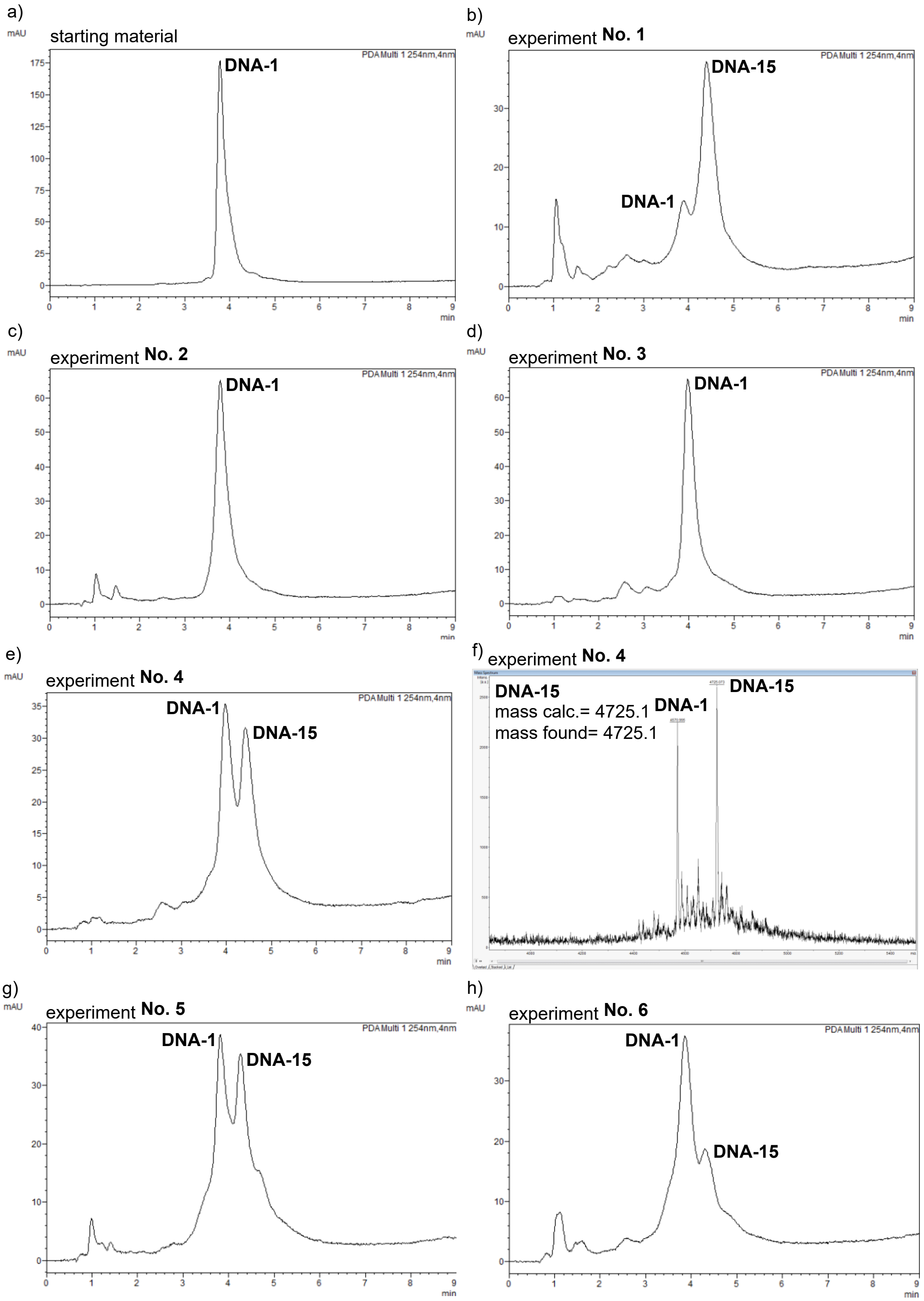
a)
starting material
DNA-1
b)
experiment No. 1
DNA-15
DNA-1
c)
experiment No. 2
DNA-1
d)
experiment No. 3
DNA-1
e)
experiment No. 4
DNA-1
DNA-15
f)
experiment No. 4
DNA-15
mass calc.= 4725.1
mass found= 4725.1
DNA-1
DNA-15
g)
experiment No. 5
DNA-1
DNA-15
h)
experiment No. 6
DNA-1
DNA-15
mAU
min
PDA Multi 1 254nm,4nm

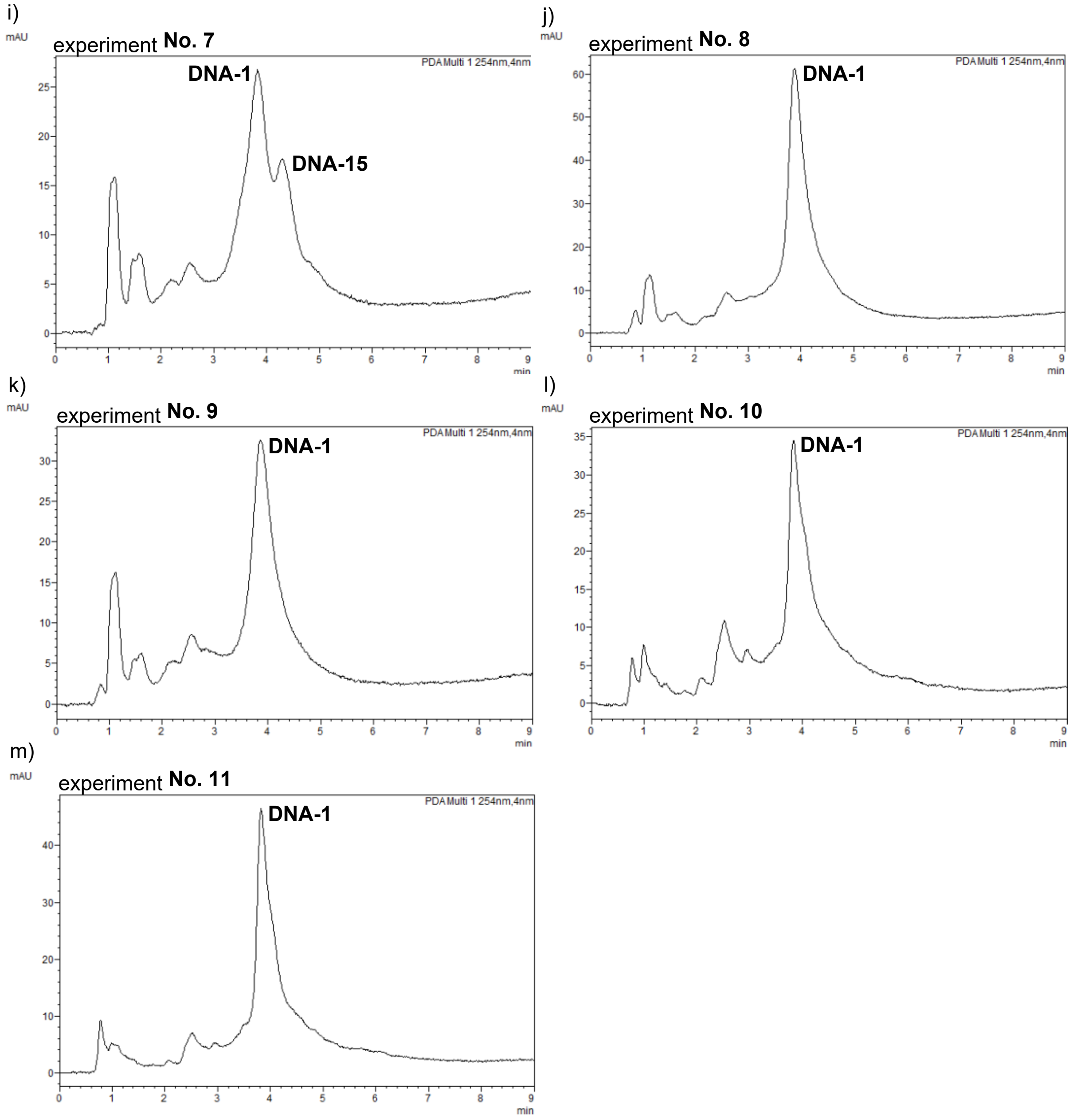

Figure S107. Effect of different reaction conditions on the copolymer micelle **ID**-mediated Biginelli reaction of the oligonucleotide-aldehyde conjugate **DNA-1**, urea **16**, and ethyl acetoacetate **17**, for reaction conditions see Table S21. HPLC traces show oligonucleotide-aldehyde conjugate **DNA-1** and experiments **No. 1** - **No. 11**. MALDI-MS spectrum shows experiment **No. 4**.

6.3. Negative control experiments for copolymer micelle **ID**-mediated Biginelli reaction to **DNA-15**

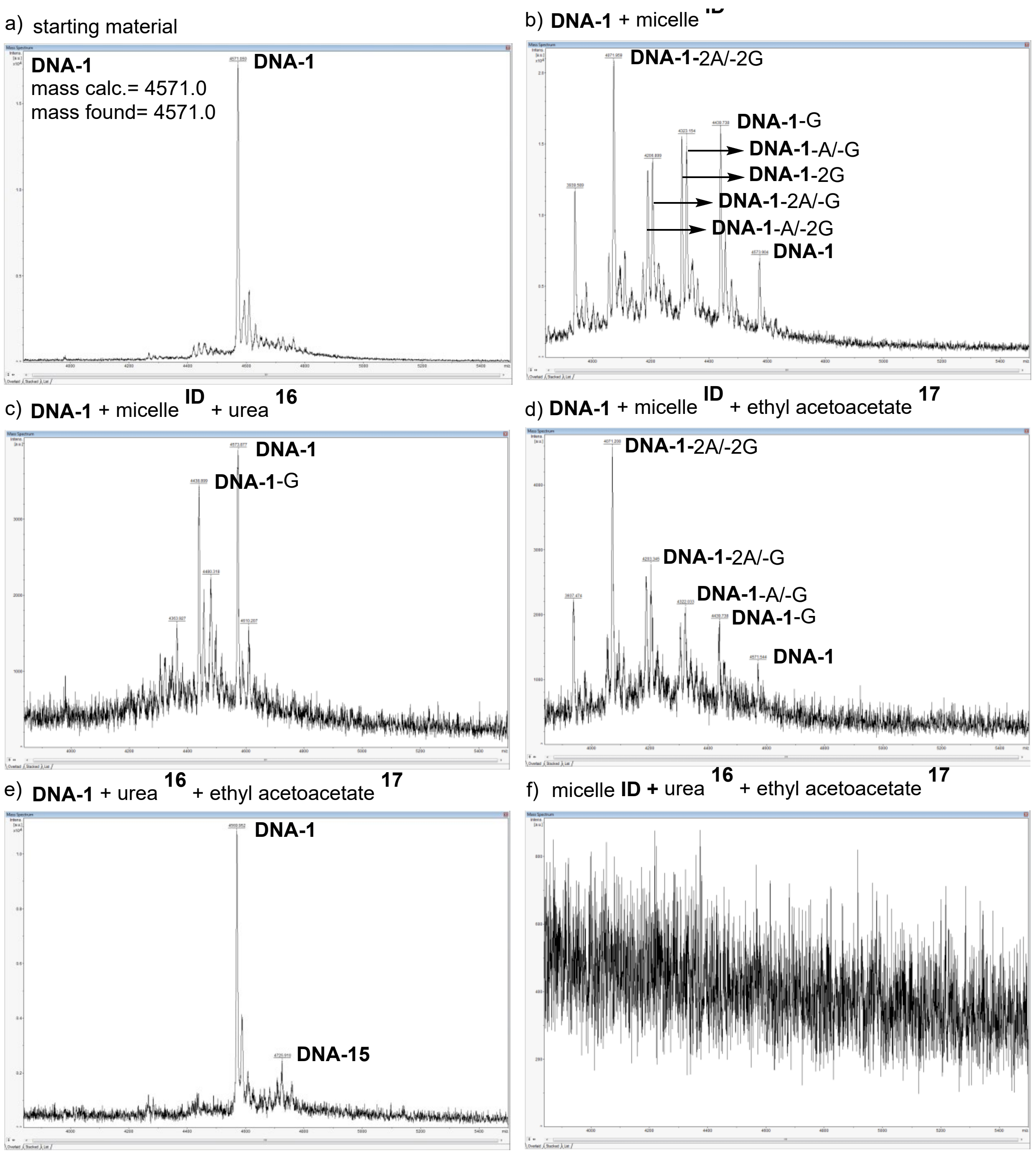

Figure S108. Negative control experiments for copolymer micelle **ID**-mediated Biginelli reaction of the oligonucleotide-aldehyde conjugate **DNA-1**, urea **16**, and ethyl acetoacetate **17** for the synthesis of **DNA-15**. If not excluded, the reactants were used in the following amounts: 25 eq. of copolymer **ID** (0.25 mM), 8000 eq. of reactant **16** and 8000 eq. of reactant **17**. The reactions were running for 63 hours at 40 °C. MALDI-MS spectra show oligonucleotide-aldehyde conjugate **DNA-1** and the systematical exclusion of reactants from the reaction.

6.4. Exploring sulfonic acid un-substituted copolymer micelle **III** for the synthesis of **DNA-15**

a)

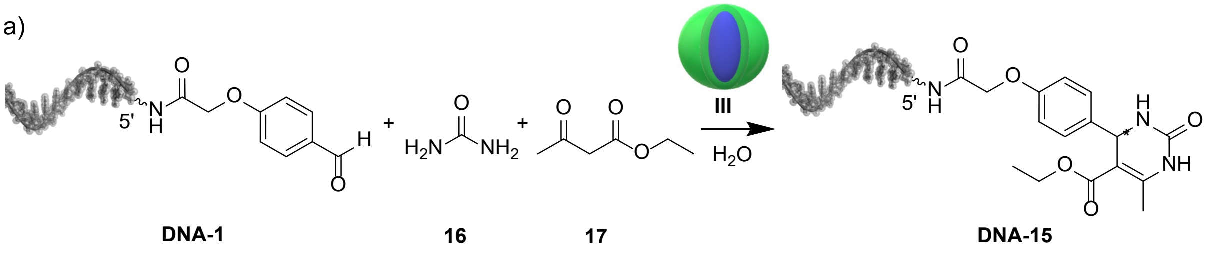

b)

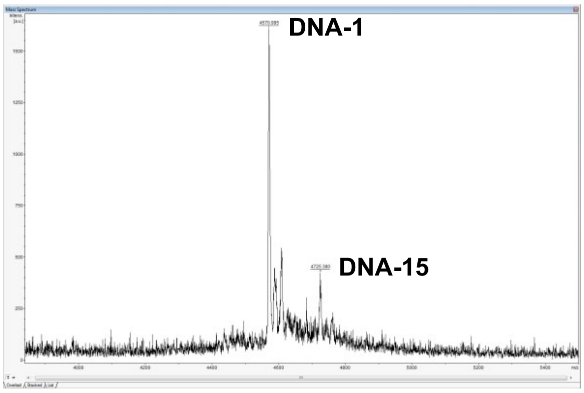

Figure S109. Exploring sulfonic acid un-substituted copolymer micelle **III** for the synthesis of **DNA-15**. a) Reaction scheme of the synthesis of **DNA-15**; conditions: 25 eq. of copolymer **III** (0.25 mM), 8000 eq. of reactant **16** and 8000 eq. of reactant **17**, water, 40 °C, 63 h. b) MALDI-MS spectrum shows experiment with copolymer micelle **III**.

6.5. Comparison of the copolymer micelle **ID**-mediated Biginelli reaction to **DNA-15** with Biginelli reaction on controlled-pore glass (CPG)-bound DNA to **DNA-15**

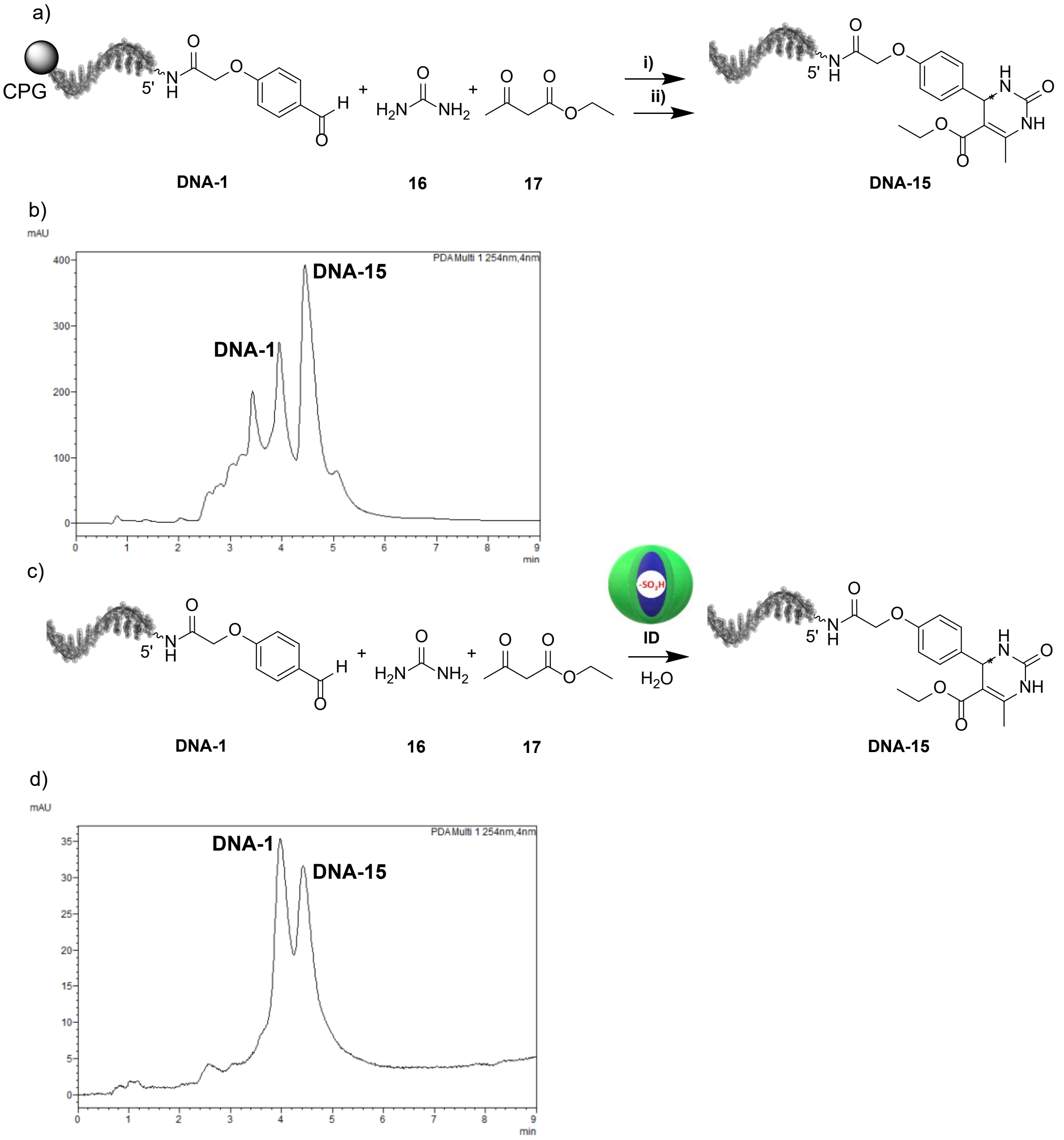

Figure S110. Comparison of the copolymer micelle **ID**-mediated Biginelli reaction with Biginelli reaction on controlled-pore glass (CPG)-bound DNA. a) Reaction scheme of the Biginelli reaction of the CPG-bound oligonucleotide-aldehyde conjugate **DNA-1**, urea **16**, and ethyl acetoacetate **17** to **DNA-15**; conditions: i) 50 eq. of (*R*)-(-)-BNDHP, 500 eq. of reactant **16** and 500 eq. of reactant **17**, ethanol, 50 °C, 20 h;[4] ii) AMA (aqueous ammonia (30%)/ aqueous methylamine (40%), 1:1, vol/vol), 4 h, room temperature. b) HPLC trace shows the control experiment on the CPG-coupled DNA. c) Reaction scheme of the copolymer micelle **ID**-mediated Biginelli reaction of oligonucleotide-aldehyde conjugate **DNA-1**, urea **16**, and ethyl acetoacetate **17** to **DNA-15**; conditions: 25 eq. of copolymer **ID** (0.25 mM), 8000 eq. of reactant **16** and 8000 eq. of reactant **17**, water, 40 °C, 63 h. d) HPLC trace shows the experiment with micelle **ID** under the optimal reaction conditions (Table S21, **No. 4**, Figure S107e)).

6.6. Copolymer micelle **ID**-mediated Biginelli reaction on DNA-coupled aldehydes: aldehyde scope

a)

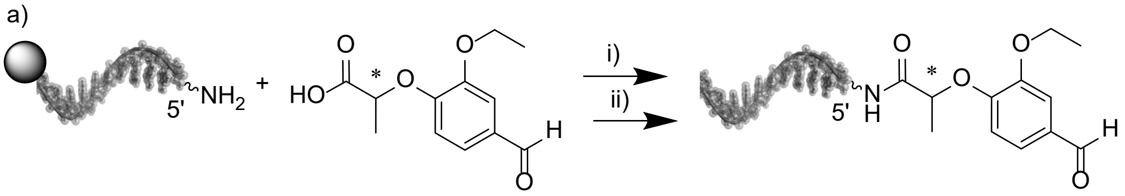

DNA-1 **23** **DNA-1b**

b)

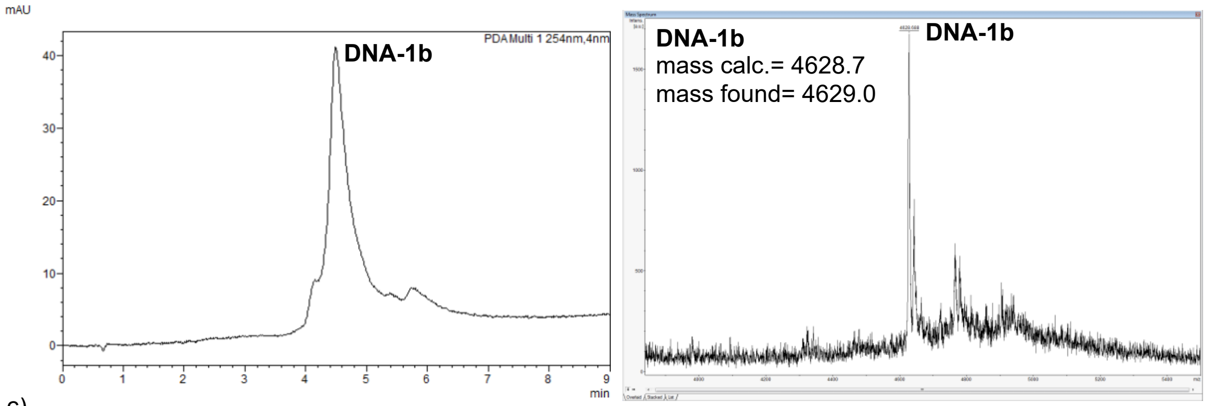

c)

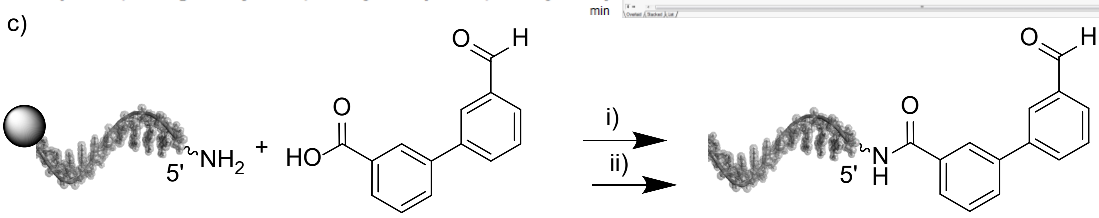

DNA-1 **24** **DNA-1c**

d)

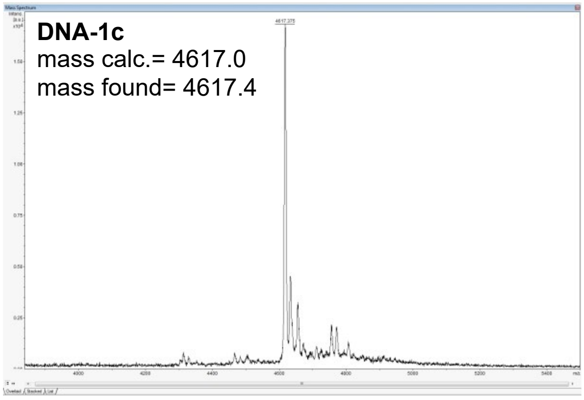

e)

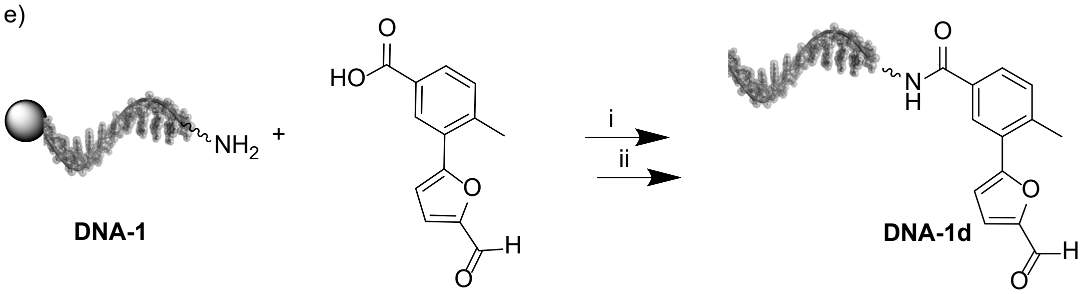

DNA-1 **DNA-1d**

f)

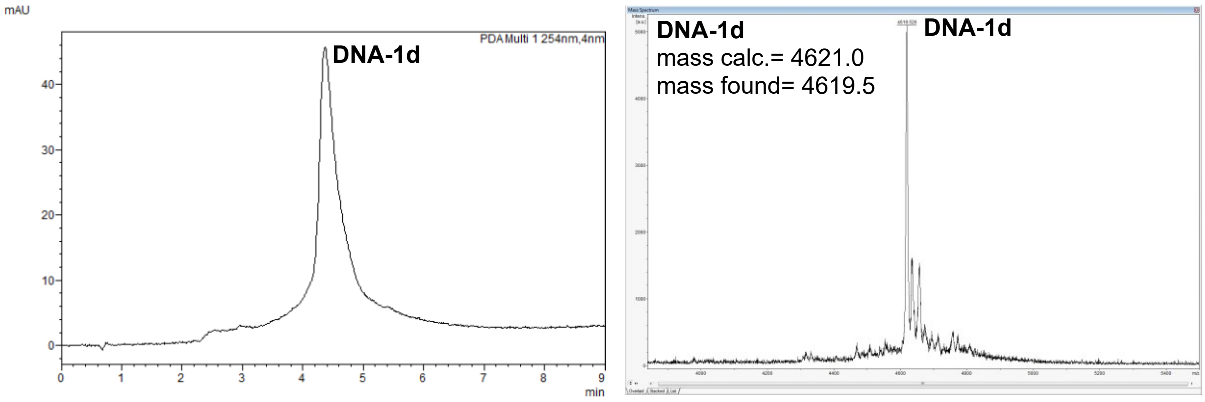

g)

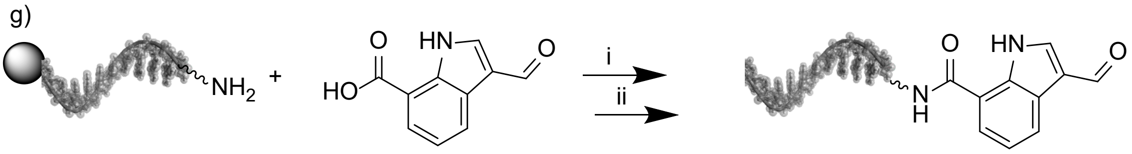

DNA-1 **26** **DNA-1e**

h)

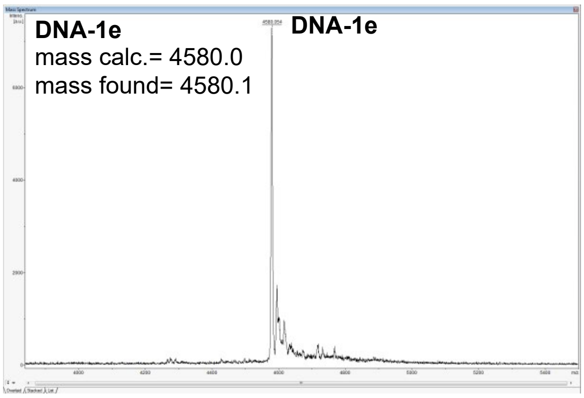

Figure S111. Synthesis of DNA-aldehyde conjugates **DNA-1b-1e**. a) Scheme of the synthesis of **DNA-1b**. b) HPLC trace of the aldehyde **DNA-1b** (left hand trace) and MALDI-MS spectrum of the aldehyde **DNA-1b** (right hand trace). c) Scheme of the synthesis of **DNA-1c**. d) MALDI-MS spectrum of the aldehyde **DNA-1c**. e) Scheme of the synthesis of **DNA-1d**. f) HPLC trace of the aldehyde **DNA-1d** (left hand trace) and MALDI-MS spectrum of the aldehyde **DNA-1d** (right hand trace). g) Scheme of the synthesis of **DNA-1e**. h) MALDI-MS spectrum of the aldehyde **DNA-1e**. Reagents and conditions: i) HATU, DIPEA, dry DMF, room temperature, 2 x 4 h; ii) AMA (aqueous ammonia (30%)/ aqueous methylamine (40%), 1:1, vol/vol), 4 h, room temperature.

a)

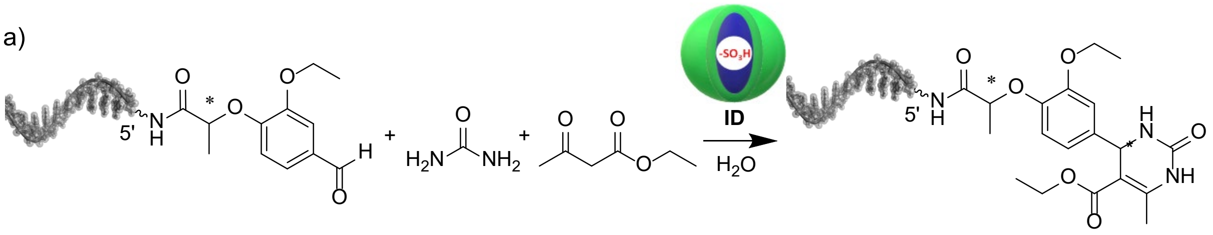

DNA-1b **16** **17** **DNA-15b**

b)

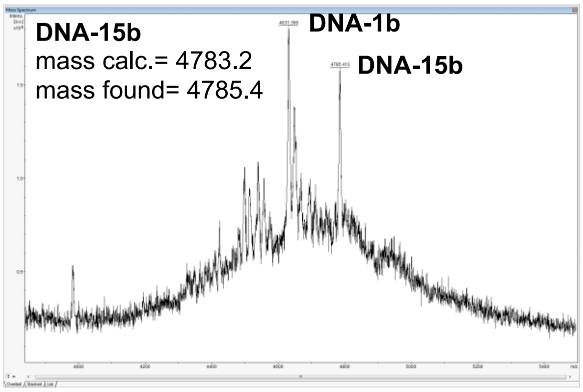

Figure S112. Reaction to DNA-dihydropyrimidin-2(1*H*)-one conjugate **DNA-15b**. a) Scheme for the synthesis of the DNA conjugate **DNA-15b**; reaction conditions **No. 4** (Table S21). b) MALDI-MS spectrum of the crude conjugate **DNA-15b**.

a)

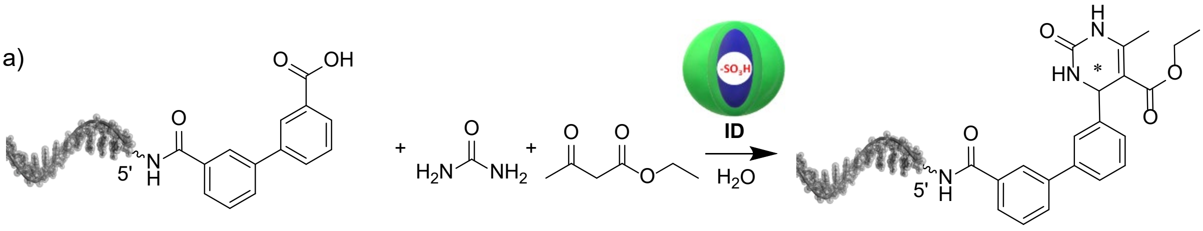

DNA-1c **16** **17** **DNA-15c**

b)

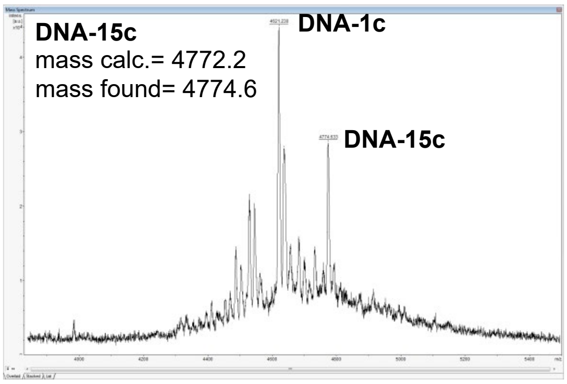

Figure S113. Reaction to DNA-dihydropyrimidin-2(1*H*)-one conjugate **DNA-15c**. a) Scheme for the synthesis of the DNA conjugate **DNA-15c**; reaction conditions **No. 4** (Table S21). b) MALDI-MS spectrum of the crude conjugate **DNA-15c**.

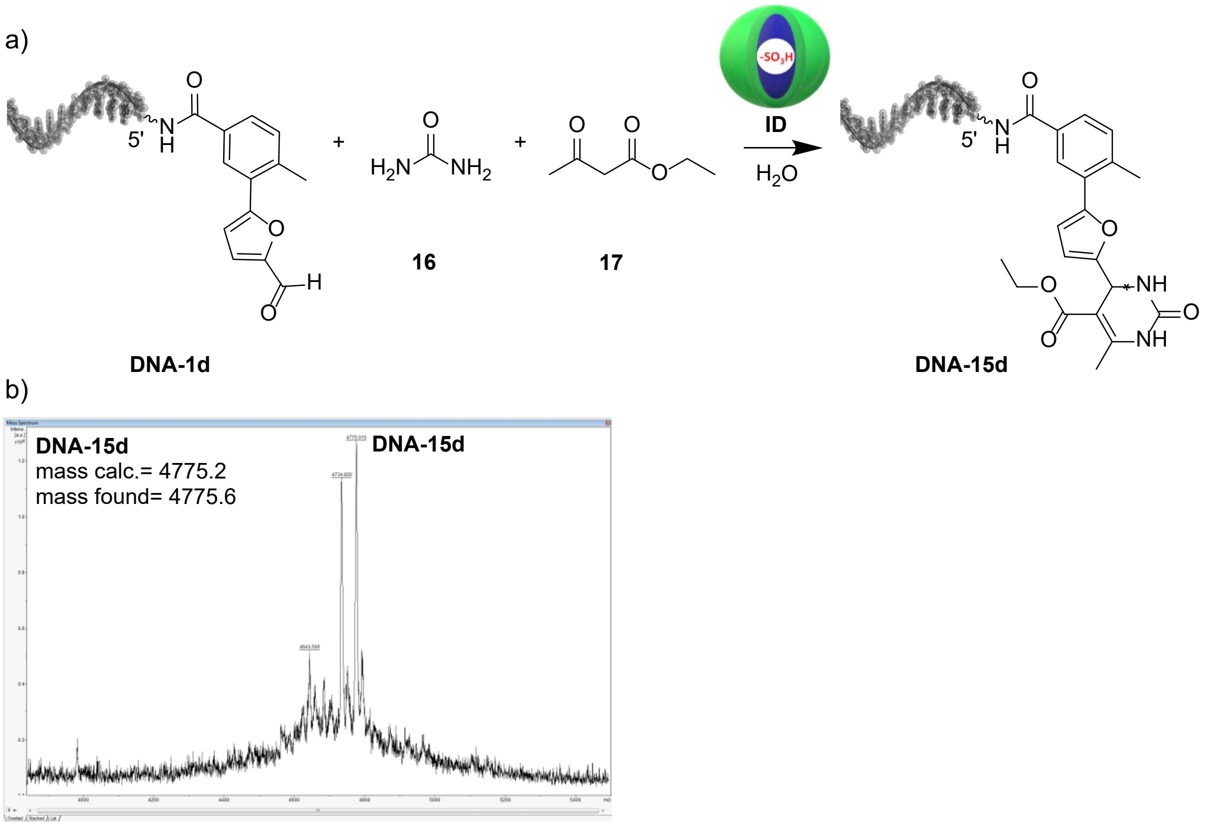

Figure S114. Reaction to DNA-dihydropyrimidin-2(1*H*)-one conjugate **DNA-15d**. a) Scheme for the synthesis of the DNA conjugate **DNA-15d**; reaction conditions **No. 4** (Table S21). b) MALDI-MS spectrum of the crude conjugate **DNA-15d**.

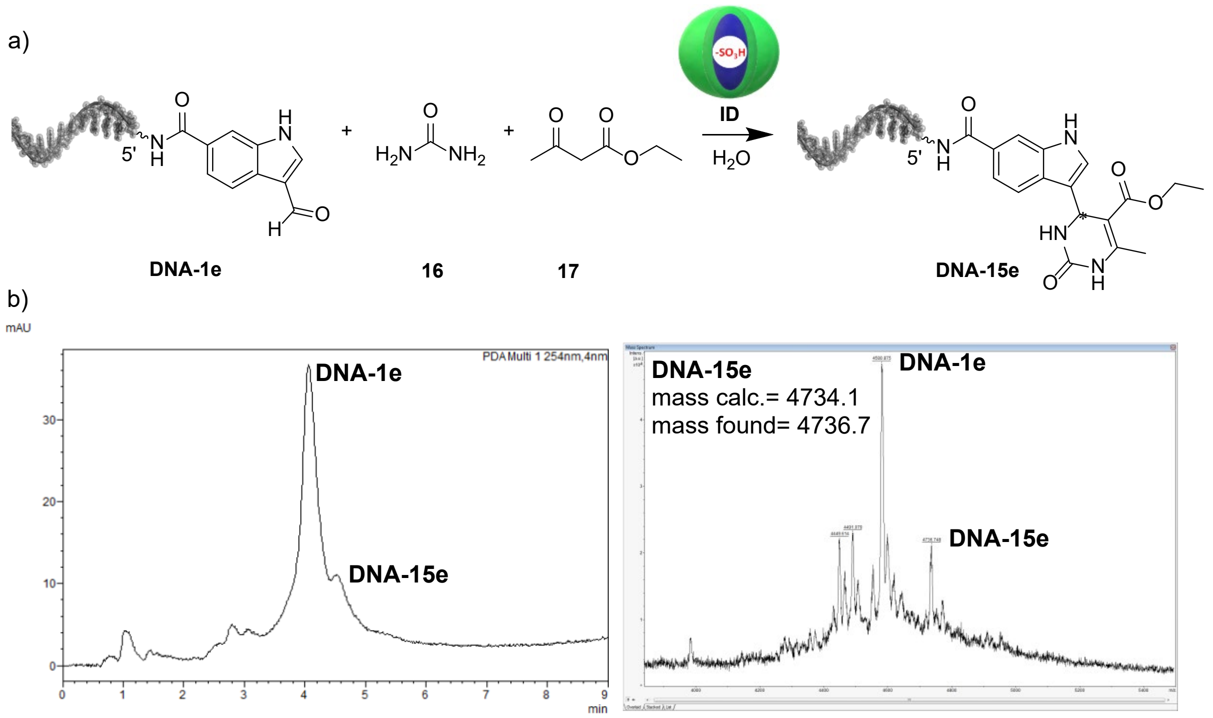

Figure S115. Reaction to DNA-dihydropyrimidin-2(1*H*)-one conjugate **DNA-15e**. a) Scheme for the synthesis of the DNA conjugate **DNA-15e**; reaction conditions **No. 4** (Table S21). b) MALDI-MS spectrum of the crude conjugate **DNA-15e**.

7. Impact of DNA chemical modification on copolymer micelle **I/II**-mediated synthesis of DNA-hexahydro-1*H*-pyrrolo[3,2-*c*]quinoline conjugates **DNA-18-21**

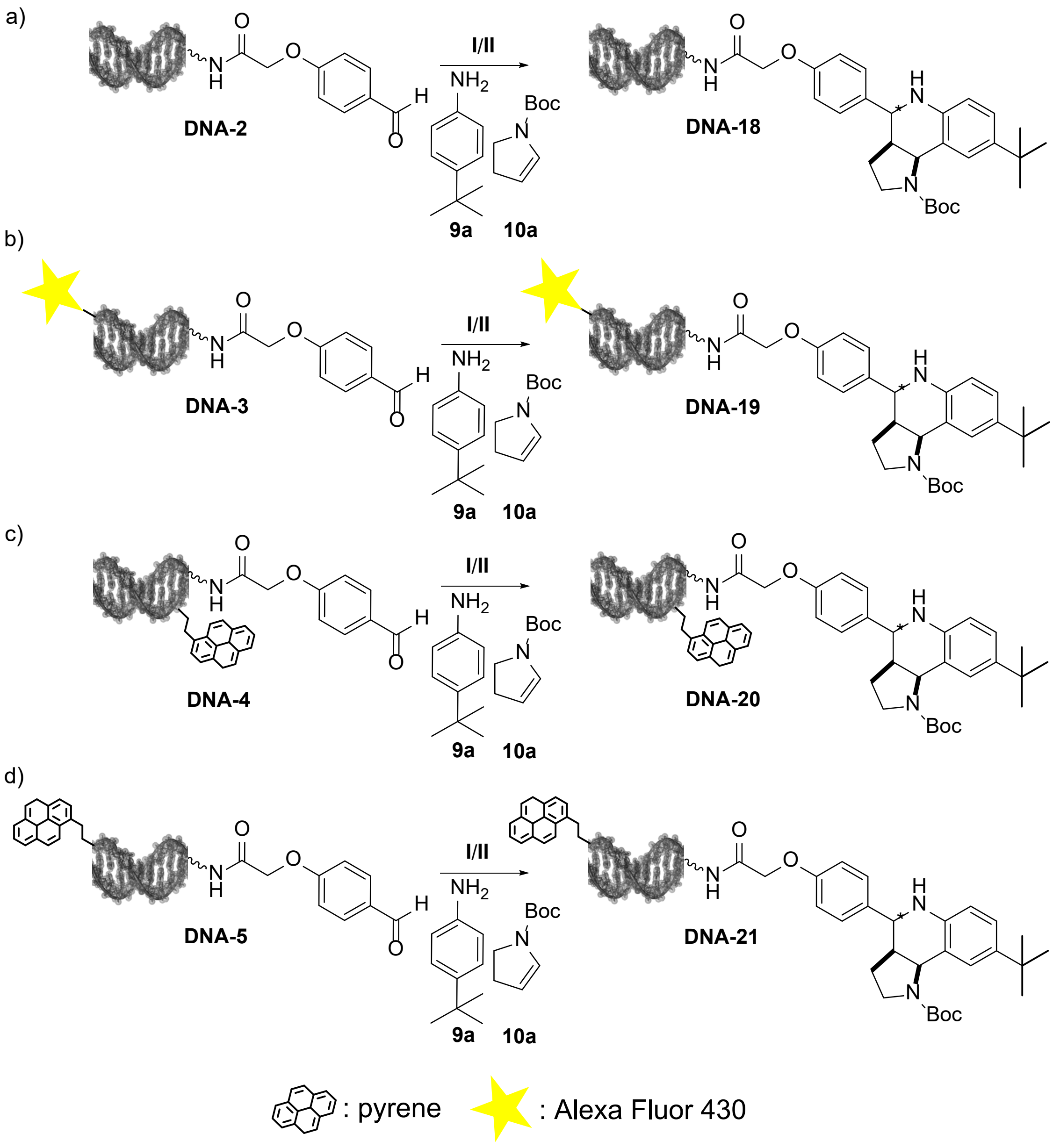

Scheme S12. Synthesis of DNA-hexahydro-1*H*-pyrrolo[3,2-*c*]quinoline conjugates **DNA-18-21** from DNA-conjugated aldehydes **DNA-2-5** by the copolymer micelle **I/II**-mediated Povarov reaction. a) Synthesis of dsDNA heterocycle **DNA-18**. b) Synthesis of 5`-Alexa-labeled dsDNA heterocycle **DNA-19**. c) Synthesis of 3`-pyrene-labeled dsDNA heterocycle **DNA-20**. d) Synthesis of 5`-pyrene-labeled dsDNA heterocycle **DNA-21**.

Table S22. Impact of DNA chemical modification on synthesis of DNA-hexahydro-1*H*-pyrrolo[3,2-*c*]quinoline conjugates **DNA-18-21**. Reaction conditions: 2000 eq. of **9a** and 2000 eq. of **10a**, 50 eq. of **I/II**, 0.5 mM **I/II**, room temperature, 1 h.

No.	copolymer name	copolymer composition	**DNA-18** [%][a]	**DNA-19** [%][a]	**DNA-20** [%][a]	**DNA-21** [%][a]
1	**IB**	DMA_{62}-(EA_{23}-SPA_2)	81	39(36)	86	85
2	**IC$_b$**	DMA_{63}-(BA_{27}-SPA_2)	67(13)	45(41)	78(10)	74(11)
3	**IIB**	(DMA_{60}-SPA_2)-EA_{23}	66(17)	41(35)	72(11)	73(8)
4	**IIE**	(DMA_{62}-SPA_2)-DDA_{12}	52(22)	36(28)	57(18)	57(19)

[a] HPLC analysis of the crude, missing percentage to 100%: mainly starting material **DNA-2-5**. Conversions in parentheses show those of a later eluting side product.

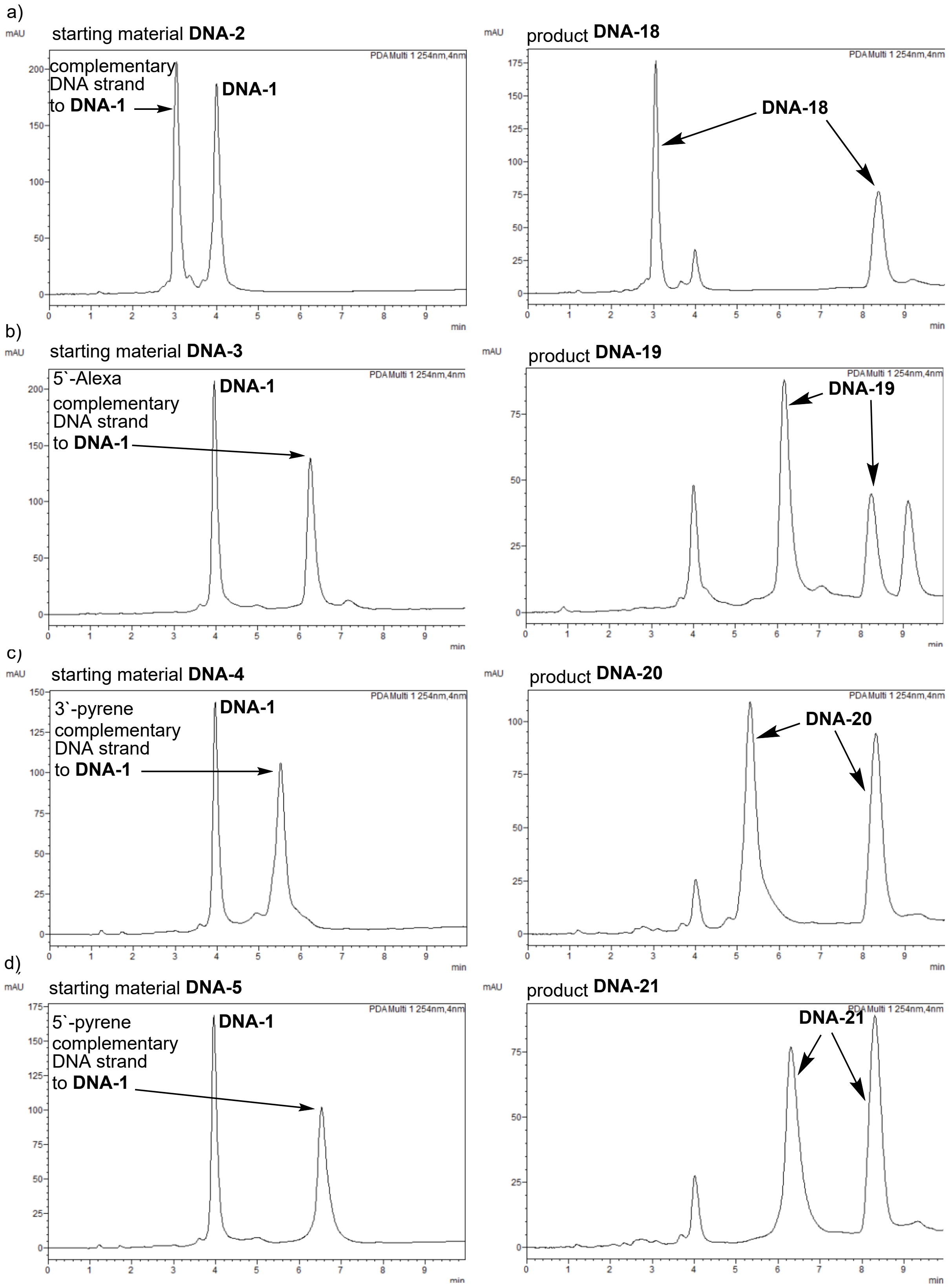

Figure S116. Copolymer **IB**-mediated Povarov reactions of oligonucleotide-aldehyde conjugates **DNA-2-5**, 4-*tert*-butylaniline **9a**, and *N*-Boc-2,3-dihydro-1*H*-pyrrole **10a** at 25 °C, for reaction conditions see Table S22. HPLC traces show oligonucleotide-aldehyde conjugates **DNA-2-5** (left-hand traces) and experiments **No. 1** (right-hand traces).

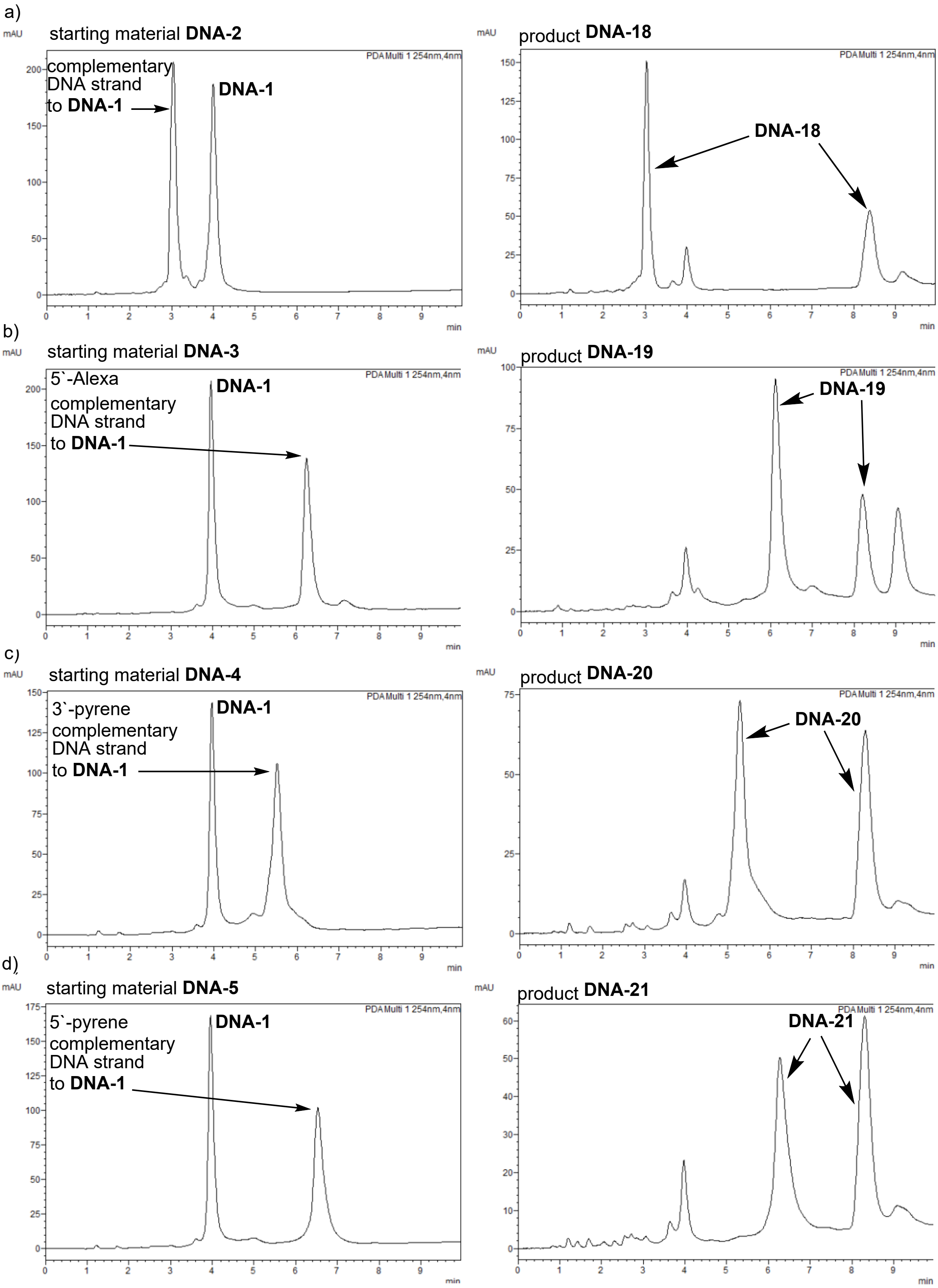

Figure S117. Copolymer **IC$_b$**-mediated Povarov reactions of oligonucleotide-aldehyde conjugates **DNA-2-5**, 4-*tert*-butylaniline **9a**, and *N*-Boc-2,3-dihydro-1*H*-pyrrole **10a** at 25 °C, for reaction conditions see Table S22. HPLC traces show oligonucleotide-aldehyde conjugates **DNA-2-5** (left-hand traces) and experiments **No. 2** (right-hand traces).

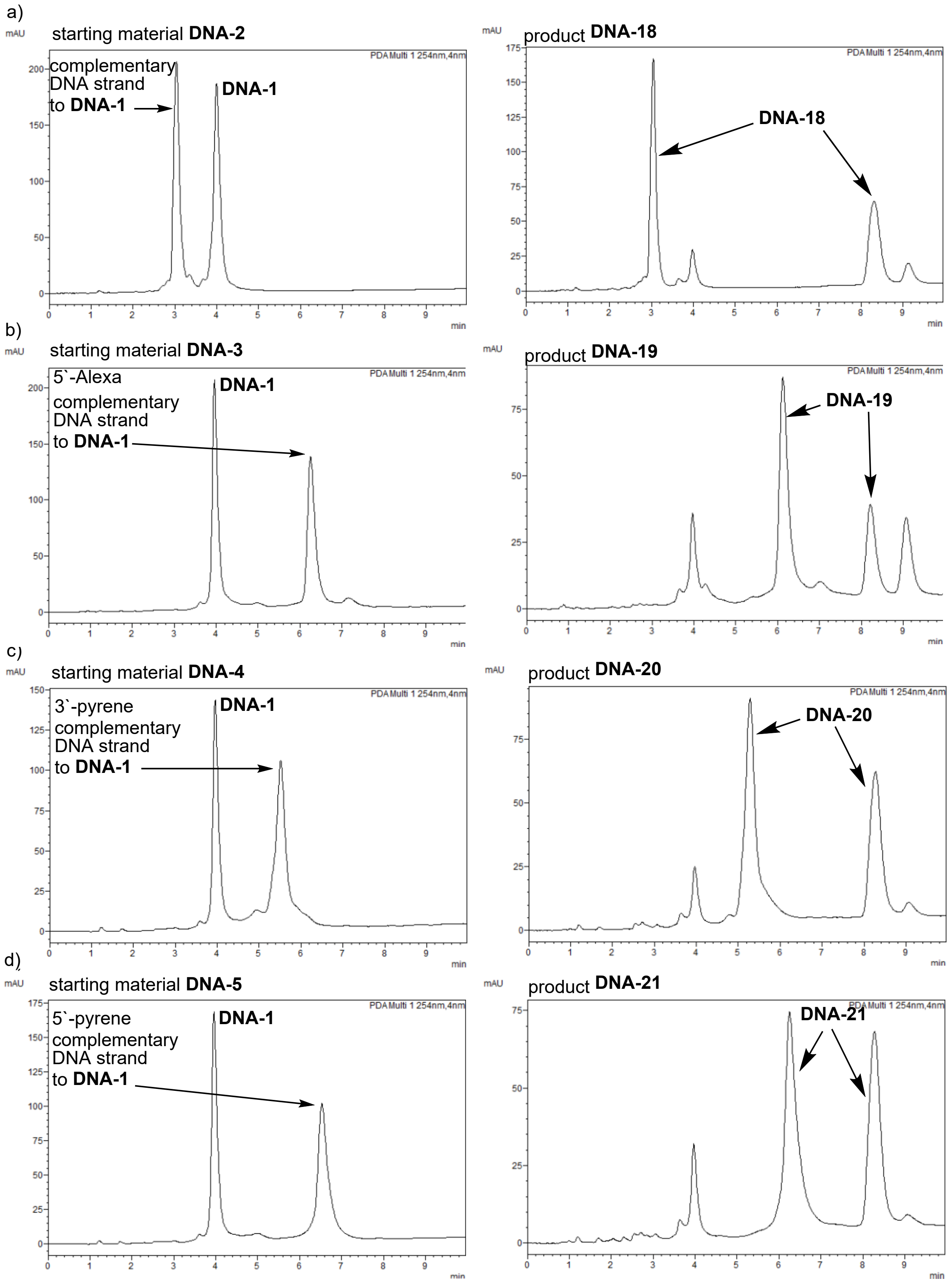

Figure S118. Copolymer **IIB**-mediated Povarov reactions of dsDNA-aldehyde conjugates **DNA-2-5**, 4-*tert*-butylaniline **9a**, and *N*-Boc-2,3-dihydro-1*H*-pyrrole **10a** at 25 °C, for reaction conditions see Table S22. HPLC traces show oligonucleotide-aldehyde conjugates **DNA-2-5** (left-hand traces) and experiments **No. 3** (right-hand traces).

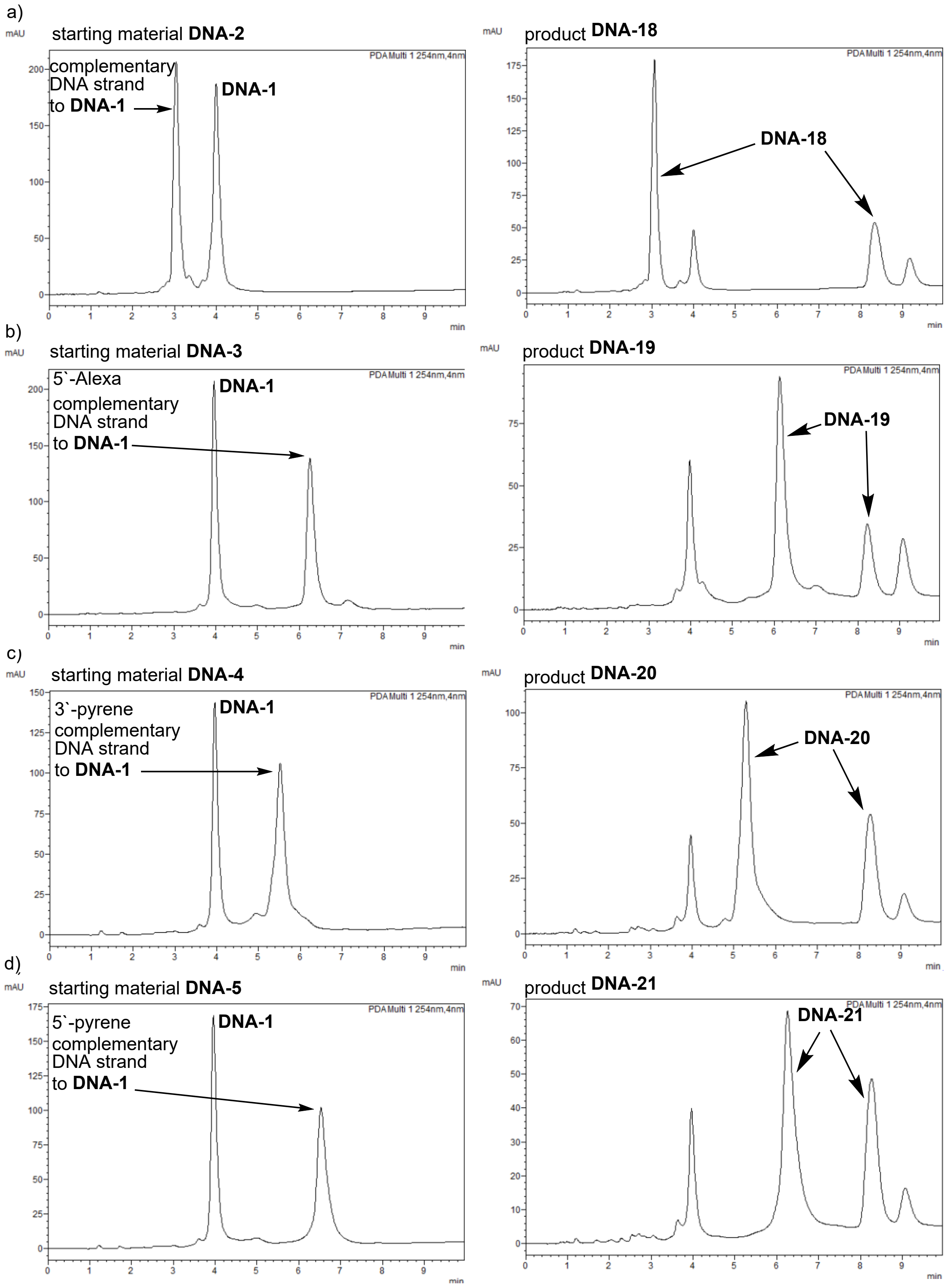

Figure S119. Copolymer **IIE**-mediated Povarov reactions of oligonucleotide-aldehyde conjugates **DNA-2-5**, 4-*tert*-butylaniline **9a**, and *N*-Boc-2,3-dihydro-1*H*-pyrrole **10a** at 25 °C, for reaction conditions see Table S22. HPLC traces show oligonucleotide-aldehyde conjugates **DNA-2-5** (left-hand traces) and experiments **No. 4** (right-hand traces).

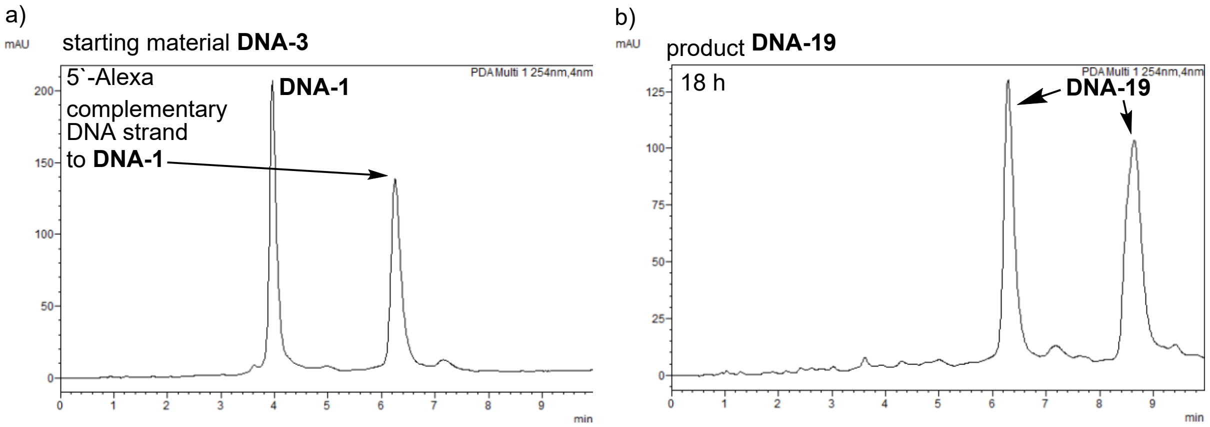

Figure S120. Copolymer **IIE**-mediated Povarov reaction of oligonucleotide-aldehyde conjugate **DNA-3**, 4-*tert*-butylaniline **9a**, and *N*-Boc-2,3-dihydro-1*H*-pyrrole **10a** at 25 °C; conditions: 50 eq. of copolymer **IIE** (0.5 mM), 8000 eq. of reactant **9a** and 8000 eq. of reactant **10a**, 25 °C, 18 h. HPLC traces show oligonucleotide-aldehyde conjugate **DNA-3** (left-hand trace) and Povarov reaction on 5´-Alexa-labelled dsDNA after 18 hours reaction time.

8. Characterization of partitioning of DNA conjugates to copolymer micelles **IIB**

8.1. X-ray Small-angle Scattering (SAXS)

SAXS measurements at ambient pressure at 25 °C were carried out on an Anton Paar SAXSess mc^2 (Graz, Austria) using a monochromatic X-ray beam (λ = 1.54 Å) and an image plate detector. A 6 mM suspension of micelles composed of copolymer **IIB** in the absence and in the presence of 50 µM dsDNA with linker and benzaldehyde moiety **DNA-2** in a volume of 20 µL was loaded into a quartz capillary (µCell of Anton Paar). Scattering patterns were collected over 30 min per image and the data were treated and analyzed with 2D-SAXSquant and SAXS Quant. Further, the background of the capillary and buffer was subtracted from the data and the scattering curves were desmeared with the scattering length profile. Silver behenate was used to calibrate the momentum transfer Q of the X-rays, which is given by $Q = (4\pi/\lambda)\sin\theta$, where λ is the wavelength of the X-rays and 2θ is the scattering angle. The pair distance distribution function, $p(r)$, which depends on the molecular particle shape and on the intra-particle scattering distribution, is given by the indirect Fourier transform of the measured scattered intensity, $I(Q)$. For a particle of uniform electron density, it is given by

$$p(r) = \frac{1}{2\pi^2}\int_0^\infty I(Q)Qr\sin(Qr)\mathrm{d}Q \qquad (1)$$

The function $p(r)$ represents the frequency of vector length r connecting small volume elements within the volume of the scattering particle, that is, the protein molecule, with maximum dimension $D_{\max}$, and allows to characterize the size and shape of the scattering particle. The $p(r)$ function was calculated by the method of Svergun using the ATSAS software package D.[5]

8.2. Steady-State Fluorescence Spectroscopy

Fluorescence quenching experiments were performed by means of a K2 fluorimeter from ISS (Champaign, Illinois, USA) equipped with a xenon arc lamp as light source. The experiments were performed at the temperature of 25 °C by using a 1 cm path length quartz cuvette with a volume of 400 µL. A solution of ~1 µM of double-stranded (ds) DNA-aldehyde conjugate **DNA-3** and aminolinker DNA **DNA-7**, both labeled with Alexa Fluor 430, in the absence or in the presence of 250 µM of the copolymer particle **IIB**, was titrated with a KI solution (~ 4.5 M). The excitation wavelength was set to 430 nm and emission spectra were collected from 480 nm to 660 nm. The slit widths for both the excitation and and emission were set to 8 nm. The data obtained were analysed by using the Stern-Volmer equation: $F_0/F = 1+K_{SV}\cdot[Q]$, where F_0 is the fluorescence intensity of labeled DNA in the absence of quencher, F is the fluorescence intensity at each step of the titration in the presence of quencher, [Q] is the quencher concentration (i.e. of I^-) and K_{SV} is the Stern-Volmer constant which depends on the fluorescence lifetime and the rate constant of the quencher.

Fluorescence emission spectra of pyrene labeled dsDNAs **DNA-9-12** were recorded by means of K2 fluorometer, from ISS (Champaign, IL, USA). A series of solution containing 6.4 µM of labeled dsDNA in the absence and in the presence of 210 µM **IIB** copolymers were prepared. Then, the fluorescence spectra were recorded with an excitation wavelength of 273 nm. The emission spectra were recorded in the range 330-520 nm. The slits of both the excitation and emission monochromators were set at 8 nm. The temperature was set at 25 °C and a 0.3 cm path length quartz cuvette (final volume of 60 µL) was used.

8.3. UV/Vis Spectroscopy

UV/Vis spectroscopy experiments were performed by means of a UV-1800 spectrophotometer (Shimdazu Corporation, Kyoto, Japan) at the temperature of 25 °C. Spectra of dsDNA with the linker **DNA-6** at the concentration of 3 µM were acquired in the absence and in the presence of 250 µM of the copolymeric particle **IIB** by using a 0.3 cm path length quartz cuvette. Instead, the spectra of **DNA-8** at 11.5 µM in the absence and in the presence of copolymeric particle **IIB** were acquired using a quartz cuvette with a path length of 0.1 cm. As a blank, a spectrum of the copolymer solution at the same concentration and conditions was subtracted. Then, spectra of the copolymer at the concentration of 250 µM in the absence and in the presence of 2.3 µM dsDNA with the linker **DNA-6** were recorded. In this case, a blank composed of a solution of **DNA-6** at the same concentration was subtracted. In the range 240-300 nm, due to the absorbance of DNA bases, information about the DNA

molecule can be obtained. In the wavelength range 270-360 nm, it is possible to follow the absorption of the trithiocarbonate moiety (S-(C=S)-S) of the copolymer chain.

8.3.1 Determination of the Mole-fraction Partition Constant (K_x)

In order to determine the partition constant of the aminolinker dsDNA **DNA-6**, UV/Vis spectroscopy was applied. Briefly, a solution of the sample at the concentration of 3 µM was titrated with a 1 mM solution of the copolymer and spectra were recorded in the range 230-400 nm. Then, from each spectrum a blank with copolymer **IIB** solution only was subtracted. Each spectrum was corrected for the dilution factor. Then, a plot of A/A_0, where A is the absorbance of the DNA at 267 nm at each step of titration and A_0 is its absorbance in the absence of the copolymer, versus the copolymer concentration was performed. The experimental data were analysed as previously described to obtain the value of the mole-fraction partition constant, K_x.[6]

8.4. Circular Dichroism Spectroscopy

Circular dichroism spectroscopy spectra were acquired on a Jasco J-715 (Jasco Corporation, Tokyo, Japan). The experiments were performed at 25 °C using a quartz cuvette with a path length of 0.1 cm. The following instrumental parameters were set: scan rate 50 nm min^{-1}, 2 s response time and 5 nm bandwidth. Spectra solutions of **DNA-6** (8.2 µM) and **DNA-8** (7.8 µM) in the absence and in the presence of 250 µM copolymer micelles **IIB** and 20 mM NaCl were recorded. From each sample, a blank (water, NaCl solution and copolymer solution) was subtracted. All the spectra recorded were normalized per concentration of single strand.

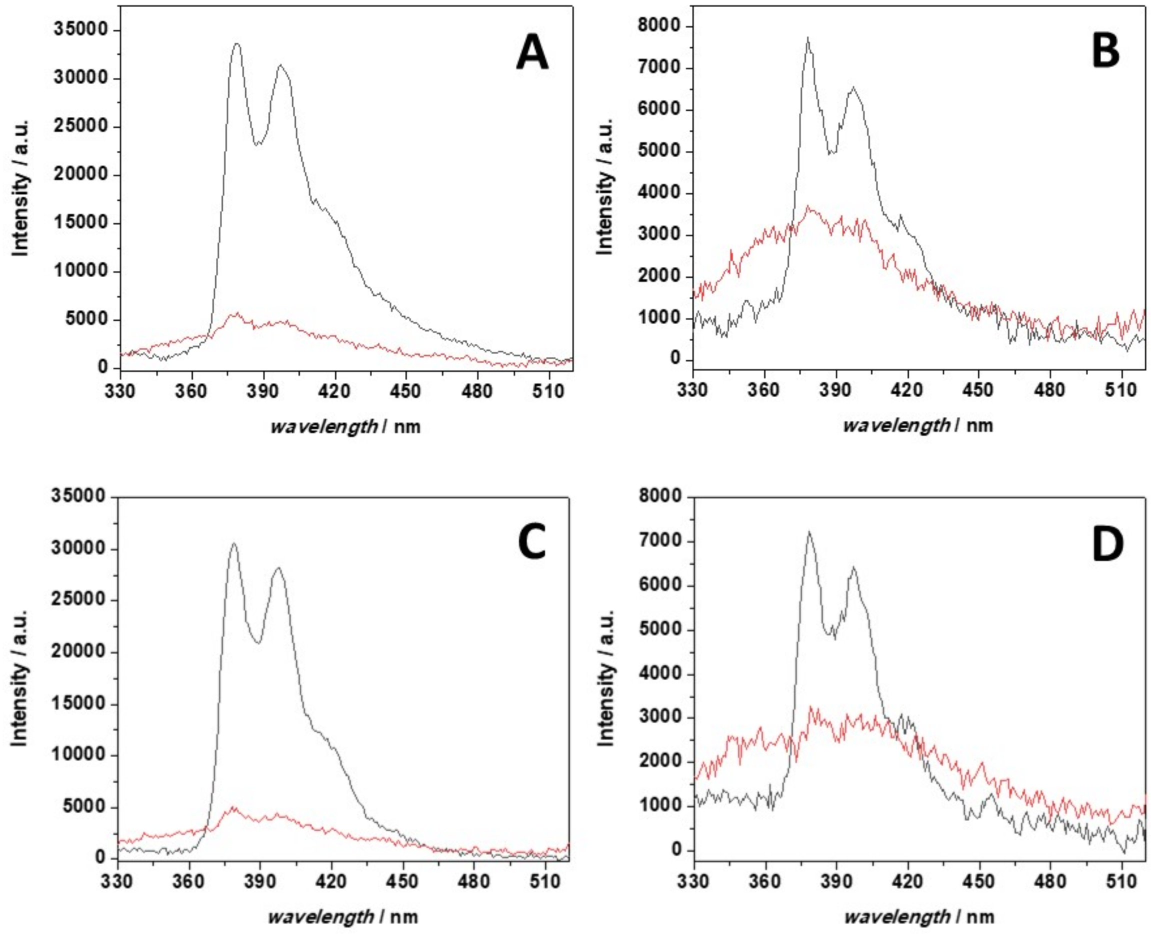

Figure S121. Fluorescence emission spectra of pyrene-labeled 6.4 µM ds DNA in the absence (black spectra) and in the presence (red spectra) of 210 µM copolymer micelle **IIB** for (A) **DNA-9**, (B) **DNA-10**, (C) **DNA-11** and (D) **DNA-12**. The spectra were recorded upon excitation at 273 nm at the temperature of 25 °C using a quartz cuvette with a path length of 0.3 cm.

8.5. Fluorescence Spectroscopy

Fluorescence spectroscopy spectra were acquired on a Hitachi F-2700 Fluorescence Spectrophotometer. The experiments were performed at 25 °C using a quartz cuvette with a volume of 5 mL and a path length of 1 cm. A dilution series from 0.5 mM to 0.00001 mM in water was prepared from the copolymers and a 0.1 mM solution of pyrene in methanol was added. The samples were then degassed with argon.

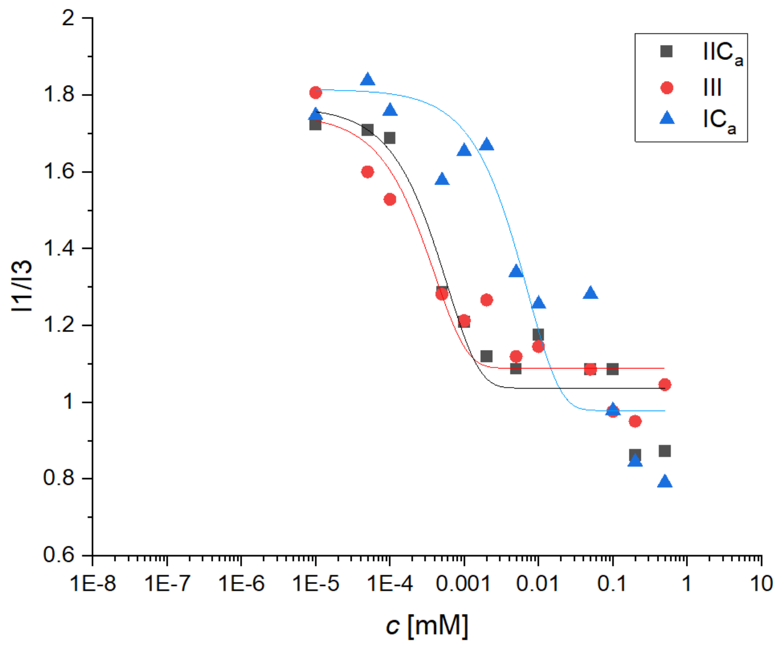

Figure S122. Fluorescence spectra of the core functionalized copolymer **IC$_a$** (blue), the shell functionalized copolymer **IIC$_a$** (black) and the unfunctionalized copolymer **III** (red).

9. Literature

1 C. Barner-Kowollik. *Handbook of RAFT Copolymerization*, VCH: Weinheim, **2008**.

2 C. Herfurth, P. Malo de Molina, C. Wieland, S. Rogers, M. Gradzielski, A. Laschewsky, *Polym. Chem.*, **2012**, *3*, 1606–1617.

3 M. Klika Škopić, K. Götte, C. Gramse, M. Dieter, S. Pospich, S. Raunser, R. Weberskirch, A. Brunschweiger, *J. Am. Chem. Soc.* **2019**, *141*, 10546–10555.

4 M. Potowski, F. Losch, E. Wünnemann, J. K. Dahmen, S. Chines, A. Brunschweiger, *Chem. Sci.* **2019**, *10*, 10481-10492.

5 D. Franke, M. V. Petoukhov, P. V. Konarev, A. Panjkovich, A. Tuukkanen, H. D. T. Mertens, A. G. Kikhney, N. R. Hajizadeh, J. M. Franklin, C. M. Jeffries, D. I. Svergun, *J. Appl. Crystallogr.* **2017**, *50*, 1212–1225.

6 a) R. Oliva, P. Del Vecchio, A. Grimaldi, E. Notomista, V. Cafaro, K. Pane, V. Schuabb, R. Winter, L. Petraccone, *Phys. Chem. Chem. Phys.* **2019**, *21*, 3989–3998. b) S. H. White, W. C. Wimley, A. S. Ladokhin, K. Hristova, *Methods in Enzymology*, **1998**, *254*, 62–87.
